# A mega-aggregation framework synthesis of the barriers and facilitators to linkage, adherence to ART and retention in care among people living with HIV

**DOI:** 10.1186/s13643-021-01582-z

**Published:** 2021-02-11

**Authors:** Lynn Hendricks, Ingrid Eshun-Wilson, Anke Rohwer

**Affiliations:** 1 Centre for Evidence-Based Health Care, Division Epidemiology and Biostatistics, Faculty of Medicine and Health Sciences, Stellenbosch University, Cape Town, South Africa; 2 Social, Methodological, Innovative, Kreative, Centre for Sociological Research, Faculty of Social Sciences, Katholieke Universiteit Leuven, Leuven, Belgium

**Keywords:** Overview, Mega-aggregation, Qualitative, Synthesis, Human immunodeficiency virus, Linkage, Adherence, Retention, ART

## Abstract

**Background:**

People living with human immunodeficiency virus (PLHIV) struggle with the challenges of living with a chronic disease and integrating antiretroviral treatment (ART) and care into their daily lives. The aims of this study were as follows: (1) to undertake the first mega-aggregation of qualitative evidence syntheses using the methods of framework synthesis and (2) make sense of existing qualitative evidence syntheses that explore the barriers and facilitators of adherence to antiretroviral treatment, linkage to care and retention in care for PLHIV to identify research gaps.

**Methods:**

We conducted a comprehensive search and did all screening, data extraction and critical appraisal independently and in duplicate. We used the Kaufman HIV Behaviour Change model (Kaufman et al., 2014) as a framework to synthesise the findings using the mega-aggregative framework synthesis approach, which consists of 8 steps: (1) identify a clearly defined review question and objectives, (2) identify a theoretical framework or model, (3) decide on criteria for considering reviews for inclusion, (4) conduct searching and screening, (5) conduct quality appraisal of the included studies, (6) data extraction and categorisation, (7) present and synthesise the findings, and (8) transparent reporting. We evaluated systematic reviews up to July 2018 and assessed methodological quality, across reviews, using the Joanna Briggs Institute Critical Appraisal Checklist for Systematic Reviews.

**Results:**

We included 33 systematic reviews from low, middle- and high-income countries, which reported on 1,111,964 PLHIV. The methodological quality of included reviews varied considerably. We identified 544 unique third-order concepts from the included systematic reviews, which were reclassified into 45 fourth-order themes within the individual, interpersonal, community, institutional and structural levels of the Kaufman HIV Behaviour Change model. We found that the main influencers of linkage, adherence and retention behaviours were psychosocial and personal characteristics—perceptions of ART, desires, fears, experiences of HIV and ART, coping strategies and mental health issues—interwoven with other factors on the interpersonal, community, institutional and structural level. Using this approach, we found interdependence between factors influencing ART linkage, retention and adherence and identified the need for qualitative evidence that explores, in greater depth, the complex relationships between structural factors and adherence, sociodemographic factors such as community violence and retention, and the experiences of growing up with HIV in low- and middle-income countries—specifically in children, youth, women and key populations.

**Conclusions:**

This is the first mega-aggregation framework synthesis, or synthesis of qualitative evidence syntheses using the methods of framework synthesis at the overview level. We found the novel method to be a transparent and efficient method for assessing the quality and making sense of existing qualitative systematic reviews.

**Systematic review registration:**

The protocol of this overview was registered on PROSPERO (CRD42017078155) on 17 December 2017.

**Supplementary Information:**

The online version contains supplementary material available at 10.1186/s13643-021-01582-z.

## Background

Qualitative evidence syntheses (QES) aggregate, integrate and interpret results from primary qualitative studies [1]. Like quantitative systematic reviews, QES follow transparent, systematic and rigorous methods. With the increase in number of QES on HIV adherence research [2], the next step is to provide an overview of existing systematic reviews to identify research gaps and an up-to-date synthesis of what is known. An overview is also referred to by names such as an umbrella review or a review of reviews. Methods of QES are complex and continue to develop [3]. Although there is guidance on summarising qualitative systematic reviews [4, 5], the application of the guidance to cases is still emerging in the literature, with examples of meta-summary of reviews [6, 7] and an application of mega-ethnography [8]. To consider evidence with the aim of assessing the quality of the existing evidence, identifying research gaps to formulate new research questions, or to make decisions about best practice, the appropriate QES method would be meta-aggregation. Meta-aggregation does not aim to produce deeper interpretative analysis of the data extracted from the primary studies but rather summarises findings to produce recommendations for action [9, 10]. Introducing an existing theory or framework [11] into this process can contribute to the efficiency, rigour and pragmatism of meta-aggregation. In this study, we illustrate mega-aggregation framework synthesis to make sense of existing qualitative systematic reviews.

### Exploring barriers and facilitators of antiretroviral therapy

Although access to HIV care has improved significantly over the past few years, people living with HIV (PLHIV) still face numerous challenges when it comes to initiating care and staying on treatment. Human immunodeficiency virus (HIV) represents one of the greatest global public health challenge in history, and since the beginning of the epidemic, approximately 78 million people worldwide have been infected with HIV and 35 million people have died [12]. The Joint United Nations Programme on HIV/acquired immunodeficiency syndrome (AIDS) (UNAIDS) set the global 90-90-90-target to combat HIV infection by 2020 [13]. The goal aims for 90% of all people to know their HIV status, of those who test positive, 90% should be linked to care, and of those being adherent to care, 90% will have achieved viral suppression. Nearly 37 million people were estimated to be living with HIV worldwide in 2017; however, only 60% were aware of their HIV status and only 49% of those who knew their status were accessing treatment [14]. The HIV burden varies considerably between countries, with regions in Africa having the highest HIV prevalence with HIV being the leading cause of death in South Africa [12]. To date there is no known cure for AIDS. However, being linked to care and adhering to antiretroviral therapy (ART) has been shown to improve quality of life for PLHIV, and in most cases provided similar life expectancy periods for those without HIV [15, 16].

### Why it is important to do this overview

PLHIV continue to be challenged by the complexities related to being HIV positive and integrating ART treatment and care into their daily lives. Unsuccessful interventions and the target driven 90-90-90 goals have increased researchers’ commitment to understanding the human experience of living with HIV and engaging in the HIV treatment cascade. Some reviews have focused only on prevention, adherence, linkage to care or retention in care. This can lead to an abundance of research in one area on the cascade and neglect of others. With the growing body of existing systematic reviews [17, 18], there is no coherent sense of what is already known across populations and settings, and there is uncertainty about the quality of the existing evidence. This can make it hard for policy makers and practitioners to make evidence-informed decisions. However, the increase in QES makes research innovation in the synthesis of qualitative review-level evidence possible. The overarching aim of this study is to debut the first application of mega-aggregation framework synthesis to qualitative systematic review-level evidence. This method of summarising QES in an overview will be applied to review-level evidence of barriers and facilitators of linkage to care, adherence to ART and retention in care for PLHIV in low-, middle- and high-income countries.

## Methods

### Paradigmatic stance

Overviews of reviews aim to provide a single synthesis or summary from multiple systematic reviews [19]. QES often have a theoretical underpinning to understand findings and interpret meaning. Qualitative research is usually positioned in the interpretive or critical-realist paradigm. Another approach to QES is meta-aggregation, which is based on the philosophy of pragmatism [20, 21] and users of this method aim for immediate usability of the review findings. This study proposes the application of mega-aggregation, which, unlike mega-ethnography, does not focus on the generation of new theory nor aims to provide deepened conceptual interpretations of findings [8], but rather aims to provide an overview of the existing evidence, identify evidence gaps and make recommendations for future research or immediate action [21, 22].

### Overview design

**Fig. 1:**
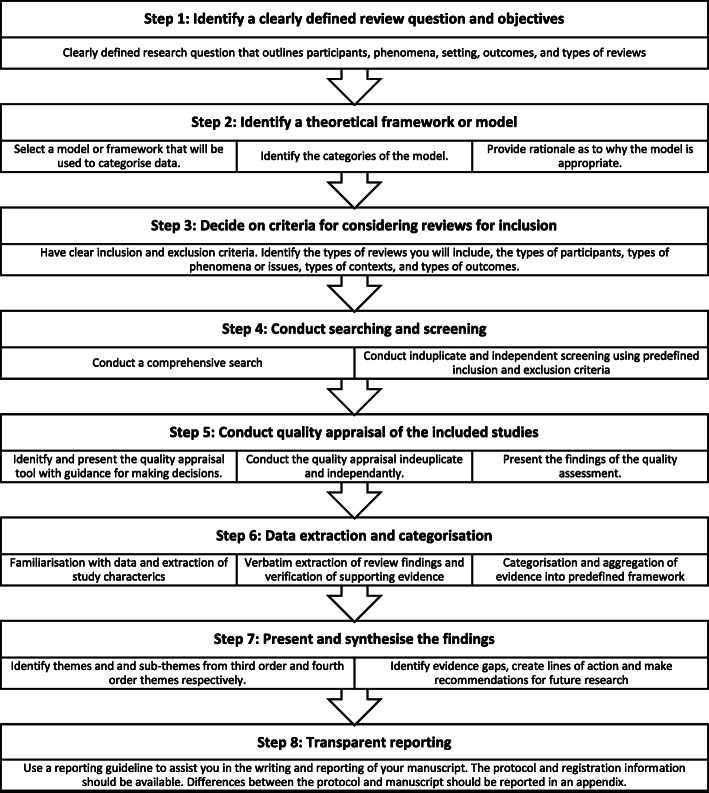
Steps of the overview using mega-aggregation framework synthesis of qualitative systematic reviews

In the context of the pragmatic stance and the anticipated large number of existing systematic reviews, a predetermined theoretical framework [23] with broad categories was selected to guide the aggregation and synthesis within this overview, which built on the steps in methods development for conducting overviews [24], QES [25, 26], systematic review synthesis [19, 27, 28], meta-aggregation [9, 20, 21] and framework synthesis [29, 30]. The novel approach of *mega-aggregation framework synthesis* was developed and utilised to identify evidence gaps and to inform future research from the evidence collated within included systematic reviews. The mega-aggregative framework synthesis approach consists of eight distinct steps (Fig. 1). The steps are as follows: (1) identify a clearly defined review question and objectives, (2) identify a theoretical framework or model, (3) decide on criteria for considering reviews for inclusion, (4) conduct searching and screening, (5) conduct quality appraisal of the included studies (although some may prefer not too), (6) data extraction and categorisation, (7) present and synthesise the findings, and (8) transparent reporting.

#### Step 1: identifying a clearly defined research question and objectives

This study aimed to answer the question:

What is the available review-level evidence of the self-reported barriers and facilitators to linkage, adherence to ART and retention in care, for people living with HIV in low-, middle- and high-income countries?

The primary objective was to gather, appraise and synthesise the systematic review-level evidence on the barriers and facilitators on behaviours related to ART among PLHIV using Kaufmans’ HIV Behaviour Change model [23]. The secondary objective was to identify evidence gaps for self-reported barriers and facilitators among PLHIV to create lines of action and make recommendations for future research, policy, and practice.

#### Step 2: identifying a theoretical framework or model

The complexities and interrelatedness of the factors influencing behaviour of PLHIV, including barriers and facilitators, can be found in multiple dimensions for linkage to ART [31], adherence to ART [32–34] and retention in care [35, 36]. The dimensions within which barriers and facilitators are understood in this overview are based on the Kaufman and colleagues HIV Behaviour Change Model [23]. The framework includes five broad domains, namely: (1) individual factors (includes factors such as knowledge, emotions, motivation, mental health, adverse drug reactions and comorbidities), (2) interpersonal and network factors (includes factors such as relationships, social networks and interpersonal violence, (3) community factors (includes factors such as stigma, peer pressure and cultural norms), (4) institutional and health system factors, (includes factors such as provision of services, service integration and relationships with health care workers), and (5) structural factors (includes factors such as poverty, political context and gender equity). This framework is useful to this overview as it provides comprehensive multi-level domains to understand the barriers and facilitators that PLHIV experience when they decide to link to ART, adhere to ART and engage in care consistently.

#### Step 3: criteria for considering systematic reviews for inclusion

*Types of reviews*

Systematic reviews were defined as those reviews that had predetermined objectives, predetermined criteria for eligibility, searched at least two data sources, of which one needed to be an electronic database, and performed standardised data extraction [37].

Systematic reviews were considered eligible if they included only qualitative studies. Reviews containing qualitative and quantitative studies were still considered eligible if outcomes were self-reported and a narrative description was used to summarise review findings. Systematic reviews only synthesising quantitative studies or only examining adherence pre- or post-exposure prophylaxis were excluded. No reviews were excluded based on whether quality assessments were conducted or not.

*Types of participants*

Eligible participants included children and adults living with HIV. Reviews were excluded if the primary sample of interest included more than 50% of the population who were not HIV positive. Although PLHIV were the target participants in this review, information obtained from health professionals and primary caregivers were considered if it pertained to perceptions of barriers and facilitators to linkage, adherence and retention in care for PLHIV. Children and youth referred to PLHIV up to the age of 24 years. Where included reviews specifically referred to the age group as youth, older than 12 years, we reported it as youth in our findings.

*Types of issues*

Eligible reviews addressed linkage to ART, adherence to treatment and retention in care of persons testing positive for HIV. Enrolment in antiretroviral therapy (ART) care following a positive HIV test is referred to as linkage to care in this overview. While no specific criterion exists with regard to linkage to care, it has been previously defined as one visit or more during the first 6 months of receiving a positive diagnosis and the initiation of antiretroviral treatment [31]. Adherence to ART refers to the extent to which a person who is HIV positive follows their prescribed regimen of care and takes their medication as they should [33, 34]. Since the introduction of ART, there has been a decline in AIDS-related deaths and life expectancy for those infected with HIV has increased [15]. Viral suppression is optimal when PLHIV have an adherence rate of 95% or more [16]. Retention in HIV care is described as constant attainment of the suitable medical care that includes attending follow-up appointments, medical tests or any other activity that was suggested by a healthcare practitioner to be maintained [35]. Reviews addressing the issues related to prevention including pre-exposure prophylaxis (PREP) and pre-ART care were excluded from this overview.

*Types of contexts*

Reviews synthesising information from high-, middle- and low-income countries were included in this overview. The geographic settings included rural and urban across all global regions.

*Types of outcomes*

The review-level outcomes of interest were self-reported barriers and facilitators to linkage to ART, adherence to ART and retention in care. Outcomes that were measured and reported using statistical associations between various factors and linkage, adherence and retention in care were not included.

#### Step 4: conduct searching and screening

A comprehensive search for systematic reviews up to 25 July 2018 was conducted in the Cochrane Library (specifically the CDSR and DARE), The Campbell Library, MEDLINE via PubMed, SCOPUS and CINAHL EBSCHOhost. PROSPERO was also checked for ongoing systematic reviews. Experts in the field were contacted and reference lists of included reviews were checked to identify further potential reviews for inclusion. An additional search on Google Scholar was conducted to search for reviews not contained within the databases. Key terms included in the search strategy were ‘HIV’, ‘linkage’, ‘adherence’, ‘retention in care’, ‘ART’, ‘qualitative’ and ‘systematic reviews’. Search terms were modified appropriately for the various databases. Detailed search strategies for all databases are reported in Additional file 1. No language, geographic or time restrictions were used in the search. Two authors (LH and AR), using Covidence [38], independently and in duplicate screened titles and abstracts of the records retrieved by the electronic searches for relevance; based on the participant characteristics, issues addressed, study design and outcomes. Full texts were retrieved for all potentially eligible reviews and were screened independently and in duplicate by two authors (LH and AR). Disagreements were recorded in Covidence [38], and these were resolved by consensus or through discussion with a third author (IEW). Reviews were categorised as included, ongoing, awaiting assessment or excluded with reasons.

#### Step 5: conduct quality appraisal of the included studies

Included systematic reviews were subjected to quality appraisal by the first author (LH) and second author (AR) independently and in duplicate. Discrepancies were resolved through discussion. Risk of bias was assessed using an amended version of the Joanna Briggs Institute Critical Appraisal Checklist for Systematic Reviews [39] (JBI-SR-Checklist) (Table 1). The JBI-SR-Checklist contains 11 guidance questions for the appraisal of systematic reviews. As this tool can be used for quantitative or qualitative reviews, we only considered those guidance questions that were appropriate for the assessment of qualitative reviews. Therefore, we omitted the question ‘Was the likelihood of publication bias assessed?’, as this was not applicable to this overview. Furthermore, we added a question that we thought was important to consider, namely ‘Was the screening and study selection appropriate?’. Each question was answered as ‘yes’, ‘no’ or ‘unclear’. The critical appraisal guide [39] provides key considerations for review authors when conducting appraisal. For the purpose of this overview, specific decision rules from the original JBI-SR-Checklist manual [39] were revised (Additional file 2) and clarified for making judgements about risk of bias, in order to ensure consistency between reviewers and across included reviews. No study was excluded based on the results of the quality assessment but rather it was used to identify weaknesses in study methodologies and to strengthen and inform the interpretation of the results of the systematic reviews.

**Table 1:** Revised Joanna Briggs Institute (JBI) 11-item checklist for systematic reviews

**Revised JBI systematic review checklist items** [39]	
1. Is the review question clearly and explicitly stated?*	
2. Were the inclusion criteria appropriate for the review question?*	
3. Was the search strategy appropriate?*	
4. Were the sources and resources used to search for studies adequate?*	
5. Was the screening and study selection appropriate?*	
6. Were the criteria for appraising studies appropriate?*	
7. Was critical appraisal conducted by two or more reviewers independently?*	
8. Were there methods to minimise errors in data extraction?*	
9. Were the methods used to combine studies appropriate?*	
10. Were recommendations for policy and/or practice supported by the reported data?	
11. Were the specific directives for new research appropriate?	

*Items used in the calculation of quality assessment score

We assessed the overall quality of systematic reviews as either low, medium or high, by considering items 1–9. Although the area of quality assessment in QES is still being debated in the field and the philosophical underpinning and epistemological reasoning behind conducting or not conducting quality assessment are unique to the rationale and question of the review authors [25], we included these in our assessment. We assessed items 10 and 11, but excluded them from our calculation for level of quality, as these questions do not relate to risk of bias, but rather to the validity of the findings, as stated in the JBI-SR-Checklist manual [39]. Additional File 2 explains how we made decisions about the overall quality of included reviews.

#### Step 6: data extraction and categorisation

The data extraction took place in two phases: (1) data extraction of characteristics of included studies and (2) data extraction of barriers and facilitators for data synthesis.

*Data extraction of characteristics of included reviews and their primary studies*

Data was extracted by the first author (LH) and checked and validated by a second author (AR) and third author (IEW) using a pre-specified piloted data extraction form in Microsoft Excel (Additional file 3). The extracted data included information on databases searched, date of the last search, what the reviews authors searched for and what they found in terms of types of studies, types of participants, the issue of interest, the setting or context, barriers and facilitators related to issues of interest. Details of critical appraisal tools, theoretical frameworks or models, methods of synthesis and limitations were also extracted. Information about the primary studies in the included systematic reviews were extracted, and these included the author names, year of publication, countries included and types of participants from primary source studies relevant to the overview, in order to describe the overlap of primary studies in systematic reviews included in the overview. Review authors were contacted for the full text papers if they were not available to the review team. Discrepancies in data extraction were discussed and once consensus was reached, the second phase commenced.

*Data extraction of barriers and facilitators for data synthesis*

The first author (LH) read the systematic reviews several times to become more familiar with the findings and recommendations made by the review authors. Following this, LH extracted barriers and facilitators verbatim into Excel for each review and categorised them according to the pre-specified dimensions of the Kaufman HIV Behaviour Change Model [23]. The review-level findings had to be supported by evidence such as references to the primary studies, direct quotes, visual or text evidence from the primary study, visual representations such as tables and figures with references to the primary studies, to be included in the extraction. The second author (AR) and third author (IEW) checked and validated the extracted barriers and facilitators in the Excel spreadsheet, and where discrepancies were raised, consensus was reached through discussion.

#### Step 7: present and synthesise the findings

The principles of meta-aggregation and framework synthesis were integrated to design and apply the novel approach of ‘*mega-aggregative framework synthesis*’ to this overview. Meta-aggregation is a method of data synthesis used in QES and focuses on aggregating primary-level findings into categories and then further aggregating those categories into synthetic statements that may be used for policy and practice without losing the critical interpretive value of the qualitative findings [21]. Mega-aggregation, which is a review-level higher, is a method of qualitative synthesis and aims to aggregate third-order review-level data into higher-order themes, called fourth-order themes with the purpose of identifying the scope of the available review-level evidence and make recommendations for research, policy and practice. In keeping with recent guidelines in selection of approaches for meta-synthesis and the large number of existing reviews available on the topic of this overview, a framework was applied to the mega-aggregative approach. Using a broad framework in mega-aggregation is useful for categorising the themes and findings of systematic review papers which, although may have included various qualitative designs, consider the same objective or issue and outcomes.

As with mega-ethnography [8], this type of synthesis considers first-order constructs (from the person), second-order constructs (interpretations of the author in a primary study), third-order constructs (findings in a systematic review) and fourth-order constructs (findings in an overview of reviews). Using the extracted third-order constructs in the framework dimensions, we then discussed and created fourth-order themes. The third-order concepts were coded into fourth-order concepts further categorised into the appropriate framework dimensions for each of the outcomes. We were then able to review the tables and identify evidence gaps and lines of action to inform future research, policy and practice. The overall number of findings contributing to each of the fourth-order themes of the overview was examined, and the most emergent (meaning the fourth-order themes with the most findings) barriers and facilitators, across included systematic reviews, were discussed in the manuscript. Evidence of all findings are presented in in-text tables and within the additional files of the manuscript. Additionally, we identified the evidence gaps and explored the gaps by country income classification, population group and fourth-order themes. Further detail on the application of mega-aggregation framework synthesis to this overview is provided in Additional file 4.

#### Step 8: transparent reporting

This overview used guidance from the Johanna Briggs Institute Methodology for Umbrella Reviews [39] and the PRISMA Extension for Scoping Reviews Checklist (PRISMA-ScR) [40] (Additional file 5). The protocol [17] pertaining to this overview was registered on PROSPERO (CRD42017078155) on 17 December 2017. Differences between the protocol and the manuscript are reported in Additional file 6.

## Results

### Overview of the search results

**Fig. 2:**
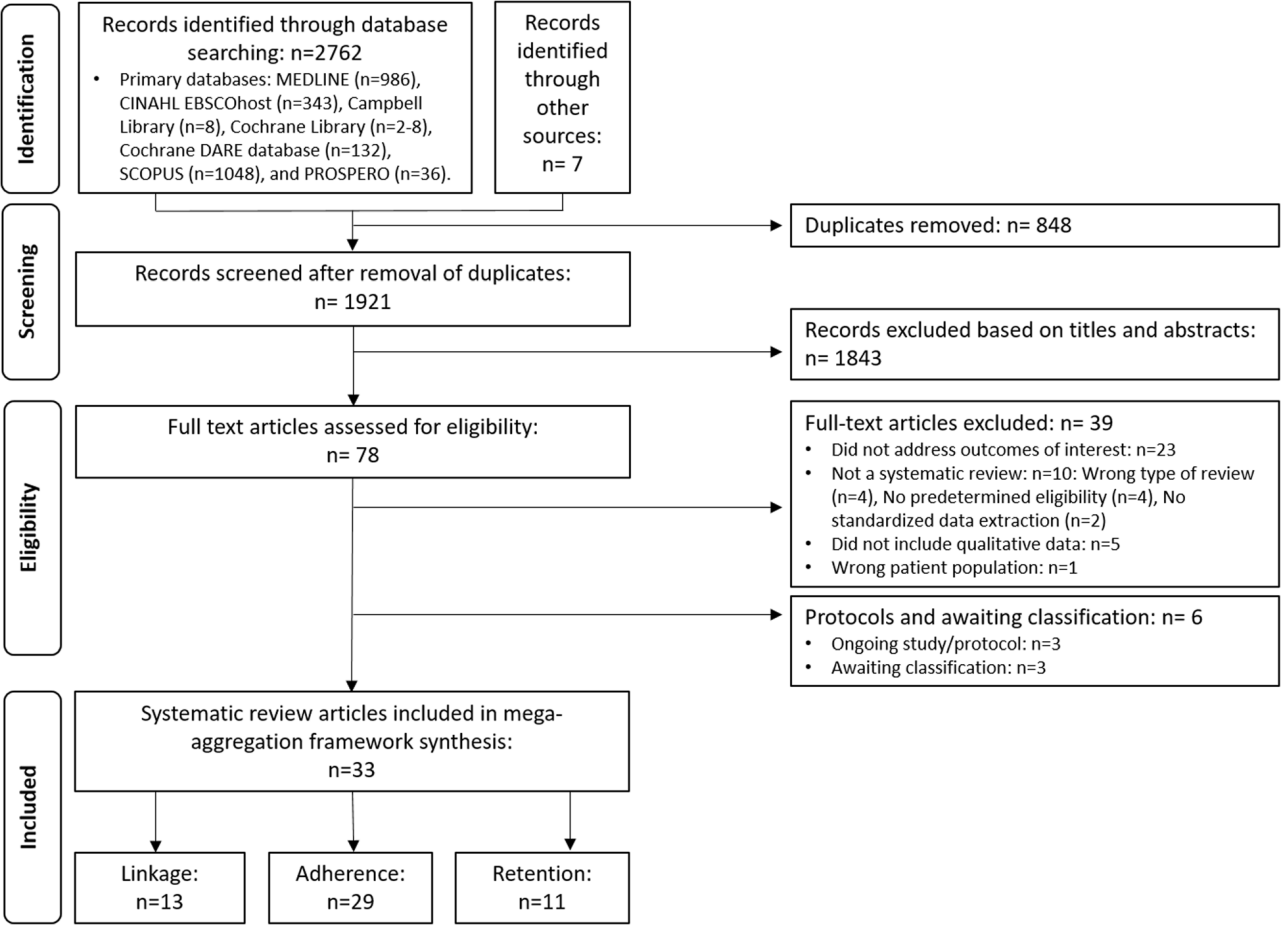
PRISMA flowchart

The database search resulted in 2762 article citations and an additional seven reviews were identified through other sources (two within the reference lists of included reviews and five through other readings). After the removal of duplicates, 1921 citations were imported into Covidence and the title and abstracts were screened, resulting in 78 retrieved for full text review. Thirty-nine reviews were excluded (Additional file 7), most reviews did not fit the criteria of a systematic review (*n* = 10), did not contain qualitative primary studies or data (*n* = 4) or did not include the target population group (*n* = 1). We were unable to obtain the full texts for two reviews and are waiting on information from one author and have classified these three reviews as ‘awaiting assessment’. Three ongoing reviews or protocols were found in our search (Additional file 7). We included 33 [41–73] systematic reviews in this overview. Figure 2 describes the flow of reviews through the different stages of this overview using the PRISMA flow diagram [74].

### Description of the systematic reviews included in the overview

**Fig. 3:**
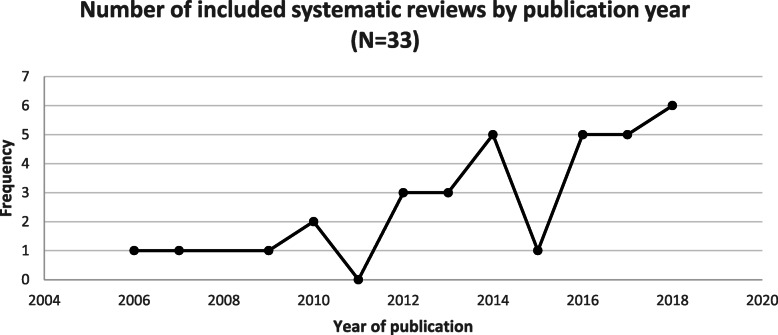
Number of included systematic reviews by publication year

Included systematic reviews were published between 2006 to June 2018, peaking at 6 publications in 2018 (Fig. 3).

**Fig. 4:**
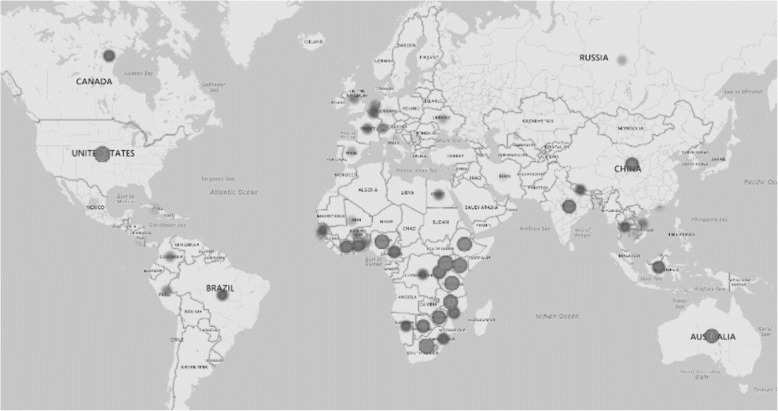
Distribution of countries included in the included systematic reviews (*N* = 33)

The included reviews (*N* = 33) synthesised primary studies that were conducted in both high-income countries and low- and middle-income countries with a large concentration of included primary studies being conducted in sub-Saharan Africa (Fig. 4). No reviews written in languages other than English were found.

Self-reported barriers and facilitators of 1,156,540 PLHIV (children and youth, and adults) are included in this overview. Some reviews included high-risk populations, such as pregnant and postpartum women, children and adolescents, commercial sex workers, men who have sex with men, transgender persons, prisoners, intravenous drug users and foreign nationals. Two reviews on children and adolescents included data from caregivers. Table 2 summarises the characteristics of included studies.

**Table 2:** Table of included studies (*N* = 33)

First author, year of publication [reference]	Search dates	Participants	Issue	Context		Types of studies	Method of synthesis	Overall quality of review
				Low- to middle-income country	High-income country			
**Ammon, 2018** [41]	3 June 2016 to 15 August 2016	*N =* 3145 participants: 2937 adolescents aged 10-19; 191 caregivers (parents, non-parental caregiver, biological relative, non-relative, or foster-carer) and 17 healthcare providers. Some adolescents living with HIV did not know about their HIV-positive status.	Adherence	Sub-Saharan Africa: *n =* 1 study each from Congo DRC, Ghana, Kenya, Rwanda, South Africa, Zambia, Zimbabwe, and *n =* 2 studies from Uganda.	None	11 studies: Qualitative (7), Quantitative (1) and Mixed Methods (3)	Thematic synthesis	Medium
**Barroso, 2017** [42]	2008 to 2013	*N =* 6189 participants: *n =* 4830 PLHIV (2197 female and 1850 male, 783 unspecified) and *n =* 1359 included provider participants (caregivers, health care providers, traditional healers, local community leaders, pharmacists, policymakers, stakeholders, peer counsellors, facility managers, volunteers, and clinical trial coordinators).	Linkage Adherence	China (5), Nigeria (5), South Africa (19), Tanzania (8), Uganda (16), and Zambia (9). All other locations for data collection contributed to fewer than five reports (Countries not reported)	Europe (9), US (28)	127 studies: Qualitative (127)	Thematic synthesis	Low
**Bolsewicz, 2015** [43]	2003 to 2013	PLHIV, excluding drug users, mothers, adolescents, prisoners, sex workers in Canada, UK, and Australia	Linkage Adherence	None	Canada (8), UK (3) and Australia (6)		Thematic synthesis	Medium
**Bravo, 2010** [44]	1990 to November 2009	*N =* 4215 PLHIV including drug users and women caring for children < 18 years; *n =* 4022 in Quantitative and *n =* 193 Qualitative studies.	Linkage Adherence	Botswana (1)	US (7), UK (1), France (1)	10 studies: Qualitative (5) and Quantitative (5)	Thematic meta-analysis	Low
**Chop, 2017** [45]	Up to 18 February 2018	Women living with HIV	Adherence	Zambia (1), Swaziland (1) and Democratic Republic of Congo (1)	France (1)	4 studies: Qualitative (3) and Quantitative (1)	Thematic analysis	Low
**Colvin, 2014** [46]	1 January 2008 to 26 March 2013	*N =* 875 308 participants: HIV-infected pregnant and/or postpartum women and/or health care providers delivering antenatal care, ART and/or PMTCT. A few studies included partners and/or family members.	Linkage Adherence Retention	Sub-Saharan Africa (38), Latin America (2), and Asia (2)	None	42 studies: Qualitative (14), Quantitative studies (25) and Mixed Methods (3)	Narrative meta-synthesis	Medium
**Croome, 2017** [47]	2005 to 24 May 2016	*N =* 37175 Adult PLHIV	Adherence	Benin, Cote d'lvoire and Mali (1), Botswana (3), Burkina Faso (1), Cameroon (4), Cote d'lvoire (1), DRC (2), Ethiopia (20), Ethiopia and Uganda (1), Ghana (4), Guinea-Bissau (1), Kenya (16), Kenya and Malawi (1), Kenya and Uganda (1), Lesotho (1), Malawi (2), Mali (1), Mozambique (3), Namibia (4), Nigeria (13), Nigeria, Tanzania and Uganda (1), Rwanda (3), Senegal (1), South Africa (30), Tanzania (10), Tanzania, Uganda and Zambia (1), Togo (1), Uganda (19), Zambia (6), Zimbabwe (2)	None	154 studies: 83 Qualitative (83), Quantitative (67) and Mixed methods (4)	Thematic content analysis	Low
**Engler, 2018** [48]	1996 to 10 March 2016	*N =* 1482 adult PLHIV (including men, women, men who have sex with men (MSM), intravenous drug user (IDU)	Adherence	None	US (35), Europe (3) (Switzerland, the Netherlands and Belgium), and Canada (2).	40 studies: Qualitative (40)	Thematic analysis	Low
**Ferguson, 2012**^(^ [49]^)^	1st January 2000 to 31st December 2010	*N =* 819 Pregnant women with HIV. Not all studies included reported sample size.	Retention	Kenya (1), South Africa (1), Tanzania (1), Zimbabwe (1), Malawi (2), Uganda (1)	None	7 studies: Qualitative (3) and Quantitative (4)	Thematic content analysis	Low
**Flores, 2018** [50]	2008 to 2013	*N =* 3257 participants: 2263 PLHIV (740 men, 1008 women, 78 transgender individuals and 437 people with unspecified gender). 994 other people were included in the studies such as family members, friends, physicians, nurses, treatment advocates, caregivers, clinic staff, programme directors, social workers, and other key stakeholders.	Linkage Retention	South Africa (9), Uganda (6), Nigeria (4), Zimbabwe (4) and China (4); 20 = unspecified	US (22 reports)	69 studies: Qualitative (69)	Thematic meta-synthesis	Low
**Gaston, 2013** [51]	1 January 2001 to 31 May 2012	African Americans LHIV Total *n =* 2846	Adherence	None	USA (16)	16 Studies: Qualitative (6) and Quantitative (10)	Thematic analysis	Low
**Geter, 2018** [52]	January 2005 to December 2016	African American females living with HIV Total *n =* 830	Adherence Retention	None	US (14)	14 studies: Qualitative (10) and Quantitative (4)	Thematic content analysis	Low
**Govindasamy, 2012** [53]	01 January 2000 to 31 May 2011	PLHIV in sub-Saharan Africa and health care workers.	Linkage	South Africa (6), Uganda (6), Kenya (2), Tanzania (2), Zambia (2), and 1 study each from Ethiopia, Swaziland, Mozambique, and South Africa and Zimbabwe.	None	21 Studies: Qualitative (11), Quantitative (7) and Mixed Methods (3)	Thematic content analysis	Low
**Heestermans, 2016** [54]	January 2002 to 27 October 2014.	161 922 Adult PLHIV	Adherence	Sub-Saharan Africa		146 studies: Qualitative (37), Quantitative (112) and Mixed methods (3)	Narrative synthesis	Low
**Hodgson, 2014** [55]	1st January 2008 to 26 March 2013	Pregnant women and postpartum women infected with HIV	Linkage Adherence Retention	Ghana (1), Nigeria (1), Malawi (5), South Africa (6), Zimbabwe (2), Tanzania (2), Kenya (5), Uganda (3), Brazil (1), Rwanda (1), Zambia (1), Latin America (1)	Australia (1), US (3), France (1),	34 studies included in the review: Qualitative (12), Quantitative (16) and Mixed Methods (6)	Thematic analysis	Medium
**Katz, 2013** [56]	Up until February 2013	PLHIV between 18 and 30 years old, providers of HIV care, single persons and those in intimate partnerships and persons with and without children. High-risk groups including men who have sex with men, injecting drug users and commercial sex workers.	Adherence	Uganda (9), South Africa (5), India (2), and 1 study each from DRC, Brazil, Botswana, Tanzania, Thailand, Egypt, Ethiopia, Vietnam, Nepal, Nigeria, Asia, Zambia, and China. Four countries were not reported.	US (1)	75 Studies: Quantitative (41) and Qualitative (34)	Meta-ethnography	Low
**Knettel, 2018** [57]	January 2012 to June 2017	736 Pregnant and postpartum women on option B+.	Retention	Malawi (13), Uganda (4), Zimbabwe (3), Mozambique (2), and 1 each from Cameroon, Ethiopia, Rwanda, South Africa, and Tanzania	None	13 Studies: Qualitative (13)	Thematic analysis	Low
**Lancaster, 2016** [58]	Up to 22 November 2013 and a second search up to 30 July 2015	*N =* 2721 Female sex workers living with HIV	Linkage Adherence	Rwanda (1), Zimbabwe (2), Benin (2), Burkina Faso (1), Nigeria (1), Swaziland (1), Kenya (1), and Uganda (1).	None	10 studies: Qualitative (3), Quantitative (6) and Mixed Methods (3)	Thematic analysis	Low
**Lankowski, 2014** [59]	Databases up until August 2011 and abstracts from 2002 to 2004 and from 2006 to 2011.	*N =* 69 506 Adults and children LHIV, HIV-infected HCW, HC Providers, HIV-infected rape victims, pregnant and postpartum women with HIV.	Linkage Adherence Retention	Uganda (10), Kenya (3), Zambia (2), Malawi (4), Nigeria (3), Corte d'Ivoire (1), Botswana (4), Tanzania (4), Togo (1), Ethiopia (1), South Africa (2), The Gambia (1), Namibia (1)	None	34 studies: Qualitative (16) and Quantitative (18)	Content analysis	Low
**Lazuardi, 2018** [60]	1990 to 2016	PLHIV: including injecting drug users, pregnant women, MSM, transgendered people, women, men, and sero-discordant couples. Found information related to service providers, community members, TB patients, caregivers, and community organisers.	Linkage Adherence Retention	Indonesia (11)	None	11 studies: Qualitative (11)	Thematic analysis	Low
**Li, 2016** [61]	1 January 2000 to 21 February 2015	Total: *N =* 192434 PLHIV including adults, children, adolescents, pregnant and postpartum women, and caregivers.	Adherence	Botswana, Tanzania and Uganda (1), Peru (1), Ukraine (1), Zambia (1), Rwanda (1), Ethiopia (1), Uganda (1), Nepal (2), Cuba (1), Southern Malawi (1), Uganda and Zimbabwe (1), China (2), Tanzania (3), South Africa (3)	US (14), Netherlands (1), Canada (1), Australia (1), Belgium and Netherlands (1), Switzerland (1)	39 studies: Qualitative (39)	Thematic analysis	Medium
**Lytvyn, 2017** [62]	1 January 2000 to 11 February 2017	*N =* 1165: Women considering pregnancy (140), pregnant women (408), and postpartum women (602). Couples desiring and/or intending to have children (15) also included.	Adherence	Puerto Rico (1), Nigeria (1), Kenya (2), Swaziland (2), Malawi (2), India (1), South Africa (1), Zimbabwe (1), and	Australia (1), US (3)	15 Studies: Qualitative (15)	Meta-ethnography	High
**Merten, 2010** [63]	2000 to 2008	*N =* 2044+ Community members, policy makers, PLHIV, health workers, female HIV+ patients, healthcare actors, In-school and out-of-school youth, patients who attended the ARV clinic, counsellors, HIV+ patients on ART for 6 months, care givers, family care givers, key informants, HIV+ patients from IDP camps, treatment partners	Adherence	Uganda (6), Zambia (5), South Africa (6), Burkina Faso (1), Malawi (2), Tanzania (5), Botswana (2), Kenya (1), Nigeria (1), Ethiopia and Uganda (1), Burkina Faso, Cote d'Ivoire and Mali (1), Nigeria, Tanzania and Uganda (1)	None	32 studies: Qualitative (32)	Meta-ethnography	Low
**Mey, 2016** [64]	January 2000 to 15 December 2015	PLHIV, Men, women, MSM, caregivers of children who are HIV positive, CAM workers (traditional healers/alternative medicines)	Linkage Adherence Retention	None	Australia (21)	35 Studies: Qualitative (14), Quantitative (14), Mixed Methods (6), and Case Report (1)	Narrative synthesis	Low
**Mills, 2006** [65]	Up to June 2005	PLHIV and caregiver Total: *N =* 12902	Adherence	12 studies were conducted in developing countries included four from Brazil and one each from Uganda, Cote d’Ivoire, South Africa, Malawi, Botswana, Costa Rica, Romania, and China.	US (56), Canada (3), UK (3), Italy (2), France (2), The Netherlands (2), Australia (1), Belgium (1) and Switzerland (1). The studies conducted in developing countries included Brazil (1) and Botswana (1) Two studies were multi-national: (countries not reported).	84 studies: Qualitative (37) and Quantitative (47)	Content analysis	High
**Morales-Aleman, 2014** [66]	Jan 2002 to April 2013	*N =* 121 Hispanic and Latino PLHIV	Linkage Adherence Retention	None	USA (4)	3 studies: Qualitative (3) and Quantitative (1)	Thematic analysis	Low
**Omonaiye, 2018** [67]	Up to December 2017	HIV-positive pregnant women (include number)	Adherence	Kenya (3), Swaziland (1), Uganda (2), South Africa (1), Cote d'voire (2), Tanzania (1), Malawi (4), Mozambique (1)	None	15 Studies: Qualitative (9) and Mixed Methods (6)	Thematic content analysis	Medium
**Reisner, 2009** [68]	1999 to 2008	*N =* 5179 HIV-positive youth and adolescents and pregnant adolescents.	Adherence	None	US (14)	14 Studies: Qualitative (4), Quantitative (7) and Mixed Methods (3)	Thematic content analysis	Low
**Santer, 2014** [69]	1996 to 2011	*N =* 96 Caregivers of children aged 0 -18 years	Adherence	None	Belgium (1) and US (2)	3 Studies: Qualitative (3)	Thematic analysis	Low
**Vervoort, 2007** [70]	1996 to 2005	*N =* 1053 Adult PLHIV	Adherence	Not specified	Not specified	24 studies containing qualitative data.	Thematic content analysis	Low
**Vitalis, 2013** [71]	Up to July 2011	HIV-positive pregnant and postpartum women between the ages of 12 to 58 years receiving ART.	Adherence	Africa (7), Brazil (2) and Puerto Rico (1)	USA (8), and Australia (1)	18 studies: Quantitative (15) and Qualitative (3)	Content analysis	Low
**Wasti, 2012** [72]	1996 to 2010	*N =* 4782 Adult PLHIV who have been prescribed ART. Quantitative Studies *n =* 4372; qualitative studies *n =* 152 and mixed methods studies *n =* 258	Adherence	India (10), China (4), Thailand (3), Cambodia (1).	None	18 studies: Quantitative (12), Qualitative (4) and Mixed Methods (2)	Thematic analysis	Low
**Williams, 2018** [73]	January 2005 to March 2016	Adolescent ages 9-20 years living with HIV	Linkage Adherence Retention	Zimbabwe (2), South Africa (3), Kenya (3), Botswana (1), Zambia (3), Tanzania (1), Uganda (1), Uganda and Zimbabwe (1), Tanzania (2), and Botswana and Tanzania (1)	None	18 studies: Qualitative (18)	Meta-ethnography	Low

We applied the conceptual definitions of the outcomes as per the overview protocol and we found that 13 reviews addressed the outcome of linkage to ART, 29 addressed the outcome of adherence to ART and 11 addressed the outcome of retention in care (see Fig. 2). The method of synthesis of the reviews varied and included thematic analysis, thematic content analysis, content analysis, narrative synthesis, meta-synthesis and meta-aggregation. Details of data are extracted, and critical appraisal of each included review is available in Additional file 8.

Due to the different ways of reporting results in the included reviews, we discerned between two population groups in terms of age, children and youth, and adults.

### Overlap between included systematic reviews

We found overlap in the qualitative, quantitative and mixed methods primary studies included within the 33 systematic reviews (Additional file 9). Review authors used varying definitions of linkage to ART, adherence to ART and retention in care when considering studies for inclusion. One author may have used a primary study to synthesise evidence on linkage to ART and another author may have used the same study to synthesis evidence on adherence to ART. The primary studies within the systematic reviews were published between and 1995 and 2017. Of the 1153 primary studies in the systematic reviews, 826 were unique studies, of which 616 were included in only one review. We found that 139 of the studies were included in two reviews, forty-seven in three reviews, fourteen in four reviews, three in five, four in six, and one study was included in seven reviews and another across eight reviews.

**Fig. 5:**
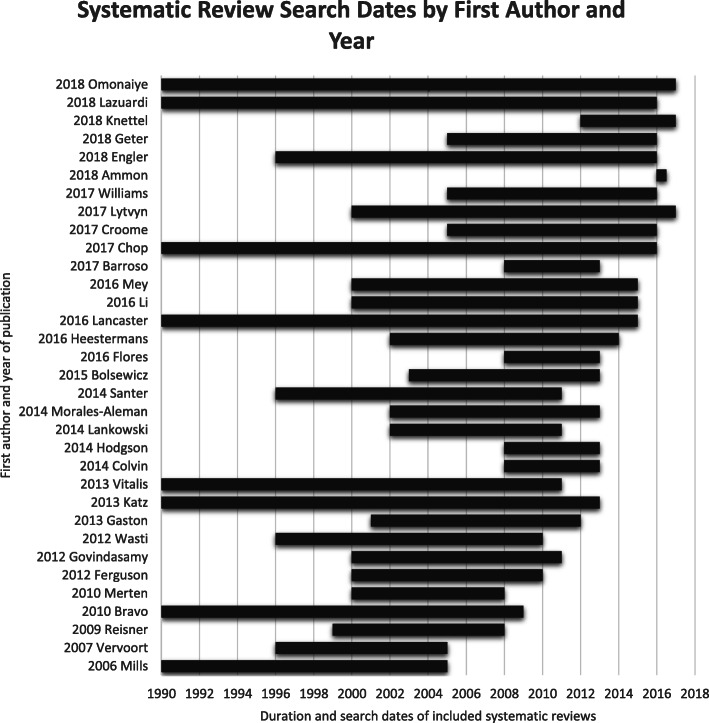
Overlap between search dates of included systematic reviews (*N* = 33)

We explored whether there was overlap in the search dates between the included reviews and found that most reviews searched between 2000 and 2013 with an average search period covering 13 years (Fig. 5). Eight studies [44, 45, 56, 58, 60, 65, 67, 71] conducted comprehensive searches up to a year before publication. One review [41], conducted as part of an online postgraduate degree programme, had very short search period of 6 months. We found considerable overlap in the search dates of included systematic reviews.

### Quality assessment of systematic reviews included in the review

The methodological quality of the included systematic reviews varied across reviews. Details of the justifications for quality judgements are reported in Additional file 8. All but five reviews had clear research questions. Key methodological aspects that were appraised as good quality were the relevance of recommendations for policy, having a clear research question and relevant directives for future research. The key aspects that were assessed as poor for the included reviews were the sources used to search and the inclusion criteria of the reviews. Some reviews did not clearly report items, and as we were thus unable to make a judgement, we assessed them as ‘unclear’. Items concerning if the ‘process of data extraction was appropriate’ and if the ‘critical appraisal was conducted by two or more authors’ were mostly identified as ‘unclear’ across included reviews. We identified 110 (30.3%) items out a possible 363 as ‘unclear’, 70 (19.3%) items as ‘no’ and 183 (50.4%) of items as ‘yes’. Two reviews [62, 65] were rated as high quality, six reviews [41, 43, 46, 55, 61, 67] were rated as medium quality and 25 reviews [42, 44, 45, 47–54, 56–60, 63, 66, 68–73, 75] were rated as low quality (Table 3).

**Table 3:** Critical appraisal

Included reviews: first author and year	JBI Critical Appraisal Questions											
	1. Review question clear	2. Inclusion criteria appropriate	3. Search strategy comprehensive	4. Sources and resources	5. Selection of studies	6. Appraisal criteria	7. Critical appraisal conducted in duplicate	8. Methods to minimise error in data extraction	9. Methods to combine studies	10. Recommendations for practice	11. Directives for research	Overall Quality
**Ammon 2018** [41]	+	+	+	?	?	?	?	?	+	+	−	Medium
**Barroso 2017** [42]	+	+	+	−	?	−	−	+	+	?	−	Low
**Bolsewicz 2015** [43]	+	+	+	+	?	?	?	?	+	+	+	Medium
**Bravo 2010** [44]	+	+	−	−	?	?	?	?	+	+	+	Low
**Chop 2017** [45]	+	+	?	−	−	−	?	+	?	+	+	Low
**Colvin 2014** [46]	+	+	+	+	?	?	?	+	+	+	+	Medium
**Croome 2017** [47]	+	+	+	+	−	+	−	?	+	+	+	Low
**Engler 2018** [48]	+	−	+	−	−	−	−	−	+	+	+	Low
**Ferguson 2012** [49]	+	+	−	+	+	?	?	−	?	+	+	Low
**Flores 2016** [50]	+	−	+	−	?	−	−	?	+	+	+	Low
**Gaston 2013** [51]	+	+	?	−	−	?	?	?	+	+	+	Low
**Geter 2018** [52]	+	+	?	−	?	?	?	?	+	+	+	Low
**Govindasamy 2012** [53]	+	+	−	+	+	−	−	?	?	?	+	Low
**Heestermans 2016** [54]	+	?	+	−	?	+	?	−	?	+	?	Low
**Hodgson 2014** [55]	+	+	−	+	+	?	?	+	+	+	+	Medium
**Katz 2013** [56]	−	−	?	+	?	+	?	?	+	+	+	Low
**Knettel 2018** [57]	+	−	+	−	+	?	?	+	+	+	+	Low
**Lancaster 2016** [58]	+	+	?	−	+	?	?	+	?	+	+	Low
**Lankowski 2014** [59]	+	+	−	−	−	−	?	−	−	+	?	Low
**Lazuardi 2018** [60]	+	+	?	+	?	?	?	−	?	+	+	Low
**Li 2017** [61]	+	+	−	+	+	+	+	?	+	+	+	Medium
**Lytvyn 2017** [62]	+	+	+	+	+	+	?	?	+	+	+	High
**Merten 2010** [63]	−	?	−	+	−	?	?	?	+	?	?	Low
**Mey 2017** [64]	+	−	+	−	−	+	?	−	+	?	+	Low
**Mills 2006** [65]	+	+	?	+	?	+	+	+	+	+	+	High
**Morales-Aleman 2014** [66]	−	−	?	−	?	?	?	?	+	+	+	Low
**Omonaiye 2018** [67]	+	+	?	−	+	+	+	+	?	+	?	Medium
**Reisner 2009** [68]	?	−	?	−	?	?	?	?	?	+	+	Low
**Santer 2014** [69]	+	−	?	+	?	+	+	?	+	+	−	Low
**Vervoort 2007** [70]	−	+	+	−	?	?	?	+	+	+	?	Low
**Vitalis 2013** [71]	+	+	?	−	?	+	?	?	?	−	+	Low
**Wasti 2012** [72]	−	−	+	−	?	?	?	+	−	−	?	Low
**Williams 2017** [73]	+	+	?	−	?	+	+	?	+	+	+	Low

### Data categorisation: what is the available review-level evidence on barriers and facilitators to linkage, adherence, and retention in care?

We found 544 unique third-order concepts from the included systematic reviews and to retain the essence of the review authors’ interpretations, we extracted concepts verbatim. We then categorised and aggregated the evidence into the predetermined framework, namely, Kaufman’s Behaviour Change Model of HIV [23].

#### Barriers and facilitators to linkage to ART

Barriers and facilitators to linkage to ART were found on all levels of the Kaufman framework (Additional file 10) and contributed to the synthesis of the barriers and facilitators to linkage to ART. One low-quality review [73] contributed to the findings on linkage for children. For adults, findings for linkage, were aggregated from one high-quality review [65], three medium-quality review [43, 46, 55], and eleven low-quality reviews [42, 44, 45, 49, 50, 53, 54, 58, 60, 64, 66].

On the *individual level*, participants reported barriers linked to sociodemographic factors (5 findings), such as being younger, whether the participant’s occupation was considered socially acceptable, gender, and not having identification documents in order to enrol in care services. Barriers related to patient fears (9 findings) were the emergent themes. PLHIV expressed fears of the consequences of disclosure, such as job loss, stigma and social isolation, fears of being on lifelong treatment and the negative side effects of ART. PLHIV reported experiencing psychological distress and emotional reactions (9 findings) and some were shocked at the news of their positive status, unsure about how they had contracted the disease, and the possibility of infidelity in their relationships. Feelings of hopelessness and depression were a recurring theme, with women questioning their self-esteem as wives and mothers. For children, two findings of barriers to linkage included negative emotions and self-perception.

PLHIV doubted their ability to adhere and commit to lifelong treatment and care. Themes for the facilitators of linkage to ART on the individual level included physical health (6 findings) and barriers regarding physical health (8 findings). In the context of participants’ psychological distress, some reviews found that participants could no longer ignore the physical symptoms of the disease or their declining health, while others found that although they tested positive for HIV, they were asymptomatic, and therefore delayed care. The desire to care for family, protect unborn children from the transmission of HIV, as well as the desire for future marriage and children facilitated children’s linkage.

On the *interpersonal level*, relationships in the household emerged as an important theme, both as barriers (7 findings for adults and 1 finding for children) and facilitators (3 findings for adults and 1 finding for children). PLHIV reported conflicts in the household, threats of domestic violence and abandonment, and the lack of autonomy for women, as barriers to linkage to ART. In contrast, supportive partners and families with mutuality-fostering relationships involving empathy facilitated linkage.

On the *community level*, the main barrier expressed was stigma and discrimination (4 findings), which is linked to community narratives around masculinity, HIV as witchcraft, and hospitals as places of death. Children reported unsupportive teachers (1 finding) as a barrier to linkage. Facilitators reported included peer support and support groups (6 findings) which served as a proxy for family support when it was lacking. One finding for adults included community beliefs and practices as a barrier with negative beliefs about ART, detrimental gender norms, and a preference for traditional healers and medicines.

At the *institutional level*, barriers such as stigma experienced at health care facilities (7 findings), service delivery (24 findings), which includes overcrowding, long queues, high staff turnovers, inconvenient client times, poor resources and participants’ experiences with limited medication availability as well as their experiences of HIV testing were reported. Four findings were related to barriers of institutional models of care. PLHIV identified gaps in the ART cascade referral process, particularly for women who test positive during their antenatal care (ANC) and are not followed-up postpartum, as well as lack of integrated services. PLHIV perceived health care models such as home visiting, as a barrier, as it might contribute to involuntary disclosure. There were fourteen findings for facilitators in the theme models of care, including offering population-specific services for adolescents, the integration of HIV care within ANC, offering mental health assessments and providing multi-level, multi-pronged approaches to care. The facilitators for the theme service delivery (2 findings) included PLHIV having positive experiences of HIV testing and encountering a clinic staff member who welcomed people into the clinic. Counselling practices and principles (6 findings) that respected the place of traditional medicine, incorporated the traditional beliefs of people, and that provided in-depth counselling before and after HIV testing were reported as facilitators on the institutional level. Children reported lack of privacy experiences at the clinic, the physical environment at the clinic and high staff turnover as barriers to linkage (1 finding).

On the *structural level*, reported barriers included the financial cost of care (2 findings), healthcare policies (3 findings), income and food security (4 findings), transport and distance to the clinic (4 findings) and one finding for living conditions and context. Facilitators included income and food security (1 finding), and transport and distance to clinic (2 findings), which includes having an escort to the clinic.

Only one high-quality review [65] was found that addressed linkage to care for HIV-positive adults. One theme was included from the high-quality review, namely, medication as a reminder of HIV status, within the individual level of the framework.

#### Barriers and facilitators to adherence to ART

Findings on barriers and facilitators to adherence to ART, and reviews reporting on these are summarised in Additional file 11. For children, one high-quality review [65], one medium-quality review [41] and two low-quality reviews [68, 73] contributed to the aggregation of findings. For adults, two high-quality reviews [62, 65], four medium-quality reviews [43, 55, 61, 67] and twenty low-quality reviews [44, 47, 48, 50–56, 58–68, 70–73] contributed to the findings for adherence to ART.

On the *individual level*, emerging themes related to barriers to adherence were linked to medication (19 findings), sociodemographic factors (18 findings) and fears (12 findings). Reviews reported medication characteristics, negative side effects, pill burden and regimen, travelling away from home and lack of privacy as barriers, and the use of reminders, simpler medication regimens as facilitators, within the medication theme. Several reviews synthesised findings on the self-reported sociodemographic characteristics such as levels of education, age and gender. In some cases, a woman’s positive HIV status was considered a result of her husband’s infidelity and reduced the risk of stigmatisation when disclosing her status. In other cases when women were seen taking their medication they were stigmatised as hypersexual and were discriminated against. Other reported barriers were grouped under the themes psychological distress and emotions (15 findings) and fears (12 findings). Fears were related to the medication toxicities, side effects, unintentional disclosure, that the treatment would harm a pregnant woman’s unborn child and the fears that ART leads to impotency, infertility, and the impossibility of sexual activity.

The theme of coping strategies (12 findings) and desires (7 findings) were identified as facilitators to mitigate fears, anticipated stigma and negative side effects of ART. Coping strategies included being aware of personal strengths and weakness, learning to manage the HIV diagnosis and interpreting physical signs of the body, drinking liquids, resting and adopting a resilient and positive attitude. People desired to be healthy to care for their families and to maintain their appearance to keep their status a secret. Knowledge and understanding was identified both as a barrier (6 findings), such as receiving conflicting messages from community members, providers, peers and the media; and as a facilitator (7 findings) such as, understanding the need for compliance.

On the *interpersonal level*, peoples’ relationships within the household emerged as a barrier (15 findings) and facilitator (7 findings) to adherence. Family involvement and emotional, material, and social support were important factors to PLHIV. Other barriers such as punishment for lack of adherence for children, negative family reactions to disclosure, enacted stigma by family members and lack of autonomy in relationships made it difficult for people to adhere.

*Community level* barriers were related to community beliefs and practices (6 findings) such as strong negative community beliefs about HIV and bypassing of clinics and hospitals for traditional healers. Peers and support groups (6 findings) played a mitigating role and helped participants adjust to their new daily routine. Financial and emotional support also facilitated adherence.

The two emergent themes identified at the *institutional level* of the framework were service delivery (27 findings), which was reported as a barrier; and models of care (15 findings), which was reported as a facilitator. People who may have had the intention of adhering to ART were discouraged by the difficulties of making a scheduled appointment and the long waiting times at the clinic when they did seek care. Negative experiences at the clinic when collecting the refills of medication included the lack of privacy, overcrowding and stigma experienced within the clinic by other patients, community members and staff. People reported spending up to a day waiting to see a health care worker and were presented with additional barriers such as drug stock-outs or limits on the amount of medication that could be dispensed at a time. Models of care, such as integrated mental health care, integrated antenatal care (ANC) and HIV care, and specialised services for adolescents, with highly skilled and trained healthcare workers,

The *structural-level* themes identified for barriers to ART adherence included the financial cost of ART, healthcare policies, and income and food security, each of which had three findings. PLHIV reported that food insecurity and no access to liquids prevented them from taking their medication. PLHIV felt discouraged by their lack of understanding of healthcare policies and some reported the barrier of access laws at health care facilities that sent patients to their birthplace to seek care. Policies directed at specific populations with criminalising threats for transgender persons, commercial sex workers, drug users and deportation threats for immigrants were reported as barriers. Even with the advent of free ART, the indirect cost of ART is still high in low-income settings with participants expressing the challenge of travelling to clinics in rural areas, the affordability of safe, reliable transport and the indirect cost of childcare when visiting the clinic in order to collect medications. Facilitators at the structural level included financial relief for care (5 findings) and income and food security (2 findings), which included the provision of grants for food supplementation and travel reimbursement.

One high-quality review [65] found that children reported their daily routines and lifestyle, desires to have their lives pre-ART normalised, fears of stigma, fears of the related effects of ART as well as actual negative effects experienced, non-acceptance of HIV status, conflicting messages regarding ART, forgetting or misplacing medication, medication characteristics, pill burden, feeling better, unsupportive family relationships and social isolation as barriers to their medication adherence. No facilitators of adherence to ART for children were reported in the high-quality review. Review findings from two high-quality reviews found that adults reported their beliefs about ART, coping strategies, daily routines, desires, fears, HIV acceptance and non-acceptance, knowledge and understanding of ART, medication factors, physical health, psychological distress, age and competing life interests on the individual level. Relationships in the household on the interpersonal level of the framework; peer and social support groups on the community level; perceptions and engagements of health care workers, integrated models of care, male only services, health care workers’ recommendations, service delivery, financial costs and health care policies on the institutional level; and food insecurity, housing and income as structural factors were self-reported by people as barriers and facilitators of adherence to ART.

#### Barriers and facilitators to retention in care

Ten reviews contributed to the findings for retention in care, and the barriers and facilitators as reported in the reviews, by country income level and quality rating, are presented in Additional file 12. Five low-quality reviews [50, 52, 58, 66, 73] reported on the barriers and facilitators of retention in care for children. Findings for adults were found in seven low-quality reviews [42, 50, 52, 56–58, 66] and two medium-quality reviews [46, 55]. No medium- or high-quality reviews were found for children and no high-quality studies were found for the PLHIV self-reported barriers and facilitators of retention in care.

The prominent themes on the *individual level* were the barriers of sociodemographic factors (5 findings), such as issues around gender, and concerns about not have registered identification documents to access care due to either immigrant status or being transgender; the themes of fears (4 findings); and psychological distress and emotional reactions (7 findings). PLHIV reported experiencing mental fatigue from being retained in care and experiencing psychological suffering as an adult with HIV, which included feeling angry, feeling like they have lost control of their lives and feelings of depression and hopelessness.

On the *interpersonal level*, emergent themes included the barriers (5 findings) and facilitators (3 findings) of disclosure. Disclosure was reported as a barrier either in cases when participants chose not to disclose, and this resulted in sporadic care within health care systems, or due to post-disclosure stigma. Family members who were supportive and relationships in the household (6 findings) were reported as facilitators of retention in care. Partners who were emotionally supportive and encouraged healthy living were considered facilitators of retention in care. In other instances, partners who were not involved in care were considered as barriers, with reviews reporting that women did not have decision-making power in some contexts, and this denied them the opportunity to seek care.

The *community level* had two emergent themes with one finding each. These included the theme of community beliefs and practices and the theme of peers and support groups, which was also reported as facilitators with six key findings. Facilitator findings included having a treatment companion, identifying a confidante, attending support groups and enlisting the help of supervisors and teachers to facilitate retention in care.

For retention in care, many themes that related to the *institutional level* were reported. Themes included service delivery (20 findings) and models of care (17 findings), followed by stigma in health care and engagement (7 findings) and engagement with health care workers (6 findings). Service delivery barriers included long waiting times and subsequent short consultations with health care workers, drug and test stock outs, lack of privacy, laboratory challenges, negative experiences of testing for HIV, the physical clinic environment and the failure of the health care facility to keep up with rapidly changing treatment protocols. PLHIV reported same-day appointments between services offerings at the clinic, their experiences of HIV testing and the provision of disability accommodations to be facilitators of service delivery (5 findings). The facilitators of models of care (9 findings) included integrated care to reduce patient burden, the treatment of depression and anxiety related to diagnosis, offering male-friendly services without needing to access care through partners’ ANC services and home visiting or mobile care units.

*Structural-level* barriers were emergent for health care policies (4 findings), financial costs of care (2 findings), transport and distance to clinic (2 findings), and one finding each for income and food security, and living condition and context. PLHIV reported the cost of attending care even while ART was universally free and accessible as a barrier to engaging in care. Indirect costs included the loss of wages when attending the clinic, transportation costs, childcare costs and the possible loss of grants due to their HIV-positive diagnosis. Only one facilitator of having a higher income was reported at the structural level.

No high-quality reviews were found for self-reported barriers and facilitators of retention in care for children or adults.

### Synthesis of findings

#### Identifying themes and subthemes

**Fig. 6:**
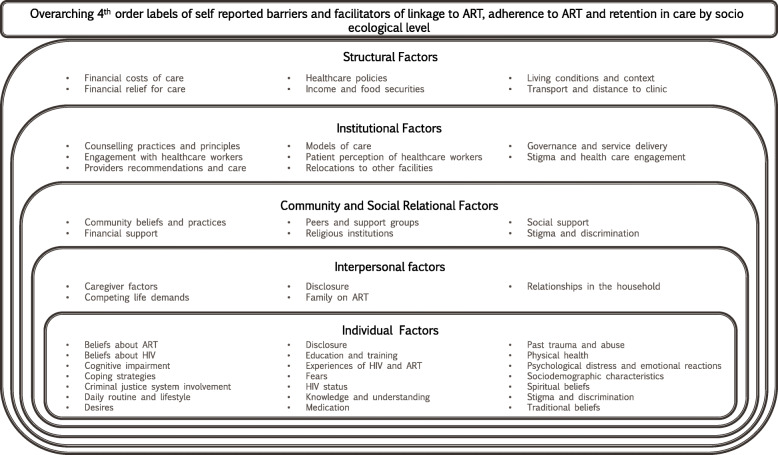
Summary of fourth-order themes by levels of the HIV behaviour change model

We reclassified the third-order concepts into 45 fourth-order themes within the five levels of the Kaufman [23] HIV behaviour change model and summarised the themes of included reviews linked to the outcomes (Additional file 13). For the individual level, we found 19 themes; for the interpersonal level, five themes; for the community level, six themes; for the institutional level, eight themes; and for the structural level, we found six themes (Fig. 6).

#### What are the knowledge gaps in the available review-level evidence about linkage to care?

Review-level evidence (Additional file 9) on the barriers and facilitators to linkage to ART for children is sparse, with a single review reporting on children and adolescents in low- and middle-income countries. A low-quality review identified the main barriers for children in both low- and middle-income countries were categorised into the interpersonal and institutional level of the HIV behaviour change model. Review-level evidence is lacking for the psychological distress and emotional reactions experienced by children and adults when learning about their positive status and possible mode of transmission, whether through unprotected sex or vertical transmission. Furthermore, the mental health of PLHIV as they engage in the continuum of care is underrepresented in the literature. Review-level evidence of the facilitators of linkage are under-reported, as are children’s perceptions of and engagement with health care workers. No high-quality reviews reporting on children’s self-reported barriers and facilitators to linkage were found. For adults, much of the review-level evidence has synthesised the barriers of linkage to ART rather than the facilitators. With only one high-quality review, there is a need for more high-quality reviews on the facilitating effect of community beliefs and practices, internal beliefs and the role of peers and support groups for linkage to treatment for ART. Additionally, there is lack of evidence on the coping strategies employed by children and adults to facilitate linkage. There is a need for high-quality evidence on environmental factors and the social determinants of health on people’s linkage to ART.

#### What are the knowledge gaps in the available review-level evidence about adherence to treatment?

There is minimal review-level evidence for children when compared to the existing evidence of adherence to ART in adult populations (Additional file 10). We found only one high-quality review for children and two high-quality reviews for adults. However, when comparing the methodological quality of reviews, there is a large body of low-quality reviews, with most reviews being conducted on populations from low-income countries. Individual beliefs, desires, coping strategies and fears are addressed in the literature for adults but not adequately for children. No review-level evidence was available on the relationships within the household in low- and middle-income countries for children. There is a large quantity of evidence on the barriers of the characteristics of the medication, the side effects, the psychological distress and emotional reactions and the effects of service delivery but a gap exists in the evidence of the facilitators that can mitigate these barriers in adults. Evidence in adults is lacking on the role of environmental factors, personal beliefs, cultural practices and traditional community beliefs on adherence to ART. Further exploration on the self-reported experiences of psychological distress and engagement with peers in the context of stigma is needed.

#### What are the knowledge gaps in the available review-level evidence about retention in care?

The existing evidence for retention in care is sparse (Additional file 11), and no high-quality reviews were found. The focus of synthesis from the included studies has primarily been conducted in adult populations in low- and middle-income countries. Five low-quality reviews and one medium-quality review addressed the outcome of retention in care among children and no high-quality reviews were found. In children, caregivers are seen as the primary caregivers and gatekeepers for management of a positive HIV diagnosis, but this is not displayed in the existing evidence, as there is a lack of research that is available. Additionally, the role of caregivers in adult populations was not available in the included reviews. In low- and middle-income countries, complexities regarding the co-existence of traditional medicine and scientific-based medicine are briefly mentioned, but not explored, in review-level evidence. Adult population self-reports of family and social relationships are synthesised in low- and middle-income countries as well as high-income countries; however, there is no review-level evidence for children. There is a need for more review-level evidence on the impact of the environment and structural community on retention in care. No high-quality reviews were found for PLHIV reporting barriers and facilitators to retention in care.

### Lines of action and recommendations

Several of the individual, interpersonal, health system and structural-level factors identified in this overview are well known and have been identified in previous literature [35, 75]. We identified key areas and population groups which were under-represented in the review literature and where syntheses of primary research may be needed to understand these factors better.

First, individual-level factors such as peoples’ fears, psychological distress, and beliefs about ART, and conflicting beliefs between cultural practices and medicine, were frequently reported; there was however little to no emphasis in qualitative reviews on the facilitators to mitigate these barriers in children or adult populations.

Second, interpersonal factors such as the dynamics of relationships within the household, and the fears, anxieties, experiences of disclosure and support from peers and support groups were identified both as facilitators and barriers to care; however, details about the complex relationship of these factors needs further exploration.

Third, models of care and service delivery practices adopted by health care centres were prominent themes: with long waiting times, the risk of unintentional disclosure and the negative treatment by health care workers reported as barriers. Skilled health care workers, targeted services for men and youth, and integration of care as facilitating care were identified. Further exploration of not only the healthcare workers, but also the clinic facility, both as a physical space and beliefs about the clinic, is needed.

Fourth, structural-level themes included transportation to the clinic, poverty and low-income levels linked to the lack of food. Emerging issues related to the sensorial experience of adherence, the physical environment and community structure has yet to be explored in qualitative systematic reviews related to HIV.

Fifth, although there is progress to achieving the 90-90-90 targets, this study identified many challenges experienced by PLHIV and the themes of self-reported barriers provide a rich overview of the interwoven complexities of the lives of PLHIV. Recent literature linking barriers and facilitators of linkage, adherence and retention in care to priority solutions propose differentiated service delivery, client-centred care, safe spaces for HIV treatment and care, adolescent friendly services, family centred services and advocacy for human rights, among others, to best serve the needs of PLHIV and reduce the burden on the health care systems. Investigation and scale up of interventions for PLHIV to promote their adherence must be intensified and barriers and facilitators even beyond the realm of interventions proposed including reminder devices, pill box organisers, reduced toxicity, mental health screening and fixed dosed combinations must be explored.

Sixth, this overview identifies the need for qualitative exploration of the agency of the material environment encountered by PLHIV, not included in existing reviews or in intervention research and will make valuable contributions to the body of knowledge. The exploration of agency goes beyond the identification and description of themes to exploring the understanding of the meaning ascribed to materials such as agency of the human body of PLHIV, the agency of matter such as money, transport vehicle, clinic environments, food, community structural factors and living spaces, context and agency of medications.

Lastly, this study identified the need for more high-quality review-level evidence for key populations, specifically, children and youth with perinatal HIV infections, as they were underrepresented in the data. Most included reviews were assessed as having low quality. Future qualitative systematic reviews in the field of HIV linkage, adherence and retention in care should follow a rigorous approach to minimise the risk of bias.

## Discussion

This study described the innovation and first application of the mega-aggregative framework synthesis approach to explore self-reported barriers and facilitators of care in PLHIV as reported in existing qualitative systematic reviews. The purpose of mega-aggregation framework synthesis is to provide an overview of review-level findings to produce lines of action which leads to recommendations. As the number of qualitative reviews increases exponentially [18, 19] and the risk of review duplication in HIV research is high, we considered it valuable to appraise and synthesise the existing evidence. This novel application was conducted on real review-level data which identified 33 systematic reviews from low-, middle- and high-income countries, reporting on linkage to ART, adherence to ART and retention in HIV care, for all populations, across all settings, from 1990 up to July 2018. The protocol was predefined and the overview conducted and reported in a transparent way, with all evidence, data extraction, critical appraisal, justifications for decisions, clear decision guides and data presented in additional files alongside the manuscript. The search was comprehensive, and data extraction, screening and critical appraisal was conducted independently by two authors.

Mega-aggregation framework synthesis for qualitative reviews has been shown to be a reliable and efficient method of synthesis when aiming to identify the quality and scope of available evidence. Researchers are encouraged to use and build on the method as overviews become more prominent in qualitative systematic review literature. Our overview found that the main self-reported barriers and facilitators to linkage, adherence and retention such as psychosocial characteristics of PLHIV such as desires, fears, experiences of HIV and ART, coping strategies and mental health intersected with other factors on the interpersonal, community, institutional and structural level. PLHIV identified stigma and lack of social support, alongside health care services that were not sensitive to their individual needs as barriers to adherence and retention in care. In low-income countries, structural, community and institutional factors were reported frequently, and in high-income countries, individual-level barriers such as fear of medication side effects were more apparent. Although the overall quality of the reviews was low, the findings of this overview provide sufficient evidence to assist with the identification of knowledge gaps in the literature and clear lines of action for research, policy and practice. To understand the experiences of PLHIV when considering committing to lifelong treatment, the potential post-disclosure life changes and the side effects, juxtaposed with their beliefs about medication and diseases, it is important to consider all the factors on the individual, interpersonal, community, institutional and structural factors that influence their decision-making and actions. Practitioners working in health care cannot treat patients only for their diagnosis of HIV but must provide holistic care and be aware of the barriers and facilitators that these patients may be experiencing, from the patients’ perspectives. Themes that were found varied across stages of the HIV cascade, and the reasons why PLHIV chose to adhere could be different to the reasons why they chose to engage in care or attend the clinic. The experiences of one patient may not be the experience of another and should be dealt with a case-by case manner within the contextual circumstance of the patient. The healthcare policies have been reported as not easily understood and methods of dissemination and education need to be considered for information uptake in communities. Exploration into the agency of the material spaces occupied and material items in the spaces of PLHIV will provide further understanding on the complex interplay of barriers and facilitators, especially in low- and middle-income countries. Community engagement workers can use their platforms to create open dialogues about HIV, the negative beliefs and stigmas, the dissemination of information on services that are provided in the communities and to promote the positive effects of staying adherent to ART for people infected with HIV. The findings of this overview provide researchers and practitioners with a broad overview of the existing evidence and are useful in the development of new research questions to respond to evidence gaps identified. It is known that the factors associated with linkage, adherence and retention in care do not occur in isolation but are in fact complex and tightly interwoven [43].

We first considered developing this method of synthesis after searching for an appropriate way to synthesise a large amount of qualitative systemic reviews. We wanted to know what was available, what was the quality of the evidence, and what new research questions should we invest our time and resources into. In the context of the pragmatic stance and the anticipated large number of existing systematic reviews, a predetermined theoretical framework [23] with broad categories was selected to guide the aggregation and synthesis within this overview, which built on the steps in methods development for conducting overviews [24], QES [25, 26], systematic review synthesis [19, 27, 28], meta-aggregation [9, 20, 21] and framework synthesis [29, 30]. While conducting this overview, a worked example of mega-ethnography [8] was published. However, there was and there currently is no specific guidance on conducting a pragmatic synthesis in an overview of qualitative review-level evidence. We initially mapped out broad steps aligned to conventional systematic review methods with deviations in the analysis. As we worked through and reflected on the synthesis, we found it critical to identify the theoretical framework that had broad categories within which we could extract data at the outset of the protocol, and to produce lines of action and recommendations at the end of synthesis. We then refined the method into 8 steps which we illustrated in this manuscript. The steps include the following: (1) identify a clearly defined review question and objectives, (2) identify a theoretical framework or model, (3) decide on criteria for considering reviews for inclusion, (4) conduct searching and screening, (5) conduct quality appraisal of the included studies (although some may prefer not too), (6) data extraction and categorisation, (7) present and synthesise the findings, and (8) transparent reporting.

Before embarking on new systematic reviews, it is important for authors and review teams to take stock of existing evidence and minimise the risk of creating research waste. This overview highlights the need for more qualitative review-level evidence, with high methodological quality, that explores the complex relationships between structural factors and adherence, between sociodemographic factors such as community violence and retention, and the experiences of growing up with HIV in low- and middle-income countries, specifically in the populations of youth, women and key populations.

### Strengths and limitations of the mega-aggregative framework approach

Through our worked application, we identified benefits of the mega-aggregative framework approach. First, the pragmatic philosophical underpinning promoted the aggregation of findings and a clear identification of where research is lacking, the quality of the research and the location of research across socio-economic statuses of countries. Some overviews only provide a synthesis without clear directives for policy and practice, and authors may see this as optional—leaving the reader to deduce or draw their own conclusions on the way forward. Using the mega-aggregative framework approach, the lines of action provide researchers and funders with clear guidance on where to direct funding and resources or to develop new research questions. It also provides practitioners with a comprehensive selection of the available evidence and the appraised quality of the evidence. A second benefit is that a mega-aggregation framework synthesis can be conducted in a relatively short amount of time. Other methods of synthesis can be iterative as review authors make sense of their own subjective interpretations. Using a framework with broad categories allows for linear working and promotes the comparing of findings between review authors. Using this method, the number of findings is counted and reported, with research gaps and recommendations for policy and practice being apparent immediately. Concrete time is then spent of the lines of action. This approach is feasible and can be especially attractive to novice researchers. A third benefit is the attention to the methodological quality of included systematic reviews; however, the benefits of the method proposed are not without its challenges or limitations. While there is ongoing debate [64, 65] about when, within which paradigmatic stance, and whether to conduct critical appraisal of included studies within a qualitative evidence synthesis, we included methodological appraisal as a step in the mega-aggregative approach. We found very few high-quality reviews examining self-reported barriers and facilitators to linkage, adherence to ART and retention in care for children and adults. In the results, we presented an aggregation of findings and the provided a separate paragraph detailing the findings of the high-quality included reviews. This does not imply that the primary studies included in the reviews are of low quality, as we only assessed the methodological quality of the systematic reviews. We used guidance from recent methodological papers [25, 26, 76–78] in the field of critical appraisal to revise the JBI-SR Checklist [78] for our overview and to determine our decision rules for overall quality of the reviews. The appraisal of the reviews challenged us as authors to think reflexively about the domains that influenced the quality of the reviews and the items on the appraisal tool that explored these domains. As with overviews of qualitative systematic reviews, the area for method development in critical appraisals of QES is understudied and tools for critical appraisal of QES are still under development. Using the mega-aggregative framework approach can limit the author team in their creativity or conceptual modelling of new theories. The mega-aggregative framework approach uses an existing framework and pragmatic approach that can make the analysis process mechanical and sometimes force the data into predefined categories. When we refined the steps of the method, we included selection of the framework as a critical step as much of the synthesis is defined by it. Using a framework with broad categories is preferable. Additionally, we experienced challenges related to the definitions of the outcomes of interest, namely, linkage, adherence and retention in care, in the included qualitative reviews, even though these were pre-specified in the protocol. In quantitative research, measurements are used to determine adherence, whereas qualitative data may be broader. We discussed these in the author team and decided to use overarching definitions and broad categorisation. It is recommended that future qualitative research interrogate the conceptual definitions of linkage, adherence and retention in care for PLHIV. We were challenged by our own assumptions that the pragmatic aggregative nature of the proposed synthesis would be easily applied to sub-group analysis. We intended to explore the data by subgroups for children 0–13 years, young people and adolescents, perinatally infected adolescents and adult subgroup populations, but due to the different reporting methods and study designs, we were unable to provide a detailed synthesis of themes for these subgroups. We were also unable to group specific themes to specific population groups or contexts. However, we grouped children and youth as up to 24 years and adults as our second population group. Furthermore, we found a high proportion of reviews to be conducted in low- and middle-income countries. We conducted the aggregation for all studies and included evidence annexes indicating the socio-economic country grouping as low, middle, or high for each reference. Lastly, the synthesis of the findings was limited to the included review authors’ interpretation of the data and we included the primary study data as per the review authors’ description. It does become challenging for research synthesis as the production of QES is increasing and the risk of sampling and re-interpretation of the same primary studies for different outcomes and objectives through different paradigmatic lenses. We extracted themes from the reviews as per the authors’ interpretation. However, using guidance from meta-aggregation [9, 22], we only included themes that were considered unequivocal, meaning the themes were supported with evidence in the included review. We provided transparent decision-making notes and evidence annexes to this manuscript to contribute to the trustworthiness of the findings of this overview.

## Conclusion

This paper describes the development and illustrates the first application of mega-aggregation framework synthesis for QES. The use of overviews to provide a synthesis of the existing review-level evidence is expected to increase as the pool of systematic reviews continues to grow. As with all research designs dependent on the research question and available resources, the aims and scope of QES—at review and overview level—are varied. Mega-aggregative framework synthesis is feasible and easy to implement and follows a linear process. Author teams interested in timeous and pragmatic ways to go beyond scoping reviews or evidence gap maps, into creating synthesised statements with practical lines of action and recommendations, are encouraged to experiment and utilise the illustrated innovative method of mega-aggregative framework synthesis for use in overviews of qualitative systematic reviews. Further evaluation and development of this method is needed to test its utility with different types of data and in comparison, to different approaches in qualitative synthesis.

## Supplementary Information

**Additional file 1.** Search Strategies for electronic databases

**Additional file 2.** Revised decision rules for JBI-SR-Checklist

**Additional file 3.** Data extraction form

**Additional file 4.** Description and application of mega-aggregation framework synthesis

**Additional file 5:.** PRISMA Extension for Scoping Reviews (PRISMA-ScR): Checklist

**Additional file 6.** Differences between protocol and manuscript

**Additional file 7. **Tables of excluded studies, ongoing studies, and protocols (*N =* 45)

**Additional file 8.** Individual summaries and critical appraisal reasons

**Additional file 9. **Overlap of included primary studies (*n =* 826)

**Additional file 10:.** Summary of review level evidence: Linkage to ART

**Additional file 11.** Summary of review level evidence: Adherence to ART

**Additional file 12.** Summary of review level evidence: Retention in care

**Additional file 13.** Summary of themes and included reviews linked to outcomes

## Data Availability

All data generated or analysed during this study are included in this published article and its supplementary information files.

## Abbreviations

AIDS: Acquired immunodeficiency syndrome
ART: Antiretroviral therapy
HAART: Highly active antiretroviral therapies
HIV: Human immunodeficiency virus
PLHIV: People living with HIV
IDU: Intravenous drug user
JBI: Joanna Briggs Institute
JBI-SR Checklist: Joanna Briggs Systematic Review Checklist
MSM: Men who have sex with men
PREP: Pre-exposure prophylaxis
PRISMA-ScR: PRISMA Extension for Scoping Reviews Checklist
