# Supplementary material for: A mega-aggregation framework synthesis of the barriers and facilitators to linkage, adherence to ART and retention in care among people living with HIV
Source: Syst Rev. 2021 Feb 11;10:54. doi: 10.1186/s13643-021-01582-z (PMC7875685; doi:10.1186/s13643-021-01582-z)
Supplement: Supplementary file 1 — Additional file 1. Search Strategies for electronic databases [file 13643_2021_1582_MOESM1_ESM.docx]

**Additional file 1: Search Strategies for electronic databases**

**Campbell Library:**

<https://www.campbellcollaboration.org/library.html>

HIV or AIDS

**Cinahl EbscoHost**

| S23 | S12 and S17 and S22 |
| --- | --- |
| S22 | S18 or S19 or S20 or S21 |
| S21 | TI (“integrative research” OR “integrative review*” OR “integrative overview*” OR “research integration*” OR “research overview*” OR “collaborative review*” OR “collaborative overview*” OR “systematic review*” OR “systematic overview*” OR “methodological overview*” OR “methodologic overview*” OR “methodological review*” OR “methodologic review*” OR “quantitative review*” OR “quantitative overview*” OR “quantitative synthes*” OR “data synthes*” OR “qualitative overview*” OR “qualitative synthes*” OR “data extraction” OR “data abstraction*”) OR AB ( “integrative research” OR “integrative review*” OR “integrative overview*” OR “research integration*” OR “research overview*” OR “collaborative review*” OR “collaborative overview*” OR “systematic review*” OR “systematic overview*” OR “methodological overview*” OR “methodologic overview*” OR “methodological review*” OR “methodologic review*” OR “quantitative review*” OR “quantitative overview*” OR “quantitative synthes*” OR “data synthes*” OR “qualitative overview*” OR “qualitative synthes*” OR “data extraction” OR “data abstraction*” ) |
| S20 | TI ( (meta analysis or meta-analysis or metaanalysis) ) OR AB ( (meta analysis or meta-analysis or metaanalysis) ) |
| S19 | MH systematic review |
| S18 | PT systematic review |
| S17 | S13 OR S14 OR S15 OR S16 |
| S16 | MH medication adherence |
| S15 | TI ( (Adherence OR adher* OR compliance OR complian* OR comply OR complied OR noncomplian* OR non-complian* OR non-adher* OR nonadher*) ) OR AB ( (Adherence OR adher* OR compliance OR complian* OR comply OR complied OR noncomplian* OR non-complian* OR non-adher* OR nonadher*) ) |
| S14 | TI ( (Retention OR retain* OR "lost to follow-up" OR ("loss*" AND "follow up") OR LTFU OR “loss-to-follow-up” OR attrition OR "loss to care" OR "loss to program*" OR default* OR engage* OR disengage* OR “retention in care” OR “lost to retention”) ) OR AB ( (Retention OR retain* OR "lost to follow-up" OR ("loss*" AND "follow up") OR LTFU OR “loss-to-follow-up” OR attrition OR "loss to care" OR "loss to program*" OR default* OR engage* OR disengage* OR “retention in care” OR “lost to retention”) ) |
| S13 | TI ( (“treatment initiation” OR link* OR “link to care” OR “link to treatment” OR “linkage to treatment” OR “link into care” OR “linkage into treatment”) ) OR AB ( (“treatment initiation” OR link* OR “link to care” OR “link to treatment” OR “linkage to treatment” OR “link into care” OR “linkage into treatment”) ) |
| S12 | S1 OR S2 OR S3 OR S4 OR S5 OR S6 OR S7 OR S8 OR S9 OR S10 OR S11 |
| S11 | AB hiv/aids |
| S10 | AB (acquired immun*) AND (deficiency syndrome) |
| S9 | AB “acquired immunodeficiency syndromes” OR “acquired immune deficiency syndrome” OR “acquired immuno-deficiency syndrome” OR “acquired immune-deficiency syndrome” |
| S8 | AB (human immun*) AND (deficiency virus) |
| S7 | AB (HIV OR hiv-1 OR hiv-2* OR hiv1 OR hiv2 OR “hiv infect*” OR “human immunodeficiency virus” OR “human immune deficiency virus” OR “human immuno-deficiency virus” OR “human immune-deficiency virus”) |
| S6 | TI hiv/aids |
| S5 | TI (acquired immun*) AND (deficiency syndrome) |
| S4 | TI “acquired immunodeficiency syndromes” OR “acquired immune deficiency syndrome” OR “acquired immuno-deficiency syndrome” OR “acquired immune-deficiency syndrome” |
| S3 | TI (human immun*) AND (deficiency virus) |
| S2 | TI (HIV OR hiv-1 OR hiv-2* OR hiv1 OR hiv2 OR “hiv infect*” OR “human immunodeficiency virus” OR “human immune deficiency virus” OR “human immuno-deficiency virus” OR “human immune-deficiency virus”) |
| S1 | MW hiv OR MW hiv infection |

**Cochrane Library and Cochrane Library DARE:**

| #1 | MeSH descriptor: [HIV] explode all trees |
| --- | --- |
| #2 | MeSH descriptor: [HIV Infections] explode all trees |
| #3 | "human immunodeficiency virus" |
| #4 | "human immuno-deficiency virus" |
| #5 | "acquired immunodeficiency syndrome" |
| #6 | acquired immuno-deficiency syndromes |
| #7 | "acquired immunodeficiency syndromes" |
| #8 | acquired immuno-deficiency syndrome |
| #9 | AIDS |
| #10 | #1 or #2 or #3 or #4 or #5 or #6 or #7 or #8 or #9 |
| #11 | "treatment initiation" or link* or "link to care" or "link to treatment" or "linkage to treatment" or "link into care" or "linkage into treatment" |
| #12 | Retention or retain* or "lost to follow-up" or ("loss*" and "follow up") or LTFU or "loss-to-follow-up" or attrition or "loss to care" or "loss to program*" or "loss to programme*" or default* or engage* or disengage* or "retention in care" or "lost to retention" |
| #13 | Adherence or adher* or compliance or complian* or comply or complied or noncomplian* or non-complian* or non-adher* or nonadher* |
| #14 | MeSH descriptor: [Medication Adherence] explode all trees |
| #15 | #15 #11 or #12 or #13 or #14 |
| #16 | #10 and #15 |

**Medline (PubMed)**

| #1 | Search "HIV"[Majr] |
| --- | --- |
| #2 | Search "HIV Infections"[Majr] |
| #3 | Search HIV[Title/Abstract] |
| #4 | Search hiv-1[Title/Abstract] |
| #5 | Search hiv-2*[Title/Abstract] |
| #6 | Search hiv1[Title/Abstract] |
| #7 | Search hiv2[Title/Abstract] |
| #8 | Search hiv infect*[Title/Abstract] |
| #9 | Search “human immunodeficiency virus”[Title/Abstract] |
| #10 | Search “human immune deficiency virus"[Title/Abstract] |
| #11 | Search “human immuno-deficiency virus”[Title/Abstract] |
| #12 | Search “human immune-deficiency virus”[Title/Abstract] |
| #13 | Search (((human immun*) AND (deficiency virus))) |
| #14 | Search “acquired immunodeficiency syndromes”[Title/Abstract] |
| #15 | Search “acquired immune deficiency syndrome”[Title/Abstract] |
| #16 | Search “acquired immuno-deficiency syndrome”[Title/Abstract] |
| #17 | Search “acquired immune-deficiency syndrome”[Title/Abstract] |
| #18 | Search HIV/AIDS[Title/Abstract] |
| #19 | Search (((acquired immun*) AND (deficiency syndrome))) |
| #20 | #1 OR #2 OR #3 OR #4 OR #5 OR #6 OR #7 OR #8 OR #9 OR #10 OR #11 OR #12 OR #13 OR #14 OR #15 OR #16 OR #17 OR #18 OR #19 |
| #21 | Search "Medication Adherence"[Majr] |
| #22 | Search (((((((((adherence[Title/Abstract]) OR adher*[Title/Abstract]) OR compliance[Title/Abstract]) OR complian*[Title/Abstract]) OR comply[Title/Abstract]) OR complied[Title/Abstract]) OR noncomplian*[Title/Abstract]) OR non-complian*[Title/Abstract]) OR non-adher*[Title/Abstract]) OR nonadher*[Title/Abstract] |
| #23 | Search ((((((((((((((retention[Title/Abstract]) OR retain*[Title/Abstract]) OR "lost to follow-up"[Title/Abstract]) OR (("loss*"[Title/Abstract] AND "follow up")[Title/Abstract])) OR LTFU[Title/Abstract]) OR "loss-to-follow-up"[Title/Abstract]) OR attrition[Title/Abstract]) OR "loss to care"[Title/Abstract]) OR "loss to program"[Title/Abstract]) OR "loss to programme"[Title/Abstract]) OR default*[Title/Abstract]) OR engage*[Title/Abstract]) OR disengage*[Title/Abstract]) OR "retention in care"[Title/Abstract]) OR "lost to retention"[Title/Abstract] |
| #24 | Search (("treatment initiation"[Title/Abstract]) OR link[Title/Abstract]) OR linkage[Title/Abstract] |
| #25 | Search (#21 OR #22 OR #23 OR #24) |
| #26 | Search "Meta-Analysis" [Publication Type] |
| #27 | Search "Meta-Analysis as Topic"[Mesh] |
| #28 | Search (Search “integrative research” OR “integrative review*” OR “integrative overview*” OR “research integration*” OR “research overview*” OR “collaborative review*” OR “collaborative overview*” OR “systematic review*” OR “systematic overview*” OR “methodological overview*” OR “methodologic overview*” OR “methodological review*” OR “methodologic review*” OR “quantitative review*” OR “quantitative overview*” OR “quantitative synthes*” OR “data synthes*” OR “qualitative overview*” OR “qualitative synthes*” OR “data extraction” OR “data abstraction*”) |
| #29 | #26 OR #27 OR #28 |
| #30 | #20 AND #25 AND #29 |

**PROSPERO:** (limit to ongoing reviews)

HIV adherence OR HIV linkage OR (HIV AND retention)

**SCOPUS**

TITLE-ABS-KEY ( ( meta-analysis OR "systematic review" ) AND ( hiv OR aids ) AND ( "treatment initiation" OR link* OR adherence OR adher* OR compliance OR complian* OR noncomplian* OR non-complian* OR non-adher* OR nonadher* OR "lost to follow-up" OR attrition ) ) AND PUBYEAR > 2015
