# Supplementary material for: A mega-aggregation framework synthesis of the barriers and facilitators to linkage, adherence to ART and retention in care among people living with HIV
Source: Syst Rev. 2021 Feb 11;10:54. doi: 10.1186/s13643-021-01582-z (PMC7875685; doi:10.1186/s13643-021-01582-z)
Supplement: Supplementary file 2 — Additional file 2. Revised decision rules for JBI-SR-Checklist [file 13643_2021_1582_MOESM2_ESM.docx]

**Additional file 2: Revised decision rules for JBI-SR-Checklist**

1. *Is the review question clearly and explicitly stated?*

The review questions are useful in guiding the search strategy, study design and how the review was conducted. Reviews with evidence of the main objective or question clearly stated that contains the PICO elements of Population, Issue, Context and Outcomes will be graded as a “yes”. As this overview is not inclusive of intervention studies the PICO elements have been redefined to incorporate qualitative systematic reviews. If the review contains many questions or aims that may not be clearly defined then the review will be graded as “unclear”. Reviews without review questions will be graded as a “no”.

1. *Were the inclusion criteria appropriate for the review question?*

Included studies should be appropriate to the review question. The inclusion criteria but be clearly stated before an assessment can be made or this appraisal question will be marked as “unclear”. If study designs are aligned to review questions, the review will be graded as a “yes”, otherwise if no alignment is established the review will be graded as a “no”.

1. *Was the search strategy appropriate?*

Systematic reviews use specific search strategies relevant to databases to locate evidence in the literature. The systematic reviews should provide evidence of a comprehensive search strategy and may be available in the methods sections of the review or available in the supplementary material of the publication. The search strategy will be deemed appropriate if it contains the PICO components of the research question or the review authors provided a description of the approach with key words and how the terms were derived. A systematic review should present a clear search strategy that addresses each of the identifiable PICO components of the review question.

Some reviews, due to word limit may only provide a description of the approach to searching, the relevant key words and terms, how the terms that were ultimately used were derived, and Subject Headings or Index terms. Search limiters such as search date or language may impact the results of the search and these will be considered for each review. If reviewers state a pre-specified search strategy was used but do not provide detail of the strategy, the reviews will be considered “unclear”. If no information on the search strategy or languages included is provided then the appraisal result for this question will be “no”.

1. *Were the sources and resources used to search for studies adequate?*

Databases searched should be relevant to the review with at least two or more databases being searched, such reviews will result in a “yes” appraisal. Resources may include grey literature, conference abstracts or thesis repositories. Lack of evidence of these will result in a downgrade for this overview. If search sources are not specified the review question will be appraised as a “no” and if too little information is available to make a decision then the review question will be appraised as “unclear”.

1. *Was the screening and study selection appropriate?*

Study title, abstract and full text screening should be conducted by two reviewers independently and in duplicate to be graded as a “yes”. If studies were selected and screened by one reviewer and a proportion or all are checked by a second reviewer the study will be appraised as a “yes”. If studies did title and abstract screening in duplicate and full text screening was only conducted by one reviewer, or vice versa, the review will be graded as a “no”. Studies reporting screening and selection to be conducted by one author will be graded as a “no”. When no information or too little information is available the review will be graded as “unclear”.

1. *Were the criteria for appraising studies appropriate?*

To be appraised as “yes” reviews need to include a clear statement that a critical appraisal tool was used and provide details of the items used to assess the included studies. Additionally, the critical appraisal tool used should be relevant to the objective and scope of the review. If either these criteria are not met, or there is no mention of the process of critical appraisal, the review will be downgraded to a “no”. If the review authors state that an appraisal was used but do not provide details of the appraisal, the review will be graded as “unclear”.

1. *Was critical appraisal conducted by two or more reviewers independently?*

In some instances all appraisals may have be conducted by one author and checked by another, however discrepancies must be resolved by consensus or with a third person to be graded as a “yes”. Not meeting these requirements will be result in a “no”. Where no critical appraisal was used in the review, the review will be graded as a “no”. Where too little information is available to make a decision, the review will be graded as “unclear”.

1. *Were there methods to minimize errors in data extraction?*

Reviews will be graded as a “yes” if efforts to minimize errors in data extraction include extracting data in duplicate and independently, using specific tools to guide the extraction or piloting of extraction tools. If it is clear that none of these strategies are used, the review will be graded as a “no”. If no information is provided, the review will be graded as “unclear”.

1. *Were the methods used to combine studies appropriate?*

If the method of synthesis the review used is aligned to the review question, the type of review and the type of evidence included in the review will be graded as a “yes”. The appraisal will also include whether the descriptive and explanatory information support the final synthesized findings from the original research. If the data synthesis method is not aligned to the review question and method, the review will be graded as a “no”. Where too little information is available to make a decision, the review will be graded as “unclear”.

1. *Were recommendations for policy and/or practice supported by the reported data?*

This question assesses the review validity rather than quality and appraises whether the recommendations made are aligned clearly to the results of the review, evidence of which will result in a “yes” grade for the review question. If recommendations are provided but are not aligned to the research data, the review question will be graded as a “no”. If no recommendations are provided, the review question will be graded as an “unclear”.

1. *Were the specific directives for new research appropriate?*

The purpose of reviews is to identify gaps in the literature and areas where further research is identified. If review authors present recommendations for future research, relevant to findings and methods of the review, the review will be graded as “yes” for this review question. If the directives for future research are not appropriate to the review, the question will be graded as a “no”. If no directives for future research are provided, the review question will be graded as “unclear”.

**Methodological quality decision rules**

| Rule | Decision on the quality of the conduct of the systematic review |
| --- | --- |
| Two or more ‘No’ | Low quality |
| One ‘No’ and 3 or more ‘Unclear’ | Low quality |
| One ‘No’ and 0-2 ‘Unclear’ | Medium quality |
| Zero ‘No’ and 3 or more ‘Unclear’ | Medium quality |
| Zero ‘No’ and 1-2 ‘Unclear’ | High quality |
| Zero ‘No’ and zero ‘Unclear’ | High quality |
