# Supplementary material for: A mega-aggregation framework synthesis of the barriers and facilitators to linkage, adherence to ART and retention in care among people living with HIV
Source: Syst Rev. 2021 Feb 11;10:54. doi: 10.1186/s13643-021-01582-z (PMC7875685; doi:10.1186/s13643-021-01582-z)
Supplement: Supplementary file 3 — Additional file 3. Data extraction form [file 13643_2021_1582_MOESM3_ESM.docx]

**Additional file 3: Data extraction form**

| **Characteristics of included systematic review** | | | | |
| --- | --- | --- | --- | --- |
| **Citation** | | | | |
|  | | | | |
| **Search Summary Details** | | | | |
| **Literature Searched** |  | | | |
| **Search Dates** |  | | **Date of last search:** | |
| **Search Criteria and Outcomes** | | | | |
|  | **What the review authors searched for** | | **What the review authors found relevant to this overview** | |
| **Studies** |  | | (N) Studies: Qualitative (n=), Quantitative (n=) and Mixed Methods (n=) | |
|  |  |  | **Studies relevant to this overview:** | |
|  |  |  | (N) Studies: Qualitative (n=), Quantitative (n=) and Mixed Methods (n=) | |
| **Participants** |  | |  | |
| **Issue** |  | |  | |
| **Setting** |  | |  | |
| **Outcome: Barriers and Facilitators** |  | | **Overview Framework**s | |
| **Systematic Review Methods** | | | | |
| **Conceptual framework** |  | | | |
| **Data extraction method** |  | | | |
| **Appraisal tool used** |  | | | |
| **Data synthesis method** |  | | | |
| **JBI Quality Appraisal** | | | | |
| **Question** | | **Judgement** | | **Justification** |
| **1. Is the review questions clearly and explicitly stated?** | |  | |  |
| **2. Were the inclusion criteria appropriate for the review question?** | |  | |  |
| **3. Was the search strategy appropriate?** | |  | |  |
| **4. Were the sources and resources used to search for studies adequate?** | |  | |  |
| **5. Was selection of studies done adequately?** | |  | |  |
| **6. Were the criteria for appraising studies appropriate?** | |  | |  |
| **7. Was the critical appraisal conducted by two or more reviewers independently?** | |  | |  |
| **8. Were there methods to minimize errors in the data extraction?** | |  | |  |
| **9. Were the methods used to combine studies appropriate?** | |  | |  |
| **10. Were recommendations for policy and/or practice supported by the reported data?** | |  | |  |
| **11. Were the specific directives for new research appropriate?** | |  | |  |
