# Supplementary material for: A mega-aggregation framework synthesis of the barriers and facilitators to linkage, adherence to ART and retention in care among people living with HIV
Source: Syst Rev. 2021 Feb 11;10:54. doi: 10.1186/s13643-021-01582-z (PMC7875685; doi:10.1186/s13643-021-01582-z)
Supplement: Supplementary file 4 — Additional file 4. Description and application of mega-aggregation framework synthesis [file 13643_2021_1582_MOESM4_ESM.docx]

**Additional file 4: Description and application of mega-aggregation framework synthesis**

| **Phases of mega-aggregative framework synthesis** | **Description of phase** | **Application in this overview** |
| --- | --- | --- |
| **Step 1: Identify a clearly defined review question and objectives** | - Have a clearly defined research question that outlines participants, phenomena, setting, outcomes, and types of reviews. | - What is the available review level evidence of the self-reported barriers and facilitators to linkage, adherence to ART, and retention in care, for people living with HIV in low to middle income countries? |
| **Step 2: Identify a theoretical framework or model** | - Identify a framework and the categories that can be used to understand and interpret review findings. - Broader frameworks are useful for overviews to synthesize existing qualitative systematic review findings. - Provide a rationale as to why the model is appropriate. | - The Kaufman et al.^(1)^ framework for HIV Behaviour Change was identified and the categories of individual, interpersonal, community, health system, and structural factors were used. - The rationale for the model is presented in the manuscript on page 6-7. |
| **Step 3: Decide on criteria for considering reviews for inclusion** | - Have a clear inclusion and exclusion criteria. - Identify the types of reviews that you will include, the types of participants, types of phenomena or issues, types of contexts, and the types of outcomes. | - Systematic reviews were defined as those reviews that had predetermined objectives, predetermined criteria for eligibility, searched at least two data sources, of which one needed to be an electronic database, and performed standardised data extraction. - Systematic reviews were considered eligible if they included only qualitative studies. Reviews containing qualitative and quantitative studies were still considered eligible if outcomes were self-reported and a narrative description was used to summarise review findings. - Further clarification on inclusion and exclusion criteria is available on page 7-10 of the manuscript. |
| **Step 4: Conduct searching and screening** | - A comprehensive search includes all relevant literature. - Screening and selection of studies should be conducted in duplicate and independently by two authors or more. | - A comprehensive search was conducted up to July 2018 with no limits on language, geographic, time period or type of publication - See figure 2 for PRISMA flowchart that provides results of the search and screening. |
| **Step 5: Conduct quality appraisal of the included studies** | - A reliable and appropriate tool must be used to conduct appraisal. - If appraisal is not conducted, reasons should be provided. - Critical appraisal of studies should be conducted in duplicate and independently by two authors or more. - Full description of judgements and the reasons for judgments should be evidenced. | - Critical appraisal was conducted using the JBI Checklist for Systematic Reviews and Meta analysis - See additional file 2 for decision rules that were specified in the protocol. - See additional file 8 for the appraisal of individual studies with reasons. |
| **Step 6: Data extraction and categorisation** | - Familiarization with the data and extraction of study characteristics. - Repeated reading of the systematic reviews with special attention to the characteristics of included studies, conceptual framework, method of synthesis, findings, discussion, recommendations for policy, practice and research, and evidence for recommendations made. - Verbatim extraction of review findings and search for supporting evidence or data for the extracted findings. - Qualitative software packages can be used to extract relevant review findings verbatim or findings can be extracted manually and captured into another processing programme like EXCEL. - The extractions are considered third order concepts. - Extracted findings are checked for supporting data or evidence. - Evidence can include a reference to the primary study/ies, direct quote, visual or text evidence from the primary study, visual representations such as tables and figures with reference to the primary study/ies that the finding was based on. - Only findings with supporting evidence is included in the overview synthesis. - Categorize codes into the predefined framework. This may also include sub-population disaggregation. | - The first and second author read the 33 included reviews repeatedly to familiarize themselves with the available evidence. - See additional file 8 for detailed individual review summaries of data extraction, appraisals and discussions were recorded in a shared database that authors could refer to. |
|  |  | - The outcomes of interest in this overview were the barriers and facilitators to linkage, adherence and retention in care. - The barriers and facilitators reported by the review authors were identified within the reviews, extracted verbatim and recorded into EXCEL as third order concepts. - Each code was checked for supporting data or evidence. - All codes had supporting evidence and were included in the synthesis. - Evidence was found in the review authors tables of included studies, within the text of the review or within other supplementary material provided by the review authors. - See Additional file 10-13 for evidence annexes and a summary of themes. - Each code was categorized into the predefined framework categories: individual, interpersonal, community, health system, and structural factors by outcome and population group into the EXCEL database. - See Additional file 10-13 for evidence annexes and a summary of themes. |
| **Step 7: Present and synthesize the findings** | - Codes may be grouped into themes (considered 4^th^ order concepts) within the framework categories. - Provide a description of the themes within the categories of the framework. - Visual diagrams can enhance the transparency of this process. - Identify evidence gaps and make recommendations for future research - Describe the scope, quality and gaps in evidence within each category. Sub-group description will be beneficial for thick description. - Describe implications for future research. | - The extracted codes were grouped into themes and considered fourth order concepts/themes. - 544 third order concepts were reclassified into 45 fourth order themes and categorized into the 5 categories of the SEM framework. - See Additional File 13 for a summary of themes and codes. - See Figure 6 for framework with themes and descriptions. - Full description provided within the manuscript in the conclusion. |
| **Step 8: Transparent reporting** | - Generate the report containing visual and text descriptions of the process of synthesis. - Using a reporting guideline to assist in the writing of the manuscript. - The protocol and registration information should be available. - Differences between the protocol and the manuscript should be reported in an appendix. | - This manuscript provides details of the synthesis approach with supporting Additional Files and Figures to enhance the transparency and thick description within the report. - See PRISMA Extension for Scoping Reviews checklist was used as a reporting guideline (additional file 5). - The protocol pertaining to this overview was registered on PROSPERO (CRD42017078155) on 17 December 2017. - Differences between the protocol and manuscript are identified in Additional file 6. |
