# Supplementary material for: A mega-aggregation framework synthesis of the barriers and facilitators to linkage, adherence to ART and retention in care among people living with HIV
Source: Syst Rev. 2021 Feb 11;10:54. doi: 10.1186/s13643-021-01582-z (PMC7875685; doi:10.1186/s13643-021-01582-z)
Supplement: Supplementary file 6 — Additional file 6. Differences between protocol and manuscript [file 13643_2021_1582_MOESM6_ESM.docx]

**Additional file 6: Differences between protocol and manuscript**

We amended the inclusion criteria for types of systematic reviews to include reviews that “included qualitative and mixed methods studies containing qualitative data. Reviews that included both qualitative and quantitative studies were included but only qualitative primary studies were used for analysis. Reviews that included only quantitative studies were excluded”.

The protocol specified:

*“Qualitative systematic reviews that include self-report data, quantitative studies (specifically cross sectional or survey studies that contain open ended questions), or mixed methods studies that make reference to the perceived barriers and facilitators to linkage, adherence and retention in care of HIV positive persons will be included in this overview.”*

We made this amendment during the screening phase as including only ‘qualitative systematic reviews’ was too specific for our inclusion and it resulted in us excluding studies containing relevant qualitative data. By amending the type of systematic reviews to ‘systematic reviews containing qualitative data’ we conducted a more comprehensive review.

Due to the complexity and amount of data in this overview, we made a decision after the protocol was submitted for publication, to create decision rules for quality appraisal cut off scores in order to rate reviews as low quality, medium quality and high quality. Reviews were not excluded based on their quality but the ratings were used in the interpretation of the review findings and to explore the gaps in evidence.
