# Supplementary material for: A mega-aggregation framework synthesis of the barriers and facilitators to linkage, adherence to ART and retention in care among people living with HIV
Source: Syst Rev. 2021 Feb 11;10:54. doi: 10.1186/s13643-021-01582-z (PMC7875685; doi:10.1186/s13643-021-01582-z)
Supplement: Supplementary file 7 — Additional file 7. Tables of excluded studies, ongoing studies, and protocols (N = 45) [file 13643_2021_1582_MOESM7_ESM.docx]

**Additional file 7: Tables of excluded studies, ongoing studies and protocols (N=45)**

**Full-text articles excluded: n= 39**

- Did not address outcomes of interest: n=23
- Did not meet systematic review criteria: n=10
- Did not include qualitative data: n=5
- Wrong patient population: n=1

| **Excluded studies references** | **Reason for exclusion** |
| --- | --- |
| Abuogi LL, Smith C, McFarland EJ. Retention of HIV-infected children in the first 12 months of anti-retroviral therapy and predictors of attrition in resource limited settings: A systematic review. PLoS One [Internet]. 2016;11(6 PG-). Available from: https://www.scopus.com/inward/record.uri?eid=2-s2.0-84976333390&partnerID=40&md5=4f5ec054984db581641fba637e283b24 NS - | Wrong Outcomes |
| Aidala AA, Wilson MG, Shubert V, Gogolishvili D, Globerman J, Rueda S, et al. Housing Status, Medical Care, and Health Outcomes Among People Living With HIV/AIDS: A Systematic Review. Am J Public Heal [Internet]. 2016;106(1 PG-e1-e23):e1–23. Available from: NS - | Wrong Outcomes |
| Al-Dakkak I, Patel S, McCann E, Gadkari A, Prajapati G, Maiese EM. The impact of specific HIV treatment-related adverse events on adherence to antiretroviral therapy: a systematic review and meta-analysis. AIDS Care [Internet]. 2013;25(4 PG-400-14):400–14. Available from: NS - | Wrong Outcomes |
| Assawasuwannakit P, Braund R, Duffull SB. A model-based meta-analysis of the influence of factors that impact adherence to medications. J Clin Pharm Ther [Internet]. 2015;40(1 PG-24-31):24–31. Available from: http://search.ebscohost.com/login.aspx?direct=true&db=cin20&AN=103869560&site=ehost-live NS- | Not a systematic review |
| Atkinson MJ, Petrozzino JJ. An evidence-based review of treatment-related determinants of patients’ nonadherence to HIV medications. AIDS Patient Care STDS [Internet]. 2009;23(11 PG-903-14):903–14. Available from: NS - | Not a systematic review |
| Azar MM, Springer SA, Meyer JP, Altice FL. A systematic review of the impact of alcohol use disorders on HIV treatment outcomes, adherence to antiretroviral therapy and health care utilization. Drug Alcohol Depend [Internet]. 2010;112(3 PG-178-93):178–93. Available from: NS - | Wrong Outcomes |
| Bangalore S, Kamalakkannan G, Parkar S, Messerli FH. Fixed-dose combinations improve medication compliance: a meta-analysis (Structured abstract) [Internet]. Vol. 120, American Journal of Medicine. 2007. p. 713–9. Available from: http://onlinelibrary.wiley.com/o/cochrane/cldare/articles/DARE-12007005994/frame.html NS - | Wrong patient population |
| Bharat S. A systematic review of HIV/AIDS-related stigma and discrimination in India: Current understanding and future needs. Sahara j [Internet]. 2011;8(3 PG-138-149):138–49. Available from: https://www.scopus.com/inward/record.uri?eid=2-s2.0-84866171091&partnerID=40&md5=973d85823d67e08e3b6e38ad987e76ab NS - | Wrong Outcomes |
| Chambers LA, Rueda S, Baker DN, Wilson MG, Deutsch R, Raeifar E, et al. Stigma, HIV and health: a qualitative synthesis. BMC Public Health [Internet]. 2015;15(PG-848):848. Available from: NS - | Wrong Outcomes |
| Claborn KR, Meier E, Miller MB, Leffingwell TR. A systematic review of treatment fatigue among HIV-infected patients prescribed antiretroviral therapy. Psychol Heal Med [Internet]. 2015;20(3 PG-255-65):255–65. Available from: NS - | No pre-determined eligibility criteria |
| Colombini M, Stöckl H, Watts C, Zimmerman C, Agamasu E, Mayhew SH. Factors affecting adherence to short-course ARV prophylaxis for preventing mother-to-child transmission of HIV in sub-Saharan Africa: a review and lessons for future elimination. AIDS Care [Internet]. 2014;26(7 PG-914-926):914–26. Available from: http://search.ebscohost.com/login.aspx?direct=true&db=cin20&AN=103936501&site=ehost-live NS - | Wrong Outcomes |
| Costa JM, Torres TS, Coelho LE, Luz PM. Adherence to antiretroviral therapy for HIV/AIDS in Latin America and the Caribbean: Systematic review and meta-analysis. J Int AIDS Soc [Internet]. 2018;21(1 PG-). Available from: NS - | Did not include qualitative studies |
| Darak S, Panditrao M, Parchure R, Kulkarni V, Kulkarni S, Janssen F. Systematic review of public health research on prevention of mother-to-child transmission of HIV in India with focus on provision and utilization of cascade of PMTCT services. BMC Public Health [Internet]. 2012;12(1 PG-). Available from: https://www.scopus.com/inward/record.uri?eid=2-s2.0-84860340493&partnerID=40&md5=3834500b0067d07a7e842d55ea7b6ef5 NS - | Wrong Outcomes |
| Falagas ME, Zarkadoulia EA, Pliatsika PA, Panos G. Socioeconomic status (SES) as a determinant of adherence to treatment in HIV infected patients: a systematic review of the literature. Retrovirology [Internet]. 2008;5(PG-13):13. Available from: NS - | Wrong Outcomes |
| Gari S, Doig-Acuna C, Smail T, Malungo JR, Martin-Hilber A, Merten S. Access to HIV/AIDS care: a systematic review of socio-cultural determinants in low and high income countries. BMC Heal Serv Res [Internet]. 2013;13(PG-198):198. Available from: NS - | Did not include qualitative studies |
| Gemeda DH, Gebretsadik LA, Dejene T, Wolde M, Sudhakar M. Determinants of non-compliance with Antiretroviral Therapy among adults living with HIV/AIDS: A Systematic Review. JBI Libr Syst Rev [Internet]. 2012;10(56 PG-3596-3648):3596–648. Available from: NS - | Did not include qualitative studies |
| Gourlay A, Birdthistle I, Mburu G, Iorpenda K, Wringe A. Barriers and facilitating factors to the uptake of antiretroviral drugs for prevention of mother-to-child transmission of HIV in sub-Saharan Africa: a systematic review. J Int AIDS Soc [Internet]. 2013;16(PG-18588):18588. Available from: NS - | Wrong Outcomes |
| Hall B, Sou K-L, Beanland R, Lacky M, Tso L, Ma Q, et al. Barriers and Facilitators to Interventions Improving Retention in HIV Care: A Qualitative Evidence Meta-Synthesis. AIDS Behav [Internet]. 2017;21(6 PG-1755-1767):1755–67. Available from: http://search.ebscohost.com/login.aspx?direct=true&db=cin20&AN=122919454&site=ehost-live&scope=site NS - | Wrong Outcomes |
| Hiko D, Jemal A, Sudhakar M, Kerie MW, Degene T. Determinants of non-compliance to Antiretroviral Therapy among adults living with HIV/AIDS: A Systematic Review. JBI Libr Syst Rev [Internet]. 2012;10(14 Suppl PG-1-14):1–14. Available from: NS - | Wrong Outcomes |
| Hudelson C, Cluver L. Factors associated with adherence to antiretroviral therapy among adolescents living with HIV/AIDS in low- and middle-income countries: a systematic review. AIDS Care [Internet]. 2015;27(7 PG-805-16):805–16. Available from: NS - | Wrong Outcomes |
| Iwelunmor J, Ezeanolue EE, Airhihenbuwa CO, Obiefune MC, Ezeanolue CO, Ogedegbe GG. Socio-cultural factors influencing the prevention of mother-to-child transmission of HIV in Nigeria: a synthesis of the literature. BMC Public Health [Internet]. 2014;14(PG-771):771. Available from: NS - | Wrong Outcomes |
| Lall P, Lim SH, Khairuddin N, Kamarulzaman A. Review: an urgent need for research on factors impacting adherence to and retention in care among HIV-positive youth and adolescents from key populations. J Int AIDS Soc [Internet]. 2015;18(2 Suppl 1 PG-19393):19393. Available from: NS - | No data extraction in systematic review |
| Ma Q, Tso LS, Rich ZC, Hall BJ, Beanland R, Li H, et al. Barriers and facilitators of interventions for improving antiretroviral therapy adherence: A systematic review of global qualitative evidence: A. J Int AIDS Soc [Internet]. 2016;19(1 PG-). Available from: https://www.scopus.com/inward/record.uri?eid=2-s2.0-85015788116&doi=10.7448%2FIAS.19.1.21166&partnerID=40&md5=1bc81e9ec66cd80d2e41deec63ddacd2 NS - | Wrong Outcomes |
| Malta M, Magnanini MM, Strathdee SA, Bastos FI. Adherence to antiretroviral therapy among HIV-infected drug users: a meta-analysis. AIDS Behav [Internet]. 2010;14(4 PG-731-47):731–47. Available from: NS - | Wrong Outcomes |
| Malta M, Strathdee SA, Magnanini MM, Bastos FI. Adherence to antiretroviral therapy for human immunodeficiency virus/acquired immune deficiency syndrome among drug users: a systematic review. Addiction [Internet]. 2008;103(8 PG-1242-57):1242–57. Available from: NS - | Wrong Outcomes |
| Mbuagbaw L, Thabane L, Ongolo-Zogo P, Yondo D, Noorduyn S, Smieja M, et al. Trends and determining factors associated with adherence to antiretroviral therapy (ART) in Cameroon: a systematic review and analysis of the CAMPS trial. AIDS Res Ther [Internet]. 2012;9(1 PG-37):37. Available from: NS - | Wrong Outcomes |
| Medved Kendrick H. Are religion and spirituality barriers or facilitators to treatment for HIV: a systematic review of the literature. AIDS Care - Psychol Socio-Medical Asp AIDS/HIV [Internet]. 2016;(PG-1-13):1–13. Available from: https://www.scopus.com/inward/record.uri?eid=2-s2.0-84978515022&partnerID=40&md5=e60550155ae06001a987f9d97f8a8b48 NS - | Did not include qualitative studies |
| Mhaskar R, Alandikar V, Emmanuel P, Djulbegovic B, Patel S, Patel A, et al. Adherence to antiretroviral therapy in India: a systematic review and meta-analysis. Indian J Community Med [Internet]. 2013;38(2 PG-74-82):74–82. Available from: NS - | Wrong Outcomes |
| Mugglin C, Estill J, Wandeler G, Bender N, Egger M, Gsponer T, et al. Loss to programme between HIV diagnosis and initiation of antiretroviral therapy in sub-Saharan Africa: systematic review and meta-analysis. Trop Med Int Heal [Internet]. 2012;17(12 PG-1509-20):1509–20. Available from: NS - | Wrong Outcomes |
| Nichols J, Paintsil E, Steinmetz A. Impact of HIV-Status Disclosure on Adherence to Antiretroviral Therapy Among HIV-Infected Children in Resource-Limited Settings: A Systematic Review. AIDS Behav [Internet]. 2017;21(1 PG-59-69):59–69. Available from: http://search.ebscohost.com/login.aspx?direct=true&db=cin20&AN=120570158&site=ehost-live&scope=site NS - | Wrong Outcomes |
| Parsons SK, Cruise PL, Davenport WM, Jones V. Religious beliefs, practices and treatment adherence among individuals with HIV in the southern United States. AIDS Patient Care STDS [Internet]. 2006;20(2 PG-97-111):97–111. Available from: NS - | Not a systematic review |
| Posse M, Meheus F, Van Asten H, Van Der Ven A, Baltussen R. Barriers to access to antiretroviral treatment in developing countries: A review. Trop Med Int Heal. 2008;13(7):904–13. | No data extraction in systematic review |
| Shubber Z, Mills EJ, Nachega JB, Vreeman R, Freitas M, Bock P, et al. Patient-Reported Barriers to Adherence to Antiretroviral Therapy: A Systematic Review and Meta-Analysis. PLoS Med [Internet]. 2016;13(11 PG-e1002183):e1002183. Available from: NS - | Did not include qualitative studies |
| Tso LS, Best J, Beanland R, Doherty M, Lackey M, Ma Q, et al. Facilitators and barriers in HIV linkage to care interventions: A qualitative evidence review. AIDS [Internet]. 2016;30(10 PG-1639-1653):1639–53. Available from: https://www.scopus.com/inward/record.uri?eid=2-s2.0-84964089361&doi=10.1097%2FQAD.0000000000001101&partnerID=40&md5=1e0fdf13e20e18bcdebfd1d634c17821 NS - | Wrong Outcomes |
| Tucker JD, Tso LS, Hall B, Ma Q, Beanland R, Best J, et al. Enhancing Public Health HIV Interventions: A Qualitative Meta-Synthesis and Systematic Review of Studies to Improve Linkage to Care, Adherence, and Retention. EBioMedicine [Internet]. 2017;17(PG-163-171):163–71. Available from: NS - | Wrong Outcomes |
| Varela Arévalo MT, Salazar Torres IC, Correa Sánchez D. Adherence to treatment in HIV/AIDS infection. Theoretical and methodological considerations for dealing with this problem. Acta Colomb Psicol [Internet]. 2009;11(2 PG-101-113):101–13. Available from: https://www.scopus.com/inward/record.uri?eid=2-s2.0-74549209029&partnerID=40&md5=9ec8c6f26fd1a5cbbea815e932b8910b NS - | Not a systematic review |
| Weinstein TL, Li X. The relationship between stress and clinical outcomes for persons living with HIV/AIDS: a systematic review of the global literature. AIDS Care [Internet]. 2016;28(2 PG-160-9):160–9. Available from: NS - | No pre-determined eligibility criteria |
| Wood E, Kerr T, Tyndall MW, Montaner JSG. A review of barriers and facilitators of HIV treatment among injection drug users. Aids [Internet]. 2008;22(PG-1247-56):1247–56. Available from: NS - | No pre-determined eligibility criteria |
| Young S, Wheeler AC, McCoy SI, Weiser SD. A review of the role of food insecurity in adherence to care and treatment among adult and pediatric populations living with HIV and AIDS. AIDS Behav [Internet]. 2014;18 Suppl 5(PG-S505-15):S505-15. Available from: NS - | No pre-determined eligibility criteria |

**Ongoing studies/protocol: n= 3**

| **Ongoing studies/protocol references** | **Reason for exclusion** |
| --- | --- |
| Eshaun-Wilson I, Rohwer A, Hendricks L, Oliver S, Garner P. Adherence, linkage and retention-in-care in antiretroviral treatment programmes in low and middle income countries: systematic review and synthesis of qualitative research. 2017;(PG-). Available from: http://www.crd.york.ac.uk/PROSPERO/display_record.asp?ID=CRD42017057335 NS - | Ongoing Study/ Protocol |
| Norberg A, Nelson J, Holly C, Jewell ST, Salmond S. Experiences of HIV-infected adults and healthcare providers with healthcare delivery practices influencing engagement in primary healthcare settings: a qualitative systematic review protocol. JBI database Syst Rev Implement reports [Internet]. 2017;15(11 PG-2645-2650):2645–50. Available from: NS - | Ongoing Study/  Protocol |
| Protti S, Evans C, Nalubega S. The experience of patients living with human-immunodeficiency virus/tuberculosis co-infection: a systematic review of qualitative evidence protocol. JBI Database Syst Rev Implement Rep [Internet]. 2015;13(7 PG-72-82):72–82. Available from: https://www.scopus.com/inward/record.uri?eid=2-s2.0-84973412396&partnerID=40&md5=d1a03c50cd5a46095635e7ef86e853fa NS - | Ongoing Study/ Protocol |

**Studies awaiting classification: n= 3**

| Costa ASRG, Pessoa IF V, Lopes SPO, Melo RCCP. What are the factors that affect HIV-positive patient adherence to therapeutic regimen: systematic review of the literature. Nurs Rev Form Contin em Enferm [Internet]. 2012;24(281 PG-18-24):18–24. Available from: http://search.ebscohost.com/login.aspx?direct=true&db=cin20&AN=108133139&site=ehost-live NS - | Awaiting appraisal |
| --- | --- |
| Detsis M, Tsioutis C, Karageorgos SA, Sideroglou T, Hatzakis A, Mylonakis E. Factors Associated with HIV Testing and HIV Treatment Adherence: A Systematic Review. Curr Pharm Des [Internet]. 2017;23(18 PG-2568-2578):2568–78. Available from: NS - | Awaiting appraisal |
| Fogarty L, Roter D, Larson S, Burke J, Gillespie J, Levy R. Patient adherence to HIV medication regimens: a review of published and abstract reports. Patient Educ Couns [Internet]. 2002;46(2 PG-93-108):93–108. Available from: NS - | Awaiting appraisal |
