# Supplementary material for: A mega-aggregation framework synthesis of the barriers and facilitators to linkage, adherence to ART and retention in care among people living with HIV
Source: Syst Rev. 2021 Feb 11;10:54. doi: 10.1186/s13643-021-01582-z (PMC7875685; doi:10.1186/s13643-021-01582-z)
Supplement: Supplementary file 8 — Additional file 8. Individual summaries and critical appraisal reasons [file 13643_2021_1582_MOESM8_ESM.docx]

**Additional file 8: Individual summaries and critical appraisal reasons**

*Ammon*

| **Characteristics of included systematic review Ammon et al. 2018** | | |
| --- | --- | --- |
| **Citation** | | |
| Ammon, N., Mason, S. and Corkery, J. M. (2018) ‘Factors impacting antiretroviral therapy adherence among human immunodeficiency virus–positive adolescents in Sub-Saharan Africa: a systematic review’, Public Health. Elsevier Ltd, 157(0), pp. 20–31. doi: 10.1016/j.puhe.2017.12.010. | | |
| **Search Summary Details** | | |
| **Literature Searched** | Peer reviewed databases: Cochrane, PubMed, CINAHL Plus, Scopus, Web of Knowledge, TRIP, Science Direct, Google Scholar; Databases of ongoing research were included, and Open Grey was searched. Relevant publications from reference lists of five articles were retrieved. | |
| **Search Dates** | 3 June 2016 - 15 August 2016 | **Date of last search: 15 August 2016** |
| **Search Criteria** | | |
|  | **What the review authors searched for** | **What the review authors found:** |
| **Studies** | Peer-reviewed, quantitative, qualitative and mixed methods studies conducted after ART induction in 2004, that displayed good quality, with ethical approval and informed participant consent | 11 studies: 7 qualitative studies and 3 mixed methods studies. |
|  |  | **Studies relevant to this overview:** |
|  |  | 11 studies: 7 qualitative studies, 1 quantitative cross-sectional study and 3 mixed methods studies. |
| **Participants** | HIV positive adolescents - vertical and horizontal infection | N=3145 participants: 2937 adolescents aged 10-19; 191 caregivers (parents, non-parental caregiver, biological relative, non-relative, or foster-carer) and 17 healthcare providers. Some ALHIV did not know about their HIV positive status. |
| **Issue** | Adherence | Adherence (n=11) |
| **Setting** | Sub-Saharan Africa | Sub-Saharan Africa: n=1 study each from Congo DRC, Ghana, Kenya, Rwanda, South Africa, Zambia, Zimbabwe and n=2 studies from Uganda. |
| **Outcomes: Barriers and Facilitators** | Patient-related, medication-related, caregiver-related and health system-related factors impacting ART adherence | **Overview Framework:** Individual, Interpersonal, Health System and Structural Factors. |
| **Systematic Review Methods** | | |
| **Conceptual framework:** | No specific framework. Categories developed for study: Patient-related, medication-related, caregiver-related and health system-related factors | |
| **Data extraction method:** | The authors state that a standard template was used but do not report who extracted the data and whether this was done in duplicate. | |
| **Appraisal tool used** | National Heart, Lung and Blood Institute - Quality Assessment Tool for Observational Cohort and Cross-Sectional Studies: https://www.nhlbi.nih.gov/health-topics/study-quality-assessment-tools | |
| **Data synthesis method:** | Thematic Synthesis | |

| **JBI Quality Appraisal** | | |
| --- | --- | --- |
| **Question** | **Judgement** | **Justification** |
| **1. Is the review questions clearly and explicitly stated?** | Yes | This systematic review aims to identify factors that enable and impede ART adherence among ALHIV in SSA. Question clearly stated. |
| **2. Were the inclusion criteria appropriate for the review question?** | Yes | Quantitative (cross sectional), qualitative and mixed methods studies that addressed factors impacting ART adherence in SSA ALHIV of all languages were included. Studies with populations of children <10 years and adults were excluded. Potentially eligible studies met the following criteria: (1) used a qualitative, quantitative or mixed methods design; (2) were peer-reviewed original studies; (3) were studies conducted after ART induction in SSA in 2004; (4) discussed and evaluated an association between ART adherence among ALHIV in SSA and at least one patient-related, medication-related, caregiver-related or health system related factor; and (5) displayed good quality, with ethical approval and informed participant consent. Only English studies were included. The review has been judged as a Yes as inclusion criteria 1-4 are appropriate. We do not agree that inclusion criteria 5 "displaying good quality' was appropriate as this is part of the review process after inclusion. |
| **3. Was the search strategy appropriate?** | Yes | The supplementary material provided evidence of search strategy, MESH terms, key terms and search filters from 3 June 2016 - 15 August 2016. Only included English studies. |
| **4. Were the sources and resources used to search for studies adequate?** | Unclear | Review searched 8 electronic databases. Cochrane, PubMed, CINAHL Plus, Scopus, Web of Knowledge, TRIP, Science Direct, Google Scholar. Databases of ongoing research were included, and Open Grey was searched. Relevant publications from reference lists of five articles were retrieved and screened for duplications. Judgement is unsure as the authors report that papers had to be peer-reviewed, but they also indicate that they searched Opengrey, which is a database of grey literature (i.e. unpublished and not peer-reviewed). Have contacted author for clarity and await response. |
| **5. Was selection of studies done adequately?** | Unclear | After removal of duplicates, one investigator, using predefined inclusion and exclusion criteria, reviewed all titles, abstracts and full-text articles. A second reviewer screened and assessed the studies independently. It is unclear whether title and abstract screening was done in duplicate. Have contacted author for clarity and await their response. |
| **6. Were the criteria for appraising studies appropriate?** | Unclear | Before inclusion, cross-sectional studies were appraised for quality of evidence, using the appraisal tool from the National Institutes of Health. For qualitative studies, the Critical Appraisal Skills Programme was used. Criteria or specific domains and results of appraisal not reported |
| **7. Was the critical appraisal conducted by two or more reviewers independently?** | Unclear | Not reported |
| **8. Were there methods to minimize errors in the data extraction?** | Unclear | The authors state that a standard template was used but do not report who extracted the data and whether this was done in duplicate. |
| **9. Were the methods used to combine studies appropriate?** | Yes | Thematic synthesis was used. Data from qualitative studies were extracted using thematic analysis with results sorted into four main categories: patient-related, medication-related, caregiver related and health system-related factors. These categories were further divided into facilitators and barriers. Quantitative data results were listed under the same headings. Finally, findings were recorded according to frequency of topics occurring across studies. |
| **10. Were recommendations for policy and/or practice supported by the reported data?** | Yes | The evidence of this review strongly suggests that culturally appropriate, large-scale campaigns, also aimed at schools, including religious authorities, could change attitudes in the wider society and create greater acceptance of ALHIV. A film produced by Picturing Health will highlight the findings of this review and aid in guiding researchers, policy-makers and healthcare workers involved with ALHIV. |
| **11. Were the specific directives for new research appropriate?** | No | General recommendations for interventions were provided. However, no specific research directives provided. |

*Barasso*

| **Characteristics of included systematic review Barasso et al. 2017** | | |
| --- | --- | --- |
| **Citation** | | |
| Barroso, J., Leblanc, N. M. and Flores, D. (2017) ‘It’s Not Just the Pills: A Qualitative Meta-Synthesis of HIV Antiretroviral Adherence Research’, Journal of the Association of Nurses in AIDS Care. Elsevier Inc, 28(4), pp. 462–478. doi: 10.1016/j.jana.2017.02.007. | | |
| **Search Summary Details** | | |
| **Literature Searched** | Peer reviewed databases search included: PubMed, PsychINFO and CINAHL were searched. No fugitive or grey literature was searched+B20. | |
| **Search Dates** | 2008-2013 | **Date of last search: 2013** |
| **Search Criteria and Outcomes** | | |
|  | **What the review authors searched for** | **What the review authors found:** |
| **Studies** | Searched for peer-reviewed English only qualitative studies including mixed methods studies which contained qualitative data. | 127 studies: Qualitative Studies (127) |
|  |  | **Studies relevant to this overview:** |
|  |  | 127 studies: Qualitative Studies (127) |
| **Participants** | HIV positive people age 13 years and older. | N=6189 participants: n=4830 PLHIV (2197 female and 1850 male, 783 unspecified) and n=1359 included provider participants (caregivers, health care providers, traditional healers, local community leaders, pharmacists, policymakers, stakeholders, peer counselors, facility managers, volunteers, and clinical trial coordinators). |
| **Issue** | Linkage to ART and Adherence | Linkage to ART (127) and Adherence (127). |
| **Setting** | Anywhere in the world | The most frequent places of data collection were the United States (28 reports), South Africa (19 reports), Uganda (16 reports), Europe (9 reports), Zambia (9 reports), Tanzania (8 reports), Nigeria (5 reports), and China (5 reports). All other locations for data collection contributed to fewer than five reports. |
| **Outcomes: Barriers and Facilitators** | Patient reported barriers to adherence | **Overview Framework:** Individual, Interpersonal, Community, Health System and Structural Factors. |
| **Systematic Review Methods** | | |
| **Conceptual framework:** | Maslow's theory of human motivation | |
| **Data extraction method:** | Data extracted into EXCEL and checked between authors | |
| **Appraisal tool used** | No appraisal was conducted | |
| **Data synthesis method:** | Thematic Synthesis | |

| **JBI Quality Appraisal** | | |
| --- | --- | --- |
| **Question** | **Judgement** | **Justification** |
| **1. Is the review questions clearly and explicitly stated?** | Yes | Synthesising the qualitative findings published between 2008-2013 related to starting ART and remaining of ART until viral suppression is achieved. Question clearly stated. |
| **2. Were the inclusion criteria appropriate for the review question?** | Yes | Reviewed qualitative research from anywhere in the world, published in English from 2008-2013 that (a) were conducted with people ages 13 years and older (we used this age cut-off as we were interested in those who could take their own medications, as opposed to children who were reliant on a caregiver to do so); and (b) involved some aspect of the HIV treatment cascade. Inclusion criteria was deliberatley broad as one search was conducted for three different reviews. |
| **3. Was the search strategy appropriate?** | Yes | Review searched 3 electronic databases. Review provides list of key terms used to search between 2008-2013. Information regarding search strategy string available in connected publication (Flores 2016). Strategy is adequate. English only studies. |
| **4. Were the sources and resources used to search for studies adequate?** | No | PubMed, PsychINFO and CINAHL were searched. No fugitive or grey literature was searched. |
| **5. Was selection of studies done adequately?** | Unclear | No information provided on the screening and selection of studies. |
| **6. Were the criteria for appraising studies appropriate?** | No | No appraisal was conducted on included articles. |
| **7. Was the critical appraisal conducted by two or more reviewers independently?** | No | No appraisal was conducted on included articles. |
| **8. Were there methods to minimize errors in the data extraction?** | Yes | Data was captured into EXCEL including authors, demographic characteristics of the study sample, and findings that related to starting on or remaining adherent to ART. They conducted quality checks on one another; one person would extract the findings and another would conduct random validations, both of the articles that were excluded (to ensure that they were correctly categorized as excluded) and of those that were included (to ensure that the data were extracted correctly). |
| **9. Were the methods used to combine studies appropriate?** | Yes | Thematic synthesis was used by initially creating 150 statements of findings followed by the aggregation of similar findings into more abstract, encompassing themes, maintaining careful and close adherence to the original meaning. Statements that captured the findings in the studies were developed. Spreadsheet of the findings for reports that contained that finding were scanned and all studies that contained that finding listed. Similar statements were linked together, under more encompassing themes, to develop cogent statements about ART adherence. At all analysis points, authors worked together, sending work electronically back and forth for validation of themes, refinement of findings, discussions of potential theoretical frameworks to elaborate on the findings, and the development of manuscripts. |
| **10. Were recommendations for policy and/or practice supported by the reported data?** | Yes | Policies and strategies that reduce the burden associated with care is recommended. The review provides recommendations to move away from individual centred care to community and social networks. Recommendations are further explored through citing a previous study Ellman (2015) and drawing correlations to the review findings. This was considered adequate. |
| **11. Were the specific directives for new research appropriate?** | No | No research recommendations provided. |

*Bolsewicz*

| **Characteristics of included systematic review Bolsewicz et al. 2015** | | |
| --- | --- | --- |
| **Citation** | | |
| Bolsewicz, K. et al. (2015) ‘Factors associated with antiretroviral treatment uptake and adherence: A review. Perspectives from Australia, Canada, and the United Kingdom’, AIDS Care - Psychological and Socio-Medical Aspects of AIDS/HIV, 27(12), pp. 1429–1438. doi: 10.1080/09540121.2015.1114992. | | |
| **Search Summary Details** | | |
| **Literature Searched** | Scopus, OVID-EMBASE, CSAillumina, CINAHL, PROQuest, Web of Science, Informit and unpublished reports and grey literature selected based on recommendations and implications for the national and regional policies | |
| **Search Dates** | 2003 - 2013 | **Date of last search:** Not specified |
| **Search Criteria and Outcomes** | | |
|  | **What the review authors searched for** | **What the review authors found relevant to this overview** |
| **Studies** | Searched for primary studies and reviews published in English, that included search terms in the title, keywords and abstracts. All study designs included | 17 Studies and 11 reports |
|  |  | **Studies relevant to this overview:** |
|  |  | 17 studies and 2 reports |
| **Participants** | People living with HIV, excluding drug users, mothers, adolescents, prisoners, sex workers in Canada, UK and Australia | People living with HIV, excluding drug users, mothers, adolescents, prisoners, sex workers in Canada, UK and Australia |
| **Issue** | Linkage to ART and adherence | Linkage to ART (19) and Adherence (19) |
| **Setting** | High income countries: Canada, Australia and the UK | 3 High income countries: Studies were from Canada (8), UK (3) and Australia (6) (HIC), reports not reported. All from the North American region |
| **Outcomes: Barriers and Facilitators** | Factors associated with initiating treatment (linkage) and staying on treatment (adherence) | **Overview framework:** Individual, Interpersonal, Community and Social, Political and Health System, and Structural Factors. |
| **Systematic Review Methods** | | |
| **Conceptual framework:** | Categorization of barriers by Begley and colleagues (2008) into intrapersonal, interpersonal and extrapersonal | |
| **Data extraction method:** | None reported | |
| **Appraisal tool used** | None reported | |
| **Data synthesis method:** | Thematic Synthesis | |

| **JBI Quality Appraisal** | | |
| --- | --- | --- |
| **Question** | **Judgement** | **Justification** |
| **1. Is the review questions clearly and explicitly stated?** | Yes | Reviewed the literature on factors associated with initiating and staying on treatment in the general adult population in Australia, Canada and the UK using an established framework of intrapersonal, interpersonal and extrapersonal. Question clearly stated. |
| **2. Were the inclusion criteria appropriate for the review question?** | Yes | included studies only from Canada, UK and Australia.Primary studies and reviews published in English. Articles restricted to those that include the review search terms. Studies focusing on specific populations were excluded. |
| **3. Was the search strategy appropriate?** | Yes | Search strategy available and keywords clearly indicated. All PICO elements contained within the search strategy. English only. |
| **4. Were the sources and resources used to search for studies adequate?** | Yes | Peer-reviewed and grey literature which informed two reviews. Medical, allied health, and social sciences online libraries: Scopus, OVID-EMBASE, CSAillumina, CINAHL, PROQuest, Web of Science, Informit and unpublished reports selected based on recommendations and implications for the national and regional policies |
| **5. Was selection of studies done adequately?** | Unclear | Study selection and screening not reported. |
| **6. Were the criteria for appraising studies appropriate?** | Unclear | Appraisal not reported. |
| **7. Was the critical appraisal conducted by two or more reviewers independently?** | Unclear | Appraisal not reported. |
| **8. Were there methods to minimize errors in the data extraction?** | Unclear | Evidence of tools used to minimise error in data extraction not reported. |
| **9. Were the methods used to combine studies appropriate?** | Yes | Used Begley (2008) categories to synthesis the data. broadly categorized into intrapersonal (cognitive or psychological processes experienced by the individual, including person’s attitudes, perception, and beliefs); interpersonal (relationship variables including relationship with health providers, community, and family; and social support); and extrapersonal (external variables including systemic, structural and demographic issues, experiences of illness, lifestyle, and treatment factors). Factors identified fit into the prescribed categories. |
| **10. Were recommendations for policy and/or practice supported by the reported data?** | Yes | ART not universal: enhance scope for people with HIV and for doctors to initiate ART; remove financial barriers to treatment uptake arising from dispensing fees, establish programmes to provide ART to people not eligible for Medicare cover. ART universal: improve accessibility to low barrier housing to homeless, expand adherence support programmes, provide services locally, "one stop shop" model of care, stable housing and support from family (especially in aboriginal youth). Recommendations are supported by the data. |
| **11. Were the specific directives for new research appropriate?** | Yes | Qualitative social research within HIV-positive communities is urgently needed to capture people’s lived experiences and may address some of this deﬁcit in understanding. Supported by the study finding gaps in evidence. |

*Bravo*

| **Characteristics of included systematic review Bravo et al. 2010** | | |
| --- | --- | --- |
| **Citation** | | |
| Bravo P, Edwards A, Rollnick S, and Elwyn E. 2010. Tough decisions faced by people living with HIV: a literature review of psychosocial problems. AIDS Rev, 12(2 PG-76-88), pp.76–88. | | |
| **Search Summary Details** | | |
| **Literature Searched** | Peer reviewed (Web of Science, Scopus, ProQuest and PubMed and reference lists of literature reviews not included. | |
| **Search Dates** | 1990-2009 | **Date of last search**: November 2009 |
| **Search Criteria and Outcomes** | | |
|  | **What the review authors searched for** | **What the review authors found** |
| **Studies** | Primary studies and reviews published in any language, that included search terms in the title, keywords and abstracts. All study designs included. Literature reviews excluded. | 46 studies: 21 qualitative studies, 21 quantitative cross-sectional studies and 4 mixed methods studies. |
|  |  | **Studies relevant to this overview:** |
|  |  | 10 studies, 5 qualitative and 5 quantitative cross sectional studies |
| **Participants** | People living with HIV | N=4215 PLHIV including drug users and women caring for children <18 years; n= 4022 in Quantitative and n=193 Qualitative studies. |
| **Issue** | Decision support needs testing, linkage and adherence | Linkage to ART (10) and Adherence (10) |
| **Setting** | All countries | 3 High income countries: USA (7), UK (1), France (1) and 1 Low to middle income country: Botswana (1). Across the North American and African region |
| **Outcomes: Barriers and Facilitators** | Barriers: Decision making dilemmas and Facilitators: Psychosocial needs for decision making | **Overview framework:** Individual, Interpersonal, Community and Social, Political and Health System, and Structural Factors. |
| **Systematic Review Methods** | | |
| **Conceptual framework:** | Paper aimed to build model based on findings - Meta synthesis | |
| **Data extraction method:** | Details of studies extracted but whether it was done in duplicate is not reported. | |
| **Appraisal tool used** | No appraisal was reported | |
| **Data synthesis method:** | Thematic Analysis: Meta-synthesis to develop model | |

| **JBI Quality Appraisal** | | |
| --- | --- | --- |
| **Question** | **Judgement** | **Justification** |
| **1. Is the review questions clearly and explicitly stated?** | Yes | To determine whether or not PLHIV have decision support needs, and if they do, to describe these needs in more detail in order to consider to what extent they could be met by the development of new services or interventions. Question clearly stated. |
| **2. Were the inclusion criteria appropriate for the review question?** | Yes | Articles were included if they described psychosocial needs or concerns of PLHV from 1990 to 2009. All research methods were eligible. Literature review articles were excluded, but their references assessed. |
| **3. Was the search strategy appropriate?** | No | Full search strategy not available but the search strategy included the following terms: (HIV) AND (decision making; OR decision need; OR decision) AND (psychosocial; OR psychological; OR social). All languages were included. |
| **4. Were the sources and resources used to search for studies adequate?** | No | Electronic searches of the following sources from 1990-2009: Web of Science, Scopus, ProQuest, and PubMed. We also specifically searched the following journals (AIDS and Behavior, AIDS Care, and Social Science and Medicine). Web of science, Scopus, ProQuest and PubMed databases. No grey literature searched. Reference list of excluded literature reviews were assessed. |
| **5. Was selection of studies done adequately?** | Unclear | Studies were assessed for relevance but not in duplicate or independently by two reviewers. |
| **6. Were the criteria for appraising studies appropriate?** | Unclear | Appraisal not reported. |
| **7. Was the critical appraisal conducted by two or more reviewers independently?** | Unclear | Appraisal not reported. |
| **8. Were there methods to minimize errors in the data extraction?** | Unclear | Data was extracted for research identification and research methods. Not reported whether data extraction was done in duplicate |
| **9. Were the methods used to combine studies appropriate?** | Yes | The study used thematic analysis. |
| **10. Were recommendations for policy and/or practice supported by the reported data?** | Yes | Provide rapid support around the time of diagnosis, helping people with disclosure issues and providing guidance for all other behavioural descisions. Interventions should incirporate elements to reduce stigma and discrimination against PLHIV, incoporating them in all the development process in order to empower them and provide a sensitive and effective psychosocial intervention. |
| **11. Were the specific directives for new research appropriate?** | Yes | Valuable to examine the influence on undertaking or not undertaking HIV tests and the decisions around this. |

*Chop*

| **Characteristics of included systematic review Chop et al. 2017** | | |
| --- | --- | --- |
| **Citation** | | |
| Chop, E. et al. (2017) ‘Food insecurity, sexual risk behavior, and adherence to antiretroviral therapy among women living with HIV: A systematic review’, Health Care for Women International. Taylor & Francis, 38(9), pp. 927–944. doi: 10.1080/07399332.2017.1337774. | | |
| **Search Summary Details** | | |
| **Literature Searched** | PubMed, CINAHL (Cumulative Index to Nursing and Allied Health Literature), PsycINFO, and Sociological Abstracts. A secondary reference search was conducted on all studies included in the review, as well as articles included in two previous reviews of food insecurity and ART adherence among people living with HIV (Singer et al., 2015; Young et al., 2014). | |
| **Search Dates** | Up to 18 February 2018 | **Date of last search: 18 February 2016** |
| **Search Criteria and Outcomes** | | |
|  | **What the review authors searched for** | **What the review authors found:** |
| **Studies** | Any study with primary data, either quantitative or qualitative, published in a peer-reviewed journal | N=7 studies: n=5 qualitative studies, and n=1 quantitative prospective cohort study. |
|  |  | **Studies relevant to this overview:** |
|  |  | 4 studies: 3 qualitative studies and n=1 quantitative cross-sectional study |
| **Participants** | Women living with HIV | Women living with HIV |
| **Issue** | Association between food insecurity and risky sexual behaviour and adherence | Adherence (4) |
| **Setting** | All countries | 3 LMIC: Zambia (1), Swaziland (1) and Democratic Republic of Congo (1) and 1 HIC: France (1) |
| **Outcomes: Barriers and Facilitators** | Food insecurity | **Overview Framework:** Structural Factors |
| **Systematic Review Methods** | | |
| **Conceptual framework:** | None reported | |
| **Data extraction method:** | Done in duplicate by two authors. | |
| **Appraisal tool used** | Qualitative studies not appraised. | |
| **Data synthesis method:** | Thematic analysis | |

| **JBI Quality Appraisal** | | |
| --- | --- | --- |
| **Question** | **Judgement** | **Justification** |
| **1. Is the review questions clearly and explicitly stated?** | Yes | The review aimed to review the impact of food insecurity on sexual risk behaviour and ART adherence among women living with HIV. Question clearly stated. |
| **2. Were the inclusion criteria appropriate for the review question?** | Yes | Studies had to contain primary data from qualitative or quantitative study designs, collected from women living with HIV, describe relationships between food insecurity and ART adherence, safer sex or sexual risk behaviours, and be published in a peer-reviewed journal prior to February 18, 2016. No restriction was placed on location. The inlcusion criteria fits the scope of the review question. |
| **3. Was the search strategy appropriate?** | Unclear | Evidence of the search strategy is provided as well as key terms. The search did not include the concept of 'adherence' in order to broaden to the search and not miss any studies. Did not include a MeSH term for HIV and did not use HIV on its own. Also not sure whether including terms for women would narrow down the search too much as more studies could have been found in the search that contain men and women. |
| **4. Were the sources and resources used to search for studies adequate?** | No | PubMed, CINAHL (Cumulative Index to Nursing and Allied Health Literature), PsycINFO, and Sociological Abstracts. A secondary reference search was conducted on all studies included in the review, as well as articles included in two previous reviews of food insecurity and ART adherence among people living with HIV (Singer et al., 2015; Young et al., 2014). Judgement is no as no grey literature searched or sourced. |
| **5. Was selection of studies done adequately?** | No | Titles, abstracts, citation information, and descriptor terms of citations identified through the search strategy were screened by only one study staff member. Full text screening was done in duplicate by two authors. |
| **6. Were the criteria for appraising studies appropriate?** | No | Qualitative studies were not appraised. Quantitative studies were appraised with the approach designed by the Evidence Project (Denison, O’Reilly, Schmid, Kennedy, & Sweat, 2008; Medley, Kennedy, O’Reilly, & Sweat, 2009). |
| **7. Was the critical appraisal conducted by two or more reviewers independently?** | Unclear | Appraisal procedure not reported |
| **8. Were there methods to minimize errors in the data extraction?** | Yes | Data extraction with differences in interpretation between reviewers was done in duplicate by two authors and resolved through discussion. |
| **9. Were the methods used to combine studies appropriate?** | Unclear | Process of synthesis not reported. |
| **10. Were recommendations for policy and/or practice supported by the reported data?** | Yes | The HIV response must create an enabling environment for women living with HIV by implementing programs that reduce hunger and food insecurity, especially for women. Programs should address the political, social, and economic contexts in which women experience food insecurity. Evidence supports this recommendation. |
| **11. Were the specific directives for new research appropriate?** | Yes | The review suggests that more research is needed that specifically addresses food security, sexual risk behavior, and ART adherence among women living with HIV. Furthermore, disaggregation of results by sex would allow future researchers to better understand gender dynamics of HIV and food insecurity. This is adequate. |

*Colvin*

| **Characteristics of included systematic review Colvin et al. 2014** | | |
| --- | --- | --- |
| **Citation** | | |
| Colvin, C.J. et al., 2014. A systematic review of health system barriers and enablers for antiretroviral therapy (ART) for HIV-infected pregnant and postpartum women. PLoS One, 9(10 PG-e108150), p.e108150. | | |
| **Search Summary Details** | | |
| **Literature Searched** | Peer reviewed (PubMed and Social Science Citation Index) and Websites searched include: United Nations Joint Program on HIV/AIDS (UNAIDS), World Health Organization (WHO), United States Agency for International Development (USAID) – Development Experience Clearinghouse (DEC), ICAP, Columbia University, Elizabeth Glaser Pediatric AIDS Foundation (EGPAF), Pathfinder International,International AIDS Society (IAS), Conference on Retroviruses and Opportunistic Infections (CROI), International Society For Sexually Transmitted Diseases Research (ISSTDR), International Conferences on Improving Use of Medicines (ICIUM). | |
| **Search Dates** | 1 January 2008-26 March 2013 | **Date of last search:** March 2013 |
| **Search Criteria and Outcomes** | | |
|  | **What the review authors searched for** | **What the review authors found** |
| **Studies** | All study designs relevant to the research question. Literature reviews excluded. Only English language included. | 42 studies: 14 qualitative studies, 25 quantitative studies and 3 mixed methods studies. |
|  |  | **Studies relevant to this overview:** |
|  |  | 42 studies: 14 qualitative studies, 25 quantitative studies and 3 mixed methods studies. |
| **Participants** | Pregnant and post partum women with HIV | HIV-infected pregnant and/or postpartum women and/or health care providers delivering antenatal care, ART and/or PMTCT. A few studies included partners and/or family members of HIV-infected pregnant or postpartum women. Participants: n=875 308 |
| **Issue** | Linkage, Adherence and Retention | Linkage to ART (30), Adherence (9) and Retention (10) from 42 studies |
| **Setting** | HIC and LMIC | LMIC: Sub-Saharan Africa (38) and Latin America (2) and Asia (2) |
| **Outcomes: Barriers and Facilitators** | Health system barriers and enablers of ART | **Overview Framework**: Politicial and Health System Factors. |
| **Systematic Review Methods** | | |
| **Conceptual framework:** | Grounded theory approach | |
| **Data extraction method:** | Used a stadardised form independently and in duplicate. | |
| **Appraisal tool used** | Appraisal tool not reported. | |
| **Data synthesis method:** | 4 stage narrative synthesis - Meta synthesis | |

| **JBI Quality Appraisal** | | |
| --- | --- | --- |
| **Appraisal tool used** | **Judgement** | **Justification** |
| **1. Is the review questions clearly and explicitly stated?** | Yes | Health system barriers and enablers for ART for HIV infected pregnant and postpartum women. Question clearly stated. |
| **2. Were the inclusion criteria appropriate for the review question?** | Yes | Any empirical qualitative or quantitative findings relevant to the review was included. All countries. Studies on community, primary care or tertiary care settings. Only English language included. |
| **3. Was the search strategy appropriate?** | Yes | For the database searches, used variations of search terms for 1) the population of interest (pregnant or postpartum women with HIV, 2) the intervention of interest (antiretroviral therapy) and 3) the outcomes of interest (initiation, adherence, retention). A full search strategy for one of the database searches can be found in the Supporting Information. Search was specific to maternal ART and could have included search on adult ART and PMTCT |
| **4. Were the sources and resources used to search for studies adequate?** | Yes | To identify eligible studies, first searched for peer-reviewed publications through the PubMed and Social Science Citation Index databases.Additional studies were identified through a gray literature search of conference abstracts, program reports, and government documentation related to ART for HIV-infected pregnant or postpartum women. A list of websites searched for gray literature available in Supporting Information. Websites searched include: United Nations Joint Program on HIV/AIDS (UNAIDS), World Health Organization (WHO), United States Agency for International Development (USAID) – Development Experience Clearinghouse (DEC), ICAP, Columbia University, Elizabeth Glaser Pediatric AIDS Foundation (EGPAF), Pathfinder International,International AIDS Society (IAS), Conference on Retroviruses and Opportunistic Infections (CROI), International Society For Sexually Transmitted Diseases Research (ISSTDR), International Conferences on Improving Use of Medicines (ICIUM). Only English articles were included. Searched studies available in published literature and grey literature through website search. |
| **5. Was selection of studies done adequately?** | Unclear | The first 100 citations were checked in duplicate but there is no description of the method of agreement was or how they checked the other citations. |
| **6. Were the criteria for appraising studies appropriate?** | Unclear | Ranked each included study as low, medium or high with respect to overall risk of bias, based on its sample size, selection criteria, sampling procedure and data analysis method. Appraisal not reported. |
| **7. Was the critical appraisal conducted by two or more reviewers independently?** | Unclear | Not described whether critical appraisal was done by 2 authors independently |
| **8. Were there methods to minimize errors in the data extraction?** | Yes | One author extracted data regarding study characteristics and key findings using a standard template, another author reviewed the studies and extracted data related to program outcomes and key health system factors. Extracted findings from both were compared and discrepancies resolved. |
| **9. Were the methods used to combine studies appropriate?** | Yes | A four-stage narrative synthesis design was used: 1. Developing a theoretical model of how the interventions work, why and for whom using the WHO Building Blocks Model, SURE Framework, and Review Scoping Discussion among team members 2. Developing a preliminary synthesis using Thematic analysis 3. Exploring relationships in the data using Comparative case analysis 4. Assessing the robustness of the synthesis product using Narrative assessment guided by 4 questions about strength and 2 questions about generalizability. |
| **10. Were recommendations for policy and/or practice supported by the reported data?** | Yes | Findings are supportive of the health systems recommendations made. Recommendations are detailed and show clear links to the data and themes. |
| **11. Were the specific directives for new research appropriate?** | Yes | Critical gaps in evidence regarding maternal ART including adherence outcomes, factors affecting adherence, the role of timeliness and timing as discrete variables, barriers and enablers for those who never make it to health services and those who drop out, and maternal ART outcomes along the full cascade and using a variety of denominators for different comparative evaluation. *Programme evaluations using strong, prospective research designs in pragmatic settings should be prioritised in order to better characterise likely maternal ART outcomes and challenges in settings outside small pilot interventions. Measures of adherence should be standardised to enable comparison across programs and studies. Specific directives for research provided. |

*Croome*

| **Characteristics of included systematic review Croome et al. 2017** | | |
| --- | --- | --- |
| **Citation** | | |
| Croome, N. et al. (2017) ‘Patient-reported barriers and facilitators to antiretroviral adherence in sub-Saharan Africa’, Aids, 31(7), pp. 995–1007. doi: 10.1097/QAD.0000000000001416. | | |
| **Search Summary Details** | | |
| **Literature Searched** | Peer reviewed databases: 10 databases: Cochrane Library, MEDLINE (OVID), PsycINFO (OVID), PsycARTICLES (OVID), EMBASE (OVID), Global Health (OVID), CINAHL (EBSCO), International Bibliography of the Social Science (IBSS; Proquest), Applied Social Sciences Index and Abstracts (AIDIA; Proquest). Hand-searching, web-searching and forward citation searching were conducted to find other relevant published and unpublished studies. Abstracts from the following international conferences were searched: International AIDS Society (IAS; 2005–2015), Conference on Retroviruses and Opportunistic Infections (CROI; 2014, 2016), AIDS Impact (2009–2015) and International AIDS Conference (2006–2014). For all potentially relevant abstracts, if no published article was found, the authors were contacted for more details if possible. Reference lists of included articles and systematic reviews were searched. Searches in Google Scholar were conducted using search terms such as ‘adherence’, ‘HIV’ and ‘Sub-Saharan Africa’. Forward tracking was also performed in the Web of Science database. | |
| **Search Dates** | 2005 to 24 May 2016 | **Date of last search: 24 May 2016** |
| **Search Criteria** | | |
|  | **What the review authors searched for** | **What the review authors found:** |
| **Studies** | Searched for peer-reviewed and reference list all-language quantitative surveys and qualitative studies | 154 studies: 83 qualitative studies, 67 quantitative cross-sectional studies and 4 mixed methods studies. |
|  |  | **Studies relevant to this overview:** |
|  |  | 154 studies: 83 qualitative studies, 67 quantitative cross-sectional studies and 4 mixed methods studies |
| **Participants** | Adult HIV positive patient | N=37175 Adult HIV positive participants |
| **Issue** | Adherence | Adherence (154) |
| **Setting** | Sub-Saharan Africa | Benin, Cote d'lvoire and Mali (1), Botswana (3) Burkina Faso (1), Cameroon (4) Cote d'lvoire (1), DRC (2), Ethiopia (20), Ethiopia and Uganda (1), Ghana (4), Guinea-Bissau (1), Kenya (16), Kenya and Malawi (1), Kenya and Uganda (1), Lesotho (1), Malawi (2), Mali (1), Mozambique (3), Namibia (4), Nigeria (13), Nigeria, Tanzania and Uganda (1), Rwanda (3), Senegal (1), South Africa (30), Tanzania (10), Tanzania, Uganda and Zambia (1), Togo (1), Uganda (19), Zambia (6), Zimbabwe (2) |
| **Outcomes: Barriers and Facilitators** | Patient reported barriers to ART adherence | **Overview Framework:** Individual, Interpersonal, Community and Social, Politicial and Health System, and Structural Factors. |
| **Systematic Review Methods** | | |
| **Conceptual framework:** | No framework reported | |
| **Data extraction method:** | Extracted into pre-defined EXCEL spreadsheet | |
| **Appraisal tool used** | Relevance, Appropriateness, Transparency, Soundness (RATS) measure used to appraise qualitative studies and Hawker et al (2016) measure used to assess quantitative studies. | |
| **Data synthesis method:** | Content analysis | |

| **JBI Quality Appraisal** | | |
| --- | --- | --- |
| **Question** | **Judgement** | **Justification** |
| **1. Is the review questions clearly and explicitly stated?** | Yes | The review identifies adult patient-reported barriers and facilitators to ART adherence in SSA from 2005 to 2016 in studies with qualitative and quantitative methodology. |
| **2. Were the inclusion criteria appropriate for the review question?** | Yes | Eligible studies met the following criteria: based in SSA, original research, any language, qualitative study or quantitative survey and included an adult patient-reported barrier or facilitator to ART adherence. Studies were excluded if they only focused on initiation to ART in treatment-naive participants, only utilized a single dose of ART treatment, for example prophylaxis, only focused on Africans living in a non-SSA country or were only reviews. |
| **3. Was the search strategy appropriate?** | Yes | Review searched 10 electronic databases. Provided evidence of search strategy, MESH terms, key terms and search filters from 2005 to 24 May 2016. All languages were included. |
| **4. Were the sources and resources used to search for studies adequate?** | Yes | Peer reviewed databases: 10 databases: Cochrane Library, MEDLINE (OVID), PsycINFO (OVID), PsycARTICLES (OVID), EMBASE (OVID), Global Health (OVID), CINAHL (EBSCO), International Bibliography of the Social Science (IBSS; Proquest), Applied Social Sciences Index and Abstracts (AIDIA; Proquest). Hand searching, web searching and forward citation searching were conducted to find other relevant published and unpublished studies. Abstracts from the following international conferences were searched: International AIDS Society (IAS; 2005–2015), Conference on Retroviruses and Opportunistic Infections (CROI; 2014, 2016), AIDS Impact (2009–2015) and International AIDS Conference (2006–2014). For all potentially relevant abstracts, if no published article was found, the authors were contacted for more details if possible. Reference lists of included articles and systematic reviews were searched. Searches in Google Scholar were conducted using search terms such as ‘adherence’, ‘HIV’ and ‘Sub-Saharan Africa’. Forward tracking was also performed in the Web of Science database. The authors searched a number of sources for published and unpublished papers. |
| **5. Was selection of studies done adequately?** | No | One author reviewed all the titles and abstracts of each record to assess potential relevance, a second author examined a random sample (10%), and a concordance rate was measured. Acceptable concordance was predefined as agreement on at least 90%. The full text screening was only conducted by one author. |
| **6. Were the criteria for appraising studies appropriate?** | Yes | Relevance, Appropriateness, Transparency, Soundness (RATS) measure used to appraise qualitative studies and Hawker et al (2016) measure used to assess quantitative studies. |
| **7. Was the critical appraisal conducted by two or more reviewers independently?** | No | Appraisal conducted by a single author |
| **8. Were there methods to minimize errors in the data extraction?** | Unclear | One author extracted all the data into a predesigned Excel spreadsheet that was double-checked for accuracy. This was not adequate for a 'Yes'. We are unsure whether the author double checked their own work or if the data extracted was checked by someone else. Author has been contacted and we await their response. |
| **9. Were the methods used to combine studies appropriate?** | Yes | After an initial read of all qualitative papers, a list of all key barriers and facilitators were identified and were combined into themes by three authors who discussed the themes and any disputes were resolved |
| **10. Were recommendations for policy and/or practice supported by the reported data?** | Yes | Factors including traditional medicines, religious beliefs or treatments, lack of access to food and sharing medication need to be acknowledged when working with people living with HIV in SSA and need particular consideration when designing interventions to improve ART adherence. |
| **11. Were the specific directives for new research appropriate?** | Yes | Frequency across studies does not necessarily equate with importance for individuals; therefore, the most reported factors may not have the greatest impact upon adherence. Future studies should include these to allow researchers to ascertain not only the range of factors that affect adherence but also the impact of each. |

*Engler*

| **Characteristics of included systematic review Engler et al. 2018** | | |
| --- | --- | --- |
| **Citation** | | |
| Engler K, Lènàrt A, Lessard D, Toupin I, Lebouché B. Barriers to antiretroviral therapy adherence in developed countries: a qualitative synthesis to develop a conceptual framework for a new patient-reported outcome measure. AIDS Care - Psychol Socio-Medical Asp AIDS/HIV. 2018;30(May):17–28. | | |
| **Search Summary Details** | | |
| **Literature Searched** | Peer reviewed databases: 3 databases: Medline, Psych INFO and Embase | |
| **Search Dates** | 1996 to 10 March 2016 | **Date of last search: 10 March 2016** |
| **Search Criteria** | | |
|  | **What the review authors searched for** | **What the review authors found:** |
| **Studies** | Searched for English or French peer-reviewed qualitative and mixed methods studies containing qualitative data. | 41 studies: 40 qualitative studies, and 1 systematic review |
|  |  | **Studies relevant to this overview:** |
|  |  | 40 studies: 40 qualitative studies |
| **Participants** | Adult HIV positive patients | N=1482 adult HIV positive participants (including Men, Women, MSM, IDU) |
| **Issue** | Adherence | Adherence (40) |
| **Setting** | Developed Countries | United States (n =35), Europe (n=3) (Switzerland, the Netherlands and Belgium) and Canada (n=2). |
| **Outcomes: Barriers and Facilitators** | Patient reported barriers to ART adherence | **Overview Framework:** Individual, Interpersonal, Community and Social, Political and Health System, and Structural Factors. |
| **Systematic Review Methods** | | |
| **Conceptual framework:** | No predetermined framework. Review aimed to develop an electronically administered patient-reported outcome measure (PRO) and then generate a conceptual framework. | |
| **Data extraction method:** | One author extracted all the data into a predesigned Excel spreadsheet that was double-checked for accuracy. | |
| **Appraisal tool used** | No appraisal was conducted | |
| **Data synthesis method:** | Thematic Synthesis | |

| **JBI Quality Appraisal** | | |
| --- | --- | --- |
| **Question** | **Judgement** | **Justification** |
| **1. Is the review questions clearly and explicitly stated?** | Yes | Based on findings derived from qualitative methods of studies conducted in developed countries with HIV positive adults, what factors are identified as barriers to taking ART as prescribed and how are they interrelated? |
| **2. Were the inclusion criteria appropriate for the review question?** | No | The sought articles published original qualitative studies from 1996 to 2016, written in English or French, reporting studies conducted with PLHIV in a developed country. Conference abstracts and studies only concerned with barriers to adherence in correctional environments or in youth under 18 years old were excluded. Because of the global, inclusion for countries the English inclusion criteria is not appropriate. |
| **3. Was the search strategy appropriate?** | Yes | Author was contacted for search strategy as paper suggests it is available upon request and the search strategies were appropriate. Provided evidence of key terms and search filters for search conducted from 1996 to 10 March 2016. The university librarian designed search queries. |
| **4. Were the sources and resources used to search for studies adequate?** | No | Review searched 3 electronic databases: Medline, Psych INFO and Embase. Downgraded as no grey literature was searched. |
| **5. Was selection of studies done adequately?** | No | Twenty percent of both the de-duplicated records and the records marked for full-text examination were evaluated by another co-author to test interrater reliability with Cohen’s Kappa. Not all included studies were checked in duplicate and independently. |
| **6. Were the criteria for appraising studies appropriate?** | No | No appraisal was conducted with reason |
| **7. Was the critical appraisal conducted by two or more reviewers independently?** | No | No appraisal was conducted with reason |
| **8. Were there methods to minimize errors in the data extraction?** | No | One author extracted all the data into a predesigned Excel spreadsheet that was double-checked for accuracy. This was not adequate for a 'Y'. It does not specifically say that another author checked it. |
| **9. Were the methods used to combine studies appropriate?** | Yes | Steps of the thematic analysis are operationalised in the paper and described using the Braun &Clarke (2006) method of synthesis. |
| **10. Were recommendations for policy and/or practice supported by the reported data?** | Yes | No single intervention can ensure the maintenance of high ART adherence. Given the patient diversity, the clinical management of ART adherence barriers should be tailored to the individual patient. Supported by the data. Detailed description for implications for practice. |
| **11. Were the specific directives for new research appropriate?** | Yes | Findings of the review supported the conceptual framework for the development of the Patient Reported Outcome (PRO) measure. |

*Ferguson*

| **Characteristics of included systematic review Ferguson et al. 2012** | | |
| --- | --- | --- |
| **Citation** | | |
| Ferguson, L. et al., 2012. Linking women who test HIV-positive in pregnancy-related services to long-term HIV care and treatment services: a systematic review. Trop Med Int Health, 17(5 PG-564-80), pp.564–580. Available at: NS  -. | | |
| **Search Summary Details** | | |
| **Literature Searched** | Medline, EMBASE, Global Health and the International Bibliography of the Social Sciences and in the reference lists of included articles. Experts were consulted and 1 thesis was included. | |
| **Search Dates** | 1st January 2000 - 31st December 2010 | **Date of last search:** Not specified |
| **Search Criteria and Outcomes** | | |
|  | **What the review authors searched for** | **What the review authors found** |
| **Studies** | Studies could be observational or descriptive. No publications were excluded on the basis of study design | 20 studies: 3 Qualitative studies, 12 Quantitative: 3 Cross-sectional, 5 Retrospective Cohort, 1 Prospective Cohort; 1 Mix Methods, 6 Programme Reviews/Evaluations/Policy Analysis/Commentaries and 1 Case Study |
|  |  | **Studies relevant to this overview:** |
|  |  | 7 studies: 3 Qualitative studies and 4 Quantitative studies |
| **Participants** | HIV positive women in pregnancy-related services | Pregnant women with HIV. Total reported N=819 reported for qualitative studies. Number not reported for qualitative studies. |
| **Issue** | Linkage | Linkage to ART (7) |
| **Setting** | Low or middle-income countries | LMIC: Kenya (1), South Africa (1), Tanzania (1), Zimbabwe (1), Malawi (2), Uganda (1) |
| **Outcomes: Barriers and Facilitators** | To quantify attrition between women testing HIV-positive in pregnancy-related services and accessing long-term HIV care and treatment services in low- or middle-income countries and to explore the reasons underlying client drop-out | **Overview Framework:** Individual, Interpersonal, Political and Health System, and Structural Factors. |
| **Systematic Review Methods** | | |
| **Conceptual framework:** | No framework reported. | |
| **Data extraction method:** | A single researcher reviewed eligible articles after consensus regarding which articles to include. | |
| **Appraisal tool used** | No appraisal reported | |
| **Data synthesis method:** | Thematic content analysis | |

| **JBI Quality Appraisal** | | |
| --- | --- | --- |
| **Question** | **Judgement** | **Justification** |
| **1. Is the review questions clearly and explicitly stated?** | Yes | This study aimed to quantify attrition along the pathway between women testing HIV-positive in pregnancy-related services and accessing long-term HIV care and treatment services in low- or middle -income countries (LMIC) and to explore the reasons underlying client dropout by synthesising current literature. Review question is clear. |
| **2. Were the inclusion criteria appropriate for the review question?** | Yes | Articles were included in the review if the studies were carried out in a LMIC and contained information specific to access to long-term HIV care and treatment services among women who test HIV-positive in the context of pregnancy. Studies could be observational or descriptive. No publications were excluded based on study design. Only studies meeting predefined quality were included. Inclusion criteria is appropriate. |
| **3. Was the search strategy appropriate?** | No | Search strategy for the literature search provided. Searched for articles published in English, Portuguese, French or Spanish between 1st January 2000 and 31st December 2010. Few key words contained. |
| **4. Were the sources and resources used to search for studies adequate?** | Yes | Medline; EMBASE; Global Health and the International Bibliography of the Social Sciences and in the reference lists of included articles. Experts in the field were consulted and 1 PhD thesis was included. |
| **5. Was selection of studies done adequately?** | Yes | Search and screening were done in duplicate. Ten percent of the titles and abstracts were screened in duplicate and an agreement calculated. Following this, one author continued independently. |
| **6. Were the criteria for appraising studies appropriate?** | Unclear | Appraisal not reported. |
| **7. Was the critical appraisal conducted by two or more reviewers independently?** | Unclear | Appraisal not reported. |
| **8. Were there methods to minimize errors in the data extraction?** | No | Eligible articles were reviewed by a single researcher after consensus regarding which articles to include. |
| **9. Were the methods used to combine studies appropriate?** | Unclear | Quantitative data was pooled and extrapolations made. Qualitative data synthesis method was not specified. |
| **10. Were recommendations for policy and/or practice supported by the reported data?** | Yes | Providing family focused care and integrating cluster of differentiation 4 (CD4 testing) and highly active antiretroviral therapy (HAART) provision into prevention of mother-to-child HIV transmission services appear promising for increasing women’s uptake of HIV-related services. Individual level factors that need to be addressed include financial constraints and fear of stigma. |
| **11. Were the specific directives for new research appropriate?** | Yes | Additional work is needed to better understand the effectiveness and sustainability of Health facility-level interventions to improve linkage between HIV testing in pregnancy-related services and long-term HIV care and treatment services. It is critical that the strengths and weakness of existing and new interventions be documented so that lessons learnt can be translated into concrete benefits in terms of access to HIV-related services for pregnant women who require them. |

*Flores*

| **Characteristics of included systematic review Flores et al. 2016** | | |
| --- | --- | --- |
| **Citation** | | |
| Flores D, Leblanc N, Barroso J. Enrolling an retaining patients with Human Immunodeficiency Virus (HIV) in their care: A Metasynthesis of qualitative studies. Int J Nurs Stud. 2016;62(2):126–36. | | |
| **Search Summary Details** | | |
| **Literature Searched** | Comprehensive automated searches of the literature found in three electronic databases (Cumulative Index to Nursing and Allied Health Literature [CINAHL], MEDLINE [PubMed] and PsycINFO). | |
| **Search Dates** | 2008 - 2013 | **Date of last search: Not specified** |
| **Search Criteria and Outcomes** | | |
|  | **What the review authors searched for** | **What the review authors found relevant to this overview** |
| **Studies** | Searched for qualitative studies that had data on linkage and retention | 69 qualitative studies |
|  |  | **Studies relevant to this overview:** |
|  |  | 69 qualitative studies |
| **Participants** | PLHIV | N=3257 people included in these studies; 2263 patients or HIV-positive participants from the community (740 men, 1008 women, 78 transgender individuals and 437 people with unspecified gender). 994 other people were included in the studies such as family members, friends, physicians, nurses, treatment advocates, caregivers, clinic staff, program directors, social workers and other key stakeholders. |
| **Issue** | Linkage and Retention | Linkage to ART (69) and retention in care (n=69) |
| **Setting** | No geographic restrictions used. | United States (22 reports) followed by South Africa (9), Uganda (6), Nigeria (4), Zimbabwe (4) and China (4); 20=unspecified |
| **Outcomes: Barriers and Facilitators** | Individual perspectives on barriers | **Overview Framework:** Individual, Interpersonal, Community and Social, Political and Health System, and Structural Factors. |
| **Systematic Review Methods** | | |
| **Conceptual framework:** | Theory of Triadic Influence | |
| **Data extraction method:** | Data extracted into EXCEL sheet and the headings described. No information about who conducted it. | |
| **Appraisal tool used** | No appraisal used with reason | |
| **Data synthesis method:** | Metasynthesis | |

| **JBI Quality Appraisal** | | |
| --- | --- | --- |
| **Question** | **Judgement** | **Justification** |
| **1. Is the review questions clearly and explicitly stated?** | Yes | The first aim of this report was to determine factors that influence linkage and retention in care with which people diagnosed with HIV infection must contend. The second aim was to determine what healthcare providers could do to enhance linkage and retention in care for people with HIV infection. |
| **2. Were the inclusion criteria appropriate for the review question?** | No | Qualitative research studies or the qualitative findings from mixed methods studies, b) published in English and in peer-reviewed journals, and c) published from 2008 to 2013. Did not specify eligibility for participants. Did not specify inclusion or exclusion criteria for the outcomes. |
| **3. Was the search strategy appropriate?** | Yes | Key terms including ‘Human Immunodeficiency Viruses, ‘Acquired Immune Deficiency Syndrome’, ‘qualitative’, and ‘themes’. The combination of Boolean terms (and, or, not) were also added. Evidence of CINAHL search strategy available and appears appropriate. |
| **4. Were the sources and resources used to search for studies adequate?** | No | Comprehensive automated searches of the literature found in three electronic databases (Cumulative Index to Nursing and Allied Health Literature [CINAHL], MEDLINE [PubMed] and PsycINFO). Grey literature, conference abstracts and literature reviews were excluded. |
| **5. Was selection of studies done adequately?** | Unclear | No information on study selection process. |
| **6. Were the criteria for appraising studies appropriate?** | No | No appraisal used with reason |
| **7. Was the critical appraisal conducted by two or more reviewers independently?** | No | No appraisal used with reason |
| **8. Were there methods to minimize errors in the data extraction?** | Unclear | Information about who conducted the data extraction not reported. The data was summarized into Excel spreadsheets using the Matrix method (Garrard, 2013). Column headings about pertinent study details (i.e., design, sample and findings) were used in these evidence tables, along with identifying which components of the Treatment Cascade they addressed (whether testing and notification of results, linkage and retention to HIV care, or antiretroviral initiation and viral suppression). Many articles had several unique findings applicable to multiple components of the HIV Treatment Cascade and therefore cited more than once. Data extraction was performed by DF and NL independently with discrepancies addressed and resolved during team discussions. |
| **9. Were the methods used to combine studies appropriate?** | Yes | Steps to meta-synthesis are outlined and detailed. Authors used the three steps of extraction, aggregation and synthesis. |
| **10. Were recommendations for policy and/or practice supported by the reported data?** | Yes | Authors provide system level recommendations that has implications for practice such logistical reinforcement of staff, structural support, multidisciplinary services, alternative care sites and the use of information technology. |
| **11. Were the specific directives for new research appropriate?** | Yes | Because multifactorial ecological factors synergistically affect individual-level outcomes, research on the macro-level impediments to system changes similar to the recommendations in this study must be a priority. Research into the experiences of providers and key stakeholders who undertake process improvement projects around HIV surveillance and care delivery can identify persistent macro-level linkage and retention barriers. |

*Gaston*

| **Characteristics of included systematic review Gaston et al. 2015** | | |
| --- | --- | --- |
| **Citation** | | |
| Gaston, G.B. & Alleyne-Green, B., 2013. The impact of African Americans’ beliefs about HIV medical care on treatment adherence: a systematic review and recommendations for interventions. AIDS Behav, 17(1 PG-31-40), pp.31–40. Available at: NS  -. | | |
| **Search Summary Details** | | |
| **Literature Searched** | Medline, PsychInfo, Academic search complete and PubMed. | |
| **Search Dates** | 1 January 2001-31 May 2012 | **Date of last search: Not specified** |
| **Search Criteria and Outcomes** | | |
|  | **What the review authors searched for** | **What the review authors found** |
| **Studies** | Both quantitative and qualitative published studies. | 16 Studies: Qualitative (6) and Quantitative (10) |
|  |  | **Studies relevant to this overview:** |
|  |  | 16 Studies: Qualitative (6) and Quantitative (10) |
| **Participants** | African Americans LHIV | African Americans LHIV Total n=2846 |
| **Issue** | Adherence | Adherence (16) |
| **Setting** | HIC USA | HIC: USA (16) |
| **Outcomes: Barriers and Facilitators** | African Americans patients perceptions of HIV medical care, specifically as it relates to adherence to medical self-care and ART medication regimens. | **Overview Framework**: Interpersonal, Community and Social, Political and Health System, |
| **Systematic Review Methods** | | |
| **Conceptual framework:** | No framework reported. | |
| **Data extraction method:** | Data extraction process not reported. | |
| **Appraisal tool used** | Appraisal not reported. | |
| **Data synthesis method:** | Thematic analysis | |

| **JBI Quality Appraisal** | | |
| --- | --- | --- |
| **Question** | **Judgement** | **Justification** |
| **1. Is the review questions clearly and explicitly stated?** | Yes | Explores African American patients' perceptions of HIV medical care, specifically as it relates to adherence to medical self-care and antiretroviral medication regimens. |
| **2. Were the inclusion criteria appropriate for the review question?** | Yes | Only include peer-reviewed journals published from January 1, 2001 through May 31, 2012. The review included both quantitative and qualitative studies that described the perceptions of HIV-related medical care and its relationship with self-care and anti-retroviral drug adherence among adult African American patients. Studies included in the review had to meet at least one of two search criteria: the perceptions of HIV medical care in relation to its impact on medical self-care and/or the perceptions of HIV medical care in relation to its impact on medical adherence to ART |
| **3. Was the search strategy appropriate?** | Unclear | Keywords have been indicated. Searches completed between January 1, 2001 to May 31, 2012. No full search strategy available. |
| **4. Were the sources and resources used to search for studies adequate?** | No | Medline, PsychInfo, Academic Search Complete and PubMed. No grey literature was searched. |
| **5. Was selection of studies done adequately?** | No | Studies were selected if they were published in peer review journals and focused of ART or medical self-care and recruited African American men. Study selection and screening was not reported to be done independently and in duplicate. |
| **6. Were the criteria for appraising studies appropriate?** | Unclear | Appraisal not reported. |
| **7. Was the critical appraisal conducted by two or more reviewers independently?** | Unclear | Appraisal not reported. |
| **8. Were there methods to minimize errors in the data extraction?** | Unclear | Data extraction process not described. |
| **9. Were the methods used to combine studies appropriate?** | Yes | The authors made use of a thematic analysis to identify three key themes in the 16 descriptive studies examined: (1) perceived racism and discrimination within health care settings, (2) conspiracy beliefs, and (3) perceived quality of health care provider relationships. |
| **10. Were recommendations for policy and/or practice supported by the reported data?** | Yes | Healthcare providers need to be aware of African Americans' historical relationship with the health care system. Health care providers should also engage African Americans in the health care process inclusive of using good communication, exploring patient beliefs about HIV care, and getting to know patients personally in order to understand what motivates them to adhere to HIV treatment regimens. Health care providers should use more open ended questions, less medical jargon and explore patient beliefs and concerns about their prescribed HIV treatment regimens. Appropriate recommendations. |
| **11. Were the specific directives for new research appropriate?** | Yes | Interventions to reduce discriminatory practices in provider judgment, behaviour, and decision-making should promote the cognitive strategy of individuation. Interventions aimed to examine patient experiences and narratives is critical. Directives are appropriate. |

*Geter*

| **Characteristics of included systematic review Geter et al. 2018** | | |
| --- | --- | --- |
| **Citation** | | |
| Geter A, Sutton MY, Hubbard McCree D. Social and structural determinants of HIV treatment and care among black women living with HIV infection: a systematic review: 2005–2016. AIDS Care [Internet]. 2018;30(4):409–16. Available from: https://www.tandfonline.com/doi/full/10.1080/09540121.2018.1426827 | | |
| **Search Summary Details** | | |
| **Literature Searched** | PubMed, PsycINFO, Scopus, Embase, Global Health, OVID/Medline and Google Scholar. | |
| **Search Dates** | January 2015 and December 2016 | **Date of last search: Not specified** |
| **Search Criteria and Outcomes** | | |
|  | **What the review authors searched for** | **What the review authors found relevant to this overview** |
| **Studies** | Published quantitative and qualitative research meeting the inclusion criteria. | 16 Studies: Qualitative studies (11 studies) and Quantitative (5 studies) |
|  |  | **Studies relevant to this overview:** |
|  |  | 14 studies: Qualitative studies (10 studies) and Quantitative Cross sectional studies (3 studies), Self-reported baseline in RCT (1) |
| **Participants** | Black women living with HIV | African American females living with HIV Total n=830 |
| **Issue** | Adherence and Retention in Care | Adherence (6 studies); Retention in Care (8 studies) |
| **Setting** | US | HIC: US (14 studies) |
| **Outcomes: Barriers and Facilitators** | Common themes for barriers to HIV Care | **Overview Framework:** Individual, Interpersonal, Community and Social, Political and Health System, and Structural Factors. |
| **Systematic Review Methods** | | |
| **Conceptual framework:** | No framework reported | |
| **Data extraction method:** | Authors do not specify who conducted the data extraction. Description of the types of data extracted is provided. | |
| **Appraisal tool used** | No appraisal reported. | |
| **Data synthesis method:** | Thematic content analysis | |

| **JBI Quality Appraisal** | | |
| --- | --- | --- |
| **Question** | **Judgement** | **Justification** |
| **1. Is the review questions clearly and explicitly stated?** | Yes | The purpose of this literature review is to identify possible social and structural factors related to the disparities in treatment and care among black women living with diagnosed HIV infection (BWLH). Review question clearly stated |
| **2. Were the inclusion criteria appropriate for the review question?** | Yes | Studies with population of >60% black and 100% female. Qualitative and quantitative studies. Specified outcomes to biomedical, structural, social and psychosocial determinants. |
| **3. Was the search strategy appropriate?** | Unclear | Search was conducted but no key terms and phrases, MESH terms and evidence of the search not reported. Details of the search not reported. |
| **4. Were the sources and resources used to search for studies adequate?** | No | PubMed, PsycINFO, Scopus, Embase, Global Health, OVID/Medline and Google Scholar. Did not search abstracts, unpublished dissertations, editorials, commentaries and studies that were conducted outside of the U.S. No grey literature included. |
| **5. Was selection of studies done adequately?** | Unclear | Study selection not reported |
| **6. Were the criteria for appraising studies appropriate?** | Unclear | Appraisal not reported |
| **7. Was the critical appraisal conducted by two or more reviewers independently?** | Unclear | Appraisal not reported |
| **8. Were there methods to minimize errors in the data extraction?** | Unclear | Authors do not specify who conducted the data extraction. Description of the types of data extracted is provided. |
| **9. Were the methods used to combine studies appropriate?** | Yes | Authors used content analysis to categorise and create themes and reported on the themes. |
| **10. Were recommendations for policy and/or practice supported by the reported data?** | Yes | Based on study findings with the specific sample of BWLHIV authors recommend the development of social and structural interventions that increase accessibility and acceptability to services. They also recommend that prevention evidence-based HIV interventions be developed specifically for BWLHIV. Supported by the data. |
| **11. Were the specific directives for new research appropriate?** | Yes | Future studies, with larger samples of BWLH, are needed to increase the number of culturally appropriate interventions and evidence-based practices for addressing social and structural factors that impede treatment and care for BWLH. Based on findings. |

*Govindasamy*

| **Characteristics of included systematic review Govindasamy et al. 2012** | | |
| --- | --- | --- |
| **Citation** | | |
| Govindasamy, D., Ford, N. & Kranzer, K., 2012. Risk factors, barriers and facilitators for linkage to antiretroviral therapy care: a systematic review. Aids, 26(16 PG-2059-67), pp.2059–2067. Available at: NS  -. | | |
| **Search Summary Details** | | |
| **Literature Searched** | Databases include: Medline, Global Health, Web Search on: Africa-Wide Information and Google Scholar and Conference abstracts from: Conference on HIV Pathogenesis and Treatment of the International AIDS Society, the Conference on etroviruses and Opportunistic Infections, the International AIDS Conference and the AIDS Educational Global Information System. Experts in the field were contacted for recommended literature. | |
| **Search Dates** | 01 January 2000-31 May 2011 | **Date of last search:** Not specified |
| **Search Criteria and Outcomes** | | |
|  | **What the review authors searched for** | **What the review authors found** |
| **Studies** | Quantitative and qualitative studies. Published in English. Conference abstracts. | 42 Studies: 6 Qualitative 17 Quantitative, 19 Mixed Methods |
|  |  | **Studies relevant to this overview:** |
|  |  | 21 Studies: 11 Qualitative, 7 Quantitative and 3 Mixed methods |
| **Participants** | PLHIV in sub-Saharan Africa | PLHIV in sub-Saharan Africa and health care workers. |
| **Issue** | Linkage | Linkage (n=21) |
| **Setting** | Studies in sub-Saharan Africa | South Africa (6); the remainder were conducted in Uganda (6), Kenya (2), Tanzania (2), Zambia (2), and 1 study each from Ethiopia, Swaziland, Mozambique, and South Africa and Zimbabwe. |
| **Outcomes: Barriers and Facilitators** | Risk factors and barriers and facilitators | **Overview Framework**: Individual, Interpersonal, Community and Social, Political and Health System, and Structural Factors. |
| **Systematic Review Methods** | | |
| **Conceptual framework:** | No framework reported | |
| **Data extraction method:** | Data extraction form was used but method and who conducted it not reported. | |
| **Appraisal tool used** | The quality assessment form was adapted from the STROBE statement checklist. | |
| **Data synthesis method:** | Thematic content analysis | |

| **JBI Quality Appraisal** | | |
| --- | --- | --- |
| **Question** | **Judgement** | **Justification** |
| **1. Is the review questions clearly and explicitly stated?** | Yes | This review summarises the characteristics of patient and programme level factors associated with retention in care during the pre-ART period and linkage to ART care in sub-Saharan Africa |
| **2. Were the inclusion criteria appropriate for the review question?** | Yes | The search was limited to quantitative and qualitative studies conducted within sub-Saharan Africa, published in English between 01 January 2000 (the time when ART first became available in this region) and 31 May 2011 and within conference abstracts. |
| **3. Was the search strategy appropriate?** | No | Search strategy available in supplementary material. Sufficient use of keywords and terms. Only searched for English studies. |
| **4. Were the sources and resources used to search for studies adequate?** | Yes | Databases include: Medline, Global Health, Web Search on: Africa-Wide Information and Google Scholar and Conference abstracts from: Conference on HIV Pathogenesis and Treatment of the International AIDS Society, the Conference on etroviruses and Opportunistic Infections, the International AIDS Conference and the AIDS Educational Global Information System. Experts in the field were contacted for recommended literature. |
| **5. Was selection of studies done adequately?** | Yes | Screening of articles taken through to full review was performed in duplicate. |
| **6. Were the criteria for appraising studies appropriate?** | No | The quality assessment form was adapted from the STROBE statement checklist. This tool is not adequate. |
| **7. Was the critical appraisal conducted by two or more reviewers independently?** | No | Process of critical appraisal not described. |
| **8. Were there methods to minimize errors in the data extraction?** | Unclear | Data extraction process not described. Data extraction form adapted from STROBE tool. No description of what was extracted or who conducted it. |
| **9. Were the methods used to combine studies appropriate?** | Unclear | Not specified. Table of included studies does not correlate with referencing system. Web appendixes available do not correlate with included studies available in Table 1. Author responded and said to add the studies in Table 1 and Web Appendix together to get to 42 included studies but this amounts to over 50 studies. Synthesis of findings in text do not relate to the Tables provided in text. |
| **10. Were recommendations for policy and/or practice supported by the reported data?** | Unclear | The results of this review indicate the need for task-shifting, decentralisation of care and integration of services to alleviate current health system barriers. Urgent interventions such as support groups and intense post-test counselling are warranted. Uncertain about the findings that were synthesised and how these support the recommendations for practice made. |
| **11. Were the specific directives for new research appropriate?** | Yes | Direction for piloting of interventions that could improve linkage to HIV and ART care. Future studies should examine interventions that could potentially reduce out-of-pocket payment for transport and minimise the travel to clinics for patients. |

*Heestermans*

| **Characteristics of included systematic review Heestermans et al. 2016** | | |
| --- | --- | --- |
| **Citation** | | |
| Heestermans T, Browne JL, Aitken SC, Vervoort SC, Klipstein-Grobusch K. Determinants of adherence to antiretroviral therapy among HIV-positive adults in sub-Saharan Africa: a systematic review. BMJ Glob Heal [Internet]. 2016;1(4):e000125. Available from: http://gh.bmj.com/lookup/doi/10.1136/bmjgh-2016-000125 | | |
| **Search Summary Details** | | |
| **Literature Searched** | 6 databases: PubMed, Cochrane Library, EMBASE, Web of Science, Popline and Global Health Library | |
| **Search Dates** | January 2002 to 27 October 2014. | **Date of last search: None specified** |
| **Search Criteria and Outcomes** | | |
|  | **What the review authors searched for** | **What the review authors found relevant to this overview** |
| **Studies** | Qualitative, quantitative or mixed methods study designs and languages included. | 146 studies: Qualitative (37), Quantitative (112) and Mixed methods (3) |
|  |  | **Studies relevant to this overview:** |
|  |  | 146 studies: Qualitative (37), Quantitative (112) and Mixed methods (3) |
| **Participants** | Adult PLHIV older than 15 years and receiving ART | 161 922 Adult PLHIV |
| **Issue** | Adherence | Adherence (146) |
| **Setting** | Sub Saharan Africa | Sub-Saharan Africa |
| **Outcome: Barriers and Facilitators** | Determinants of Adherence | **Overview Framework:** Individual, Interpersonal, Community and Social, Political and Health System, and Structural Factors. |
| **Systematic Review Methods** | | |
| **Conceptual framework** | None reported. | |
| **Data extraction method** | Used a standardised extraction form and carried out by a single author with a second author available if clarification was needed. | |
| **Appraisal tool used** | For qualitative studies used tool by Vervoort et al. In addition, an adapted version of the Cochrane Collaboration tool for quantitative studies. Mixed methods studies were scored by both measures. | |
| **Data synthesis method** | Narrative descriptions of qualitative data. | |

| **JBI Quality Appraisal** | | |
| --- | --- | --- |
| **Question** | **Judgement** | **Justification** |
| **1. Is the review questions clearly and explicitly stated?** | Yes | The aim of this review was to identify and summarise determinants of adherence to ART among HIV-positive adults. Clear aim identified. |
| **2. Were the inclusion criteria appropriate for the review question?** | Unclear | The authors say that all languages are eligible when searching and then state that in selection studies not in English or Dutch were excluded. |
| **3. Was the search strategy appropriate?** | Yes | Search terms composed of MeSH and combined text for domain. The terms and search strategy are appropriate and provided in the supplementary material. |
| **4. Were the sources and resources used to search for studies adequate?** | No | PubMed/ MEDLINE, The Cochrane Library, EMBASE, Web of Science, POPLINE, and the Global Health Library for all publications up to 27 October 2014. No grey literature searched. |
| **5. Was selection of studies done adequately?** | Unclear | Selection was done independently and in duplicate. However, authors only report this for titles and abstracts and do not report on the screening of full texts. |
| **6. Were the criteria for appraising studies appropriate?** | Yes | For qualitative studies used tool by Vervoort et al. And an adapted version of the Cochrane Collaboration tool for quantitative studies. Mixed methods studies were scored by both measures. Measures are appropriate. |
| **7. Was the critical appraisal conducted by two or more reviewers independently?** | Unclear | Not reported. |
| **8. Were there methods to minimize errors in the data extraction?** | No | Used a standardised extraction form and carried out by a single author with a second author available if clarification was needed. 10% of qualitative studies extraction was conducted by another second reviewer. Rules regarding reporting of duplicate studies. Downgraded to no as extraction was done by a single author. |
| **9. Were the methods used to combine studies appropriate?** | Unclear | Meta-analysis and pooled estimates using odds ratio for quantitative data. Qualitative data was described narratively. No further description is provided. |
| **10. Were recommendations for policy and/or practice supported by the reported data?** | Yes | The recommendations are supported by the reported data. |
| **11. Were the specific directives for new research appropriate?** | Unclear | Not reported. |

*Hodgson*

| **Characteristics of included systematic review Hodgson et al. 2014** | | |
| --- | --- | --- |
| **Citation** | | |
| Hodgson, I. et al., 2014. A systematic review of individual and contextual factors affecting ART initiation, adherence, and retention for HIV-infected pregnant and postpartum women. PLoS One, 9(11 PG-e111421), p.e111421. Available at: NS  -. | | |
| **Search Summary Details** | | |
| **Literature Searched** | PubMed and SSCI (also grey literature through conference databases, multilateral and bilateral agency websites, NGO's) | |
| **Search Dates** | 1st January 2008 - 26 March 2013 | **Date of last search:** Not specified |
| **Search Criteria and Outcomes** | | |
|  | **What the review authors searched for** | **What the review authors found** |
| **Studies** | Any study that reported empirical qualitative or quantitative findings relevant to the review question. English language. | 34 studies included in the review: Qualitative (12), Quantitative (16) and Mixed methods (6), |
|  |  | **Studies relevant to this overview:** |
|  |  | 34 studies included in the review: Qualitative (12), Quantitative (16) and Mixed methods (6), |
| **Participants** | Pregnant women and postpartum women infected with HIV | Pregnant women and postpartum women infected with HIV |
| **Issue** | Initiation, adherence and retention | Linkage to ART (14) and Adherence (24) |
| **Setting** | LMIC and HIC | Ghana (1), Nigeria (1), Malawi (5), South Africa (6), Zimbabwe (2), Tanzania (2), Kenya (5), Uganda (3), Brazil (1), Australia (1), USA (3), Rawanda (1), France (1), Zambia (1), Latin America (1) |
| **Outcomes: Barriers and Facilitators** | Individual and contextual factors | **Overview Framework:** Individual, Interpersonal, Community and Social, Political and Health System, and Structural Factors. |
| **Systematic Review Methods** | | |
| **Conceptual framework** | WHO health systems framework and the Supporting the Use of Research Framework (SURE) | |
| **Data extraction method** | Independently and in duplicate | |
| **Appraisal tool used** | Appraisal and quality assessment was conducted but the tools and who conducted the appraisal are unclear and not reported. | |
| **Data synthesis method** | Thematic Analysis | |

| **JBI Quality Appraisal** | | |
| --- | --- | --- |
| **Question** | **Judgement** | **Justification** |
| **1. Is the review questions clearly and explicitly stated?** | Yes | What are the individual and contextual risk factors affecting the initiation, adherence, and retention to ART among HIV-infected pregnant women during and following pregnancy? |
| **2. Were the inclusion criteria appropriate for the review question?** | Yes | Studies from low and middle-income countries (LMICs), as well as high-income countries, were included, as were studies conducted in community or health system settings. Included studies that described health systems-related factors if these were described health systems-related factors if these were described from the women's perspectives or experiences |
| **3. Was the search strategy appropriate?** | No | Adequate search strategy available in supporting information and keywords available in text. Did not include grey literature and only included English studies. |
| **4. Were the sources and resources used to search for studies adequate?** | Yes | Both peer-reviewed journal articles were searched systematically in the PubMed and Social Sciences Citation Index. English language only studies. Key words were indicated. Grey literature was searched through conference abstract databases, multilateral and bilateral agency websites, and websites of non-governmental organisations. Studies published between January 1, 2008 and March 26, 2013 |
| **5. Was selection of studies done adequately?** | Yes | Studies were selected in two stages: First three review authors independently assessed the first 100 abstracts from PubMed and then each reviewers list compared, and discrepancies discussed and resolved. From this, the inclusion and exclusion criteria were refined and clarified. Second, one review author reviewed the remaining studies to include or exclude. |
| **6. Were the criteria for appraising studies appropriate?** | Unclear | Ranked each included study as low, medium or high with respect to overall risk of bias, based on its sample size, selection criteria, sampling procedure and data analysis method. Appraisal tool used was not reported. |
| **7. Was the critical appraisal conducted by two or more reviewers independently?** | Unclear | Not reported |
| **8. Were there methods to minimize errors in the data extraction?** | Yes | Once the study selection process was concluded, one review author extracted data from the studies using a standard template. Initial data extraction captured both the study characteristics as well as key findings related to factors associated with initiation, adherence, and retention of ART. A second author also reviewed the studies and extracted data relating to key individual and contextual barriers and enablers associated with initiation, adherence, and retention. Extracted findings from both authors were compared and discrepancies resolved. |
| **9. Were the methods used to combine studies appropriate?** | Yes | Study findings were analysed thematically. The barriers and enablers identified were arranged thematically within a framework of individual, interpersonal, community, and structural categories. These categories were further divided into enabling factors and barriers to ART adherence, e.g., knowledge about HIV or ART or wanting to protect one’s child (individual-level enablers); domestic violence or spousal dependence (interpersonal-level barriers); stigma (community-level barrier); or health worker attitude or support group participation (structural-level enablers). This method was adequate. |
| **10. Were recommendations for policy and/or practice supported by the reported data?** | Yes | Recommendations supported by data and findings. |
| **11. Were the specific directives for new research appropriate?** | Yes | Identified evidence gaps and methodological gaps based on the data and synthesis. |

*Katz*

| **Characteristics of included systematic review Katz et al. 2013** | | |
| --- | --- | --- |
| **Citation** | | |
| Katz IT, Ryu AE, Onuegbu AG, Psaros C, Weiser SD, Bangsberg DR, et al. Impact of HIV-related stigma on treatment adherence: systematic review and meta-synthesis. J Int AIDS Soc. 2013;16(3 Suppl 2). | | |
| **Search Summary Details** | | |
| **Literature Searched** | Published and grey literature in BIOSIS previews, CINAHL, Embase, ERIC, Medline, ProQuest Dissertations and Theses, PsychInfo, Web of Science and the WHO African Index Medicus. Grey literature searched. | |
| **Search Dates** | Up until February 2013 | **Date of last search:** February 2013 for PubMed and 2011 for all other databases |
| **Search Criteria and Outcomes** | | |
|  | **What the review authors searched for** | **What the review authors found** |
| **Studies** | Published and grey literature. Qualitative and quantitative. No languages excluded | 75 Studies: 41 Quantitative, 34 Qualitative (published between 1999-2013) |
|  |  | **Studies relevant to this overview:** |
|  |  | 34 Qualitative studies |
| **Participants** | PLHIV | PLHIV between 18-30 years old, providers of HIV care, single persons and those in intimate partnerships and persons with and without children. High risk groups including men who have sex with men, injection drug users and commercial sex workers. |
| **Issue** | The relationship between HIV-related stigma and ART adherence | Adherence (34) |
| **Setting** | LMIC and HIC | Uganda (9), South Africa (5), India (2), and 1 study each from DRC, US, Brazil, Botswana, Tanzania, Thailand, Egypt, Ethiopia, Vietnam, Nepal, Nigeria, Asia, Zambia and China. Four countries were not reported. |
| **Outcomes: Barriers and Facilitators** | Stigma, disclosure and ART adherence | **Overview Framework:** Individual, Interpersonal, Community and Social, Political and Health System, and Structural Factors. |
| **Systematic Review Methods** | | |
| **Conceptual Framework:** | Not specified but methods of synthesis reported resembles grounded theory to generate new conceptual model. | |
| **Data extraction method** | Not reported | |
| **Appraisal tool used** | CASP Tool | |
| **Data synthesis method** | Meta-ethnography | |

| **JBI Quality Appraisal** | | |
| --- | --- | --- |
| **Question** | **Judgement** | **Justification** |
| **1. Is the review questions clearly and explicitly stated?** | No | We undertook this review to systematically assess the relationship between HIV-related stigma and ART adherence |
| **2. Were the inclusion criteria appropriate for the review question?** | No | Inclusion criteria not explicitly stated. |
| **3. Was the search strategy appropriate?** | Unclear | Authors report that the search strategy is available in the supplementary material and we followed the links. No material could be found. Author has been contacted and we are waiting for a response. |
| **4. Were the sources and resources used to search for studies adequate?** | Yes | Published and grey literature in BIOSIS Previews, CINAHL, EMBASE, ERIC, MEDLINE, ProQuest Dissertations and Theses, PsycINFO, Web of Science, and the WHO African Index Medicus. Searches were done in May 2011. ProQuest search completed in June 2011. In February 2013 one author updated Medline search. Consulted experts in the field. |
| **5. Was selection of studies done adequately?** | Unclear | Removed duplicates. Screened studies that appeared to be potentially related to ART adherence. Not specified whether this was done in duplicate. |
| **6. Were the criteria for appraising studies appropriate?** | Yes | Critical appraisal skills programme quality assessment tool for qualitative studies was adapted into conceptual domains being clearly described and these include the role of the researcher, sampling method, data collection and method of analysis. Quantitative studies were assessed through the domains of sample probability, validated self-report scale to measure stigma, validated self-report scale to measure ART adherence, statistical analysis accounts for missing numbers, study adjusts for confounding in analysis, and computing interests declared. |
| **7. Was the critical appraisal conducted by two or more reviewers independently?** | Unclear | They do not report whether one or more than one person assessed risk of bias |
| **8. Were there methods to minimize errors in the data extraction?** | Unclear | No evidence provided of tools used to minimise error in data extraction |
| **9. Were the methods used to combine studies appropriate?** | Yes | Iterative process of Meta synthesis (also described as meta-ethnography) to identify themes. This method was considered appropriate. |
| **10. Were recommendations for policy and/or practice supported by the reported data?** | Yes | Interventions aimed at enhancing social support. Structural interventions that strengthen the livelihoods of HIV-positive persons may also be a promising avenue for subverting HIV-related stigma. Results support the recommendation. |
| **11. Were the specific directives for new research appropriate?** | Yes | More research is needed to understand the conditions under which HIV-related outcomes are better than expected despite the experiences of HIV- and stigma-related adversity (which can be thought of as being related to the concept of resilience). Results support the recommendation. |

*Knettel*

| **Characteristics of included systematic review Knettel et al. 2018** | | |
| --- | --- | --- |
| **Citation** | | |
| Knettel BA, Cichowitz C, Ngocho JS, Knippler ET, Chumba LN, Mmbaga BT, et al. Retention in HIV Care During Pregnancy and the Postpartum Period in the Option B+ Era. JAIDS J Acquir Immune Defic Syndr [Internet]. 2017;77(5):1. Available from: http://insights.ovid.com/crossref?an=00126334-900000000-96776 | | |
| **Search Summary Details** | | |
| **Literature Searched** | Used 3 databases: PubMed, EMBASE, and the African Index Medicus (AIM). Screened abstracts from the nternational AIDS Society Conference and the Conference on Retroviruses and Opportunistic Infection during the study period of 2012– 2017. | |
| **Search Dates** | January 2012 to June 2017 | **Date of last search: June 15, 2017** |
| **Search Criteria and Outcomes** | | |
|  | **What the review authors searched for** | **What the review authors found relevant to this overview** |
| **Studies** | Qualitative and quantitative studies reported self-reported data. Studies published after January 2012 | 35 Studies: 13 Qualitative and 22 Quantitative |
|  |  | **Studies relevant to this overview:** |
|  |  | 13 Studies: 13 Qualitative |
| **Participants** | Pregnant and post-partum women on option B+. Special key populations were excluded (IDU, prisoners, sex workers and cohorts with low CD4 counts) | 736 Pregnant and post-partum women on option B+. |
| **Issue** | Linkage and Retention in Care. | Retention in Care (13) |
| **Setting** | Africa | Malawi (13 studies), Uganda (4), Zimbabwe (3), Mozambique (2), and 1 each from Cameroon, Ethiopia, Rwanda, South Africa, and Tanzania |
| **Outcome: Barriers and Facilitators** | Factors associated with retention in care | **Overview Framework:** Individual, Interpersonal, Community and Social, Political and Health System, and Structural Factors. |
| **Systematic Review Methods** | | |
| **Conceptual framework** | Not reported | |
| **Data extraction method** | Independently and in duplicate using pre-specified form. | |
| **Appraisal tool used** | None reported. | |
| **Data synthesis method** | Qualitative data was analysed using thematic analysis. | |

| **JBI Quality Appraisal** | | |
| --- | --- | --- |
| **Question** | **Judgement** | **Justification** |
| **1. Is the review questions clearly and explicitly stated?** | Yes | The goal of this systematic review was to identify and summarize the existing data on initiation and retention in care after starting lifelong ART (option B+) for pregnant and postpartum women in Africa. Aim and objectives are provided. |
| **2. Were the inclusion criteria appropriate for the review question?** | No | Studies were included if they reported patient-level data on retention in care among pregnant or postpartum women who received care under the clinical conditions of option B+ (i.e., lifetime initiation of ART during pregnancy or breastfeeding) in an African country. Studies using modelling estimates as opposed to actual patient data were excluded. For aim 2, studies were included if they explored factors associated with retention under the clinical conditions of option B+, including both quantitative and qualitative study designs. Special populations were excluded without reason. |
| **3. Was the search strategy appropriate?** | Yes | Designed in consultation with information specialist from Duke University Library. Used standardized search terms and key words related to the constructs of (1) HIV or AIDS, (2) option B+, universal “test and treat,” or lifelong ART, (3) pregnancy or the postpartum period, and (4) Africa orany African nation. When available, controlled vocabulary was used to capture broader categories related to the search terms, indexed by the databases (e.g., PubMed Medical Subject Headings, or MeSH). The specific search terms used for the PubMed database are detailed in supplementary material. |
| **4. Were the sources and resources used to search for studies adequate?** | No | Used 3 databases: PubMed, EMBASE, and the African Index Medicus (AIM). Screened abstracts from the international AIDS Society Conference and the Conference on Retroviruses and Opportunistic Infection during the study period of 2012– 2017. Conference abstracts related to retention in option B+ were compared with results from the database searches to ensure that relevant published articles derived from the abstracts had been captured. Did not search grey literature. |
| **5. Was selection of studies done adequately?** | Yes | Title and abstract and full text screened independently by two authors using Covidence.Disagreements resolved by discussion with a third author. |
| **6. Were the criteria for appraising studies appropriate?** | Unclear | Not reported |
| **7. Was the critical appraisal conducted by two or more reviewers independently?** | Unclear | Not reported |
| **8. Were there methods to minimize errors in the data extraction?** | Yes | Data extraction was performed using a standardized form and was performed independently and in duplicate by two authors. List of information extracted available in manuscript. |
| **9. Were the methods used to combine studies appropriate?** | Yes | Abstracted variables that were found to significantly predict retention or loss to follow-up in HIV care. For qualitative results, we abstracted themes related to risk or protective factors for retention in care. Using the abstracted results of all studies that examined factors associated with retention, we used thematic analysis to synthesize the data and identify common themes in the findings. Presented a narrative review of both the quantitative and qualitative data. Adequate. |
| **10. Were recommendations for policy and/or practice supported by the reported data?** | Yes | Authors describe the findings and recommendations disaggregated by study design. These are considered appropriate. |
| **11. Were the specific directives for new research appropriate?** | Yes | Practical directives for research are clearly and directly provided. |

*Lancaster*

| **Characteristics of included systematic review Lancaster et al 2016** | | |
| --- | --- | --- |
| **Citation** | | |
| Lancaster KE, Cernigliaro D, Zulliger R, Fleming PF, Hill C, Carolina N, et al. living with HIV in sub-Saharan Africa : A systematic review. 2017;15(4):377–86. | | |
| **Search Summary Details** | | |
| **Literature Searched** | PubMed, Embase, Web of Science, SCOPUS, CINAHL, Global Health, Psycinfo, Sociological Abstracts, and Popline | |
| **Search Dates** | Up to 22 November 2013 and a second search up to 30 July 2015 | **Date of last search: 30 July 2015** |
| **Search Criteria and Outcomes** | | |
|  | **What the review authors searched for** | **What the review authors found relevant to this overview** |
| **Studies** | Original and empirical qualitative and quantitative data collected after 2000 and published in English. | 10 studies: Qualitative (3), Quantitative (6) and Mixed Methods (3) |
|  |  | **Studies relevant to this overview:** |
|  |  | 10 studies: Qualitative (3), Quantitative (6) and Mixed Methods (3) |
| **Participants** | Female sex workers living with HIV | N=2721 Female sex workers living with HIV |
| **Issue** | Linkage to and retention in HIV Care Pre-ART, treatment initiation, ART adherence and viral suppression. | Linkage to ART (6) and Adherence (6) from 10 studies |
| **Setting** | Sub Saharan Africa | Rwanda (n = 1), Zimbabwe (n = 2), Benin (n = 2), Burkina Faso (n = 1), Nigeria (n = 1), Swaziland (n = 1), Kenya (n = 1), and Uganda (n = 1). |
| **Outcome: Barriers and Facilitators** | Care experiences and factors associated. | **Overview Framework:** Individual, interpersonal and structural factors |
| **Systematic Review Methods** | | |
| **Conceptual framework** | Conceptual model was developed as part of this review. | |
| **Data extraction method** | Used a standardised data abstraction sheet. Two reviewers independently and in duplicate extracted data. | |
| **Appraisal tool used** | Not reported. | |
| **Data synthesis method** | Thematic analysis | |

| **JBI Quality Appraisal** | | |
| --- | --- | --- |
| **Question** | **Judgement** | **Justification** |
| **1. Is the review questions clearly and explicitly stated?** | Yes | To examine and synthesise the findings in the quantitative and qualitative literature regarding the care experiences and factors associated with linkage to and retention in HIV care, treatment initiation, and ART adherence and viral suppression among FSW living with HIV in sub-Saharan Africa. |
| **2. Were the inclusion criteria appropriate for the review question?** | Yes | The inclusion criteria is described in detail and appropriate. Studies had to include female sex workers living with HIV, include original empirical quantitative or qualitative data, have data collected after 2000 and be published in English. Had to address determinants related to linkage, adherence or retention in sub-Saharan Africa. |
| **3. Was the search strategy appropriate?** | Unclear | Key terms provided. Search strategy for at least one database not reported. |
| **4. Were the sources and resources used to search for studies adequate?** | No | PubMed, Embase, Web of Science, SCOPUS, CINAHL, Global Health, Psycinfo, Sociological Abstracts, and Popline. Search was conducted on 22 November 2013 and updated on 30 July 2015. Did not include grey literature. |
| **5. Was selection of studies done adequately?** | Yes | Each review step included two independent reviewers who evaluated whether or not the article should be included, based on the following a priori inclusion criteria. Discrepancies were discussed with all four authors until a decision was reached. |
| **6. Were the criteria for appraising studies appropriate?** | Unclear | None reported |
| **7. Was the critical appraisal conducted by two or more reviewers independently?** | Unclear | None reported |
| **8. Were there methods to minimize errors in the data extraction?** | Yes | Used a standardised data abstraction sheet. Two reviewers independently and in duplicate extracted data. |
| **9. Were the methods used to combine studies appropriate?** | Unclear | First organised results by the care and treatment steps of the HIV care cascade: linkage and retention to care, ART initiation, and ART adherence and viral suppression. Given the focus on the care experiences and determinants of care among FSW living with HIV, the HIV testing step of the HIV care cascade was not included as part of our results. Also developed a conceptual framework to organise results using a multilevel framework that includes individual, interpersonal, and structural levels. Unclear as method of synthesis or more detail to process reported. |
| **10. Were recommendations for policy and/or practice supported by the reported data?** | Yes | The recommendations of integrating substance use treatment with HIV care and treatment programmes is supported by the data. The review highlights stigma and discrimination as prominent themes in the data and recommends interventions to reduce these such as sex work sensitization training. |
| **11. Were the specific directives for new research appropriate?** | Yes | The review was able to identify gaps in evidence on FSW and engagement in the cascade of care. |

*Lankowski*

| **Characteristics of included systematic review Lankowski et al. 2014** | | |
| --- | --- | --- |
| **Citation** | | |
| Lankowski AJ, Siedner MJ, Bangsberg DR, Tsai AC. Impact of geographic and transportation-related barriers on HIV outcomes in sub-Saharan Africa: A systematic review. AIDS Behav. 2014;18(7):1199–223. | | |
| **Search Summary Details** | | |
| **Literature Searched** | PubMed and Web of Science. Searched all abstracts from the International Conference on HIV treatment and Prevention Adherence of the International Association of Physicians in AIDS care. Included a limited number of manuscripts and abstracts recommended by experts in the field but not identified in the systematic search. PubMed and Google Scholar was searched again to identify IAPAC abstracts that had been subsequently published as full-length manuscripts. | |
| **Search Dates** | Databases up until August 2011 and abstracts from 2002-2004 and from 2006-2011. | **Date of last search:** Not specified. |
| **Search Criteria and Outcomes** | | |
|  | **What the review authors searched for** | **What the review authors found** |
| **Studies** | Descriptive, quantitative and qualitative studies, no language exclusion | 66 studies: Quantitative (29) Quantitative Descriptive (17) Qualitative (15) and Mixed Methods (5) |
|  |  | **Studies relevant to this overview:** |
|  |  | 34 studies included: Qualitative (16) Quantitative descriptive (18) |
| **Participants** | Either predominantly HIV-infected or prescribed ART for other reasons | N=69 506 Adults and children LHIV, HIV infected HCW, HC Providers, HIV infected rape victims, pregnant and postpartum women with HIV. |
| **Issue** | Voluntary counselling and testing, pre-ART linkage, loss to follow up/mortality and ART adherence or viral suppression. | Linkage (6), Adherence (23) and Retention (5) |
| **Setting** | Sub-Saharan Africa | Uganda (7), Malawi (4), Nigeria (3), Kenya (3), Zambia (2), Corte d'Ivoire (1), Botswana (3), Tanzania (2), Togo (1), Ethiopia (1), South Africa (2), The Gambia (1), Namibia (1), Zimbabwe (1), Uganda, Tanzania and Nigeria (1), Uganda, Tanzania and Botswana (1). |
| **Outcomes: Barriers and Facilitators** | Geographic and Transportation-related barriers | **Overview Framework:** Structural Factors |
| **Systematic Review Methods** | | |
| **Conceptual framework** | No framework reported | |
| **Data extraction method** | Used a standardised data extraction tool. Was conducted by a single author | |
| **Appraisal tool used** | No appraisal was conducted for qualitative studies. Only studies reporting a statistical association for transportation and geographic related barriers were assessed using a designed assessment tool. | |
| **Data synthesis method** | Odds ratio was calculated for quantitative studies. No data analysis method described for qualitative studies. | |

| **JBI Quality Appraisal** | | |
| --- | --- | --- |
| **Question** | **Judgement** | **Justification** |
| **1. Is the review questions clearly and explicitly stated?** | Yes | This review aimed to systematically assess the extent to which—and in what manner—geographic and transportation-related barriers affect HIV outcomes in SSA. |
| **2. Were the inclusion criteria appropriate for the review question?** | Yes | Studies that examined associations between geographic or transportation-related barriers and HIV outcomes in SSA: Voluntary counselling and testing, pre-ART linkage, loss to follow up/mortality and ART adherence or viral suppression. |
| **3. Was the search strategy appropriate?** | No | Full search strategy with search terms available as an appendix. Search terms for Africa setting not included. Search strategy is not comprehensive. |
| **4. Were the sources and resources used to search for studies adequate?** | No | PubMed and Web of Science. Searched all abstracts from the International Conference on HIV treatment and Prevention Adherence of the International Association of Physicians in AIDS care. Included a limited number of manuscripts and abstracts recommended by experts in the field but not identified in the systematic search. PubMed and Google Scholar was searched again to identify IAPAC abstracts that had been subsequently published as full-length manuscripts. There was no inclusion of databases relevant to Africa. Judged as N as authors should have included all manuscripts and abstracts recommended by experts in the field. |
| **5. Was selection of studies done adequately?** | No | Two reviewers screened the first 150 studies independently and in duplicate, agreement on the selection of studies for full text review reported as k=0.74, thereafter a single investigator completed the remainder of the screening. Full text screening was not conducted in duplicate. |
| **6. Were the criteria for appraising studies appropriate?** | No | For studies reporting a statistical association between a geographic or transportation-related barrier and an HIV outcome, we designed an assessment tool that accounted for seven parameters within the following four domains: (1) study design and population, (2) exposure measurement, (3) outcome measurement, and (4) data analysis. No appraisal conducted for qualitative studies. |
| **7. Was the critical appraisal conducted by two or more reviewers independently?** | Unclear | Not reported |
| **8. Were there methods to minimize errors in the data extraction?** | No | Using a standardized extraction form, data from all eligible studies were extracted by a single investigator. This should have been done in duplicate. |
| **9. Were the methods used to combine studies appropriate?** | No | The identification of transport and distance as barriers and the reporting thereof is available in the review for qualitative and descriptive studies. There was not a synthesis of findings or reporting of how qualitative data was synthesised. |
| **10. Were recommendations for policy and/or practice supported by the reported data?** | Yes | We found that geographic and transportation-related barriers impede access to care at all points in the HIV care continuum. this systematic review has important implications for HIV policy and programming in SSA. Policy-makers are urged to aggressively pursue service decentralization, and to prioritize investment in the necessary rural health care infrastructure. The recomendations were supported by the data. |
| **11. Were the specific directives for new research appropriate?** | Unclear | No research recommendations were made. |

*Lazuardi*

| **Characteristics of included systematic review Lazuardi et al. 2018** | | |
| --- | --- | --- |
| **Citation** | | |
| Lazuardi E, Bell S, Newman CE. A “scoping review” of qualitative literature about engagement with HIV care in Indonesia. Sex Health. 2018; | | |
| **Search Summary Details** | | |
| **Literature Searched** | Scopus, Medline, ProQuest and Web of Science was searched. Further searches were conducted using reference lists of relevant papers, the UNSW Sydney library and Google Scholar between July and December 2016. An additional search of a journal called Indonesia was performed using the same search terms used to search electronic databases. | |
| **Search Dates** | 1990 - 2016 | **Date of last search: 22 July 2016** |
| **Search Criteria and Outcomes** | | |
|  | **What the review authors searched for** | **What the review authors found relevant to this overview** |
| **Studies** | Qualitative studies | 17 Studies: Qualitative (17): 13 peer-reviewed articles and 4 book chapters |
|  |  | **Studies relevant to this overview:** |
|  |  | 11 Qualitative studies |
| **Participants** | PLHIV | PLHIV: including injecting drug users, pregnant women, MSM, transgendered people, women, men and sero-discordant couples. Also found information related to service providers, community members, TB patients, caregivers and community organisers. |
| **Issue** | Engagement in HIV Care: Linkage to care and Treatment Uptake and Adherence. | Linkage (2) and Adherence (2) Retention in Care (10) in 11 studies. |
| **Setting** | Indonesia | Indonesia (11) |
| **Outcome: Barriers and Facilitators** | Factors influencing engagement in the HIV cascade of care. | **Overview Framework:** Individual, Interpersonal, Community and Social, Political and Health System, and Structural Factors. |
| **Systematic Review Methods** | | |
| **Conceptual framework** | Not reported. | |
| **Data extraction method** | Information extracted is described but the process is not reported. | |
| **Appraisal tool used** | Not reported | |
| **Data synthesis method** | Thematic synthesis. | |

| **JBI Quality Appraisal** | | |
| --- | --- | --- |
| **Question** | **Judgement** | **Justification** |
| **1. Is the review questions clearly and explicitly stated?** | Yes | Paper aimed to conduct a comprehensive scoping review of published qualitative research about engagement with HIV care in Indonesia. Research question: What does qualitative research contribute to what is known about the HIV care cascade in Indonesia? |
| **2. Were the inclusion criteria appropriate for the review question?** | Yes | Excluded other languages, countries, also excluded quantitative, and non-peer reviewed studies. |
| **3. Was the search strategy appropriate?** | Unclear | The following search terms were used: (‘HIV’ OR ‘HIV infection’) AND (‘qualitative’ OR ‘qualitative research’ OR ‘ethnography’) AND (‘Indonesia’). The publication period included all years between 1990 and 2016. English only. Full search strategy not provided. |
| **4. Were the sources and resources used to search for studies adequate?** | Yes | Scopus, Medline, ProQuest and Web of Science was searched. Further searches were conducted using reference lists of relevant papers, the UNSW Sydney library and Google Scholar between July and December 2016. An additional search of a journal called Indonesia was performed using the same search terms used to search electronic databases. Although the did not include grey literature and should be judged an N, the aim of the paper was to review published literature, therefore judged as adequate. |
| **5. Was selection of studies done adequately?** | Unclear | Method of selection not reported |
| **6. Were the criteria for appraising studies appropriate?** | Unclear | Critical appraisal not reported |
| **7. Was the critical appraisal conducted by two or more reviewers independently?** | Unclear | Critical appraisal not reported |
| **8. Were there methods to minimize errors in the data extraction?** | No | Information that was extracted was described but not by whom and if it was done independently or in duplicate. |
| **9. Were the methods used to combine studies appropriate?** | Unclear | Authors used thematic synthesis but do not describe how it was implemented and by whom. |
| **10. Were recommendations for policy and/or practice supported by the reported data?** | Yes | The study conclusions regarding the usefulness of qualitative data to support programme initiatives and government policies are evidenced in the data that they provided. |
| **11. Were the specific directives for new research appropriate?** | Yes | The study recommends more qualitative research within key populations and identifies the gaps in evidence in the Indonesian setting. |

*Li*

| **Characteristics of included systematic review Li et al. 2016** | | |
| --- | --- | --- |
| **Citation** | | |
| Li, H. et al., 2016. The Role of ARV Associated Adverse Drug Reactions in Influencing Adherence Among HIV-Infected Individuals: A Systematic Review and Qualitative Meta-Synthesis. AIDS Behav, (PG-1-11), pp.1–11. Available at: https://www.scopus.com/inward/record.uri?eid=2-s2.0-84986243900&partnerID=40&md5=eaf19d18950931a8483f9b5583fc7e08 NS  -. | | |
| **Search Summary Details** | | |
| **Literature Searched** | CENTRAL, EMBASE, LILACS,PsychINFO, PubMed (MEDLINE), Web of Science/Web of Social Science, CINAHL, British Nursing Index and Archive, Social Science citation Index, AMED, DAI, EPPI-Centre, ESRC, Global Health (EBSCO), Anthrosource, JSTOR, conference proceedings include the conferences on Retroiruses and Opportunistic Infections (CROI), International AIDS conference (IAC), and alternating year International AIDS Society (IAS). Reference mining of included studies were conducted. | |
| **Search Dates** | 1st January 2000 until 21st February 2015 | **Date of last search: 21 Feb 2015** |
| **Search Criteria and Outcomes** | | |
|  | **What the review authors searched for** | **What the review authors found** |
| **Studies** | All studies in English, studies were selected that used qualitative research designs, including but not limited to ethnographic research, case studies, process evaluations, and mixed methods research using a framework analysis approach | 39 Qualitative Studies |
|  |  | **Studies relevant to this overview:** |
|  |  | 39 Qualitative Studies |
| **Participants** | HIV-infected individuals | Total: N=192434 HIV- infected individuals including adults, children, adolescents, pregnant and post-partum women and caregivers. |
| **Issue** | Adherence | Adherence (n=39) |
| **Setting** | HIC and LMIC | HIC (19): USA (14), Netherlands (1), Canada (1), Australia (1), Belgium and Netherlands (1), Switzerland (1) and LMIC (20): Botswana, Tanzania and Uganda (1), Peru (1), Ukraine (1), Zambia (1), Rwanda (1), Ethiopia (1), Uganda (1), Nepal (2), Cuba (1), Southern Malawi (1), Uganda and Zimbabwe (1), China (2), Tanzania (3), South Africa (3) |
| **Outcomes: Barriers and Facilitators** | Perception and experience of ARV adverse drug reactions | **Overview Framework:** Individual Factors |
| **Systematic Review Methods** | | |
| **Conceptual Framework:** | None Specified | |
| **Data extraction method** | Pre-specified data extraction form | |
| **Appraisal tool used** | CASP tool | |
| **Methods of data synthesis:** | Thematic Synthesis | |

| **JBI Quality Appraisal** | | |
| --- | --- | --- |
| **Question** | **Judgement** | **Justification** |
| **1. Is the review questions clearly and explicitly stated?** | Yes | The aim of this qualitative synthesis was to systematically review and synthesize the qualitative literature examining how perception and experience of ARV adverse drug reactions influence drug adherence among HIV-infected individuals |
| **2. Were the inclusion criteria appropriate for the review question?** | Yes | Studies were selected that used qualitative research designs, including but not limited to ethnographic research, case studies, process evaluations, and mixed methods research. Studies also had to include qualitative data collection techniques (e.g., participant observation, in-depth interview, or focus group) and a qualitative data analysis approach (e.g., framework analysis, or thematic analysis). The review included studies that provided description and interpretation of the impact of adverse drug reactions on adherence for all HIV-infected individuals. We excluded studies that only used quantitative methods to investigate the same phenomenon. |
| **3. Was the search strategy appropriate?** | No | In accordance with guidance from PRISMA checklist, ENTREQ, and meta-synthesis guidance from the Cochrane group, we used a comprehensive search strategy to identify all relevant studies in English. Search strategy available in supplementary material. Only included English studies. |
| **4. Were the sources and resources used to search for studies adequate?** | Yes | The following electronic journal and dissertation/thesis databases were searched from January 1st, 2000 until February 21st 2015 (to limit to recent ARV drug regimens): CENTRAL (Cochrane Central Register of Controlled Trials), EMBASE, LILACS, PsycINFO, PubMed (MEDLINE), Web of Science/Web of Social Science, CINAHL, British Nursing Index and Archive, Social science citation index, AMED (Allied and Complementary Medicine Database), DAI (Dissertation Abstracts International), EPPI-Centre (Evidence for Policy and Practice Information and Coordinating Centre), ESRC (Economic and Social Research CoUil), Global Health (EBSCO), Anthrosource, and JSTOR. Conference proceedings including the Conferences on Retroviruses and Opportunistic Infections (CROI), International AIDS Conference (IAC), and alternating year International AIDS Society (IAS) clinical meetings were searched from their inception dates (1993, 1985 and 2001, respectively). References of included studies were checked and authors were contacted to provide additional information as required |
| **5. Was selection of studies done adequately?** | Yes | Two authors independently reviewed abstracts and made a list of categories for exclusion, and then independently reviewed full text manuscripts and notes reasons for exclusion. Discordance between the two authors was resolved by discussion with a third author. |
| **6. Were the criteria for appraising studies appropriate?** | Yes | The seven scale CASP quality assessment tool was used, however they do not describe the domains of the CASP |
| **7. Was the critical appraisal conducted by two or more reviewers independently?** | Yes | Prior to conducting the synthesis, each paper’s quality was assessed by two authors and using a seven scale criteria tool adapted from the critical appraisal skills programme (CASP), which has been used in other reviews |
| **8. Were there methods to minimize errors in the data extraction?** | Unclear | Pre-specified data extraction form. Data extracted authors, year published, study design, location, key populations/subgroups. Not reported whether data extraction was done by two authors |
| **9. Were the methods used to combine studies appropriate?** | Yes | Thematic synthesis was adopted in the data analysis. Two authors conducted a line-by-line coding of the findings of primary studies prior to organizing into related areas to construct descriptive themes. Analytical themes were developed based on the descriptive themes. Pre-defined subgroup analyses were conducted among specific groups of adults, children and adolescents, and pregnant women. CERQUAL was also used to appraise confidence in the findings. |
| **10. Were recommendations for policy and/or practice supported by the reported data?** | Yes | This synthesis identified 39 qualitative studies from diverse countries and data supported the recommendations for a structured informing approach about the adverse reactions of the medication. All recommendations supported. |
| **11. Were the specific directives for new research appropriate?** | Yes | This qualitative synthesis found very limited studies considering perspectives of children, adolescent, and pregnant women, and the findings were in moderate and low confidence levels respectively. Future qualitative studies should focus on exploring subjective experiences and perceptions of ARV adverse drug reactions from their perspectives. Results support the conclusion. |

*Lytvyn*

| **Characteristics of included systematic review Lytvyn et al 2017** | | |
| --- | --- | --- |
| **Citation** | | |
| Lytvyn L, Siemieniuk RA, Dilmitis S, Ion A, Chang Y, Bala MM, et al. Values and preferences of women living with HIV who are pregnant, postpartum or considering pregnancy on choice of antiretroviral therapy during pregnancy. BMJ Open. 2017;7(9):1–9. | | |
| **Search Summary Details** | | |
| **Literature Searched** | MEDLINE, EMBASE and PsycINFO. Included grey literature and performed an additional search on GOOGLE. | |
| **Search Dates** | 1 January 2000 to 11 February 2017 | **Date of last search:** 12 February 2017 |
| **Search Criteria and Outcomes** | | |
|  | **What the review authors searched for** | **What the review authors found relevant to this overview** |
| **Studies** | Quantitative and qualitative studies from any setting and location. | 15 Studies: Qualitative (15) |
|  |  | **Studies relevant to this overview:** |
|  |  | 15 Studies: Qualitative (15) |
| **Participants** | Women living with HIV of childbearing age (15-50 years), who were diagnosed at any time, that evaluated the initiation, adherence or change in ART regimen during pregnancy | N=1165: Women considering pregnancy (140), pregnant women (408), and post-partum women (602). Couples desiring and/or intending to have children (15) also included. |
| **Issue** | Linkage and adherence | Adherence (15) |
| **Setting** | Any setting | Puerto Rico (1), Nigeria (1), Kenya (2), Swaziland (2), Malawi (2), India (1), Australia (1), South Africa (1), Zimbabwe (1), and USA (3) |
| **Outcome: Barriers and Facilitators** | Values and preferences of women considering or discussing ART during pregnancy | **Overview Framework:** Individual and intrapersonal factors. |
| **Systematic Review Methods** | | |
| **Conceptual framework** | Not reported | |
| **Data extraction method** | Two reviewers independently and in duplicate extracted data using a standardised spreadsheet in EXCEL | |
| **Appraisal tool used** | CASP Tool | |
| **Data synthesis method** | Meta-ethnography | |

| **JBI Quality Appraisal** | | |
| --- | --- | --- |
| **Question** | **Judgement** | **Justification** |
| **1. Is the review questions clearly and explicitly stated?** | Yes | To inform the BMJ Rapid Recommendation on cART regimens in pregnancy, we performed this systematic review to explore how women living with HIV value possible benefits and harms of ART. |
| **2. Were the inclusion criteria appropriate for the review question?** | Yes | Considered eligible both quantitative and qualitative studies from any setting and location, enrolling women living with HIV of childbearing age (15–50 years), who were diagnosed at any time, that evaluated the initiation, adherence or change in ART regimen during pregnancy. This inclusion criterion is appropriate for the review question. |
| **3. Was the search strategy appropriate?** | Yes | Used a combination of keywords and MeSH/EMTREE terms for ‘HIV’ AND ‘pregnancy’ AND ‘antiretroviral therapy’. Search strategy was available in supplementary material and is appropriate. All languages were included in the search. |
| **4. Were the sources and resources used to search for studies adequate?** | Yes | MEDLINE, EMBASE and PsycINFO. Included grey literature and performed an additional search on GOOGLE. |
| **5. Was selection of studies done adequately?** | Yes | Title and abstract as well as full-text screening was done independently and in duplicate by reviewer pairs (LL, MMB, YC). Disagreements were resolved by consensus. |
| **6. Were the criteria for appraising studies appropriate?** | Yes | All eligible studies reported qualitative outcomes. We used the Critical Appraisal Skills Programme checklist to assess methodological quality of individual qualitative studies. |
| **7. Was the critical appraisal conducted by two or more reviewers independently?** | Unclear | Not reported |
| **8. Were there methods to minimize errors in the data extraction?** | Unclear | Two reviewers independently and in duplicate extracted data using a standardised spreadsheet in EXCEL. Detailed description of data was extracted is provided. |
| **9. Were the methods used to combine studies appropriate?** | Yes | Authors used thematic synthesis and explain the steps that they followed. First extracted all quotes and author identified themes relevant to taking ART and these were coded and categorised to identify common themes. |
| **10. Were recommendations for policy and/or practice supported by the reported data?** | Yes | Based on the findings in this specific population recommendations are made for practice. These are supported by the data. |
| **11. Were the specific directives for new research appropriate?** | Yes | The authors make several directives for research including no studies have addressed women being given the choice of ART alternatives or about the priorities of outcomes related to taking ART. Methodological recommendations for research are provided such as reflexivity and transparency. These are supported and appropriate. |

*Merten*

| **Characteristics of included systematic review Mertern et al. 2010** | | |
| --- | --- | --- |
| **Citation** | | |
| Merten, S. et al., 2010. Patient-reported barriers and drivers of adherence to antiretrovirals in sub-Saharan Africa: a meta-ethnography. Trop Med Int Health, 15 Suppl 1(PG-16-33), pp.16–33. Available at: NS  -. | | |
| **Search Summary Details** | | |
| **Literature Searched** | Medline, AIO, SSCI, CINAHL, IBSS, EMBASE and JSTOR and additional hand searches | |
| **Search Dates** | 2000-2008 | **Date of last search: 5 January 2010** |
| **Search Criteria and Outcomes** | | |
|  | **What the review authors searched for** | **What the review authors found** |
| **Studies** | Qualitative studies with qualitative design and analysis method | 32 Qualitative studies |
|  |  | **Studies relevant to this overview:** |
|  |  | 32 Qualitative studies |
| **Participants** | PLHIV | N=2044+ Community members, policy makers, HIV+ patients, health workers, female HIV+ patients, healthcare actors, In-school and out-of-school youth, patients who attended the ARV clinic, counsellors, HIV+ patients on ART for 6 months, care givers, family care givers, key informants, HIV+ patients from IDP camps, treatment partners |
| **Issue** | ART access and adherence in cultural settings | Adherence in cultural settings (32) |
| **Setting** | Sub-Saharan Africa | Uganda (6), Zambia (5), South Africa (6), Burkina Faso (1), Malawi (2), Tanzania (5), Botswana (2), Kenya (1), Nigeria (1), Ethiopia and Uganda (1), Burkina Faso, Cote d'Ivoire and Mali (1), Nigeria, Tanzania and Uganda (1) (LMIC) |
| **Outcomes: Barriers and Facilitators** | Patient reported barriers and drivers of adherence in cultural settings | **Overview Framework:** Individual, Interpersonal, Community and Social, Political and Health System, and Structural Factors. |
| **Systematic Review Methods** | | |
| **Conceptual framework** | None reported. | |
| **Data extraction method** | Data extraction was conducted in duplicate; however, there is no mention of a standard data extraction form. | |
| **Appraisal tool used** | Appraisal conducted but no procedure or process reported. | |
| **Data synthesis method** | Meta-ethnography | |

| **JBI Quality Appraisal** | | |
| --- | --- | --- |
| **Question** | **Judgement** | **Justification** |
| **1. Is the review questions clearly and explicitly stated?** | No | This article presents an update and systematic review of findings from qualitative studies on ART adherence in SSA and discusses how these factors interact. The question is not clearly formulated. |
| **2. Were the inclusion criteria appropriate for the review question?** | Unclear | Studies using a qualitative design and analysis method were included. Appropriate for use with meta-ethnography. Participants not clearly identified in the inclusion criteria. |
| **3. Was the search strategy appropriate?** | No | Indicated keywords. Articles reported findings from 2000-2008. No search strategy reported. No language criteria. |
| **4. Were the sources and resources used to search for studies adequate?** | Yes | Medline, AIO, SSCI, CINAHL, IBSS, EMBASE and JSTOR and additional hand searches. No grey literature included. Authors report hand searches but not how, where or by whom it was conducted. |
| **5. Was selection of studies done adequately?** | No | Titles and abstracts were reviewed in duplicate by three researchers. Removed duplicates. No information regarding full text screening. |
| **6. Were the criteria for appraising studies appropriate?** | Unclear | Articles were appraised but there is no mention of the tool that was used or the procedure that was followed. |
| **7. Was the critical appraisal conducted by two or more reviewers independently?** | Unclear | Not reported |
| **8. Were there methods to minimize errors in the data extraction?** | Unclear | Data extraction was conducted in duplicate; however, there is no mention of a standard data extraction form. Additionally, data extracted into Table 1 is for 32 studies but authors report 31 studies in PRISMA chart and search strategy and outcomes. |
| **9. Were the methods used to combine studies appropriate?** | Yes | The method of meta-ethnography has proven to be an effective tool to synthesize qualitative studies to attain deeper understanding of complex health-related topics, such as adherence to medication for chronic conditions. While a literature review summarizes the main findings of qualitative studies on a single theme, meta-ethnography aims at synthesis, which ‘involves the juxtaposition of studies and the connections between them, to develop a more sophisticated understanding. Key themes and concepts were extracted and peer reviewed for inclusiveness. Key themes found were consolidated into a line of argument (third-order analysis), which is presented in the synthesis/discussion section. |
| **10. Were recommendations for policy and/or practice supported by the reported data?** | Unclear | The review does not provide information regarding the way forward for policy and practice |
| **11. Were the specific directives for new research appropriate?** | Unclear | Recommendations for further research has not been provided |

*Mey*

| **Characteristics of included systematic review Mey et al. 2017** | | |
| --- | --- | --- |
| **Citation** | | |
| Mey, A. et al., 2016. Motivations and Barriers to Treatment Uptake and Adherence Among People Living with HIV in Australia: A Mixed-Methods Systematic Review. AIDS Behav, (PG-1-34), pp.1–34. Available at: https://www.scopus.com/inward/record.uri?eid=2-s2.0-84994388389&partnerID=40&md5=fa7d78974dfe5881731fabdbc91d2be3 NS  -. | | |
| **Search Summary Details** | | |
| **Literature Searched** | Ovid Medline, CINAHL, Scopus, Health and Society Database and Sociological Abstracts | |
| **Search Dates** | January 2000-15 December 2015 | **Date of last search:** Between November and December 2015 |
| **Search Criteria and Outcomes** | | |
|  | **What the review authors searched for** | **What the review authors found** |
| **Studies** | Qualitative, quantitative and mixed methods studies found in published literature. | 72 studies: Qualitative (30) Quantitative (34), Mixed methods (6), Case Report (1) and Short Communication Article (1). |
|  |  | **Studies relevant to this overview:** |
|  |  | 35 Studies: Qualitative (7), Quantitative (21), Mixed Methods (6), and 1 Case report. |
| **Participants** | People living with HIV | PLHIV, Men, women, MSM, caregivers of children who are HIV positive, CAM workers (traditional healers/alternative medicines) |
| **Issue** | Treatment uptake (linkage) and Treatment Adherence | Linkage to ART (13) and Adherence (26) and Retention (7) from 21 studies. |
| **Setting** | Australia | Australia (21) |
| **Outcomes: Barriers and Facilitators** | Identify motivations and barriers to treatment uptake and adherence | **Overview Framework:** Individual, Interpersonal, Community and Social, Political and Health System, and Structural Factors. |
| **Systematic Review Methods** | | |
| **Conceptual framework** | No framework reported | |
| **Data extraction method** | Only a second author independently reviewed a random selection of articles. | |
| **Appraisal tool used** | Quality appraisal utilised the Mixed Methods Appraisal Tool (MMAT) Version 2011 | |
| **Data synthesis method** | Narrative synthesis | |

| **JBI Quality Appraisal** | | |
| --- | --- | --- |
| **Question** | **Judgement** | **Justification** |
| **1. Is the review questions clearly and explicitly stated?** | Yes | The purpose of this research is to provide a greater understanding of the perspectives of PLHIV and to identify motivations and barriers to treatment uptake and adherence. |
| **2. Were the inclusion criteria appropriate for the review question?** | No | Studies were included if they referred to i) living with HIV, ii) treatment uptake and iii) treatment adherence. Qualitative, quantitative and mixed methods studies were reviewed. Articles were considered for analysis if they described the experiences, knowledge, attitudes and beliefs of people living with HIV in the Australian context. Included studies from 2000, which may include data reported on that, may not refer to aspects of medication and care in practice after 2000. |
| **3. Was the search strategy appropriate?** | Yes | Articles published post 2000 until December 15 2015. Full search strategy available in text and is adequate. |
| **4. Were the sources and resources used to search for studies adequate?** | No | Ovid Medline, CINAHL, Scopus, Health and Society Database and Sociological Abstracts were accessed between November and December 2015. Review articles and grey literature were excluded from analysis but their reference lists were scrutinised and in some cases, yielded new articles to include in the analysis. Keywords were indicated as well |
| **5. Was selection of studies done adequately?** | No | Authors report that one author screened titles and abstracts, and a second author to check that none was missed for inclusion reviewed abstracts. One author did full text screening and another author checked a random sample of excluded studies. Full text should have been conducted in duplicate and independently. |
| **6. Were the criteria for appraising studies appropriate?** | Yes | Quality appraisal utilised the Mixed Methods Appraisal Tool (MMAT) Version 2011. Mixed methods studies were assessed according to: i) appropriateness of the research design, ii) appropriateness of triangulation, and iii) appropriate acknowledgement of limitations. For qualitative studies, methodological quality was assessed according to the relevance/appropriateness of four domains relative to the research question: i) the data source ii) the analytical process, iii) the findings and iv) reflexivity |
| **7. Was the critical appraisal conducted by two or more reviewers independently?** | Unclear | It was reported that the search strategy, data table and study quality assessment were reviewed by SD and disagreement resolved by consensus" but there is no mention of a second author that did critical appraisal, therefore judged as unclear. |
| **8. Were there methods to minimize errors in the data extraction?** | No | Only a random selection of articles were independently reviewed by a second author. |
| **9. Were the methods used to combine studies appropriate?** | Yes | Data was synthesised using thematic analysis. Independent reviewers analysed a random selection of studies and developed a codebook then discussed their views and agreed on a coding framework. One author coded the remaining articles. The remaining authors each reviewed 5 articles that were coded in relation to the framework for discussion. Final themes were based on the outcomes of discussion. This was found adequate. |
| **10. Were recommendations for policy and/or practice supported by the reported data?** | Unclear | The review does not provide information regarding the way forward for policy and practice |
| **11. Were the specific directives for new research appropriate?** | Yes | While the study provides clues, more research is needed to understand how these meanings are generated and how they give rise to rationales regarding HIV management. In addition, a review of studies focused on testing and risk-taking behaviour is required to provide a holistic overview of the barriers and facilitators to the effective management of HIV in the Australian context. |

*Mill*

| **Characteristics of included systematic review Mill et al. 2006** | | |
| --- | --- | --- |
| **Citation** | | |
| Mills, E.J. et al., 2006. Adherence to HAART: a systematic review of developed and developing nation patient-reported barriers and facilitators. PLoS Med, 3(11 PG-e438), p.e438. Available at: NS  -. | | |
| **Search Summary Details** | | |
| **Literature Searched** | AMED, Campbell Collaboration, CINAHL, Cochrane Library, Embase, ERIC, MedLine, NHS EED. The UK national research register and conference abstracts were sourced from international conference web sites. Searched bibliographies of key papers. | |
| **Search Dates** | Up to June 2005 | **Date of last search**: Not specified |
| **Search Criteria and Outcomes** | | |
|  | **What the review authors searched for** | **What the review authors found** |
| **Studies** | Qualitative and quantitative studies. No language exclusions. Published literature and conference abstracts. | 85 studies: 38 qualitative studies and 47 quantitative survey studies |
|  |  | **Studies relevant to this overview:** |
|  |  | 85 studies: 38 qualitative studies and 47 quantitative survey studies |
| **Participants** | PLHIV | People living with HIV and caregiver N=12902 |
| **Issue** | Barriers and facilitators to antiretroviral adherence | Barriers and facilitators to antiretroviral adherence |
| **Setting** | Representative of developed and developing nations according to the United Nations Development Index | 72 studies conducted in developed countries: Fifty-six were from the US, Canada (3), UK (3), Italy (2), France (2), The Netherlands (2), Australia (1), Belgium (1) and Switzerland (1). The studies conducted in developing countries included Brazil (1) and Botswana (1) Two studies were multi-national: (countries not reported). 12 studies were conducted in developing countries included four from Brazil and one each from Uganda, Cote d’Ivoire, South Africa, Malawi, Botswana, Costa Rica, Romania and China. |
| **Outcomes: Barriers and Facilitators** | Patient reported barriers and facilitators | **Overview Framework:** Individual, Interpersonal, Community and Social, Political and Health System, and Structural Factors |
| **Systematic Review Methods** | | |
| **Conceptual framework** | No framework reported | |
| **Data extraction method** | Qualitative data was analysed using content analysis and quantitative data was pooled together. | |
| **Appraisal tool used** | Authors used their pre-specified appraisal tool specific for qualitative studies and another for quantitative studies. | |
| **Data synthesis method** | Content analysis | |

| **JBI Quality Appraisal** | | |
| --- | --- | --- |
| **Question** | **Judgement** | **Justification** |
| **1. Is the review questions clearly and explicitly stated?** | Yes | Review question is not clearly stated with the PICO elements clearly identified in the introduction. In the search strategy, the aim of the study is identified. |
| **2. Were the inclusion criteria appropriate for the review question?** | Yes | Eligible studies met the following criteria: (1) reported an original research study, (2) contained content addressing barriers or facilitators to antiretroviral adherence, and (3) were either a qualitative study or quantitative survey. All languages were included. Criteria was appropriate. |
| **3. Was the search strategy appropriate?** | Unclear | Keywords have been indicated. Author has been contacted for full search strategy. Awaiting response. |
| **4. Were the sources and resources used to search for studies adequate?** | Yes | Databases searched included: AMED, Campbell Collaboration, CINAHL, Cochrane Library, Embase, ERIC, MedLine, NHS EED. Grey literature was searched (unpublished studies) in the UK national research register and conference abstracts were sourced from international conference web sites. Reference mining was also conducted. Published and unpublished studies were sourced. |
| **5. Was selection of studies done adequately?** | Unclear | Two authors independently reviewed abstracts. Nevertheless, they do not describe how they selected full texts. |
| **6. Were the criteria for appraising studies appropriate?** | Yes | We extracted data on the quality of both qualitative and quantitative studies using pre-determined criteria for quality. We previously reported our rationale for assessing the quality of qualitative studies and in this study have extended our quality assessment to examine quantitative surveys. Authors describe the domains used for appraisal in detail for both quantitative and qualitative studies. |
| **7. Was the critical appraisal conducted by two or more reviewers independently?** | Yes | Two reviewer independently extracted data and appraised the quality and content of the studies. |
| **8. Were there methods to minimize errors in the data extraction?** | Yes | A coding template was iteratively developed through initial extraction that was then used for a second review of the papers to identify barriers and facilitators. Data extraction was done independently and in duplicate. |
| **9. Were the methods used to combine studies appropriate?** | Yes | Qualitative data was analysed using content analysis and quantitative data was pooled together. These were adequate. |
| **10. Were recommendations for policy and/or practice supported by the reported data?** | Yes | The review identified a broad range of barriers and facilitators to adherence. These barriers should be inferred as guides for interventional research to improve adherence rates. Given the many factors tabulated in this review, clinicians should use this information to engage in open discussion with patients to promote adherence and identify barriers and facilitators within their own populations. Recommendations were supported by data. |
| **11. Were the specific directives for new research appropriate?** | Yes | Further research on HAART adherence in developing countries that incorporates both qualitative and quantitative elements as a priority. Directives provided were appropriate. |

*Morales-Aleman*

| **Characteristics of included systematic review Morales-Aleman et al. 2014** | | |
| --- | --- | --- |
| **Citation** | | |
| Morales-Aleman, M.M. & Sutton, M.Y., 2014. Hispanics/Latinos and the HIV continuum of care in the Southern USA: a qualitative review of the literature, 2002–2013. AIDS Care, 26(12 PG-1592-1604), pp.1592–1604. Available at: http://search.ebscohost.com/login.aspx?direct=true&db=cin20&AN=103905221&site=ehost-live NS  -. | | |
| **Search Summary Details** | | |
| **Literature Searched** | PsychInfo, PubMed, Medline, and Google Scholar | |
| **Search Dates** | Jan 2002 - April 2013 | **Date of last search: Not specified** |
| **Search Criteria and Outcomes** | | |
|  | **What the review authors searched for** | **What the review authors found** |
| **Studies** | Empirical studies published in peer review journals | 13 studies: 10 quantitative studies, 3 qualitative studies |
|  |  | **Studies relevant to this overview:** |
|  |  | 3 qualitative studies and 1 quantitative study. |
| **Participants** | Studies reporting sample of >50% Hispanics and Latinos LHIV | N=121 Hispanic and Latino PLHIV |
| **Issue** | Factors associated with the HIV continuum of care at various stages: diagnosis/testing, Linkage/Retention in Care; and Adherence. | Linkage to Care (3), Adherence (1), Retention in Care (3) from 4 studies. |
| **Setting** | Southern USA | Southern USA 4) |
| **Outcomes: Barriers and Facilitators** | Barriers, facilitators and gaps in HIV testing and Care | **Overview Framework:** Individual, Interpersonal, Community and Social, Political and Health System, and Structural Factors |
| **Systematic Review Methods** | | |
| **Framework** | HIV Continuum of care | |
| **Data extraction method** | Summarised into tables. No indication who conducted it. | |
| **Appraisal tool used** | Not reported | |
| **Data synthesis method** | Thematic Analysis | |

| **JBI Quality Appraisal** | | |
| --- | --- | --- |
| **Question** | **Judgement** | **Justification** |
| **1. Is the review questions clearly and explicitly stated?** | No | Review states: We conducted a systematic review of the literature to examine each step of the HIV continuum of care, a framework that has recently gained nationwide attention and utilization, for Hispanics/Latinos in the southern USA |
| **2. Were the inclusion criteria appropriate for the review question?** | No | We included empirical articles that (1) were published in peer-reviewed journals between January 2002 and April 2013; (2) included samples of ≥50% Hispanics/Latinos; (3) were conducted exclusively in one or more Southern US states; and (4) identified or examined at least one of the five aspects of the continuum of care. We extracted and summarized these data in table form. Articles were analysed based on the HIV continuum of care stages, utilizing directed content analysis. We identified significant variables, barriers, facilitators, and gaps in HIV testing and care for Hispanics/Latinos in the South |
| **3. Was the search strategy appropriate?** | Unclear | Searches were limited to peer-reviewed, original research articles and abstracts published between 1 January 2002 and 1 April 2013. Keywords have been indicated. Full search strategy is not reported. |
| **4. Were the sources and resources used to search for studies adequate?** | No | PsychInfo, PubMed, Medline, and Google Scholar. No grey literature included or attempts to find unpublished studies. |
| **5. Was selection of studies done adequately?** | Unclear | Removed duplicates. Screened studies that appeared to be potentially related to ART adherence. Selection process is not described adequately. |
| **6. Were the criteria for appraising studies appropriate?** | Unclear | Not reported |
| **7. Was the critical appraisal conducted by two or more reviewers independently?** | Unclear | Not reported |
| **8. Were there methods to minimize errors in the data extraction?** | Unclear | Data was extracted and summarised in table form. Who conducted it and methods to minimise errors not reported. |
| **9. Were the methods used to combine studies appropriate?** | Yes | Articles were analysed based on the HIV continuum of care stages using direct content analysis to identify significant variables, barriers, facilitators, and gaps in HIV testing and care. Adequate. |
| **10. Were recommendations for policy and/or practice supported by the reported data?** | Yes | Increased implementation of effective provider strategies at the individual-, clinician-, and organizational-level may be effective in increasing access to HIV care for Hispanics/Latinos in the South. Open dialog among stakeholders (e.g., Hispanic/Latino immigrants, health-care policy-makers, and health-care providers) about HIV care access will be a vital step in reducing HIV-related health disparities as part of domestic HIV prevention efforts. These are some findings such as local media coverage that is not addressed in the recommendations. However, recommendations made are supported by the data. |
| **11. Were the specific directives for new research appropriate?** | Yes | Future studies should consider country of origin, geographic location, level of acculturation unique roles in the context of barriers and facilitators. Authors make relevant suggestions on future research methods to use participatory approaches. |

*Omonaiye*

| **Characteristics of included systematic review** | | |
| --- | --- | --- |
| **Citation** | | |
| Omonaiye O, Kusljic S, Nicholson P, Manias E. Medication adherence in pregnant women with human immunodeficiency virus receiving antiretroviral therapy in sub-Saharan Africa: a systematic review. 2018;1–20. Available from: https://doi.org/10.1186/s12889-018-5651-y | | |
| **Search Summary Details** | | |
| **Literature Searched** | MEDLINE Complete (1916-Dec 2017), Embase (1947-Dec 2017), Global Health (1910-Dec 2017) and CINAHL Complete (1937-Dec 2017). Included papers were searched for additional papers. | |
| **Search Dates** | Up to December 2017 | **Date of last search: Not reported** |
| **Search Criteria and Outcomes** | | |
|  | **What the review authors searched for** | **What the review authors found relevant to this overview** |
| **Studies** | Qualitative and quantitative studies. No language and publication restrictions. | 51 Studies: Qualitative (9), Quantitative (36) and Mixed Methods (6) |
|  |  | **Studies relevant to this overview:** |
|  |  | 15 Studies: Qualitative (9) and Mixed Methods (6) |
| **Participants** | HIV positive pregnant women | HIV positive pregnant women (include number) |
| **Issue** | Adherence to ART | Adherence (15) |
| **Setting** | sub-Saharan Africa | Kenya (3), Swaziland (1), Uganda (2), South Africa (1), Cote d'voire (2), Tanzania (1), Malawi (4), Mozambique (1) |
| **Outcome: Barriers and Facilitators** | Patient related factors, condition, therapy related factors, and health care team and health system factors. | **Overview Framework:** Individual, Interpersonal, Community and Social, Political and Health System, and Structural Factors |
| **Systematic Review Methods** | | |
| **Conceptual framework** | WHO Multidimensional adherence model | |
| **Data extraction method** | The data was extracted by one author and then reviewed by the other authors and discrepancies resolved via discussion | |
| **Appraisal tool used** | Used the Mixed Methods Appraisal Tool version 2011 | |
| **Data synthesis method** | Thematic Analysis | |

| **JBI Quality Appraisal** | | |
| --- | --- | --- |
| **Question** | **Judgement** | **Justification** |
| **1. Is the review questions clearly and explicitly stated?** | Yes | The aim of this review is to examine barriers and enablers of medication adherence to ART among HIV positive pregnant women in sub-Saharan Africa. |
| **2. Were the inclusion criteria appropriate for the review question?** | Yes | Inclusion criteria was specific to the question. |
| **3. Was the search strategy appropriate?** | Unclear | HIV AND (Pregnancy OR Pregnant*) AND (PMTCT OR “PMTCT Cascade” OR “Vertical Transmission” OR “Mother-to-Child”) AND (Prevent OR Prevention) AND (HAART OR “Antiretroviral Therapy” OR “Triple Therapy”) AND (Retention OR Concordance OR adherence OR Compliance). Example specific to database not provided. |
| **4. Were the sources and resources used to search for studies adequate?** | No | MEDLINE Complete (1916-Dec 2017), Embase (1947-Dec 2017), Global Health (1910-Dec 2017) and CINAHL Complete (1937-Dec 2017). Included papers were searched for additional papers. No grey literature included. |
| **5. Was selection of studies done adequately?** | Yes | One researcher (O.O.) initiated and screened all titles and abstracts to identify potentially relevant studies. A second researcher independently screened all titles and abstracts to identify potentially relevant studies (E.M.). Three researchers (O.O., E.M. and S.K.) to determine whether they met the inclusion criteria independently examined the full texts of potentially relevant studies. |
| **6. Were the criteria for appraising studies appropriate?** | Yes | Used the Mixed Methods Appraisal Tool version 2011, which provides criteria to evaluate the methodological quality of studies. 19 items assessing quality and possible risk of bias. Tool may be used with different study designs. This was judged Y |
| **7. Was the critical appraisal conducted by two or more reviewers independently?** | Yes | Two researchers conducted assessment independently and in duplicate. |
| **8. Were there methods to minimize errors in the data extraction?** | Yes | Although a standard template was used. One author only did data extraction. The extraction was reviewed by the other authors and discrepancies resolved via discussion. |
| **9. Were the methods used to combine studies appropriate?** | Unclear | WHO Multidimensional adherence model was used to categorize findings. The presentation of results and narrative synthesis provided is adequate. However, the method of synthesis is not reported. |
| **10. Were recommendations for policy and/or practice supported by the reported data?** | Yes | The recommendations are well linked to the data and themes. Authors also provide feedback on the limitations of the data. |
| **11. Were the specific directives for new research appropriate?** | Unclear | No directives provided. |

*Reisner*

| **Characteristics of included systematic review Reisner et al. 2009** | | |
| --- | --- | --- |
| **Citation** | | |
| Reisner, S.L. et al., 2009. A review of HIV antiretroviral adherence and intervention studies among HIV-infected youth. Top HIV Med, 17(1 PG-14-25), pp.14–25. Available at: NS  -. | | |
| **Search Summary Details** | | |
| **Literature Searched** | Medline, PubMed, PsychInfo. In addition, bibliographies of relevant articles were reviewed for additional studies. | |
| **Search Dates** | 1999-2008 | **Date last search: Not specified** |
| **Search Criteria and Outcomes** | | |
|  | **What the review authors searched for** | **What the review authors found** |
| **Studies** | Qualitative and quantitative published studies. | 21 Studies: Qualitative (4), Quantitative (7), Mixed Methods (3) and 7 Intervention Studies. |
|  |  | **Studies relevant to this overview:** |
|  |  | 14 Studies: Qualitative (4), Quantitative (7) and Mixed Methods (3) |
| **Participants** | HIV positive adolescents and youth ages 13 to 24 years | N=5179 HIV positive youth and adolescents and pregnant adolescents. |
| **Issue** | Medication adherence and exercising an intervention technique to enhance antiretroviral adherence. | Adherence (14) |
| **Setting** | United States | United States (14) |
| **Outcomes: Barriers and Facilitators** | Demographic, psychosocial, HIV disease, treatment regimen and practitioner factors | **Overview Framework:** Individual, Interpersonal, Community and Social, Political and Health System, and Structural Factors |
| **Systematic Review Methods** | | |
| **Conceptual Framework** | Not specified | |
| **Data extraction method** | Not specified | |
| **Appraisal tool used** | Not specified | |
| **Data synthesis method:** | Thematic content analysis | |
| **Limitations:** | All studies included regardless of methodological rigour, no search strategy provided. No duplicate screening. | |

| **JBI Quality Appraisal** | | |
| --- | --- | --- |
| **Question** | **Judgement** | **Justification** |
| **1. Is the review questions clearly and explicitly stated?** | Unclear | This article states that the reviews published adherence studies on HIV-infected youth (ages 13 to 24 years), focusing on rates of adherence to antiretroviral regimens and interventions designed to enhance adherence. However, data collated summarises factors influencing adherence as well as interventions that enhance adherence. |
| **2. Were the inclusion criteria appropriate for the review question?** | No | Included were quantitative and qualitative studies reporting original data on medication adherence among HIV-infected youth (ages 13 to 24 years) and on exercising an intervention technique to enhance antiretroviral adherence among this population. Studies that included children as well as adolescents and young adults were incorporated for review as long as the mean age of participants fell within the 13- to 24-year-old age range. All relevant studies were included in the review, regardless of methodologic rigor. Authors did not specify country as part of the inclusion criteria. |
| **3. Was the search strategy appropriate?** | Unclear | Using combinations of the keywords HIV/AIDS, youth, adolescents, young adults, adherence (or compliance), non-adherence (or noncompliance), medical treatments, highly active antiretroviral therapy (HAART), anti-retroviral, resistance, and intervention (also keywords associated with specific types of interventions, such as education, telephone, and peer). However, the search does not seem comprehensive and there is no evidence of a search strategy for at least one database. |
| **4. Were the sources and resources used to search for studies adequate?** | No | Medline, PubMed, PsychInfo. In addition, bibliographies of relevant articles were reviewed for additional studies. They did not include grey literature. |
| **5. Was selection of studies done adequately?** | Unclear | Not reported |
| **6. Were the criteria for appraising studies appropriate?** | Unclear | Not reported |
| **7. Was the critical appraisal conducted by two or more reviewers independently?** | Unclear | Not reported |
| **8. Were there methods to minimize errors in the data extraction?** | Unclear | A coding manual was developed to extract descriptive information on setting, study design, population and sample characteristics, definition of adherence used, adherence measurement method, key study variables, and reported findings. The authors do not report on who conducted the extraction and whether this was conducted in duplicate. |
| **9. Were the methods used to combine studies appropriate?** | Unclear | For the qualitative papers, a combination of content analysis and an iterative process of variable sorting and concept formation common in qualitative research was employed to identify 9 categories in which all the variables could be classified. The analysis of quantitative studies is not reported. |
| **10. Were recommendations for policy and/or practice supported by the reported data?** | Yes | Detailed descriptions of the included studies are provided and support the recommendations made. |
| **11. Were the specific directives for new research appropriate?** | Yes | Not many research directives were provided but the directives reported are appropriate. |

*Santer*

| **Characteristics of included systematic review Santer et al. 2014** | | |
| --- | --- | --- |
| **Citation** | | |
| Santer, M. et al., 2014. Treatment non-adherence in paediatric long-term medical conditions: Systematic review and synthesis of qualitative studies of caregivers’ views. BMC Paediatrics, 14(1 PG-). Available at: https://www.scopus.com/inward/record.uri?eid=2-s2.0-84899486142&partnerID=40&md5=16144f05b226c744b60274a03e977597 NS  -. | | |
| **Search Summary Details** | | |
| **Literature Searched** | Medline, EMBASE, Cinahl, Psychinfo. Additional papers were sought by writing to authors and examining reference lists of included studies. | |
| **Search Dates** | 1996 to 2011 | **Date of last search: December 2011** |
| **Search Criteria and Outcomes** | | |
|  | **What the review authors searched for** | **What the review authors found** |
| **Studies** | Qualitative studies in English and German | 17 qualitative studies (19 papers) |
|  |  | **Studies relevant to this overview:** |
|  |  | 3 Studies: Qualitative studies (3) |
| **Participants** | Parents and caregivers of children aged 12 or younger with long term conditions such as asthma, diabetes | N=96 Caregivers of children aged 0 -18 years |
| **Issue** | Treatment adherence and Non adherence | Adherence (3) |
| **Setting** | Developed countries | Belgium (1) and US (2) |
| **Outcomes: Barriers and Facilitators** | Barriers to treatment adherence. | **Overview Framework:** Individual, Interpersonal, Community and Social; and Political and Health System. |
| **Systematic Review Methods** | | |
| **Conceptual framework** | Not reported | |
| **Data extraction method** | Standard data extraction form to capture study design and participants. Study findings and discussion were separately captured into Nvivo 9 | |
| **Appraisal tool used** | CASP Tool | |
| **Data synthesis method** | Thematic Synthesis by coding text, developing descriptive and general analytical themes. | |

| **JBI Quality Appraisal** | | |
| --- | --- | --- |
| **Question** | **Judgement** | **Justification** |
| **1. Is the review questions clearly and explicitly stated?** | Yes | We therefore conducted a systematic review and synthesis of the qualitative literature to investigate parents and caregivers' accounts of their reasons for adherence and non-adherence to prescribed treatments in paediatric long-term medical conditions |
| **2. Were the inclusion criteria appropriate for the review question?** | No | Included qualitative studies from the perspectives of parents and other caregivers of children with long-term conditions. Focused on caregiver adherence. Studies had to include data on parents or caregivers of children 12 years or younger. Studies only reporting on children older than 12 were excluded. Focused on clinical conditions with long-term outcomes for children and studies where caregivers were given specific treatment advice and instructions. The authors excluded studies from developing countries, as they believed barriers to treatment adherence would differ substantially in their context. Only German and English included, as these were the languages spoken by the authors. The study would have benefitted from including studies from developing countries and be more language inclusive. |
| **3. Was the search strategy appropriate?** | Unclear | Searches took place in December 2011 and included studies from 1996. Keywords have been indicated as well. The search strategy key terms are not comprehensive. Marked as unclear as the author mention that this is just one example but do not evidence others. |
| **4. Were the sources and resources used to search for studies adequate?** | Yes | Medline, EMBASE, Cinahl, Psychinfo. Additional papers were sought by writing to authors and examining reference lists of included papers. Titles and abstracts were initially screened and if these indicated that the paper might meet the inclusion criteria, the full text paper was retrieved and examined against the inclusion criteria. |
| **5. Was selection of studies done adequately?** | Unclear | Selection is reported but the method was not reported. Authors mention that if there was uncertainty it was discussed with the research team. |
| **6. Were the criteria for appraising studies appropriate?** | Yes | CASP quality assessment tool was used and reported within Tables of the paper. This was considered adequate. |
| **7. Was the critical appraisal conducted by two or more reviewers independently?** | Yes | Assessing potential papers against inclusion criteria and assessing quality was completed independently by two researchers and then collaboratively to compare findings. Any differences were resolved through discussion. |
| **8. Were there methods to minimize errors in the data extraction?** | Unclear | Data was extracted in two phases. First, information about the study design and participants were extracted into a previously adapted template. Second, findings and discussion from included papers relating to treatment adherence were captured into Nvivo9. Who conducted the extraction is not reported. |
| **9. Were the methods used to combine studies appropriate?** | Yes | Used the principles of thematic synthesis, an established approach previously used in public health. In order to develop descriptive themes, three reviewers independently coded this text in 10 original papers – these were chosen as they covered different conditions and provided a breadth of findings – to identify provisional themes according to meaning and content. These three reviewers then discussed their independently derived themes and agreed a preliminary coding frame of main themes. This coding frame was then applied to data in all papers. Two reviewers with any differences between coders resolved through discussion and the coding frame refined where necessary coded data independently. This process involved extensive team discussion and reflection to refine descriptive themes and develop overarching analytical themes derived from all included studies. |
| **10. Were recommendations for policy and/or practice supported by the reported data?** | Yes | Such findings can inform everyday consultations in paediatric long-term conditions. The additional complexity in the paediatric encounter of the ‘therapeutic triad’ rather than a ‘therapeutic dyad’ represents a challenge to health professionals to develop sophisticated communication strategies. For instance, health professionals may be able to assist parents and caregivers by helping the child view their treatment as enabling health and a 'normal life’, rather than representing ill ness and interference. Participants in these studies wished for more support from health professionals in devising simpler treatment regimens that take account of family life, seeking solutions to barriers to adherence and communicating with their child about adherence. Providing opportunities to discuss barriers to adherence before repetitive resistance develops could be a great help to caregivers. This is appropriate. |
| **11. Were the specific directives for new research appropriate?** | No | Authors suggest that a synthesis of qualitative studies focusing on the views of children and young people with long-term conditions would be therefore valuable in future. Directives are not very specific. Authors did not mention directives such as including developing countries, differences between the diseases. |

*Vervoort*

| **Characteristics of included systematic review Vervoort et al. 2007** | | | |
| --- | --- | --- | --- |
| **Citation** | | | |
| Vervoort, S.C.J.M. et al., 2007. Adherence in antiretroviral therapy: A review of qualitative studies. Aids, 21(PG-271-281), pp.271–281. Available at: NS  -. | | | |
| **Search Summary Details** | | | |
| **Literature Searched** | CINAHL, PUBMED and Web of Science | | |
| **Search Dates** | 1996 to 2005 | | **Date of last search: Not specified** |
| **Search Criteria and Outcomes** | | | |
|  | **What the review authors searched for** | | **What the review authors found** |
| **Studies** | Qualitative and quantitative studies but only qualitative data was used. English language. | | 24 Studies: All studies contained qualitative data was extracted (methods of larger studies not reported) |
|  |  |  | **Studies relevant to this overview:** |
|  |  |  | *24 Studies containing qualitative data.* |
| **Participants** | Patients living with HIV | | *Author has been contacted for supporting information:* N=1053 Adult PLHIV |
| **Issue** | Patient perspectives, barriers, facilitators and the process of adherence to HAART | | Adherence (24) |
| **Setting** | Western (unclear) | | *Author has been contacted for supporting information:* Not specified |
| **Outcomes: Barriers and Facilitators** | Therapy related, condition related, patient related, health care team and system related and socioeconomic factors | | **Overview Framework:** Individual, Interpersonal, Community and Social, Political and Health System, and Structural Factors |
| **Systematic Review Methods** | | | |
| **Conceptual framework** | | WHO guide on factors affecting adherence | |
| **Data extraction method** | | Extraction was done by one author and checked by another author. This was adequate. | |
| **Appraisal tool used** | | Identified and used specific criteria for review of methodological quality | |
| **Data synthesis method** | | Thematic content analysis | |

| **JBI Quality Appraisal** | | |
| --- | --- | --- |
| **Question** | **Judgement** | **Justification** |
| **1. Is the review questions clearly and explicitly stated?** | No | The PICO elements are identified in the methods section of the paper, however there is no clearly stated question or aim. |
| **2. Were the inclusion criteria appropriate for the review question?** | Yes | Qualitative studies published from 1996 through April of 2005 were selected for this review if they focused on the patients’ perspectives, barriers, facilitators and the process of adherence to HAART. The search was restricted to articles written in English. According to Table 1 and reasons for exclusions were quantitative only studies, children and adolescents, non-English, non-Western and non-antiretroviral adherence. |
| **3. Was the search strategy appropriate?** | Yes | Search terms provided. 1996 through April 2005. Articles restricted to English. Table 1 provides summary if search strategies per database, number of hits, reasons for exclusions and references for included studies. |
| **4. Were the sources and resources used to search for studies adequate?** | No | CINAHL, PUBMED and Web of Science. No grey literature included. |
| **5. Was selection of studies done adequately?** | Unclear | Not reported. |
| **6. Were the criteria for appraising studies appropriate?** | Unclear | Appraisal considered the nature of the sample, recruitment strategy, the population and the sample size. The quality of data collection was appraised for measures taken to assure validity, quality of the data collector (interviewer), interview type, data triangulation and the likely thickness of the data (i.e., whether enough data had been collected to support the conclusions, as can be inferred from the interview guide and the number and duration of the interviews). |
| **7. Was the critical appraisal conducted by two or more reviewers independently?** | Unclear | Two reviewers for methodological quality evaluated studies meeting the inclusion criteria. Not reported whether this was done independently or in duplicate. |
| **8. Were there methods to minimize errors in the data extraction?** | Yes | Extraction was done by one author and checked by another author. This was adequate. |
| **9. Were the methods used to combine studies appropriate?** | Yes | The included publications were read several times. Findings were coded inductively and interpreted after which articles were organised into thematic groups and compared within these groups. The process is reported to be done by the first author and controlled by the second author. Discrepancies were discussed by consensus. A grounded theory approach was used. |
| **10. Were recommendations for policy and/or practice supported by the reported data?** | Yes | Specific practice recommendations are made within each of the categories of analysis including therapy-related factors, condition-related factors, patient-related factors, healthcare team and system-related factors and socioeconomic factors. |
| **11. Were the specific directives for new research appropriate?** | Unclear | Further qualitative studies can make an important contribution in this field, particularly when the research approaches deal with the respondents’ own perspective. Such methods are essential given the complexity of adherence. This is very vague. |

*Vitalis*

| **Characteristics of included systematic review Vitalis et al. 2013** | | |
| --- | --- | --- |
| **Citation** | | |
| Vitalis. Factors affecting antiretroviral therapy adherence among HIV-positive pregnant and postpartum women: An adapted systematic review. Int J STD AIDS [Internet]. 2013;24(6):427–32. Available from: http://www.embase.com/search/results?subaction=viewrecord&from=export&id=L370229543%5Cnhttp://dx.doi.org/10.1177/0956462412472807%5Cnhttp://sfx.library.uu.nl/utrecht?sid=EMBASE&issn=09564624&id=doi:10.1177/0956462412472807&atitle=Factors+affecting+antiret | | |
| **Search Summary Details** | | |
| **Literature Searched** | Ovid Medline, Ovid Embase, Ovid PsycInfo, Ebsco Cinahl Plus, JSTOR, ISI Web of Science and the Cochrane Library | |
| **Search Dates** | Up to July 2011 | **Date of last search: 7 July 2011** |
| **Search Criteria and Outcomes** | | |
|  | **What the review authors searched for** | **What the review authors found** |
| **Studies** | Quantitative and qualitative studies. English only studies. No date limits. | 18 studies: 15 Quantitative and 3 Qualitative |
|  |  | **Studies relevant to this overview:** |
|  |  | *Author has been contacted for supporting information: 18 studies: 15 Quantitative and 3 Qualitative* |
| **Participants** | Hiv positive pregnant and post partum women receiving ART | HIV positive pregnant and post-partum women between the ages of 12 to 58 years receiving ART. |
| **Issue** | Factors affecting antiretroviral therapy | Adherence (18) |
| **Setting** | Resource rich and resource constrained countries, with a focus on the latter | USA (8), Africa (7), Australia (1), Brazil (2) and Puerto Rico (1) |
| **Outcomes: Barriers and Facilitators** | Individual factors, relationship and social factors, stigma, interpersonal violence, health system factors | **Overview Framework:** Individual, Interpersonal, Community and Social, Political and Health System, and Structural Factors |
| **Systematic Review Methods** | | |
| **Conceptual framework** | No conceptual framework specified | |
| **Data extraction method** | Used a modified data collection form designed using items from Table 7.3.an of the Cochrane Handbook Version 5.1.0.33 | |
| **Appraisal tool used** | CASP | |
| **Data synthesis method** | Content Analysis | |

| **JBI Quality Appraisal** | | |
| --- | --- | --- |
| **Question** | **Judgement** | **Justification** |
| **1. Is the review questions clearly and explicitly stated?** | Yes | To conduct a systematic review of the literature to determine the studies that address the risk factors and determinants of Adherence in seropositive pregnant and postpartum women in international settings. |
| **2. Were the inclusion criteria appropriate for the review question?** | Yes | A systematic literature search was conducted for both qualitative and quantitative studies in both resource-rich and resource constrained countries, with a focus on the latter settings. All designs (qualitative and quantitative), Pregnant and postpartum women with HIV, and the barriers and facilitators to adherence to ART. |
| **3. Was the search strategy appropriate?** | Unclear | Keywords provided. Searches from 1806-2011. There were no date limits on the searches, but studies were limited to those written in English which included the study population |
| **4. Were the sources and resources used to search for studies adequate?** | No | Ovid Medline, Ovid Embase, Ovid PsycInfo, EBSCO CINHAL Plus, JSTOR, ISI Web of Science and the Cochrane Library. Reference list of selected studies were hand searched. Grey literature was not included. |
| **5. Was selection of studies done adequately?** | Unclear | Abstracts identified from the PICO was reviewed but the authors do not indicate whether this was done independently or in duplicate. |
| **6. Were the criteria for appraising studies appropriate?** | Yes | The qualitative studies were assessed using the Critical Appraisal Skills Program (CASP), a 10-item tool for qualitative research and the Effective Public Health Practice Project for quantitative studies. |
| **7. Was the critical appraisal conducted by two or more reviewers independently?** | Unclear | Not reported. |
| **8. Were there methods to minimize errors in the data extraction?** | Unclear | Data were extracted using a modified data collection form designed using items from Table 7.3.a of the Cochrane Handbook Version 5.1.0.33. Key information (author, year, participants, methods, interventions, outcomes and results) from each study was summarized on this form. Predetermined but whether this was independently or in duplicate is not reported. |
| **9. Were the methods used to combine studies appropriate?** | Unclear | Not reported. |
| **10. Were recommendations for policy and/or practice supported by the reported data?** | No | Although interventions to improve adherence in pregnant and postpartum women are non-existent at this time, strategies undertaken in the general HIV/AIDS population can be modified or use in this population. Broad recommendations. |
| **11. Were the specific directives for new research appropriate?** | Yes | The paucity of information on adherence among pregnant and postpartum women, particularly in resource-constrained countries, warrants urgent attention. As major funding sources such as the Global Fund, World Bank and USAID scale back on financial resources for this epidemic, targeted research is needed on the facilitators and barriers to adherence among this group that not only measures adherence, but also research that can provide insight on the myriad reasons why women fail to adhere. Based on the thin description of the evidence the broad recommendations are sufficient for research directives. |

*Wasti*

| **Characteristics of included systematic review Wasti et al. 2012** | | |
| --- | --- | --- |
| **Citation** | | |
| Wasti, S.P. et al., 2012. Factors influencing adherence to antiretroviral treatment in Asian developing countries: a systematic review. Trop Med Int Health, 17(1 PG-71-81), pp.71–81. Available at: NS | | |
| **Search Summary Details** | | |
| **Literature Searched** | Medline/Ovid, Cochrane library, CINAHL, Scopus, Psych INFO, English articles | |
| **Search Dates** | 1996-2010 | **Date of last search: Not specified** |
| **Search Criteria and Outcomes** | | |
|  | **What the review authors searched for** | **What the review authors found** |
| **Studies** | Quantitative, qualitative and mixed methods studies. English only. Published studies. | 18 studies: 12 Quantitative, 4 Qualitative and 2 Mixed Methods |
|  |  | **Studies relevant to this overview:** |
|  |  | 18 Studies: 12 Quantitative, 4 Qualitative and 2 Mixed Methods |
| **Participants** | Adult PLHIV who have been prescribed ART. | N=4782 Adult PLHIV who have been prescribed ART. Quantitative Studies n=4372; qualitative studies n=152 and mixed methods studies n=258 |
| **Issue** | Adherence to ART | Positive and negative factors affecting adherence |
| **Setting** | 24 Asian developing countries as defined by the World Bank (2010) | India (10), China (4), Thailand (3), Cambodia (1). |
| **Outcomes: Barriers** | Factors influencing adherence to ART | **Overview Framework:** Individual, Interpersonal, Community and Social, Political and Health System, and Structural Factors |
| **Systematic Review Methods** | | |
| **Conceptual framework** | No conceptual framework specified | |
| **Data extraction method** | Used a standardised form. Data extraction was double-checked and if necessary amendments were made. | |
| **Appraisal tool used** | Tool by Hawker et al (2002) which is validated for assessing methodological quality of quantitative and qualitative studies. | |
| **Data synthesis method** | Thematic synthesis was performed. | |

| **JBI Quality Appraisal** | | |
| --- | --- | --- |
| **Question** | **Judgement** | **Justification** |
| **1. Is the review questions clearly and explicitly stated?** | No | Reviewed published articles on factors affecting adherence to ART in Asia. The population is later specified in the inclusion/exclusion criteria but the question is not clearly defined. |
| **2. Were the inclusion criteria appropriate for the review question?** | No | The population consisted of participants >18 years who had been prescribed ART. Data describing ART service providers were included to provide the staff’s perspective. The included studies considered populations from 24 Asian developing countries as defined by the World Bank (2010). Papers not written in English, published before 1996, review articles, policy documents and adherence training manuals were excluded. The inclusion is specific to Asia, therefore non-English papers should have been included. |
| **3. Was the search strategy appropriate?** | Yes | Articles sourced from 1996 through to December 2010. Keywords have been indicated. Reference mining has also been completed. English only studies. Keywords are not comprehensive and no evidence of the search strategy is available. |
| **4. Were the sources and resources used to search for studies adequate?** | No | Medline/Ovid, Cochrane library, CINAHL, Scopus, PsychInfo. Did not include grey literature. Screened the reference list of included studies. |
| **5. Was selection of studies done adequately?** | Unclear | Two authors independently reviewed and retrieved studies at abstract and title level. No information reported on who conducted full text screening. |
| **6. Were the criteria for appraising studies appropriate?** | Unclear | The appraisal tool used was developed by Hawker et al. (2002) whose tool is validated for both quantitative and qualitative systematic reviews in health care settings. The tool is indicated to have 9 questions and the scoring system is provided but the domains of the tool are not available. |
| **7. Was the critical appraisal conducted by two or more reviewers independently?** | Unclear | Not reported |
| **8. Were there methods to minimize errors in the data extraction?** | Yes | Used a standardised form. Data extraction was double-checked and if necessary amendments were made. |
| **9. Were the methods used to combine studies appropriate?** | No | Owing to the heterogeneity of the data (quantitative and qualitative), meta-analysis was not appropriate and a thematic synthesis was performed instead and results are presented in table form. The analysis used resembles content analysis however, the authors report it as thematic, therefore No |
| **10. Were recommendations for policy and/or practice supported by the reported data?** | No | Recommendations are broad and only partially supported by data as the findings were not analysed for depth and little application to theory has been made. Authors report that drawing coherent conclusions was hampered by the scarce data and methodological limitations of included studies. Recommendations were too broad and not supported by data. |
| **11. Were the specific directives for new research appropriate?** | Unclear | Not reported |

*Williams*

| **Characteristics of included systematic review Wiiliams et al. 2017** | | |
| --- | --- | --- |
| **Citation** | | |
| Williams S, Renju J, Ghilardi L, Wringe A. Scaling a waterfall: A meta-ethnography of adolescent progression through the stages of HIV care in sub-Saharan Africa. J Int AIDS Soc [Internet]. 2017;20(1):1–17. Available from: https://doi.org/10.7448/IAS.20.1.21922 | | |
| **Search Summary Details** | | |
| **Literature Searched** | PubMed, Web of Science, Scopus, Global Health and ADOLEC. | |
| **Search Dates** | January 2005 to March 2016 | **Date of last search: Not specified.** |
| **Search Criteria and Outcomes** | | |
|  | **What the review authors searched for** | **What the review authors found relevant to this overview** |
| **Studies** | Qualitative studies published in English. | 24 Studies: Qualitative (24) |
|  |  | **Studies relevant to this overview:** |
|  |  | 18 Studies: Qualitative (18) |
| **Participants** | Adolescents LHIV (ages 10-19 year) | Adolescent ages 9-20 LHIV |
| **Issue** | Testing, Linkage, adherence to ART and retention | Linkage (8), Adherence (15) and Retention (10) from 18 studies. |
| **Setting** | sub-Saharan Africa | Zimbabwe (2), South Africa (3), Kenya (3), Botswana (1), Zambia (3), Tanzania (1), Uganda (1), Uganda and Zimbabwe (1), Tanzania (2), and Botswana and Tanzania (1) |
| **Outcome: Barriers and Facilitators** | Factors influencing engagement with cascade of care. | **Overview Framework**: Individual, Interpersonal, Community and Social, Political and Health System, and Structural Factors. |
| **Systematic Review Methods** | | |
| **Conceptual framework** | Socio-ecological model (Individual, Family/Peer, Community and Structural) | |
| **Data extraction method** | Not reported. | |
| **Appraisal tool used** | Oxford CASP | |
| **Data synthesis method** | Meta-ethnography | |

| **JBI Quality Appraisal** | | |
| --- | --- | --- |
| **Question** | **Judgement** | **Justification** |
| **1. Is the review questions clearly and explicitly stated?** | Yes | Meta-ethnography includes qualitative studies undertaken across the region, in order to understand the most influential issues affecting adolescent initiation of, and retention in, HIV care in SSA. |
| **2. Were the inclusion criteria appropriate for the review question?** | Yes | Studies were limited to adolescent’s age 10-19 years, qualitative studies and in sub-Saharan Africa. Published in English. |
| **3. Was the search strategy appropriate?** | Unclear | The key terms are provided but there is no evidence of the search strategy. Only English studies |
| **4. Were the sources and resources used to search for studies adequate?** | No | PubMed, Web of Science, Scopus, Global Health and ADOLEC. Did not include grey literature. |
| **5. Was selection of studies done adequately?** | Unclear | Not reported how studies were selected and who was involved |
| **6. Were the criteria for appraising studies appropriate?** | Yes | Oxford CASP was used and is appropriate. Description of the scoring is provided. |
| **7. Was the critical appraisal conducted by two or more reviewers independently?** | Yes | Appraisal was conducted independently and in duplicate |
| **8. Were there methods to minimize errors in the data extraction?** | Unclear | Not reported how data was extracted and who was involved |
| **9. Were the methods used to combine studies appropriate?** | Yes | Meta-ethnographic analysis was reported to be used to synthesise the results. Method is described and how it was conducted. Did not state who conducted the analysis. Quotes are used to support the themes that emerged. |
| **10. Were recommendations for policy and/or practice supported by the reported data?** | Yes | The recommendations are based on findings. Reference is made to the data and conclusions are appropriate. |
| **11. Were the specific directives for new research appropriate?** | Yes | The authors identified the gaps of gender specific experiences for adolescents and the lack of research on risk perceptions, knowledge of HIV transmission, sources of SRH education and experiences of adolescent females with HIV services. All of these are discussed in the data included in the paper. |
