# Supplementary material for: A mega-aggregation framework synthesis of the barriers and facilitators to linkage, adherence to ART and retention in care among people living with HIV
Source: Syst Rev. 2021 Feb 11;10:54. doi: 10.1186/s13643-021-01582-z (PMC7875685; doi:10.1186/s13643-021-01582-z)
Supplement: Supplementary file 9 — Additional file 9. Overlap of included primary studies (n = 826) [file 13643_2021_1582_MOESM9_ESM.docx]

**Additional file 9: Overlap of included primary studies (n=826)**

Overlap of included primary studies (n=826) within included systematic reviews (n=33)

| **Primary studies included in reviews**  **(First author, Year of publication)** | **Country** | **Sample size** | **Total number of reviews including the primary study** |
| --- | --- | --- | --- |
|  |  |  |  |
| Abaasa, 2008 | Columbia | 897 | 1 |
| Abah, 2014 | South Africa | 588 | 1 |
| Abdelrahman, 2013 | Uganda |  | 2 |
| Abel, 2003 | Egypt | 11 | 3 |
| Aboubacrine, 2007 | USA | 270 | 1 |
| Abrahams, 2010 | Burkina Faso and Mali | 29 | 1 |
| Adam, 2003 | South Africa | 35 | 3 |
| Adamian, 2004 | Canada | 20 | 1 |
| Addala, 2010 | USA | 13163 | 1 |
| Addo-Atuah, 2012 |  | 20 | 2 |
| Adedimeji, 2011 | Ghana | 50 | 1 |
| Adewuya, 2010 | Zambia | 182 | 1 |
| Adeyemo, 2009 | Nigeria | 127 | 1 |
| Afolabi, 2009 | Nigeria | 120 | 1 |
| Agbonyitor, 2009 | Nigeria | 30 | 2 |
| Ahsan Ullah, 2011 | Nigeria |  | 1 |
| Akello, 2011 | Bangladesh |  | 1 |
| Akhila, 2010 | South Africa | 313 | 1 |
| Alakija, 2010 | India | 253 | 1 |
| Albrecht, 2006 | Nigeria |  | 2 |
| Alemu, 2011 |  | 1722 | 2 |
| Alemu, 2013 | Ethiopia |  | 1 |
| Alfonso, 2006 | South Africa |  | 3 |
| Alfonso, 2009 |  | 12 | 2 |
| Allen, 2011 | Canada | 41 | 1 |
| Alliance, 2004 | Uganda |  | 1 |
| Amankwah, 2015 | Zambia | 136 | 1 |
| Amberbir, 2008 | Ghana | 400 | 2 |
| Ammassari, 1200 | Ethiopia |  | 1 |
| Amolloh, 2011 |  |  | 1 |
| Amuron, 2009 | Tanzania | 2483 | 1 |
| Ankrah, 2016 | Uganda | 19 | 1 |
| Aransiola, 2014 | Nigeria | 15 | 1 |
| Arem, 2011 | Uganda |  | 1 |
| Arrivillaga 2011 | Columbia | 47 | 2 |
| Arrivillaga, 2012 | Nigeria |  | 1 |
| Asgary, 2014 | Ethiopia | 18 | 1 |
| Asiimwe, 2007 | Uganda | 84 | 1 |
| Aspeling, 2008 | South Africa | 11 | 3 |
| Assefa, 2010 | Ethiopia |  | 3 |
| Atanga, 2017 | Cameroon | 36 | 1 |
| Atuyambe, 2008 | Uganda | 32 | 1 |
| Audu, 2013 | Nigeria | 35 | 2 |
| Audu, 2014 | Nigeria | 35 | 2 |
| Auvinen, 2013 | Zambia |  | 1 |
| Aversa, 1996 | USA |  | 1 |
| Avong, 2015 | Nigeria | 502 | 1 |
| Awiti-Ujiji, 2011 |  | 26 | 2 |
| Axelsson, 2015 | Lesotho | 28 | 1 |
| Ayuo, 2013 |  | 4284 | 2 |
| Aziz, 2011 | Uganda | 40 | 1 |
| Badahdah, 2011 | Egypt | 27 | 2 |
| Baghazal, 2011 | Kenya | 27 | 1 |
| Bajunirwe, 2009 | Uganda | 175 | 3 |
| Balcha, 2011 | Ethiopia | 14 | 3 |
| Balira, 2010 | Tanzania | 244 | 1 |
| Bancheno, 2010 | Swaziland | 64 | 1 |
| Bardeguez, 2008 | USA | 519 | 3 |
| Barnett, 2013 | South Africa | 12 | 1 |
| Barr, 2013 |  | 19735 | 1 |
| Barton Laws, 2000 | USA | 25 | 1 |
| Bartos, 2010 | Australia | 76 | 1 |
| Bastard, 2011 | Senegal | 330 | 1 |
| Batchelder , 2013 | USA | 15 | 1 |
| Becker, 2002 | USA | 3788 | 1 |
| Beckham, 2009 | Tanzania | 68 | 1 |
| Beer, 2009 | USA | 37 | 2 |
| Beer, 2012 | USA | 47 | 1 |
| Begley, 2008 | Australia | 179 | 2 |
| Belenky, 2014 | Tanzania | 403 | 1 |
| Belzer, 1999 | USA | 31 | 1 |
| Benoit, 2013 | Kenya | 30 | 1 |
| Benotsch, 2008 |  |  | 1 |
| Benzekri, 2015 | Senegal | 109 | 1 |
| Bernays, 2009 |  |  | 1 |
| Bernays, 2010 | Uganda and Zimbabwe |  | 1 |
| Bernays, 2015 | Serbia | 20 | 1 |
| Beusterien, 2008 | USA | 35 | 4 |
| Beyene, 2009 | Ethiopia |  | 2 |
| Bezabhe, 2014 | Ethiopia | 58 | 1 |
| Bezabhe, 2014 | Ethiopia | 24 | 1 |
| Bezabhe, 2014 | Ethiopia | 58 | 1 |
| Bhagwanjee, 2008 | South Africa | 19 | 2 |
| Bhagwanjee, 2011 | South Africa | 33 | 2 |
| Bhat, 2010 | South Africa | 168 | 2 |
| Bhengu, 2011 | South Africa | 149 | 1 |
| Bhushan, 2015 | USA | 30 | 1 |
| Bianchi, 2010 | South Africa |  | 1 |
| Bii, 2007 | Kenya |  | 2 |
| Bila, 2009 | Burkina Faso |  | 1 |
| Birbeck, 2009 | Zambia | 255 | 1 |
| Birbeck, 2011 | Zambia | 496 | 1 |
| Blake, 2008 | USA | 23 | 2 |
| Boateng, 2013 | Ghana | 23 | 2 |
| Boehme AK, 2012 |  |  | 1 |
| Bogart, 2004 | USA | 181 | 1 |
| Bogart, 2010 | USA | 214 | 1 |
| Bogart, 2011 | USA | 315 | 1 |
| Bogart, 2013 | South Africa | 51 | 2 |
| Bohle, 2014 | Tanzania | 59 | 1 |
| Bontempi, 2004 |  |  | 1 |
| Bowden, 2006 | USA | 10 | 1 |
| Bowie, 2010 | Malawi | 1266 | 1 |
| Boyer, 2011 | Cameroon | 2381 | 1 |
| Braitstein, 2011 | Kenya | 97 | 1 |
| Braunstein, 2011 | Rwanda | 141 | 1 |
| Brigido | Brazil | 182 | 1 |
| Brinkley- Rubinstein, 2013 | South Africa | 171 | 1 |
| Brion, 2008 | USA | 24 | 4 |
| Brody, 2016 | USA | 22 | 1 |
| Brook, 2001 |  |  | 1 |
| Buesseler, 2014 | Ivory Coast | 29 | 1 |
| Bullo, 2009 |  | 291 | 1 |
| Buregyeya, 2017 | Uganda | 57 | 1 |
| Buseh, 2006 | USA | 20 | 1 |
| Busesh, 2008 | USA | 29 | 1 |
| Busza, 2014 | Tanzania |  | 1 |
| Butt, 2011 | Indonesia |  | 1 |
| Butt, 2013 | Indonesia |  | 1 |
| Butt, 2015a | Indonesia |  | 1 |
| Butt, 2015b | Indonesia |  | 1 |
| Bwirire, 2008 | Malawi | 25 | 2 |
| Byakika-Tusiime, 2005 | Uganda | 304 | 3 |
| Byakika-Tusiime, 2009 | Uganda | 15 | 2 |
| Byron, 2008 | Kenya | 79 | 2 |
| Campbell, 2011 | Zimbabwe | 48 | 2 |
| Campbell, 2015 | Zimbabwe |  | 1 |
| Carlucci, 2008 | Uganda | 424 | 1 |
| Cataldo, 2007 | Malawi | 24 | 1 |
| Catz, 2000 |  |  | 1 |
| Cauldbeck, 2009 | India | 60 | 1 |
| CDC, 2013 |  |  | 1 |
| Chabikuli 2010 | South Africa | 100 | 1 |
| Chakrapani, 2009 | India | 19 | 2 |
| Chakrapani, 2011 | India | 34 | 2 |
| Chan, 2011 | Malawi | 306 | 1 |
| Charurat, 2010 | Nigeria | 5760 | 1 |
| Chen, 2009 | China | 29 | 2 |
| Chen, 2013 | China | 29 | 1 |
| Chesney, 2000 |  |  | 1 |
| Chhagan, 2008 | South Africa | 21 | 1 |
| Chi, 2009 | Zambia | 27115 | 1 |
| Chileshe, 2010 | Zambia | 7 | 1 |
| Chinkonde, 2009 |  | 40 | 5 |
| Chomat, 2009 | India | 26 | 2 |
| Chow, 2012 | Australia | 188 | 2 |
| Church K, 2012 |  |  | 1 |
| Ciambrone, 2006 | Puerto Rico |  | 2 |
| Ciambrone, 2007 | Puerto Rico | 17 | 1 |
| Clark, 2008 | USA | 113 | 1 |
| Clouse, 2010 |  | 273 | 1 |
| Clouse, 2013 | South Africa | 50 | 1 |
| Clouse, 2014 | Zimbabwe and South Africa |  | 1 |
| Cluver, 2015 | Zambia | 684 | 2 |
| Cocohoba, 2013 | USA | 19 | 1 |
| Cohn, 2008 | USA | 149 | 3 |
| Comulada 2003 | USA | 253 | 1 |
| Cook, 2009 |  |  | 1 |
| Cooper, 2007 | South Africa | 61 | 1 |
| Coria, 2012 |  | 473 | 1 |
| Corneli | Uganda |  | 1 |
| Cowan, 2013 | Zimbabwe | 870 | 1 |
| Crane, 2006 | Uganda | 10 | 1 |
| Crankshaw, 2010 | South Africa | 300 | 1 |
| Crisp, 2004 | USA | 137 | 1 |
| Culbert, 2015 | Indonesia |  | 1 |
| Cummins, 2004 | Australia | 33 | 1 |
| Cupsa, 2000 |  |  | 1 |
| Curioso, 2010 | Peru | 31 | 1 |
| Curiosom, 2010 | Peru | 31 | 1 |
| Daftary, 2012 | South Africa | 40 | 2 |
| Daftary, 2013 | South Africa | 40 | 1 |
| Dahab, 2008 | South Africa | 6 | 5 |
| Dahab, 2011 | South Africa | 27 | 2 |
| Dale, 2014 | USA | 138 | 1 |
| Dalmida, 2010 | USA | 20 | 1 |
| Damar, 2010 | Indonesia |  | 1 |
| Dang, 2010 | USA | 22 | 2 |
| Dawson-Rose, 2016 | Mozambique | 57 | 1 |
| de la Hera, 2011 | Spain | 23 | 1 |
| de Sumari-de Boer, 2015 | Tanzania | 5 | 1 |
| de visser | Australia | 894 | 1 |
| Dean, 2012 |  | 7 | 2 |
| Delvaux, 2009 |  | 236 | 4 |
| Demas, 2005 |  |  | 2 |
| Demessie, 2014 | Ethiopia | 350 | 1 |
| Demoss, 2013 | USA | 12 | 1 |
| Denison, 2015 | Congo | 55 | 2 |
| Dewing, 2015 | South Africa | 600 | 1 |
| Diabaté, 2007 | Benin | 53 | 1 |
| Diabaté, 2011 | Benin | 747 | 1 |
| Diabaté, 2013 | Ivory Coast | 614 | 1 |
| Dilorio, 2009 | USA | 236 | 1 |
| Do, 2010 | Botswana | 300 | 2 |
| Dodds, 2003 | USA | 21 | 1 |
| Down, 2014 | Australia | 53 | 1 |
| Duff, 2010 | Uganda | 45 | 8 |
| Dutcher, 2011 |  |  | 1 |
| Duwell, 2013 | South Africa | 172 | 1 |
| Dyrehave, 2015 | Guinee Bissau | 494 | 1 |
| Ebuy, 2015 | Ethiopia | 227 | 1 |
| Edwards, 2006 | USA | 20 | 3 |
| Edwards, 2012 | USA | 20 | 1 |
| Eholie, 2007 | Ivory Coast | 308 | 2 |
| Eholie, 2009 | Ivory Coast | 308 | 1 |
| Ekama, 2012 | Nigeria | 170 | 2 |
| Ekwunife, 2012 | Nigeria | 212 | 1 |
| Eldred, 1998 |  |  | 1 |
| Elliott et al., 2011 | Cambodia | 27 | 1 |
| Elul, 2013 | Rwanda | 1417 | 2 |
| Elwell, 2015 | Malawi | 78 | 1 |
| Elwell, 2016 | Malawi | 78 | 2 |
| Enriquez, 2013 |  |  | 1 |
| Erah, 2008 | Nigeria | 125 | 1 |
| Erlen, 1999 |  | 6 | 1 |
| Erlen, 2002 |  |  | 1 |
| Esiru, 2013 |  | 350 | 1 |
| Essomba, 2015 | Cameroon | 524 | 1 |
| Etard, 2007 | Senegal | 158 | 1 |
| Etienne, 2010 | Kenya, Uganda, Zambia, Nigeria and Rwanda | 921 | 1 |
| Evans, 2009 |  |  | 1 |
| Eyassu, 2015 | South Africa | 290 | 1 |
| Ezekiel, 2009 | Tanzania | 189 | 1 |
| Ezzy, 2000 | Australia | 1320 | 1 |
| Fagbami, 2015 | USA | 18 | 1 |
| Falang, 2012 | Nigeria | 461 | 1 |
| Fassinou, 2004 | Ivory Coast |  | 1 |
| Ferguson, 2002 |  | 1129 | 1 |
| Ferguson, 2012 | Kenya | 19 | 2 |
| Ferguson, 2014 |  |  | 1 |
| Fetzer, 2011 | Zimbabwe | 40 | 2 |
| Feucht, 2007 | South Africa |  | 1 |
| Fielding-Miller, 2014 | Swaziland | 20 | 2 |
| Flax, 2017 | Malawi | 64 | 1 |
| Fletcher, 2006 | USA | 42 | 1 |
| Ford, 2010 | South Africa | 207 | 1 |
| Foster, 2010 | Uganda | 40 | 2 |
| Fox, 2010 | Zambia |  | 1 |
| Frank, 2009 | South Africa | 7 | 1 |
| Fredriksen-Goldsen, 2010 | China | 10 | 2 |
| Gachanja, 2016 | Kenya | 16 | 1 |
| Garang, 2009 | Uganda | 200 | 2 |
| Garland PM, 2011 |  |  | 1 |
| Georgette 2016 | South Africa | 100 | 1 |
| Georgeu, 2012 | South Africa | 1 | 1 |
| Giacomet, 2003 |  |  | 1 |
| Gibb, 2003 |  |  | 1 |
| Gilbert, 2007 | South Africa |  | 1 |
| Gilbert, 2009 | South Africa | 44 | 1 |
| Gilbert, 2010 | USA | 90 | 1 |
| Giles, 2009 | Australia | 45 | 1 |
| Gill, 2017 | Rwanda | 121 | 1 |
| Goar, 2015 | Nigeria | 160 | 1 |
| Gold, 2000 | Australia | 270 | 1 |
| Gold, 2001 | Australia | 20 | 1 |
| Goldman, 2008 | Zambia | 913 | 1 |
| Golin, 2002. | USA | 24 | 3 |
| Golooba, 2007 | South Africa | 27 | 1 |
| Golub, 2006 | USA | 42 | 2 |
| Goode, 2003 | Australia | 18 | 2 |
| Goudge, 2011 | South Africa | 22 | 3 |
| Gourlay, 2014 | Tanzania | 91 | 1 |
| Govender, 2015 | South Africa | 17 | 1 |
| Govindasamy, 2011 | South Africa |  | 1 |
| Graham, 2013 | Kenya | 68 | 1 |
| Granato, 2016 | Ivory Coast | 30 | 1 |
| Graney, 2003 | USA | 67 | 1 |
| Grant, 2008 | Zambia | 40 | 4 |
| Gray, 2006 | USA | 11 | 1 |
| Grierson, 2004 | Australia |  | 2 |
| Grierson, 20041 | Australia | 925 | 1 |
| Grierson, 2011 | Australia |  | 2 |
| Grierson, 2013 | Australia |  | 1 |
| Groft, 2007 |  |  | 1 |
| Groh, 2011 | Mozambique | 164 | 1 |
| Gross, 2015 | Zimbabwe | 262 | 1 |
| Guiro, 2011 | Burkina Faso | 412 | 1 |
| Gusdal, 2009a | Ethiopia |  | 1 |
| Gusdal, 2009b | Multi-country | 118 | 3 |
| Gusdal, 2011 |  | 79 | 1 |
| Guy, 2013 | Australia | 961 | 1 |
| Habib, 2009 | Nigeria | 58 | 1 |
| Habib, 2010 | Nigeria | 142 | 1 |
| Hammami, 2004 | Belgium | 11 | 2 |
| Han, 2009 | Thailand | 27 | 1 |
| Hardon, 2007 |  | 377 | 3 |
| Harzke, 2004a | USA | 137 | 1 |
| Harzke, 2004b | USA |  | 1 |
| Hatchett, 2004 | Malawi | 46 | 1 |
| Heckman, 2004 | USA |  | 1 |
| Hegazi, 2010 | Australia | 147 | 1 |
| Hermann, 2008 | Australia | 357 | 1 |
| Hermann, 2012a | Australia | 152 | 1 |
| Herrmann, 2012b |  | 22 | 2 |
| Hidayana, 2015 | Indonesia |  | 1 |
| Hill, 2003 |  | 78 | 2 |
| Ho, 2010 | China |  | 2 |
| Hodgson, 2012 | Zambia |  | 3 |
| Hofer, 2004 | Rio de Jeneiro |  | 1 |
| Holtzman, 2015 | USA | 51 | 1 |
| Hong, 2014 | Namibia | 390 | 1 |
| Horne, 2004 | France, Germany, Italy, Spain, UK, US | 111 | 1 |
| Horne, 2008 |  |  | 1 |
| Horwood, 2010 | USA | 312 | 1 |
| Hosek, 2005 | USA | 42 | 1 |
| Huntington, 2011 |  |  | 1 |
| Hussen, 2014 | Ethiopia | 20 | 1 |
| Ibrahim, 2011 | Indonesia |  | 2 |
| Ickovics, 2002 |  |  | 2 |
| Idindili, 2012 | Tanzania | 316 | 1 |
| Imelda, 2014 | Indonesia |  | 1 |
| Izugbara, 2011 |  |  | 2 |
| Jaquet, 2010 | Mali | 2920 | 2 |
| Jasserson, 2013 | France | 2952 | 1 |
| Jeneke, 2011 | South Africa | 40 | 1 |
| Jerome, 2011 |  | 18 | 2 |
| Joglekar., 2011 | India | 32 | 1 |
| Johansen, 2012 |  |  | 1 |
| Johnson, 2003 |  |  | 1 |
| Johnston-Roberts, 2000 | USA | 20 | 1 |
| Johnston-Roberts, 2002 | USA | 28 | 1 |
| Johnston-Roberts, 2003 | USA | 38 | 1 |
| Jones, 2009 | South Africa | 35 | 2 |
| Jones, 2011 | Zambia | 160 | 1 |
| Jones, 2012 | South Africa | 50 | 1 |
| Jones, 2014 | Zambia | 446 | 1 |
| Joseph, 2011 |  |  | 1 |
| Kagee, 2012 | South Africa | 10 | 1 |
| Kalanzi, 2009 | Uganda |  | 1 |
| Kalichman, 1999 | USA | 343 | 1 |
| Kalichman, 2010 | USA |  | 1 |
| Kamau, 2012 | Kenya | 354 | 1 |
| Kanjipite, 2012 | Zambia | 2759 | 1 |
| Ka'opua, 2004 | USA | 80 | 1 |
| Karanja, 2013 | Kenya | 22 | 1 |
| Kasenga, 2010 |  |  | 3 |
| Kastner, 2014 | Uganda | 25 | 1 |
| Katirayi, 2016a | Malawi | 132 | 3 |
| Katiyari, 2016b | Swaziland | 132 | 1 |
| Kawuma, 2014 | Zimbabwe | 26 | 1 |
| Kebede, 2012 | Ethiopia | 296 | 1 |
| Kekwaletswe, 2014 | South Africa | 304 | 2 |
| Kempf, 2010 | USA |  | 1 |
| Kemppainen, 2001 | USA | 46 | 1 |
| Kemppainen, 2004 | USA | 46 | 2 |
| Kerr, 1999 |  |  | 1 |
| Ketema, 2015 | Ethiopia | 422 | 1 |
| Kgatlwane, 2006 | Botswana | 249 | 1 |
| Khalid, 2012 | Tanzania | 15 | 1 |
| Kiarie, 2003 | Kenya |  | 1 |
| Kibicho, 2011 | USA | 19 | 1 |
| Kidia, 2015 | Zimbabwe | 47 | 1 |
| Killam, 2010 |  | 37203 | 1 |
| Kim, 2012 | Malawi | 1688 | 1 |
| Kim, 2016 | Malawi | 65 | 3 |
| Kimmel, 2012 | South Africa | 12 | 1 |
| Kingori, 2012 | Kenya | 370 | 1 |
| Kip, 2009 | Botswana | 400 | 3 |
| Kirsten, 2011 |  | 122 | 5 |
| Kisenyi, 2013 | Uganda | 220 | 1 |
| Kleeberger, 2001 |  |  | 1 |
| Klitzman, 2004 | USA | 152 | 2 |
| Knowlton, 2001 | USA | 295 | 1 |
| Knowlton, 2005 | USA | 287 | 1 |
| Kohler, 2012 | Kenya | 615 | 1 |
| Kohler, 2014 | Kenya | 213 | 1 |
| Konate, 2011 | Burkina Faso | 658 | 1 |
| Konkle-Parker , 2008 |  |  | 2 |
| Koole, 2016 | Kenya, Uganda and Zambia | 4425 | 1 |
| Kourrouski, 2009 | Brazil | 9 | 1 |
| Krebs, 2008 | Zambia | 430 | 1 |
| Kreitchmann, 2012 | Latin America | 393 | 1 |
| Kremer, 2006 | USA | 79 | 1 |
| Kremer, 2009 | USA | 79 | 1 |
| Krummenacher, 2014 | Switzerland | 17 | 2 |
| Kumarasamy, 2005 | India | 60 | 2 |
| Kunapareddy, 2014 | Kenya | 23 | 1 |
| Kunapareddy, 2015 | Kenya |  | 1 |
| Kunihira, 2010 | Uganda |  | 1 |
| Kunutsor, 2010 | Uganda | 967 | 2 |
| Kuonza, 2010 |  | 212 | 2 |
| Kuteesa, 2012 | Uganda | 40 | 1 |
| Kyajja, 2010 | Uganda | 166 | 1 |
| Laine, 2000 |  |  | 2 |
| Laniènce, 2003 |  | 158 | 1 |
| Lawan, 2012 | Nigeria | 124 | 1 |
| Laws, 2012 | USA | 46 | 1 |
| Laws, 2000 | USA | 82 | 3 |
| Lekhuleni, 2013 | South Africa | 25 | 1 |
| Lencha, 2015 | Ethiopia | 239 | 1 |
| Letta, 2015 | Ethiopia | 626 | 1 |
| Levy, 2009 | Malawi |  | 1 |
| Li, 2010 | Thailand | 507 | 2 |
| Lifson, 2013 | Ethiopia | 21 | 1 |
| Lowenthal, 2014 | Botswana |  | 1 |
| Lubega, 2010 | Uganda | 74 | 1 |
| Lubega, 2010 | Uganda | 1 | 3 |
| Lubinga, 2012 | Uganda | 334 | 1 |
| Luseno, 2008 | South Africa |  | 1 |
| Lyimo, 2012 | Tanzania | 61 | 3 |
| Lyimo, 2014 | Tanzania | 158 | 1 |
| MacLachlan, 2016 | Namibia | 10 | 1 |
| Maixenchs, 2015 | Mozambique | 51 | 1 |
| Makua, 2015 | South Africa | 18 | 1 |
| Malangu, 2008 | South Africa | 180 | 2 |
| Malcolm, 2000 | USA | 44 | 1 |
| Malcolm, 2003 |  | 44 | 1 |
| Mandala, 2009 |  | 14815 | 1 |
| Maqutu, 2010 | South Africa | 688 | 1 |
| Maqutu, 2011 | South Africa | 688 | 1 |
| Marcellin, 2008 |  | 533 | 1 |
| Marhefka, 2004 |  |  | 1 |
| Markos, 2008 | Ethiopia | 286 | 1 |
| Markos, 2009 | Ethiopia | 291 | 1 |
| Martin, 2011 | Uganda | 20 | 1 |
| Martin, 2013 | Latin America and Caribbean | 1 | 1 |
| Martin, 2013 | Uganda | 20 | 1 |
| Martinez, 2000 | USA | 25 | 1 |
| Masquillier, 2015 | South Africa | 32 | 1 |
| Matovu, 2012 | Uganda |  | 2 |
| Mattes, 2014 | Tanzania | 24 | 2 |
| Mavhu, 2013 | Zimbabwe |  | 2 |
| Mavhu, 2013 | Zimbabwe | 229 | 1 |
| Mawar, 2007 | India | 31 | 1 |
| Mayanja, 2013 | Uganda | 315 | 1 |
| Mbonye, 2013 | Uganda | 24 | 3 |
| Mbonye, 2014 | Uganda | 40 | 1 |
| Mbopi-Kéou, 2012 | Cameroon | 356 | 1 |
| Mbuagbaw, 2012 | Cameroon | 30 | 2 |
| Mburu, 2014 | Zambia |  | 1 |
| McAllister, 2013 | Australia | 335 | 2 |
| McCoy, 2009 | USA |  | 1 |
| McDonald, 2001 | Australia | 16 | 1 |
| McDonald, 2011 | Australia | 13 | 6 |
| McDonaldm 2010 | Australia | 1001 | 1 |
| McDoom, 2015 | USA | 20 | 1 |
| McGuire, 2010 | Malawi | 221 | 2 |
| McKinney, 2014 | Malawi | 8 | 1 |
| McLean, 2017 | Tanzania, Uganda and Malawi | 22 | 1 |
| Medley, 2014 |  | 353 | 1 |
| Melchior, 2007 | Brazil |  | 1 |
| Mellins, 2008 |  | 396 | 4 |
| Memiah, 2013 |  | 234 | 1 |
| Mendelsohn, 2014 | Kenya | 12 | 1 |
| Mepham, 2011 | South Africa | 199 | 4 |
| Merati, 2005 | Indonesia |  | 1 |
| Merzel, 2008 | USA | 14 | 1 |
| Meystre-Agustoni, 2000 | Switzerland | 37 | 1 |
| Mfecane, 2011 | South Africa | 25 | 1 |
| Midtbo, 2012 | Botswana and Tanzania |  | 2 |
| Mill, 2009 | Canada |  | 1 |
| Miller, 2002 | USA | 30 | 1 |
| Miller, 2010 | South Africa | 14 | 1 |
| Miller, 2012 | Malawi | 24 | 1 |
| Milloy, 2012 |  |  | 1 |
| Mimiaga, 2010 | Ukraine | 16 | 1 |
| Mirkuzie, 2010 |  | 282 | 1 |
| Mirkuzie, 2011 |  | 663603 | 1 |
| Misener, 1998 | USA | 22 | 1 |
| Mitchell, 2007 | South Africa |  | 1 |
| Mitiku, 2013 | Ethiopia | 239 | 1 |
| Mkandawire-Valhmu, 2012 | Kenya and Malawi | 180 | 1 |
| Moatti, 2000 |  |  | 1 |
| Mohammadpour, 2010 |  |  | 2 |
| Mohammed, 2004 | USA |  | 1 |
| Moiloa, 2012 | South Africa | 24 | 1 |
| Molassiotis, 2002 |  |  | 1 |
| Moneyham, 2010 | USA |  | 1 |
| Monreal, 2002 | Brazil |  | 1 |
| Monroe, 2013 | USA | 35 | 1 |
| Montoya, 2014 | USA | 20 | 1 |
| Moremi, 2012 | South Africa | 20 | 1 |
| Morojele, 2014 | South Africa | 304 | 1 |
| Mostashari, 1998 |  |  | 1 |
| Mouala, 2006 |  |  | 1 |
| Mshana, 2006 | Tanzania | 170 | 2 |
| Mtetwa, 2013 | Zimbabwe | 38 | 1 |
| Muchedzi, 2010 |  | 147 | 3 |
| Muessig, 2015 | USA | 56 | 1 |
| Muhamadi, 2010 | Uganda | 20 | 2 |
| Muma, 1995 |  |  | 1 |
| Mûnene, 2014 | Kenya | 392 | 1 |
| Munseri, 2008 | Tanzania | 8 | 1 |
| Mupambireyi, 2014 | Zimbabwe |  | 1 |
| Murphy, 2000 | USA | 231 | 3 |
| Murphy, 2001 | USA | 161 | 1 |
| Murphy, 2003 | USA | 39 | 4 |
| Murphy, 2005 | USA | 74 | 1 |
| Murray, 2009 | Zambia | 47 | 6 |
| Musheke, 2012 | Zambia | 25 | 1 |
| Musheke, 2013 (a) | Zambia | 37 | 1 |
| Musheke, 2013 (b) | Zambia | 62 | 1 |
| Musumari, 2013 | Democratic Republic of Congo | 38 | 4 |
| Musumari, 2014 | Ivory Coast | 38 | 3 |
| Mutabazi-Mwesigire (2014) [70] | Uganda | 20 | 1 |
| Mutchler, 2011 | USA | 25 | 2 |
| Mutithi, 2015 | Kenya | 12 | 1 |
| Mutumba, 2015 | Uganda | 38 | 2 |
| Mutwa, 2013 | Rwanda | 42 | 2 |
| Muya, 2014 | Tanzania | 4424 | 1 |
| Muyingo, 2008 | Uganda and Zimbabwe | 2957 | 1 |
| Myer, 2012 (a) |  | 221 | 2 |
| Myer, 2012 (b) |  | 490 | 1 |
| Naar-King, 2006 | USA | 24 | 1 |
| Nabukeera-Barungi, 2015 | Uganda | 1824 | 1 |
| Nachega, 2004 | South Africa | 12 | 1 |
| Nachega, 2006 | South Africa |  | 3 |
| Nachenga, 2006 | South Africa | 19 | 1 |
| Nachenga, 2009 | South Africa | 7622 | 1 |
| Nachenga, 2012 | South Africa | 274 | 1 |
| Nagata, 2012 | Kenya | 49 | 1 |
| Naidoo, 2010 | South Africa | 3164 | 1 |
| Naidoo, 2013 | Kenya |  | 1 |
| Nakigozi, 2013 | Uganda |  | 1 |
| Nakimuli- Mpungu, 2009 | Uganda | 122 | 1 |
| Nakimuli- Mpungu, 2013 | Uganda | 400 | 1 |
| Nakiyemba, 2006 | Uganda | 124 | 1 |
| Nam, 2008 | Botswana | 32 | 7 |
| Napua, 2016 | Mozambique | 51 | 2 |
| Nassali, 2009 |  | 289 | 3 |
| Ncama, 2008 | South Africa | 149 | 1 |
| Ndiaye, 2013 | Botswana | 82 | 1 |
| Ndirangu, 2009 | UK |  | 1 |
| Ndlovu, 2009 | Zimbabwe | 15 | 1 |
| Nduaguba, 2015 | Nigeria | 361 | 1 |
| Negash, 2013 | Ethiopia | 355 | 1 |
| Nel, 2013 | South Africa | 101 | 1 |
| Newman, 2007 |  |  | 2 |
| Newman, 2012 | Australia | 27 | 1 |
| Newman, 2015 (a) | Australia | 27 | 1 |
| Newman, 2015 (b) | Burundi, Cameroon and DRC | 18839 | 1 |
| Ngarina, 2013 | Tanzania | 23 | 2 |
| Nghoshi, 2016 | Namibia | 281 | 1 |
| Ngidi, 2013 |  |  | 1 |
| Nguyen, 2007 | Multi-country |  | 1 |
| Nguyen, 2013 | vietnam | 10 | 1 |
| Nieuwkerk, 2001 |  |  | 1 |
| Njunga, 2010 | Malawi |  | 1 |
| Nozaki, 2011 | Zambia | 518 | 2 |
| Nsigaye, 2010 | South Africa |  | 1 |
| Nsimba, 2010 | Tanzania | 207 | 2 |
| Nunn, 2010 | USA | 19 | 1 |
| Nunu, 2010 | Swaziland |  | 1 |
| Nwauche, 2006 | Nigeria | 187 | 2 |
| Nyanzi-Wakholi, 2009 | Uganda | 82 | 3 |
| Nyanzi-Wakholi, 2012 | Uganda | 120 | 4 |
| Nyogea, 2015 | Tanzania |  | 1 |
| O’Brien, 2013 | Zimbabwe |  | 1 |
| Obirikorang, 2013 | Ghana | 201 | 1 |
| Ochieng-Ooko, 2010 | Kenya | 50275 | 1 |
| Odlum M, 2012 |  |  | 1 |
| Oggins, 2003 | USA | 62 | 1 |
| O'Gorman, 2010 |  | 70 | 3 |
| Ohene, 2013 | Ghana | 683 | 1 |
| Okoror, 2013 | Nigeria | 35 | 3 |
| Oku, 2013 | Nigeria | 411 | 2 |
| Oku, 2014 | Nigeria | 393 | 2 |
| O'Laughlin, 2012 |  |  | 1 |
| Olisah, 2010 | Nigeria | 310 | 1 |
| Olowookere, 2008 | Nigeria | 318 | 2 |
| Olupot-Olupot, 2008 | Uganda | 40 | 2 |
| Omenka, 2012 | Nigeria | 28 | 1 |
| Omole, 2012 | Nigeria | 305 | 1 |
| Omosanya, 2014 | Nigeria | 100 | 1 |
| Omotala, 2015 | Nigeria | 12 | 1 |
| Onyango, 2013 | Kenya | 116 | 1 |
| Orner, 2008 | South Africa |  | 1 |
| Orrell, 2003 | South Africa | 289 | 1 |
| Otieno, 2010 | Kenya | 116 | 1 |
| Oumar, 2007 | Mali | 344 | 1 |
| Oyore, 2013 | Kenya | 450 | 1 |
| Oyugi, 2007 | Uganda | 97 | 1 |
| Pai, 2012 |  | 1002 | 1 |
| Palmer, 2003 |  |  | 1 |
| Parasher, 2011 |  |  | 1 |
| Parkes, 2006 | Uganda |  | 1 |
| Parkes-Ratanshi, 2010 | Uganda | 400 | 2 |
| Parrott, 2011 | Malawi | 60 | 1 |
| Pecoraro, 2013 | USA | 38 | 1 |
| Pefura- Yone, 2013 | Cameroon | 889 | 2 |
| Peltzer, 2010 |  | 815 | 6 |
| Peltzer, 2011 |  | 746 | 3 |
| Penn, 2011 | South Africa | 34 | 2 |
| Peraza, 2015 | Cuba | 21 | 1 |
| Peretti-Watel, 2006 | France | 2932 | 2 |
| Petersen, 2014 | South Africa |  | 1 |
| Peterson, 2006 | The Gambia | 64 | 1 |
| Phakathi Z, 2011 | South Africa |  | 1 |
| Phaswana-Mafuya, 2009 | South Africa | 1 | 1 |
| Pienaar, 2012 | South Africa | 6 | 1 |
| Pinheiro, 2002 | Brazil |  | 1 |
| Plitt, 2009 |  |  | 1 |
| Portelli, 2012 |  |  | 2 |
| Posse, 2009 | Uganda |  | 1 |
| Potchoo, 2010 | Togo |  | 2 |
| Powell-Cope, 2003 | USA | 24 | 4 |
| Prentice, 2001 | Canada |  | 1 |
| Price et al, 2014 | Malawi | 43 | 1 |
| Primary studies included in reviews | Country | Sample size | Total number of reviews including the primary study |
| Proctor, 1999 | USA | 39 | 3 |
| Prutch, 2005 | USA | 15 | 1 |
| Pugatch, 2002 | USA | 6 | 1 |
| Puoane, 2012 | South Africa | 6 | 1 |
| Pyne-Mercier | Kenya | 2534 | 2 |
| Quinlivan EB, 2013 |  |  | 1 |
| Radcliffe, 2006 | USA | 30 | 1 |
| Rajabiun ,2011 |  |  | 1 |
| Ramadhani, 2007 | Tanzania | 150 | 1 |
| Ramers, 2010 |  | 57210 | 1 |
| Rao, 2007 | USA | 25 | 1 |
| Rasmussen, 2013 | Guinea-Bissau | 20 | 1 |
| Rasschaert, 2014 | Mozambique | 79 | 1 |
| Reback, 2003 | USA | 23 | 2 |
| Reddington, 2000 |  |  | 1 |
| Remien, 2003 | USA | 152 | 4 |
| Reynolds, 1999 | USA | 15 | 1 |
| Reynolds, 2004 |  |  | 1 |
| Rhodes, 2012 | Russia | 38 | 1 |
| Richter, 2002 | USA | 33 | 2 |
| Rivero-Mendez, 2010 | Puerto Rico | 1 | 2 |
| Roberson, 2009 | USA | 12 | 1 |
| Roberson, 2012 | USA | 12 | 1 |
| Roberts, 2000 (a) | USA | 28 | 2 |
| Roberts, 2003 | USA | 23 | 1 |
| Roberts, 2005 | USA | 20 | 1 |
| Rochon, 2011 | USA | 31 | 1 |
| Rongkavilit, 2010 | Thailand | 10 | 1 |
| Root, 2013 | Swaziland | 79 | 1 |
| Ross, 2011 | South Africa | 19 | 2 |
| Rougemont, 2009 | Cameroon | 312 | 1 |
| Roura, 2009 (a) | Tanzania | 77 | 1 |
| Roura, 2009 (b) | Tanzania | 42 | 1 |
| Roura, 2009 (c ) | Tanzania | 53 | 1 |
| Roura, 2009 (d) | Tanzania | 42 | 1 |
| Roux, 2011 | Cameroon | 401 | 1 |
| Rowe, 2005 | South Africa | 6 | 1 |
| Ruanjahn, 2010 | Thailand | 32 | 3 |
| Russell, 2003 | Uganda | 38 | 1 |
| Russell, 2016 | USA | 57 | 1 |
| Ryan, 2003 | USA | 27 | 3 |
| Saberi, 2013 | USA | 14 | 1 |
| Sabin, 2008 | China | 36 | 3 |
| Sacajiu, 2009 | USA | 90 | 1 |
| Safren, 2005 | India | 304 | 1 |
| Salami, 2010 | Nigeria | 253 | 1 |
| Salmen, 2015 | Kenya | 82 | 1 |
| Sam, 2015 | Ghana | 426 | 1 |
| Samuels, 2016 | Indonesia |  | 1 |
| Sanjobo, 2008 | Zambia | 60 | 3 |
| Sanjobo, 2009 | Zambia | 60 | 1 |
| Sankar, 2002 | USA | 15 | 3 |
| Sankar, 2011 | USA | 80 | 1 |
| Sarang, 2013 | Russia | 34 | 1 |
| Sarna, 2008 | India | 310 | 1 |
| Sasaki, 2012 | Zambia | 157 | 2 |
| Savini, 2003 |  |  | 1 |
| Schenk, 2014 | Kenya |  | 1 |
| Schilder, 2001 | Canada | 47 | 2 |
| Schneider, 2004 |  |  | 1 |
| Schrader, 2011 |  |  | 1 |
| Schrimshaw, 2005 | USA | 158 | 1 |
| Schumaker, 2008 | Zambia | 31 | 1 |
| Schwartz, 2001 | USA | 215 | 1 |
| Seeley, 2010 | Uganda | 70 | 1 |
| Selman, 2013 | Kenya and Uganda | 83 | 1 |
| Senkomago, 2011 | Uganda | 140 | 2 |
| Shaahu, 2008 | Nigeria | 428 | 1 |
| Shah, 2007 | India | 279 | 1 |
| Shalihu, 2014 | Namibia | 18 | 1 |
| Shambley-Ebron, 2001 | USA | 10 | 1 |
| Sharma, 2007 | India | 226 | 1 |
| Shedlin, 2013 | USA | 113 | 3 |
| Sherr, 2008 | UK | 502 | 1 |
| Shin, 2011 | Peru | 13 | 2 |
| Shroufi, 2013 | South Africa |  | 1 |
| Shumba, 2013 | Uganda | 763 | 1 |
| Shuster, 2009 | South Africa | 1 | 2 |
| Sidat, 2007 |  |  | 1 |
| Siegel, 2000 | USA | 51 | 2 |
| Siegel, 2001 |  |  | 2 |
| Simoni, 2002 | USA |  | 1 |
| Siril, 2014 | Tanzania | 78 | 1 |
| Sisay, 2013 | Ethiopia | 518 | 1 |
| Sison, 2013 | USA |  | 1 |
| Siu, 2013 | Uganda | 17 | 2 |
| Skhosana, 2006 | South Africa |  | 1 |
| Skovdal, 2011 (a) | Zimbabwe | 78 | 1 |
| Skovdal, 2011 (b) | Zimbabwe | 78 | 1 |
| Small, 2009 | British Columbia | 12 | 1 |
| Smith, 2007 | Namibia | 22 | 1 |
| Sogarwal, 2009 | India | 1366 | 1 |
| Sow, 2012 | Senegal | 60 | 1 |
| Sowell, 2001 | USA | 322 | 1 |
| Spire, 2002 | Cambodia | 346 | 1 |
| Spire, 2008 |  |  | 1 |
| Sprague, 2011 |  | 153 | 1 |
| Starks, 2008 | China | 29 | 2 |
| Stein, 2000 |  |  | 1 |
| Sterhout, 2005 | Malawi |  | 1 |
| Sternhell | Australia | 79 | 1 |
| Stevens, 2009 | USA | 55 | 1 |
| Stevens, 2009 | USA |  | 2 |
| Stinson, 2010 |  | 14987 | 1 |
| Stinson, 2012 | South Africa | 28 | 2 |
| Stirrat, 2006 | USA | 215 | 1 |
| Stone, 1998 | USA | 56 | 2 |
| Stoskopf , 2001 | USA | 111 | 1 |
| Stout, 2004 | Costa Rica |  | 1 |
| Stubbs, BA; et al; 2009 | Mozambique | 375 | 1 |
| Tadesse, 2014 | Ethiopia | 647 | 2 |
| Talam, 2008 | Kenya | 384 | 2 |
| Tapp, 2011 |  |  | 1 |
| Tarakeshwar, 2007 | India |  | 1 |
| Taylor, 2014 | USA | 35 | 1 |
| Tessema, 2010 | Ethiopia | 504 | 2 |
| Theilgaard, 2011 | Tanzania | 40 | 2 |
| Thielman, 2014 | Tanzania | 442 | 1 |
| Thobias, 2009 | Namibia |  | 1 |
| Thomas, 2015 | Australia | 163 | 1 |
| Thompson, 2009 | Australia | 20 | 2 |
| Thorpe, 2008 (a) | Australia | 18 | 1 |
| Thorpe, 2008 (b) | Australia | 18 | 1 |
| Thurman, 2010 | Rwanda | 75 | 1 |
| Tilahun, 2012 | Ethiopia | 9 | 1 |
| Tiruneh, 2016 | Ethiopia | 105 | 1 |
| Tiyou, 2010 | Ethiopia | 319 | 1 |
| Tomori, 2014 | Tanzania | 14 | 1 |
| Topp, 2010 | Zambia |  | 1 |
| Toth, 2013 | USA | 140 | 1 |
| Treffry-Goatley, 2016 | South Africa | 20 | 1 |
| Treves-Kagan, 2016 | South Africa |  | 1 |
| Tripathi, 2013 | Ukraine |  | 1 |
| Tsague, 2010 |  | 40674 | 1 |
| Tsarenko, 2011 | Australia | 15 | 1 |
| Tsega, 2015 | Ethiopia | 351 | 1 |
| Tshabalala, 2010 |  |  | 1 |
| Tsuyuki, 2015 | USA | 44 | 1 |
| Tucker, 2004 |  |  | 1 |
| Tuller, 2009 | Uganda | 41 | 1 |
| Tuller, 2010 | Uganda | 41 | 2 |
| Tumwine, 2012 | Uganda | 39 | 1 |
| Turner, 2000 |  |  | 2 |
| Tweya, 2014 | Malawi | 111 | 1 |
| Ujiji, 2011 | Kenya | 37 | 2 |
| Ukwe, 2010 | Nigeria | 299 | 1 |
| Unge, 2008 | Kenya | 830 | 3 |
| Unge, 2009 | Kenya | 352 | 1 |
| Unge, 2010 | Kenya |  | 1 |
| Uzochukwu, 2009 | Nigeria | 174 | 1 |
| Van den Ven |  | 532 | 1 |
| Van Dijk, 2009 | Zambia | 192 | 1 |
| Van Dyk, 2011 | vietnam | 48 | 1 |
| van Griensven, 2010 | Rwanda | 609 | 1 |
| van Loggerenberg, 2015 | South Africa | 30 | 1 |
| Van Oosterhout, 2005 | Malawi | 176 | 2 |
| Van Schalkwyk, 2013 |  | 250 | 1 |
| Van Tam, 2011 (a) | Vietnam |  | 1 |
| Van Tam, 2011 (b) | South Africa | 394 | 1 |
| Varga, 2008 |  | 140 | 1 |
| Varga, 2008 | South Africa | 100 | 1 |
| Vaughan, 2011 | USA | 31 | 1 |
| Vaz, 2007 |  |  | 2 |
| Venkatesh, 2010 | India | 198 | 1 |
| Vervoort, 2009 | Multi-country | 30 | 3 |
| Vissman, 2011 | USA | 25 | 1 |
| Vissman, 2011 | USA | 66 | 1 |
| Vissman, 2012 | USA | 20 | 1 |
| Vreeman, 2012 | Kenya |  | 1 |
| Vyankandondera (b) | Rwanda | 47 | 2 |
| Vyankandondera, 2013 (a) | Rwanda | 213 | 1 |
| Vyavaharkar, 2008 | USA | 340 | 2 |
| Vyavaharkar, 2010 | USA | 22 | 1 |
| Wagner, 2004 | Uganda | 24 | 1 |
| Wagner, 2009 |  |  | 1 |
| Wakibi, 2011 | Kenya | 403 | 2 |
| Walcott, 2016 | USA | 60 | 1 |
| Walsh, 2001 |  |  | 1 |
| Walstrom, 2013 | Rwanda | 18 | 1 |
| Wanchu, 2007 | India | 200 | 1 |
| Wang, 2007 | China | 308 | 1 |
| Wang, 2008 | China | 181 | 1 |
| Wang, 2011 | China | 36 | 2 |
| Ware, 2005 |  |  | 1 |
| Ware, 2006 | Multi-country | 252 | 1 |
| Ware, 2009 | USA | 52 | 3 |
| Ware, 2013 |  |  | 1 |
| Wasti, 2009 | Nepal | 34 | 1 |
| Wasti, 2012 | Nepal | 17 | 4 |
| Watermeyer, 2011 | South Africa | 1 | 1 |
| Watermeyer, 2012 | South Africa | 26 | 1 |
| Watson-Jones, 2012 |  | 433 | 2 |
| Watt, 2010 | Tanzania | 340 | 2 |
| Watt., 2009 | Tanzania | 36 | 6 |
| Wei, 2013 | China |  | 1 |
| Weidle, 1999 | Uganda | 987 | 1 |
| Weidle, 2006 |  |  | 1 |
| Weigel, 2012 |  | 942 | 1 |
| Weiser, 2003 | Botswana | 169 | 3 |
| Weiser, 2010 | Uganda | 47 | 4 |
| Weiser, 2014 | Uganda | 438 | 1 |
| Wekesa, 2013 | Nairobi | 41 | 1 |
| Wendorf, 2012 | USA | 21 | 1 |
| Westerfelt, 2004 | USA | 21 | 3 |
| Whetten, 2006 | USA | 611 | 1 |
| Whetten, 2013 | Tanzania | 468 | 1 |
| Williams, 2006 | USA | 219 | 1 |
| Williams, 2011 |  |  | 1 |
| Wilson, 2001 | USA | 66 | 3 |
| Wilson, 2002 | USA | 200 | 2 |
| Wilson, 2004 | Australia |  | 1 |
| Winestone, 2010 |  | 36 | 1 |
| Winestone, 2012 | Kenya |  | 1 |
| Witteveen, 2002 | Netherlands | 28 | 4 |
| Wood, 2003 | USA | 36 | 1 |
| Wood, 2004 |  | 36 | 3 |
| Wooley, 2012 | Australia | 3 | 1 |
| Woolgar, 2014 | South Africa | 15 | 1 |
| Wroe, 2014 | Rwanda | 292 | 1 |
| Wrubel, 2005 | USA | 71 | 1 |
| Wrubel, 2010 | USA | 40 | 1 |
| Wyatt et al | USA | 75 | 1 |
| Xavier, 2012 | USA |  | 1 |
| Yahaya, 2006 | Nigeria | 100 | 1 |
| Yeap, 2010 | South Africa |  | 1 |
| Zablotska, 2009 | Australia | 270 | 1 |
| Zekeri, 2009 | USA | 205 | 1 |
| Zhou, 2016 | Malawi | 65 | 1 |
