# Supplementary material for: A mega-aggregation framework synthesis of the barriers and facilitators to linkage, adherence to ART and retention in care among people living with HIV
Source: Syst Rev. 2021 Feb 11;10:54. doi: 10.1186/s13643-021-01582-z (PMC7875685; doi:10.1186/s13643-021-01582-z)
Supplement: Supplementary file 10 — Additional file 10:. Summary of review level evidence: Linkage to ART [file 13643_2021_1582_MOESM10_ESM.docx]

**Additional file 10: Summary of review level evidence: Linkage to ART**

| **HIV model levels** | **Themes** | **Sub-themes** | **Children** | | **Adults** | |
| --- | --- | --- | --- | --- | --- | --- |
|  |  |  | **Barriers** | **Facilitators** | **Barriers** | **Facilitators** |
| **Individual** | **Beliefs about ART** | Beliefs about the right to decide |  |  | [64]L** |  |
|  |  | Negative beliefs about ART |  |  | [43]M**, [53]L, [60]L |  |
|  | **Coping strategies** | Poor coping strategies |  |  | [43]M** |  |
|  | **Daily routine and lifestyle** | Daily routine and lifestyle |  |  |  | [54]L |
|  | **Daily routine and lifestyle** | Substance Use |  |  | [43]M** |  |
|  | **Desires** | Care for family and children |  |  |  | [64]L** |
|  |  | Marriage and children | [73]L | [73]L |  |  |
|  |  | Normalisation to life before ART | [73]L | [73]L |  |  |
|  | **Disclosure** | Disclosure |  | [73]L |  |  |
|  | **Education and training Skills** | Education and training Skills | [73]L |  |  | [42]L* |
|  | **Experiences of HIV and ART** | Experiences of HIV and ART |  |  |  |  |
|  | **Fears** | Fear of economic loss related to treatment |  |  | [55]M** |  |
|  |  | Fear of reason for positive HIV diagnosis |  |  |  |  |
|  |  | Fear of the future |  |  |  |  |
|  |  | Fears of stigma | [73]L |  | [43]M**, [49]L, [50]L*, [53]L |  |
|  |  | Fears related to the effects of ART |  |  | [53]L, [60]L, [64]L** |  |
|  | **HIV Status** | Non-acceptance of HIV status |  |  | [50]L*, [53]L |  |
|  | **Knowledge and understanding** | Knowledge of HIV, ART and HAART |  | [73]L | [43]M**, [49]L, [50]L*, [53]L | [66]L** |
|  |  | Uncertainty and conflicting messages |  |  | [53]L |  |
|  | **Medication** | Forgetting and misplacing medication |  |  | [55]M** |  |
|  |  | Negative side effects of medication |  |  | [45]L, [64]L** |  |
|  |  | Pill burden and regimen |  |  | [64]L** |  |
|  |  | Reminder of status |  |  | [43]M** | [65]H** |
|  | **Past trauma and abuse** | Experienced past trauma or abuse | [73]L |  |  |  |
|  | **Physical health** | Comorbidities |  |  | [43]M**, [53]L | [43]M** [54]L, |
|  |  | Feeling better and healthier |  |  | [50]L*, [53]L, [54]L, [55]M** |  |
|  |  | Feeling ill and disease progression |  |  | [64]L** | [50]L*, [53]L, [54]L, [64]L** |
|  | **Psychological distress and emotional reactions** | Demotivated |  |  | [43]M**, [49]L, [53]L |  |
|  |  | Negative emotion | [73]L |  | [43]M**, [50]L* |  |
|  |  | Perception of self | [73]L |  | [50]L**, [60]L |  |
|  |  | Psychological distress and emotional impact |  |  | [50]L*, [64]L** |  |
|  | **Socio-demographic** | Age |  |  | [43]M** | [64]L** |
|  |  | Employment |  |  | [43]M** |  |
|  |  | Gender |  |  | [60]L, [64]L** |  |
|  |  | Identification document |  |  | [66]L** |  |
| **Interpersonal** | **Competing life demands** | Competing life demands |  |  | [53]L, [50]L*, [60]L, [66]L** |  |
|  | **Disclosure** | Disclosure | [73]L | [73]L | [50]L* | [50]L* |
|  |  | Non-disclosure | [73]L |  | [49]L, [50]L*, [53]L, [55]M**, [60]L |  |
|  | **Relationships in household** | Conflict and tension in family relationships |  |  | [43]M** |  |
|  |  | Gender and power in household |  |  | [53]L, [50]L*, [55]M |  |
|  |  | Supportive family relationships |  | [73]L |  | [44]L**, [50]L* |
|  |  | Supportive partner |  |  |  | [58]L |
|  |  | Unsupportive family relationships | [73]L |  | [43]M**, [50]L* |  |
|  |  | Unsupportive partner |  |  | [55]M** |  |
| **Community** | **Community beliefs and practices** | Beliefs about HIV and ART |  |  | [50]L* |  |
|  |  | Gender norms |  |  | [50]L* |  |
|  |  | Preference for traditional healers and medicines |  |  | [50]L* |  |
|  | **Peers and support groups** | Medication companion |  | [73]L |  | [42]L*, [53]L |
|  |  | Peer support |  |  |  | [42]L* |
|  |  | Support groups |  | [73]L |  |  |
|  |  | Supportive supervisors and teachers |  | [73]L |  |  |
|  |  | Unsupportive supervisors and teachers | [73]L |  |  |  |
|  | **Religious institutions** | Religious institutions |  | [73]L |  | [50]L* |
|  | **Social support** | Social support |  |  |  | [44]L**, [53]L |
|  | **Stigma and discrimination** | Experiences of stigma | [73]L |  | [43]M**, [44]L* [53]L, |  |
| **Institutional** | **Counselling practices and principles** | Awareness of literacy and language barriers |  |  | [50]L*, [66]L** |  |
|  |  | Awareness of who is providing the counselling |  |  |  | [50]L* |
|  |  | In depth pre and post counselling when testing |  | [73]L | [50]L* | [42]L*, [50]L*, [53]L, [64]L** |
|  |  | Including patients’ beliefs and respecting cultural practices |  |  |  | [50]L* |
|  |  | Poor counselling | [73]L |  | [53]L |  |
|  | **Engagement with health care workers** | Disengaged and unsupportive relationships | [73]L |  | [53]L |  |
|  |  | Frequency and duration of engagements |  |  | [50]L* | [46]M*** |
|  |  | Supportive and collaborative relationships |  |  |  | [50]L*, [53]L |
|  | **Health care worker recommendations and care** | Health care worker does not do timely tests or referrals |  |  | [49]L |  |
|  |  | Health care worker does not provide holistic care |  |  | [49]L |  |
|  |  | Provider input |  |  | [43]M** | [50]L*, [53]L |
|  | **Models of Care** | Adolescent services |  | [73]L |  |  |
|  |  | Gaps in referrals |  |  | [49]L |  |
|  |  | Hospital admission |  |  |  | [53]L |
|  |  | Integrated care |  |  |  | [50]L* |
|  |  | Integrated mental health care |  | [73]L |  | [64]L** |
|  |  | Involving patients as peer facilitators |  | [73]L |  |  |
|  |  | Lack of integrated care |  |  | [49]L |  |
|  |  | Mobile and home visits |  |  | [53]L |  |
|  |  | PMTCT, ANC and HIV Integration |  |  | [46]M*** | [49]L, [64]L** |
|  | **Perception of health care workers** | Negative perceptions of health care workers |  |  | [43]M**, [53]L |  |
|  |  | Positive perceptions of health care workers |  |  |  | [50]L* |
|  | **Relocation to other facility** | Transfers and Relocation |  |  | [53]L |  |
|  | **Service delivery** | Clinic times |  |  | [50]L*, [53]L |  |
|  |  | Drug and test resources |  |  | [53]L |  |
|  |  | Lack of privacy | [73]L |  | [50]L*, [53]L, [55]M**, [60]L |  |
|  |  | Experiences at the clinic | [73]L |  | [49]L, [50]L*, [53]L, [58]L, [60]L | [50]L* |
|  |  | Physical clinic environment | [73]L |  | [49]L, [53]L | [53]L |
|  |  | Scheduled appointments |  |  | [50]L*, [55]M** |  |
|  |  | Staff turnover | [73]L |  | [49]L, [50]L*, [53]L, [60]L |  |
|  | **Stigma and health care engagement** | Favouritism  Gender and sexuality bias |  |  | [60]L, [64]L** |  |
|  |  | Gender bias |  |  | [50]L* |  |
|  |  | HIV related stigma |  |  | [50]L*, [53]L |  |
|  |  | Patient anticipates stigma | [73]L |  | [50]L*, [53]L, [58]L |  |
| **Structural** | **Financial costs for care** | Free ART still has costs | [73]L |  | [43]M**, [49]L |  |
|  | **Financial relief for care** | Grants |  |  |  | [45]L, [53]L |
|  | **Healthcare policies** | Access and eligibility policies |  |  | [50]L*, [58]L |  |
|  |  | Health insurance |  |  | [50]L* |  |
|  | **Income and food security** | Income and financial status | [73]L |  | [43]M**, [45]L, [53]L, [58]L | [44]L** |
|  | **Living conditions and context** | Housing |  |  | [43]M** |  |
|  | **Transport and distance to clinic** | Transport and distance to clinic | [73]L |  | [53]L, [55]M**, [58]L, [59]L | [53]L, [66]L** |

. Low and middle income countries; *High income countries; **Not able to discern economic category of countries in review; ***Both high income countries and low and middle income countries. ^L^ indicates a low quality review, ^M^ indicates a medium quality review, ^H^ indicates a high quality review.
