# Supplementary material for: A mega-aggregation framework synthesis of the barriers and facilitators to linkage, adherence to ART and retention in care among people living with HIV
Source: Syst Rev. 2021 Feb 11;10:54. doi: 10.1186/s13643-021-01582-z (PMC7875685; doi:10.1186/s13643-021-01582-z)
Supplement: Supplementary file 11 — Additional file 11. Summary of review level evidence: Adherence to ART [file 13643_2021_1582_MOESM11_ESM.docx]

**Additional file 11: Summary of review level evidence: Adherence to ART**

| **HIV Model levels** | **Themes** | **Sub-themes** | **Children and Youth (0-24 years)** | | **Adults** | |
| --- | --- | --- | --- | --- | --- | --- |
|  |  |  | **Barriers** | **Facilitators** | **Barriers** | **Facilitators** |
| **Individual** | **Beliefs about ART** | **Negative beliefs about ART** | [41]M*, [73]L |  | [43]H*, [47]L, [48]L*, [51]L*, [54]L, [56]L, [64]L*, [65]H***, [70]L***, [71]L*** |  |
|  |  | **Positive beliefs about ART** |  | [41]M |  | [47]L, [54]L, [55]M*, [56]L, [64]L*, [65]H***, [70]L***, [72]L |
|  | **Beliefs about HIV** | **Negative beliefs about HIV** |  |  | [54]L, [63]L |  |
|  | **Cognitive impairment** | **Cognitive impairment** |  |  | [54]L | [54]L |
|  | **Coping strategies** | **Coping strategies for emotional regulation and self-management** |  | [41]M, [68]L* |  | [41]M72, [44]L***, [47]L, [48]L*, [52]L*, [54]L, [56]L, [63]L***, [64]L*, [65]H***, [66]L*, [67]M, [71]L*, [72]L |
|  |  | **Poor coping strategies** | [68]L* | [73]L | [43]H*, [70]L*** |  |
|  | **Criminal justice system involvement** | **Criminal justice system involvement** |  |  | [48]L* |  |
|  | **Daily routine and lifestyle** | **Daily routine and lifestyle** | [65]H* | [41]M | [44]L*, [47]L, [48]L*, [63]L, [64]L*, [65]H***, [67]M, [70]L***, [72]L | [44]L***, [54]L, [70]L*** |
|  |  | **Lifestyle** |  |  | [54]L |  |
|  |  | **Sleeping** |  |  | [47]L, [65]H*, [70]L***, [72]L |  |
|  |  | **Substance Use** | [68]L* |  | [43]H*, [47]L, [48]L*, [53]L, [55]M, [56]L, [61]M*, [64]L*, [65]H***, [70]L***, [71]L***, [72]L |  |
|  | **Desires** | **Care for family and children** |  | [73]L | [44]L*, [71]L*** | [47]L, [51]L*, [54]L, [55]M*, [62]H***, [63]L, [64]L*, [65]H*, [67]M, [70]L***, [71]L***, [72]L |
|  |  | **Look and feel healthy** |  |  | [70]L*** | [47]L, [54]L, [55]M* [56]L, [62]H |
|  |  | **Marriage and children** |  | [73]L |  | [63]L |
|  |  | **Normalisation to life before ART** | [65]H*, [73]L | [73]L | [65]H*, [72]L | [47]L |
|  | **Disclosure** | **Disclosure** |  | [41]M, [73]L |  | [55]M*, [70]L*** |
|  | **Education and Training Skills** | **Education and Training Skills** |  | [73]L |  | [41]L** |
|  |  | **Experiences of HIV and ART** |  |  |  | [55]M, [63]L |
|  | **Experiences of HIV and ART** | **Experiences of HIV and ART** |  |  |  | [47]L, [54]L, [55]M, [61]M, [63]L, [67]M, [70]L |
|  | **Fears** | **Fear of declining physical health** |  |  |  | [54]L |
|  |  | **Fear of reason for positive HIV diagnosis** |  |  | [47]L |  |
|  |  | **Fear of the future** |  |  | [63]L |  |
|  |  | **Fears of stigma** | [41]M, [65]H***, [68]L*, [73]L*** | [41]M* | [43]H*, [47]L, [48]L*, [50]L**, [52]L*, [54]L, [55]M***, [56]L, [63]L, [64]L*, [65]H***, [67]M, [70]L***, [71]L***, [72]L |  |
|  |  | **Fears related to the effects of ART** | [65]H* |  | [55]M*, [62]H***, [63]L, [64]L*, [65]H*, [70]L***, [71]L*, [72]L |  |
|  | **HIV Status** | **Acceptance of HIV status** |  |  |  | [44]L, [47]L, [48]L*, [54]L, [63]L, [65]H*, [70]L*** |
|  |  | **Non-acceptance of HIV status** | [41]M, [65]H* |  | [44]L, [48]L*, [54]L, [55]M*, [56]L, [65]H* |  |
|  | **Knowledge and understanding** | **Knowledge of HIV Status** | [41]M, [73]L | [41]M, [73]L | [61]M, [65]H*** |  |
|  |  | **Knowledge of HIV, ART and HAART** |  | [41]M, [73]L | [43]H, [44]L, [65]H***, [67]M, [72]L | [41]L**, [47]L, [54]L, [55]M*, [64]L*, [65]H*, [67]M, [70]L*** |
|  |  | **Uncertainty and conflicting messages** | [41]M, [65]H***, [73]L | [41]M | [54]L, [63]L, [65]H***, [67]M, [70]L***, [71]L*** | [55]M, [63]L, [67]M |
|  | **Medication** | **Being away from home** | [41]M, [73]L |  | [47]L, [54]L, [55]M, [65]H***, [70]L***, [72]L | [47]L |
|  |  | **Forgetting and misplacing medication** | [41]M, [65]H* |  | [47]L, [54]L, [55]M*, [58]L, [64]L*, [65]H*, [70]L***, [72]L |  |
|  |  | **Medication characteristics** | [41]M, [65]H***, [29]*, [73]L |  | [47]L, [48]L*, [56]L, [65]H***, [70]L*** |  |
|  |  | **Medication reminders** |  | [41]M, [73]L |  | [47]L, [54]L, [55]M, [65]H***, [70]L***, [72]L |
|  |  | **Negative side effects of medication** | [41]M, [65]H*, [68]L*, [29]*, [73]L |  | [41]M, [43]H*, [44]L*, [48]L*, [61]M*, [62]H*, [64]L*, [65]H*, [66]L*, [70]L*, [73]L |  |
|  |  | **No privacy when taking pills** | [41]M |  | [41]L**, [47]L, [70]L*** |  |
|  |  | **Pill burden and regimen** | [41]M, [65]H***, [68]L*, [29]*, [73]L | [68]L* | [43]H*, [44]L*, [47]L, [48]L*, [54]L, [55]M, [56]L, [62]H, [64]L*, [65]H***, [70]L***, [72]L | [41]L**, [44]L*, [65]H***, [71]L*** |
|  |  | **Reminder of status** |  |  | [43]H*, [70]L*** |  |
|  |  | **Skipping medication** |  | [41]M | [70]L*** |  |
|  | **Past trauma and abuse** | **Experienced past trauma or abuse** | [68]L*, [73]L |  | [54]L | [54]L |
|  | **Physical health** | **Comorbidities** |  |  | [43]H*, [48]L*, [65]H*** | [55]M* |
|  |  | **Feeling better and healthier** | [41]M, [65]H***, [73]L |  | [47]L, [54]L, [65]H***, [72]L | [43]H, [47]L, [54]L, [61]M, [63]L, [65]H*, [67]M, [70]L***, [72]L |
|  |  | **Feeling ill and disease progression** | [68]L*, [73]L |  | [47]L, [65]H***, [67]M | [54]L, [63]L*, [64]L* |
|  | **Psychological distress and emotional reactions** | **Demotivated** |  |  | [47]L, [48]L*, [63]L, [70]L*, [71]L, [72]L |  |
|  |  | **Negative emotion** | [41]M, [68]L*, [73]L | [73]L | [43]H*, [44]L*, [47]L, [54]L, [63]L, [64]L*, [65]H*, [70]L***, [71]L***, [72]L | [54]L |
|  |  | **Perception of self** | [41]M, [61]M*, [73]L | [73]L | [47]L, [54]L, [65]H*, [71]L*** |  |
|  |  | **Psychological distress and emotional impact** | [41]M, [61]M*, [73]L |  | [44]L*, [54]L, [61]M***, [63]L, [65]H***, [67]M, [70]L*** | [54]L |
|  | **Sociodemographic** | **Age** | [41]M, [68]L* | [68]L* | [43]H*, [54]L, [55]M*, [71]L*** | [71]L |
|  |  | **Education** | [68]L* | [68]L | [54]L, [55]M* | [54]L, [55]M |
|  |  | **Employment** |  |  | [43]H*, [48]L*, [54]L | [54]L, [58]L, [66]L |
|  |  | **Gender** | [41]M, [68]L* | [68]L* | [43]H*, [48]L*, [54]L, [63]L*, [64]L* | [54]L |
|  |  | **Marital status** |  |  | [54]L |  |
|  |  | **Race/ nationality** |  |  | [48]L*, [55]M |  |
|  |  | **Sexual partners** |  |  | [54]L |  |
|  |  | **Identification** |  |  | [66]L* |  |
|  | **Spiritual beliefs** | **Beliefs: Spiritual** | [41]M | [41]M | [47]L, [54]L, [67]M | [47]L, [54]L, [56]L, [63]L, [71]L*** |
|  | **Stigma and discrimination** | **Experiences of stigma** |  |  | [56]L |  |
|  | **Traditional Beliefs** | **Beliefs: Traditional** |  |  |  |  |
| **Interpersonal** | **Caregiver Factors** | **Access to caregivers** |  |  | [41]M |  |
|  |  | **Caregiver beliefs** | [41]M, [29]* |  | [67]M |  |
|  |  | **Caregiver Disclosure** | [41]M | [41]M |  |  |
|  |  | **Caregiver education** | [68]L* | [68]L* | [55]M* |  |
|  |  | **Caregiver reminders** |  | [41]M |  | [65]H*** |
|  |  | **Relation to caregiver** |  | [68]L* |  |  |
|  | **Competing life demands** | **Competing life demands** |  |  | [52]L, [55]M*, [63]L, [65]H*, [71]L*** |  |
|  | **Disclosure** | **Disclosure** |  |  |  |  |
|  |  | **Non-disclosure** | [41]M, [73]L |  | [47]L, [56]L, [63]L, [64]L, [70]L***, [71]L*** |  |
|  | **Family on ART** | **Family on ART** |  | [41]M | [47]L, [54]L, [55]M*, [67]M | [54]L |
|  | **Medication** | **Negative side effects of medication** |  |  | [48]L*, [56]L |  |
|  | **Relationships in household** | **Conflict and tension in family relationships** |  |  | [43]H*, [52]L*, [63]L, [65]H, [72]L | [54]L |
|  |  | **Gender and power in household** |  |  | [47]L, [55]M, [56]L, [63]L, [67]M, [71]L |  |
|  |  | **Supportive family relationships** |  | [73]L |  | [51]L*, [52]L*, [55]M*, [56]L, [63]L, [65]H*, [70]L*, [71]L*, [72]L |
|  |  | **Supportive partner** |  |  |  | [58]L, [67]M, [70]L*** |
|  |  | **Unsupportive family relationships** | [41]M, [65]H*, [68]L*, [73]L |  | [43]H*, [55]M***, [56]L, [65]H*, [70]L*, [72]L |  |
|  |  | **Unsupportive partner** |  |  | [70]L*** |  |
| **Community** | **Community beliefs and practices** | **Beliefs about HIV and ART** |  |  | [63]L, [67]M | [67]M |
|  |  | **Gender norms** |  |  | [54]L, [63]L | [54]L |
|  |  | **Patient lacks autonomy** |  |  | [55]M, [67]M |  |
|  |  | **Preference for traditional healers and medicines** |  |  | [63]L |  |
|  |  | **Supportive traditions** |  |  |  | [51]L* |
|  | **Financial support** | **Financial support** |  |  |  | [63]L, [70]L***, [72]L |
|  | **Peers and support groups** | **Medication companion** | [73]L | [73]L |  | [44]L, [54]L, [62]H**, [63]L |
|  |  | **Peer support** |  | [41]M |  | [62]H** |
|  |  | **Social isolation** | [65]H* |  | [44]L*, [65]H* |  |
|  |  | **Support groups** |  | [73]L |  | [47]L |
|  |  | **Supportive supervisors and teachers** |  | [73]L |  | [62]H** |
|  |  | **Unsupportive supervisors and teachers** | [73]L |  |  |  |
|  | **Religious institutions** | **Religious institutions** |  | [73]L |  | [64]L* |
|  | **Social support** | **Social support** |  |  |  | [47]L, [52]L*, [54]L, [56]L, [63]L, [65]H*, [70]L***, [71]L*** |
|  | **Stigma and discrimination** | **Experiences of stigma** | [73]L |  | [43]H*, [54]L, [64]L*, [72]L |  |
| **Institutional** | **Counselling practices and principles** | **Addressing shared community uncertainties** |  |  |  | [44]L* |
|  |  | **Awareness of literacy and language barriers** |  |  | [54]L, [66]L* |  |
|  |  | **In depth pre and post counselling when testing** |  | [41]M, [73]L |  | [47]L, [62]H**, [63]L |
|  |  | **Including patients beliefs and respecting cultural practices** |  |  | [63]L | [52]L*, [62]H** |
|  |  | **Poor counselling** | [73]L |  | [47]L, [63]L, [70]L***, [72]L |  |
|  |  | **Types of narrative used by health care workers** |  | [73]L |  |  |
|  | **Engagement with health care workers** | **Disengaged and unsupportive relationships** |  | [73]L | [47]L, [48]L*, [54]L, [63]L, [65]H*, [70]L*** |  |
|  |  | **Frequency and duration of engagements** |  | [68]L* |  |  |
|  |  | **Supportive and collaborative relationships** |  |  |  | [44]L*, [47]L, [51]L*, [56]L, [65]H*, [70]L*, [72]L |
|  | **Health care worker recommendations and care** | **Provider input** | [68]L* |  | [43]H* |  |
|  | **Models of Care** | **Adolescent services** |  | [73]L |  |  |
|  |  | **Family driven care** |  |  |  | [56]L |
|  |  | **Integrated mental health care** |  | [73]L | [54]L | [52]L, [54]L, [56]L |
|  |  | **Involving patients as peer facilitators** |  | [73]L |  |  |
|  |  | **Male services** |  |  | [62]H**, [63]L |  |
|  |  | **Mobile and home visits** |  |  | [63]L |  |
|  |  | **PMTCT, ANC and HIV Integration** |  |  | [46]M*** | [55]M***, [67]M |
|  |  | **PMTCT, ANC and HIV Integration** |  |  | [46]M*** |  |
|  | **Perception of health care workers** | **Expectations of providers** |  |  | [51]L*, [62]H** |  |
|  |  | **Negative perceptions of health care workers** |  |  | [46]M***, [47]L |  |
|  |  | **Positive perceptions of health care workers** |  | [41]M | [63]L |  |
|  | **Relocation to other facility** | **Transfers and relocation** |  |  | [64]L*, [66]L* |  |
|  | **Service delivery** | **Clinic times** | [41]M |  | [47]L, [52]L*, [54]L, [65]H*** | [70]L*** |
|  |  | **Drug and test resources** |  |  | [46]M***, [47]L, [48]L, [54]L, [55]M*, [61]M, [62]H**, [65]H*** | [56]L |
|  |  | **Lack of privacy** | [41]M |  | [47]L, [54]L, [55]M***, [62]H**, [63]L, [71]L*** |  |
|  |  | **Negative experiences at the clinic** | [73]L | [73]L | [47]L, [48]L*, [54]L, [55]M*, [65]H*, 55 |  |
|  |  | **Physical clinic environment** | [41]M |  | [54]L, [63]L |  |
|  |  | **Scheduled appointments** | [41]M | [41]M | [46]M*, [48]L*, [54]L, [55]M, [63]L, [67]M |  |
|  |  | **Staff turnover** | [73]L |  | [46]M* , [63]L |  |
|  |  | **Weak systems and protocols** |  |  | [46]M*** |  |
|  | **Stigma and health care engagement** | **Gender and sexuality bias** |  |  | [56]L |  |
|  |  | **HIV related stigma** |  |  | [52]L, [56]L, [63]L, [67]M, [72]L |  |
|  |  | **Patient anticipates stigma** |  |  | [51]L* |  |
| **Structural** | **Financial costs for care** | **Free ART still has costs** | [73]L | [73]L | [43]H*, [47]L, [52]L*, [54]L, [55]M*, [56]L, [60]L, [62]H**, [63]L, [64]L* | [41]M**, [47]L |
|  | **Financial relief for care** | **Grants** |  | [41]M |  | [47]L, [54]L, [62]H** |
|  | **Healthcare policies** | **Access and eligibility policies** |  |  | [62]H |  |
|  |  | **Health insurance** |  |  | [48]L |  |
|  |  | **Policies are hard to understand** |  |  | [62]H |  |
|  | **Income and food security** | **Income and financial status** |  |  | [47]L, [48]L*, [54]L, [55]M, [58]L, [63]L, [65]H*, [67]M, [70]L***, [72]L | [47]L |
|  | **Income and food security** | **Income and financial status** | [41]M, [73]L |  | [48]L*, [54]L, [55]M, [56]L, [62]H**, [65]H***, [67]M, [70]L***, [71]L***, [72]L | [55]M, [72]L |
|  | **Living conditions and context** | **Housing** | [68]L* |  | [43]H*, [48]L*, [52]L, [54]L, [65]H***, [70]L*** |  |
|  | **Transport and distance to clinic** | **Transport and distance to clinic** | [41]M, [73]L | [41]M | [43]H, [54]L, [55]M, [59]L, [60]L, [61]M, [62]H*** [63]L, [65]H, [67]M, [71]L, [72]L |  |

. Low and middle income countries; *High income countries; **Not able to discern economic category of countries in review; ***Both high income countries and low and middle income countries. L indicates a low quality review, M indicates a medium quality review, H indicates a high quality review.
