# Supplementary material for: A mega-aggregation framework synthesis of the barriers and facilitators to linkage, adherence to ART and retention in care among people living with HIV
Source: Syst Rev. 2021 Feb 11;10:54. doi: 10.1186/s13643-021-01582-z (PMC7875685; doi:10.1186/s13643-021-01582-z)
Supplement: Supplementary file 12 — Additional file 12. Summary of review level evidence: Retention in care [file 13643_2021_1582_MOESM12_ESM.docx]

**Additional file 12: Summary of review level evidence: Retention in care**

| **HIV Model level** | **Themes** | **Sub-themes** | **Children and Youth (0-24 years)** | | **Adults** | |
| --- | --- | --- | --- | --- | --- | --- |
|  |  |  | **Barriers** | **Facilitators** | **Barriers** | **Facilitators** |
| **Individual** | **Beliefs about ART** | **Negative beliefs about ART** | [73]L |  | [57]L |  |
|  | **Criminal justice system involvement** | **Criminal justice system involvement** |  |  | [42]L** |  |
|  | **Daily routine and lifestyle** | **Substance Use** |  |  | [52]L* |  |
|  | **Desires** | **Care for family and children** |  | [73]L |  | [57]L |
|  | **Desires** | **Look and feel healthy** |  |  |  | [57]L |
|  | **Desires** | **Marriage and children** |  | [73]L |  |  |
|  | **Desires** | **Normalisation to life before ART** | [73]L | [73]L |  |  |
|  | **Disclosure** | **Disclosure** |  | [73]L |  |  |
|  | **Education and Training Skills** | **Education and Training Skills** |  | [73]L |  | [42]L** |
|  | **Experiences of HIV and ART** | **Experiences of HIV and ART** |  |  | [50]L** |  |
|  | **Fears** | **Fears of stigma** | [73]L |  | [50]L**, [55]M*, [57]L, [66]L* |  |
|  |  | **Fears related to the effects of ART** |  |  | [57]L |  |
|  | **HIV Status** | **Non-acceptance of HIV status** |  |  | [55]M*, [57]L |  |
|  | **Knowledge and understanding** | **Knowledge of HIV, ART and HAART** |  | [73]L |  | [55]M*, [66]L* |
|  |  | **Uncertainty and conflicting messages** | [73]L |  | [55]M, [57]L | [55]M |
|  | **Medication** | **Negative side effects of medication** | [73]L |  | [57]L |  |
|  |  | **Pill burden and regimen** |  |  | [57]L |  |
|  | **Past trauma and abuse** | **Experienced past trauma or abuse** | [73]L |  | [52]L* |  |
|  | **Physical health** | **Feeling better and healthier** |  |  | [50]L**, [57]L |  |
|  |  | **Feeling ill and disease progression** |  |  | [57]L | [50]L** |
|  | **Psychological distress and emotional reactions** | **Demotivated** |  |  | [57]L |  |
|  |  | **Negative emotion** | [73]L |  | [52]L*, [57]L |  |
|  |  | **Perception of self** | [73]L | [73]L |  |  |
|  |  | **Psychological distress and emotional impact** |  |  | [50]L** |  |
|  | **Sociodemographic** | **Employment** | [52]L*, [58]L |  | [58]L |  |
|  |  | **Identification** | [66]L* |  |  |  |
|  | **Spiritual beliefs** | **Beliefs: Spiritual** |  |  | [55]M, [57]L |  |
|  | **Traditional Beliefs** | **Beliefs: Traditional** | [73]L |  |  |  |
| **Interpersonal** | **Competing life demands** | **Competing life demands** |  |  | [52]L*, [50]L**, [66]L* |  |
|  | **Disclosure** | **Disclosure** | [73]L | [73]L |  | [50]L**, [55]M* |
|  |  | **Non-disclosure** | [73]L |  | [50]L |  |
|  | **Relationships in household** | **Gender and power in household** |  |  | [50]L**, [56]L, [57]L |  |
|  |  | **Supportive family relationships** |  |  | [50]L** | [50]L**, [52]L* |
|  |  | **Supportive partner** |  |  |  | [58]L |
|  |  | **Unsupportive family relationships** | [73]L |  | [50]L**, [57]L |  |
|  |  | **Unsupportive partner** |  |  | [55]M, [57]L | [55]M |
| **Community** | **Community beliefs and practices** | **Beliefs about HIV and ART** |  |  | [50]L** |  |
|  |  | **Gender norms** |  |  | [50]L** |  |
|  |  | **Preference for traditional healers and medicines** |  |  | [50]L**, [73]L |  |
|  | **Peers and support groups** | **Medication companion** |  |  | [73]L |  |
|  |  | **Peer support** |  |  |  | [42]L** |
|  |  | **Support groups** |  | [73]L |  | [57]L |
|  |  | **Supportive supervisors and teachers** |  | [73]L |  |  |
|  |  | **Unsupportive supervisors and teachers** |  |  | [73]L |  |
|  | **Religious institutions** | **Religious institutions** |  | [73]L |  | [50]L** |
|  | **Social support** | **Social support** |  |  |  | [52]L* |
|  | **Stigma and discrimination** | **Experiences of stigma** | [73]L |  | [50]L**, [52]L*, [57]L, [66]L* |  |
| **Institutional** | **Counselling practices and principles** | **Awareness of literacy and language barriers** |  |  | [50]L**, [66]L* | [42]L** |
|  |  | **Awareness of who is providing the counselling** |  |  |  | [50]L** |
|  |  | **In depth pre and post counselling when testing** |  | [73]L | [50]L** | [42]L** |
|  |  | **Including patients beliefs and respecting cultural practices** |  |  |  | [42]L**, [50]L** |
|  |  | **Poor counselling** | [73]L |  | [57]L |  |
|  |  | **Types of narrative used by health care workers** |  | [73]L |  | [42]L** |
|  | **Engagement with health care workers** | **Disengaged and unsupportive relationships** |  | [73]L | [42]L**, [52]L*, [60]L |  |
|  |  | **Frequency and duration of engagements** |  |  | [50]L** | [46]M,  [50]L** |
|  |  | **Supportive and collaborative relationships** |  |  |  | [42]L**, [50]L** |
|  | **Health care worker recommendations and care** | **Health care worker provides holistic care** |  |  |  | [42]L** |
|  |  | **Provider input** |  |  |  | [50]L** |
|  | **Models of Care** | **Adolescent services** |  | [73]L |  |  |
|  |  | **Integrated care** | [50]L** |  |  | [50]L** |
|  |  | **Integrated mental health care** | [73]L |  |  | [52]L* |
|  |  | **Involving patients as peer facilitators** | [73]L |  |  |  |
|  |  | **Male services** |  |  |  | [42]L** |
|  |  | **Mobile and home visits** |  |  |  | [42]L** |
|  |  | **PMTCT, ANC and HIV Integration** |  |  | [46]M***, [55]M*** |  |
|  | **Perception of health care workers** | **Expectations of providers** |  |  |  | [42]L** |
|  |  | **Negative perceptions of health care workers** |  |  | [46]M*** | [42]L** |
|  |  | **Perception of health care workers** |  |  | [42]L** | [42]L** |
|  |  | **Positive perceptions of health care workers** |  |  |  | [42]L**, [50]L**, [55]M* |
|  | **Service delivery** | **Clinic times** |  |  | [42]L**, [50]L**, [52]L*, [57]L |  |
|  |  | **Drug and test resources** |  |  | [42]L**, [46]M*** | [42]L** |
|  |  | **Lack of privacy** |  |  | [42]L**, [50]L**, [52]L*, [55]M, [57]L |  |
|  |  | **Negative experiences at the clinic** | [73]L | [73]L | [52]L*, [50]L**, [55]M, [57]L, [60]L, [66]L* | [50]L** |
|  |  | **Physical clinic environment** |  | [73]L | [57]L | [42]L** |
|  |  | **Scheduled appointments** |  |  | [42]L**, [46]M***, [50]L**, [52]L*,[55]* | [42]L** |
|  |  | **Staff turnover** | [73]L |  | [46]M***, [50]L** |  |
|  |  | **Weak systems and protocols** |  |  | [42]L**, [46]M*** |  |
|  | **Stigma and health care engagement** | **Gender bias** |  |  | [50]L** |  |
|  |  | **HIV related stigma** |  |  | [50]L**, [52]L*, [57]L |  |
|  |  | **Patient anticipates stigma** | [73]L |  | [50]L**, [57]L |  |
| **Structural** | **Financial costs for care** | **Free ART still has costs** | [73]L |  | [42]L**, [52]L*, [58]L, [60]L |  |
|  | **Healthcare policies** | **Access and eligibility policies** |  |  | [42]L, [50]L**, [58]L |  |
|  |  | **Health insurance** |  |  | [50]L** |  |
|  |  | **Policies are hard to understand** |  |  | [42]L |  |
|  | **Income and food security** | **Income and financial status** | [73]L |  | [52]L*, [55]M, [57]L, [60]L | [55]M |
|  | **Living conditions and context** | **Housing** |  |  |  |  |
|  | **Transport and distance to clinic** | **Transport and distance to clinic** | [73]L |  | [52]L***, [55]M, [57]L, [59]L, [60]L | [59]L, [66]L* |
|  | **Financial relief for care** | **Grants** |  | [73]L |  |  |

. Low and middle income countries; *High income countries; **Not able to discern economic category of countries in review; ***Both high income countries and low and middle income countries. L indicates a low quality review, M indicates a medium quality review, H indicates a high quality review.
