# Supplementary material for: A mega-aggregation framework synthesis of the barriers and facilitators to linkage, adherence to ART and retention in care among people living with HIV
Source: Syst Rev. 2021 Feb 11;10:54. doi: 10.1186/s13643-021-01582-z (PMC7875685; doi:10.1186/s13643-021-01582-z)
Supplement: Supplementary file 13 — Additional file 13. Summary of themes and included reviews linked to outcomes [file 13643_2021_1582_MOESM13_ESM.docx]

**Additional file 13: Summary of themes and included reviews linked to outcomes**

| **Socio-ecological Level** | **Fourth order theme** | **Fourth order sub-themes** | **Fourth order codes** | **Third order concepts** | **Linkage** | | **Adherence** | | **Retention** | |
| --- | --- | --- | --- | --- | --- | --- | --- | --- | --- | --- |
|  |  |  |  |  | **Barrier** | **Facilitator** | **Barrier** | **Facilitator** | **Barrier** | **Facilitator** |
| **Individual** | **Beliefs about ART** | Beliefs about right to decide | Participants perceived right to decide | Participants believed it is their right to consider taking treatment over the good of the public in preventing transmission | [24] |  |  |  |  |  |
| **Individual** | **Beliefs about ART** | Negative beliefs about ART | Negative beliefs about ART | Strong beliefs in HIV and a conspiracy |  |  | [51] |  |  |  |
| **Individual** | **Beliefs about ART** | Negative beliefs about ART | Negative beliefs about ART | Perception that ART initiation was an acceptance of impending death | [64] |  | [64] |  |  |  |
| **Individual** | **Beliefs about ART** | Negative beliefs about ART | Negative beliefs about ART | Belief ART is a sexual stimulant |  |  | [54] |  |  |  |
| **Individual** | **Beliefs about ART** | Negative beliefs about ART | Negative beliefs about ART | Suspicious about medication: ART is harmful, toxic and will not work | [53] |  | [47], [54], [56], [64], [65], [70], [71], [73] |  | [17], [44] |  |
| **Individual** | **Beliefs about ART** | Negative beliefs about ART | Negative beliefs about ART | Pregnant women perceived the likelihood of their baby suffering from taking Art was greater than their baby acquiring HIV infection. |  |  | [71] |  |  |  |
| **Individual** | **Beliefs about ART** | Negative beliefs about ART | Negative beliefs about ART | Belief that drugs increased appetite | [13 |  |  |  |  |  |
| **Individual** | **Beliefs about ART** | Negative beliefs about ART | Negative beliefs about ART | Negative beliefs about benefits of taking ART and resistance | [43], [60] |  | [43], [48], [54], [64], [65], [69], [70], [71] | [1] | [17] |  |
| **Individual** | **Beliefs about ART** | Negative beliefs about ART | Negative beliefs about ART | Should not mix ART with other treatments | [53] |  | [47] |  |  |  |
| **Individual** | **Beliefs about ART** | Positive beliefs about ART | Positive beliefs about ART | ART helps you look healthy to others |  |  |  | [7] |  |  |
| **Individual** | **Beliefs about ART** | Positive beliefs about ART | Positive beliefs about ART | Belief in ART benefit and that medication works |  |  |  | [1], [7], [14], [55], [16], 24], [25] |  |  |
| **Individual** | **Beliefs about ART** | Positive beliefs about ART | Positive beliefs about ART | Increased confidence in ART with use |  |  |  | [14] |  |  |
| **Individual** | **Beliefs about ART** | Positive beliefs about ART | Positive beliefs about ART | Medication takes priority over substance abuse |  |  | [64], [65] |  |  |  |
| **Individual** | **Beliefs about ART** | Positive beliefs about ART | Positive beliefs about ART | Belief that ART will keep them alive and help them live longer |  |  |  | [41], [43] |  |  |
| **Individual** | **Beliefs about HIV** | Negative beliefs about HIV | Negative beliefs about HIV | Belief that ART and national health policies are used to exploit low income countries by wealthier nations |  |  | [63] |  |  |  |
| **Individual** | **Beliefs about HIV** | Negative beliefs about HIV | Negative beliefs about HIV | Doubtful of tests reliability | [13 |  |  |  |  |  |
| **Individual** | **Beliefs about HIV** | Negative beliefs about HIV | Negative beliefs about HIV | Lack of belief in the existence of HIV | [13 |  | [54] |  |  |  |
| **Individual** | **Beliefs about HIV** | Negative beliefs about HIV | Negative beliefs about HIV | HIV is witchcraft and magic can protect communities |  |  | [63] |  |  |  |
| **Individual** | **Cognitive impairment** | Cognitive impairment | Cognitive impairment | Cognitive impairment |  |  | [54] | [14] |  |  |
| **Individual** | **Coping strategies** | Coping strategies for emotional regulation and self-management | Being hopeful | Being hopeful |  |  | [47] | [12] |  |  |
| **Individual** | **Coping strategies** | Coping strategies for emotional regulation and self-management | Coping strategies for emotional regulation and self-management | Prisoners keep own prescription |  |  | [42] | [2] |  |  |
| **Individual** | **Coping strategies** | Coping strategies for emotional regulation and self-management | Coping strategies for emotional regulation and self-management | Self-efficacy-active coping, self-motivations, self-advocacy and using resources/ Strong belief in adherence self-efficacy |  |  | [41], [44], [47], [52], [54], [41], [63], [64], [65], [67], [71], [72] |  |  |  |
| **Individual** | **Coping strategies** | Coping strategies for emotional regulation and self-management | Perceived behavioural control | Perceived behavioural control |  |  | [66] | [24], [26], [28] |  |  |
| **Individual** | **Coping strategies** | Coping strategies for emotional regulation and self-management | Positive attitude and life satisfaction | Positive attitude and life satisfaction |  |  | [64], [66], [68] | [7] |  |  |
| **Individual** | **Coping strategies** | Coping strategies for emotional regulation and self-management | Resilience | Resilience |  |  | [52] | [1], [4], [7], [12], [14], [1], [23], [24], [25], [27], [42], [43] |  |  |
| **Individual** | **Coping strategies** | Coping strategies for emotional regulation and self-management | Self-awareness | High levels of self-awareness (personal strengths and weaknesses and multiple factors contributing to life choices and trajectories) |  |  | [47], [52], [63], [64] | [7], [8], [23], [26] |  |  |
| **Individual** | **Coping strategies** | Coping strategies for emotional regulation and self-management | Want to take control of their health | Want to take control of their health |  |  | [47], [48], [63], [66] | [26] |  |  |
| **Individual** | **Coping strategies** | Coping strategies to recede side effects | Coping strategies to reduce side effects | Learned to manage HIV diagnosis and treatment/ Interpreting body and adjusting needs accordingly |  |  | [48], [67] | [27] |  |  |
| **Individual** | **Coping strategies** | Coping strategies to reduce side effects | Coping strategies to reduce side effects | Coping strategies to cope with adverse side effects such as drinking lots of fluids, resting to reduce dizziness and eating a snack before swallowing pills. |  |  | [61], [63] | [21], [23] |  |  |
| **Individual** | **Coping strategies** | Poor coping strategies | Poor coping strategies | Poor coping skills (such as using substances, lying about diagnosis, ascribing reasons for medications to other diseases, not taking medications to avoid disclosure) | [43] |  | [43], [68], [70], [73] | [44] |  |  |
| **Individual** | **Criminal justice system involvement** | Criminal justice system involvement | Involvement in the criminal justice system | Involvement in the criminal justice system |  |  | [48] |  |  |  |
| **Individual** | **Criminal justice system involvement** | Criminal justice system involvement | Repeated incarcerations | Repeated incarcerations |  |  |  |  | [2] |  |
| **Individual** | **Daily routine and lifestyle** | Daily routine and lifestyle | Change daily routine to integrate ART | Change daily routine to integrate ART |  | [54] | [41], [44], [47], [54], [63], [67], [70] | [1], [14], [41] |  |  |
| **Individual** | **Daily routine and lifestyle** | Daily routine and lifestyle | Life demands and organisational issues is disrupted by ART | Life demands and organisational issues is disrupted by ART/ not having time to attend the clinic due to busy lifestyle/ busy and distracted |  | [54] | [44], [47], [48], [63], [64], [65], [67], [70], [72] |  |  |  |
| **Individual** | **Daily routine and lifestyle** | Lifestyle | Unable to maintain healthy lifestyle | Unable to maintain healthy lifestyle |  |  | [54] |  |  |  |
| **Individual** | **Daily routine and lifestyle** | Sleeping | Sleeping | Sleeping |  |  | [47], [65], [70], [72] |  |  |  |
| **Individual** | **Daily routine and lifestyle** | Substance Use | Substance Use | Substance Abuse/IDU (aspects of illicit drug use and alcohol consumption. Regular use of more than one type of recreational drug significantly increases likelihood of non-adherence. | [43] |  | [43], [47], [48], [53], [55], [56], [57], [61], [64], [65], [68], [70], [71], [72] |  | [12] |  |
| **Individual** | **Desires** | Care for family and children | Being a parent and having children | Patient relying on child-rearing responsibilities can mitigate experiences of racism. |  |  | [51] | [11] |  |  |
| **Individual** | **Desires** | Care for family and children | Care for family and be healthy for children | Be healthy to care for and protect ones children (and prevent transmission for pregnant women) |  |  | [44] | [7], [14], [55], [23], [25], [41], [42], [43], [44] |  | [17], [44] |
| **Individual** | **Desires** | Care for family and children | Child Health | Desire for child to be healthy |  | [64] | [62], [64], [71] | [22], [24], [42] |  | [17] |
| **Individual** | **Desires** | Care for family and children | Child Health | Being pregnant and desire to reduce transmission to child |  | [64] | [62], [64], [67] | [22], [24], [27] |  | [17] |
| **Individual** | **Desires** | Look and feel healthy | Desire to be healthy and choosing 'life' over 'death' | Desire to be healthy and choosing 'life' over 'death' |  |  | [54], [55], [56], [62] | [14], [55], [16], [22] |  |  |
| **Individual** | **Desires** | Look and feel healthy | Desire to maintain appearance, stay healthy and keep status hidden | Desire to maintain appearance, stay healthy and keep status hidden (want to look healthy to others and prevent visible signs of the disease) |  |  | [47], [70] | [7] |  | [17] |
| **Individual** | **Desires** | Marriage and children | Desire for marriage and children | Desire for marriage and children |  | [73] | [63], [73] | [23] | [44] |  |
| **Individual** | **Desires** | Normalisation to life before ART | Normalisation to life before ART | Normalisation (feeling the same as others or same as before HIV) | [73] |  | [47], [65], [72], [73] | [7], [44] | [44] | [44] |
| **Individual** | **Disclosure** | Disclosure | Disclosure without stigma | Disclosure without stigma | [73] |  |  | [1], [55], [41], [44] |  | [44] |
| **Individual** | **Education and Training Skills** | Education and Training Skills | Goal setting and coping skills | Goal setting and coping skills |  |  |  | [2] |  |  |
| **Individual** | **Education and Training Skills** | Education and Training Skills | Nutrition education to help them optimise ART regimens and minimise side effects | Nutrition education to help them optimise ART regimens and minimise side effects |  |  |  | [2] |  |  |
| **Individual** | **Education and Training Skills** | Education and Training Skills | Private storage of medication | Devising ways of storing ART in obvious locations but in nondescript or containers |  |  |  | [2] |  |  |
| **Individual** | **Education and Training Skills** | Education and Training Skills | Stigma management and disclosure support education | Stigma management and disclosure support education | [73] |  |  | [2], [44] |  | [44] |
| **Individual** | **Education and Training Skills** | Education and Training Skills | Vocational training to promote livelihoods for those financially dependent on other family | Vocational training to promote livelihoods |  | [42] |  | [2] |  | [2] |
| **Individual** | **Education and Training Skills** | Education and Training Skills | Want to be kept abreast of new formulations of ART, new formulations and how to deal with missed doses and how to manage side effects | Want to be kept abreast of new formulations of ART, new formulations and how to deal with missed doses and how to manage side effects |  |  |  | [2] |  | [2] |
| **Individual** | **Education and Training Skills** | Experiences of HIV and ART | Healthy children responding to ART of HIV positive mother establishes role of mother in the family | Healthy children responding to ART of HIV positive mother establishes role of mother in the family |  |  |  | [55], [23] |  |  |
| **Individual** | **Experiences of HIV and ART** | Experiences of HIV and ART | Experienced an HIV-related illness and do not want to be ill again | Experienced an HIV-related illness and do not want to be ill again |  |  |  | [7] |  |  |
| **Individual** | **Experiences of HIV and ART** | Experiences of HIV and ART | Experiencing health benefits | Experiencing health benefits of taking ART |  |  |  | [21], [23], [41] |  |  |
| **Individual** | **Experiences of HIV and ART** | Experiences of HIV and ART | Having a child before the introduction of HAART | Having children before the introduction of HAART |  |  | [64] |  |  |  |
| **Individual** | **Experiences of HIV and ART** | Experiences of HIV and ART | Knowing others with HIV accessing care | Knowing others who have HIV and on ART and felt healthier |  |  |  | [14], [23], [27] |  |  |
| **Individual** | **Experiences of HIV and ART** | Experiences of HIV and ART | Knowing others with HIV who died | Knowing others who had HIV and died, want to be healthier and live because of their experiences |  |  |  | [14] |  |  |
| **Individual** | **Experiences of HIV and ART** | Experiences of HIV and ART | Low perception of need for treatment/insignificance of experienced HIV illness | Low perception of need for treatment/insignificance of experienced HIV illness/ No instant consequences for non-adherence | [50], [53] |  |  | [14] | [10] |  |
| **Individual** | **Experiences of HIV and ART** | Experiences of HIV and ART | Negative experiences with ART | Makes patient feel different to peers |  |  | [41] |  |  |  |
| **Individual** | **Fears** | Fear of declining physical health | Becoming ill again | Fear of returning to pre ART health state |  |  |  | [14] |  |  |
| **Individual** | **Fears** | Fear of economic loss related to treatment | Fear of job loss | Fear of job loss | [55] |  |  |  |  |  |
| **Individual** | **Fears** | Fear of reason for positive HIV diagnosis | Cause of HIV | Fear of cause of HIV | [53] |  | [47] |  |  |  |
| **Individual** | **Fears** | Fear of the future | Fear of the unknown and future | Fear of the unknown and inability to formulate expectations | [50] |  | [63] |  |  |  |
| **Individual** | **Fears** | Fears of stigma | Consequences of disclosure | Fear of disclosure will lead to stigma and discrimination | [43], [49], [50], [53], [64], [73] |  | [41], [43], [55], [56], [63], [64], [65], [67], [70], [73] |  | [17], [44] |  |
| **Individual** | **Fears** | Fears of stigma | Isolation | Fear of isolation | [53], [55], [73] |  | [55], [73] |  | [55], [44] |  |
| **Individual** | **Fears** | Fears of stigma | Marginalisation | Marginalisation of IDU intensified for HIV positive patients |  |  | [56] |  |  |  |
| **Individual** | **Fears** | Fears of stigma | Perceived or actual stigma | Anticipated HIV stigma and concealment (not wanting others to know about status, may see taking medication | [50], [53], [64], [66], [73] |  | [41], [47], [48], [50], [52], [54], [55], [64], [68], [70], [71], [72], [73] |  | [10], [55], [17], [26], [44] |  |
| **Individual** | **Fears** | Fears of stigma | Perceived or actual stigma | Unwanted stigma and disclosure when taking medication | [43] |  | [43], [67], [68], [70], [72], [73] | [1] | [44] |  |
| **Individual** | **Fears** | Fears related to the effects of ART | ART is harmful to unborn babies | Fear that treatment would have a negative impact on children or harm children | [60], [64] |  | [55], [62], [64], [71] |  |  |  |
| **Individual** | **Fears** | Fears related to the effects of ART | Being on ARVs | Fear of being on ARVS | [53] |  |  |  |  |  |
| **Individual** | **Fears** | Fears related to the effects of ART | Drug resistance | Fear of drug resistance |  |  | [72] |  |  |  |
| **Individual** | **Fears** | Fears related to the effects of ART | Fear that ART leads to impotency, infertility and impossibility of sexual activity | Fear that ART leads to impotency, infertility and impossibility of sexual activity |  |  | [63] |  |  |  |
| **Individual** | **Fears** | Fears related to the effects of ART | Negative beliefs about benefits of taking ART and resistance | Fear of drug toxicities | [53], [64] |  | [64], [65], [70] |  |  |  |
| **Individual** | **Fears** | Fears related to the effects of ART | Side effects | Fear of side effects | [53] |  | [60], [65], [70] |  | [17] |  |
| **Individual** | **HIV Status** | Acceptance of HIV status | Acceptance of HIV status | Acceptance of HIV status |  |  |  | [4], [7], [8], [14], [23], [25], [41] |  |  |
| **Individual** | **HIV Status** | Non-acceptance of HIV status | Non-acceptance of HIV status | Non-acceptance (Avoidance, denial of ones HIV status, diagnosis and what it entails) | [50], [53] |  | [41], [47], [48], [54], [55], [56], [65] |  | [55], [17] |  |
| **Individual** | **Knowledge and understanding** | Knowledge of HIV Status | Knowledge of HIV status | Knowledge of HIV status |  |  |  | [1], [44] |  |  |
| **Individual** | **Knowledge and understanding** | Knowledge of HIV Status | Not knowing HIV status is reason for medication | Not knowing reasons for taking drugs (Lack of prior knowledge of status before being given ART made children resistant) |  |  | [41], [61], [65], [73] |  |  |  |
| **Individual** | **Knowledge and understanding** | Knowledge of HIV, ART and HAART | Experiences with ART improves knowledge | Long term experience with ART leads to more knowledge and strategies to incorporate ART into lifestyle |  |  |  | [7], [14], [41] |  |  |
| **Individual** | **Knowledge and understanding** | Knowledge of HIV, ART and HAART | High knowledge of HIV, HAART, PMTCT and referral processes | High knowledge of HIV, HAART, PMTCT and referral processes |  |  |  | [55], [27] |  | [55] |
| **Individual** | **Knowledge and understanding** | Knowledge of HIV, ART and HAART | Knowledge of ART benefits | Understanding of the benefits of taking ART, drug reactions and how to manage side effects |  |  |  | [1], [2], [21], [24] |  |  |
| **Individual** | **Knowledge and understanding** | Knowledge of HIV, ART and HAART | Low knowledge of HIV and HAART | Lack of Knowledge (pertains to having insufficient or incorrect knowledge of ART and adherence, including having unanswered questions, being faced with conflicting information and difficulty understanding | [43], [49], [50], [53], [64] | [54] | [43], [47], [65], [67], [72] |  |  |  |
| **Individual** | **Knowledge and understanding** | Knowledge of HIV, ART and HAART | More sources of information about HIV services | Media channels providing information about HIV services | [66], [73] |  |  | [44] |  | [26], [44] |
| **Individual** | **Knowledge and understanding** | Knowledge of HIV, ART and HAART | Understanding importance of adherence | Understand need for compliance |  |  |  | [2], [25] |  |  |
| **Individual** | **Knowledge and understanding** | Uncertainty and conflicting messages | Conflicting messages from religious leaders and community leaders. | Religious beliefs and spirituality (barrier: belief in religious cures for HIV over conventional medicine and conflicting messages from community leaders | [53] |  | [41], [54], [55], [63], [67], [71] | [1], [55], [23] | [55] | [55], [17] |
| **Individual** | **Knowledge and understanding** | Uncertainty and conflicting messages | Local traditions, beliefs and medicines | Uncertain about the pros and cons of traditional medication and how to respect for local traditions and beliefs while using Western medicine |  |  | [41], [63] |  |  |  |
| **Individual** | **Knowledge and understanding** | Uncertainty and conflicting messages | Uncertain of long term effects | Uncertainty of long term effects |  |  | [65], [70] |  |  |  |
| **Individual** | **Knowledge and understanding** | Uncertainty and conflicting messages | Uncertainty about onset of labour | Uncertainty about the onset of labour in order to swallow NVP |  |  |  |  |  |  |
| **Institutional** | **Knowledge and understanding** | Uncertainty and conflicting messages | Adolescents are uncertain about services | Adolescents are uninformed about service cost and location |  |  | [73] |  | [44] |  |
| **Individual** | **Medication** | Being away from home | Travelling interrupting treatment | Carrying ART while out of the house/travelling. Delaying doses (when traveling or entertaining. Some take a treatment holiday) |  |  | [41], [47], [54], [55], [65], [70], [72], [73] | [7] |  |  |
| **Individual** | **Medication** | Forgetting and misplacing medication | Forgetfulness (Study 14: due to using alcohol) | Forgetfulness (Study 14: due to using alcohol) | [55] |  | [41], [47], [54], [55], [58], [64], [65], [70], [72] |  |  |  |
| **Individual** | **Medication** | Medication characteristics | Type, palatability, smell and colour | Type, palatability, smell, colour and in tablet form |  |  | [41], [47], [48], [56], [65], [69], [70], [73] | [1] |  |  |
| **Individual** | **Medication** | Medication reminders | Use of reminders | Use of reminders (watches, clocks and mobile phones) |  |  |  | [1], [7], [14], [55], [25], [41], [43], [44] |  |  |
| **Individual (this row must be added to table)** | **Medication** | Negative side effects of medication | Physical manifestations of HIV and AIDS may lead to isolation and belief that the person is already dead | Physical manifestations of HIV and AIDS may lead to isolation and belief that the person is already dead |  |  | [48], [56] |  |  |  |
| **Individual** | **Medication** | Negative side effects of medication | Negative side effects | Anticipated side effects |  |  | [60], [65] |  |  |  |
| **Individual** | **Medication** | Negative side effects of medication | Negative side effects | Unpleasant side effects |  |  | [41], [43], [48], [54], [65], [66], [69], [70], [72] |  |  |  |
| **Individual** | **Medication** | Negative side effects of medication | Negative side effects | Adverse reactions with EFV containing regimens (intense body heat, delusions, anxiety, intense dizziness and nightmares: pain and suffering |  |  | [61], [65], [70], [73] |  | [44] |  |
| **Individual** | **Medication** | Negative side effects of medication | Negative side effects | Body changes, buffalo hump, excess sweating, darkening of the skin led to low self-esteem and taking longer treatment holidays |  |  | [61], [70] |  |  |  |
| **Individual** | **Medication** | Negative side effects of medication | Negative side effects | Side effects (increased with taking ART without food) such as diarrhoea and changing body, buffalo hump, excess sweating, darkening of the skin, body door, hair loss, weight loss/gain, skin rash. Fear that this may lead to involuntary disclosure | [45], [64] |  | [41], [44], [47], [48], [54], [55], [60], [61], [62], [64], [65], [66], [67], [68], [70], [72], [73] |  | [17], [44] |  |
| **Individual** | **Medication** | No privacy when taking pills | Lack of privacy | Dispensing windows in prisons may cause accidental disclosure |  |  | [42] |  |  |  |
| **Individual** | **Medication** | No privacy when taking pills | Lack of privacy | Lack of private spaces to take medication (for children at boarding schools and at home) |  |  | [41], [47], [70] |  |  |  |
| **Individual** | **Medication** | Pill burden and regimen | Pill burden and regimen | Change in medication or treatment |  |  | [70] |  |  |  |
| **Individual** | **Medication** | Pill burden and regimen | Pill burden and regimen | Dietary Instructions |  |  | [54], [70] |  |  |  |
| **Individual** | **Medication** | Pill burden and regimen | Pill burden and regimen | Duration of treatment | [64] |  | [43], [54], [68], [72] |  |  |  |
| **Individual** | **Medication** | Pill burden and regimen | Pill burden and regimen | Pill burden (quantity and size)/regimen complexity | [64] |  | [41], [43], [44], [47], [48], [54], [55], [56], [62], [64], [65], [68], [69], [70], [72], [73] | [4] | [17] |  |
| **Individual** | **Medication** | Pill burden and regimen | Pill burden and regimen | Middle of the day/early morning dose difficult to maintain |  |  | [65] |  |  |  |
| **Individual** | **Medication** | Pill burden and regimen | Pill burden and regimen | Simple regimen |  |  |  | [2], [25], [28], [42], [44] |  |  |
| **Individual** | **Medication** | Reminder of status | Unwanted reminder of disease | Denial and Taking medication is an unwanted reminder of disease | [43] | [65] | [43], [70] |  |  |  |
| **Individual** | **Medication** | Skipping medication | Avoiding medication to avoid disclosure | Skip medication to avoid disclosure |  |  | [41], [70] |  |  |  |
| **Individual** | **Medication** | Skipping medication | Taking medication breaks to rid body of toxins and relieve side effects temporarily | Taking medication breaks to rid body of toxins and relieve side effects temporarily |  |  | [70] |  |  |  |
| **Individual** | **Past trauma and abuse** | Experienced past trauma or abuse | Experienced trauma | Trauma |  |  | [54] |  |  |  |
| **Individual** | **Past trauma and abuse** | Experienced past trauma or abuse | Psychological abuse | Psychological abuse |  |  |  |  | [12] |  |
| **Individual** | **Past trauma and abuse** | Experienced past trauma or abuse | Sexual abuse | Sexual abuse |  |  | [68] |  |  |  |
| **Individual** | **Past trauma and abuse** | Experienced past trauma or abuse | Suicidal | Prior suicidal attempt or feeling suicidal | [73] |  | [68], [73] |  | [44] |  |
| **Individual** | **Physical health** | Comorbidities | Living with comorbidities and drug interactions | Comorbidities and drug interactions: Including wanting to complete TB treatment first and coping with multiple diseases and ailments barrier. Co-infections of HPV facilitator. | [43], [53] | [54], [64] | [43], [48], [55], [65] | [55] |  |  |
| **Individual** | **Physical health** | Feeling better and healthier | Feeling better | Physical benefits associated with taking medication |  |  | [43], [61], [63], [67] | [21], [23], [27] |  |  |
| **Individual** | **Physical health** | Feeling better and healthier | Feeling better | Regaining life, strength and sexual power. Improved body image and attractiveness |  |  | [63] | [23] |  |  |
| **Individual** | **Physical health** | Feeling better and healthier | Feeling better and being able to work again | Being able to work again |  |  | [47], [54] | [7], [14] |  |  |
| **Individual** | **Physical health** | Feeling better and healthier | Feeling better and not wanting further care | Feeling better or healthier/seeing positive results, could also lead to people feeling like they have been cured. | [50] | [54] | [47], [54], [63], [65], [70], [72] | [7], [14], [23], [25], [41], [43] | [10] |  |
| **Individual** | **Physical health** | Feeling better and healthier | Feeling better and not wanting further care | HIV infected but asymptomatic, the successful management of the disease due to being in care led patients to question the necessity of repeated medical tests and procedures (Feeling better) | [50], [53], [55] |  | [41], [47], [54], [63], [65], [72], [73] | [7], [23] | [10], [17] |  |
| **Individual** | **Physical health** | Feeling ill and disease progression | Disease Progression | Disease progression. Feeling worse |  |  | [67], [68], [73] |  |  |  |
| **Individual** | **Physical health** | Feeling ill and disease progression | Experiences of HIV related symptoms | Experience of HIV related symptoms |  |  | [63] | [23] |  |  |
| **Individual** | **Physical health** | Feeling ill and disease progression | Feeling sick and decreasing quality of health | Feeling sick or ill/ impairments of general health/quality of life decreasing |  | [50], [53] | [47], [64], [65] |  |  | [10] |
| **Individual** | **Physical health** | Feeling ill and disease progression | Low CD4 count | Low CD4 count or advanced immunodeficiency |  | [54] | [54] | [14] |  |  |
| **Individual** | **Physical health** | Feeling ill and disease progression | When feeling ill take medication | Feeling ill. Seeking healthcare is a necessity. | [64] | [64] | [73] |  | [17] |  |
| **Individual** | **Psychological distress and emotional reactions** | Demotivated | Lack of commitment | Not feeling ready to commit or intimidated by lifelong treatment (will have lifelong financial implications | [43], [49], [53], [55] |  | [63], [70], [71] |  | [17] |  |
| **Individual** | **Psychological distress and emotional reactions** | Demotivated | Lack of motivation | Lack of motivation to adhere; desire to control one’s life, disease, and or pill taking. |  |  | [47], [48], [63], [72] |  |  |  |
| **Individual** | **Psychological distress and emotional reactions** | Negative emotion | Feeling lonely | Feeling lonely |  |  | [47] |  |  |  |
| **Individual** | **Psychological distress and emotional reactions** | Negative emotion | Feeling low or depressed | Depression (study 14: feeling ready to die/Mental health problems. Feeling low mood, hopeless and down | [43], [73] |  | [41], [43], [44], [47], [54], [63], [64], [65], [68], [70], [71], [72] | [14], [44] | [12], [44] |  |
| **Individual** | **Psychological distress and emotional reactions** | Negative emotion | Feeling shame | Shame associated with specific transmission modes | [50] |  |  |  |  |  |
| **Individual** | **Psychological distress and emotional reactions** | Negative emotion | Frustration around lack of independence | Frustration around lack of independence |  |  | [73] |  | [44] |  |
| **Individual** | **Psychological distress and emotional reactions** | Negative emotion | Negative attitude towards treatment regimen | Negative attitude towards treatment regimen |  |  | [47], [70], [72] |  | [17] |  |
| **Individual** | **Psychological distress and emotional reactions** | Perception of self | Doubt self-efficacy | Doubt ability to adhere | [73] |  | [65], [73] | [44] | [44] | [44] |
| **Individual** | **Psychological distress and emotional reactions** | Perception of self | Self-efficacy and self-worth | Self-image and identity | [50] |  | [41], [71] |  |  |  |
| **Individual** | **Psychological distress and emotional reactions** | Perception of self | Self-efficacy and self-worth | Self-esteem and self-worth as an infected wife and mother | [60] |  |  |  |  |  |
| **Individual** | **Psychological distress and emotional reactions** | Perception of self | Self-stigma | Internalised stigma when taking medication |  |  | [47], [54], [73] |  | [44] |  |
| **Individual** | **Psychological distress and emotional reactions** | Psychological distress and emotional impact | Emotional impact | Emotional impact |  |  | [44] |  |  |  |
| **Individual** | **Psychological distress and emotional reactions** | Psychological distress and emotional impact | Mental treatment fatigue | Mental fatigue from being engaged in care and treatment fatigue | [50] |  | [41], [73] |  | [10] |  |
| **Individual** | **Psychological distress and emotional reactions** | Psychological distress and emotional impact | Psychological state on diagnosis | Psychological state at diagnosis | [50] |  | [67] |  |  |  |
| **Individual** | **Psychological distress and emotional reactions** | Psychological distress and emotional impact | Psychological state on diagnosis | Shock from unexpected results and revealed infidelity issues in their primary relationships | [50], [64] |  | [67] |  |  |  |
| **Individual** | **Psychological distress and emotional reactions** | Psychological distress and emotional impact | Psychological suffering and stress | Psychological suffering (being reminded of being sick, delusional, fear of being killed by taking drugs, getting mad) |  |  | [54], [61], [63], [67], [70] | [14] | [10] |  |
| **Individual** | **Psychological distress and emotional reactions** | Psychological distress and emotional impact | Wants to maintain control | Wants to maintain control |  |  | [65] |  |  |  |
| **Individual** | **Sociodemographic** | Age | Age | Being older (for women it means more responsibilities, in women having children)/ in adolescents over the age of 15 years | [64] |  | [41], [54], [55], [71] | [55], [42] |  |  |
| **Individual** | **Sociodemographic** | Age | Age | Younger age linked to lower adherence | [43] |  | [43], [54], [55], [68], [71] | [28] |  |  |
| **Individual** | **Sociodemographic** | Education | Education level | Higher levels of education |  |  | [54], [55] | [14], [55], [28] |  |  |
| **Individual** | **Sociodemographic** | Education | Education level | Lower levels of education |  |  | [54], [55], [68] |  |  |  |
| **Individual** | **Sociodemographic** | Education | Education level | Being in school |  |  |  | [28] |  |  |
| **Individual** | **Sociodemographic** | Education | Education level | Repeating a grade in school |  |  | [68] |  |  |  |
| **Individual** | **Sociodemographic** | Employment | Employment | Having employment |  |  | [54] | [14], [26] |  |  |
| **Individual** | **Sociodemographic** | Employment | Employment | Lacking or insecure employment | [43] |  | [43], [48], [54] |  | [12] |  |
| **Individual** | **Sociodemographic** | Employment | Type of occupation | Occupation (such as sex work. Participants felt they had to leave their jobs in order to adhere as using substance and alcohol during work) | [45] |  |  | [18] | [18] |  |
| **Individual** | **Sociodemographic** | Gender | Gender | Gender | [60], [64] |  | [43], [48], [54], [64], [68] | [14], [28] |  |  |
| **Individual** | **Sociodemographic** | Gender | Gender | Being male |  |  | [41], [43], [54] | [14] |  |  |
| **Individual** | **Sociodemographic** | Gender | Gender | Female gender: in some places female gender did not influence willingness to engage and in some cases women’s status was seen as a result of partners infidelity, in other places women is seen as hyper sexual | [60], [64] |  | [54], [63], [64] | [14] |  |  |
| **Individual** | **Sociodemographic** | Marital status | Marital Status | Marital status |  |  | [54] |  |  |  |
| **Individual** | **Sociodemographic** | Race/nationality | Race/nationality | Immigration Status |  |  | [48] |  |  |  |
| **Individual** | **Sociodemographic** | Race/nationality | Race/nationality | Race/being in a minority group |  |  | [48], [55] |  |  |  |
| **Individual** | **Sociodemographic** | Sexual partners | Number sexual partners | Number of sexual partners |  |  | [54] |  |  |  |
| **Individual** | **Sociodemographic** | Identification | Identification | Being undocumented | [66] |  | [66] |  | [26] |  |
| **Individual** | **Sociodemographic** | Identification | Identification | Fear of deportation | [66] |  | [66] |  | [26] |  |
| **Individual** | **Sociodemographic** | Identification | Identification | Inability to provide documentation for care and lack of health insurance | [66] |  | [66] |  | [26] |  |
| **Individual** | **Spiritual beliefs** | Beliefs: Spiritual | Belief in a higher power | Belief in Gods will and relinquishing control of their lives |  |  |  | [16] |  |  |
| **Individual** | **Spiritual beliefs** | Beliefs: Spiritual | Belief in a higher power | Belief and faith that God provided the knowledge to make ART |  |  |  | [7], [14] |  |  |
| **Individual** | **Spiritual beliefs** | Beliefs: Spiritual | Belief in a higher power | Prayers or faith in God |  |  |  | [7], [14], [23] |  |  |
| **Individual** | **Spiritual beliefs** | Beliefs: Spiritual | Belief in a higher power | Religious fasting |  |  | [41] |  |  |  |
| **Individual** | **Spiritual beliefs** | Beliefs: Spiritual | Belief in a higher power | Religious fasting period needs to adjust dosing time regiments |  |  |  | [1] |  |  |
| **Individual** | **Spiritual beliefs** | Beliefs: Spiritual | Belief in a higher power | Religious beliefs or treatments. |  |  | [47], [61], [71] | [14], [42] | [55], [17] |  |
| **Individual** | **Stigma and discrimination** | Experiences of stigma | Stigma about mode of transmission | Mode of transmission is not regarded as socially acceptable behaviour |  |  | [56] |  |  |  |
| **Individual** | **Traditional Beliefs** | Beliefs: Traditional | Cultural attitudes and beliefs | Cultural attitudes and beliefs | [43], [50], [53], [73] | [43] | [67], [73] |  | [44] |  |
| **Individual** | **Traditional Beliefs** | Beliefs: Traditional | Traditional beliefs and alternate medicines | Alternate medication can reduce side effects of ART | [43], [50], [53], [73] | [43] |  | [24] |  |  |
| **Individual** | **Traditional Beliefs** | Beliefs: Traditional | Traditional beliefs and alternate medicines | Traditional beliefs or medicines may be superior that science-based medicines | [50], [53], [64] |  | [47], [63], [64], [67] |  |  |  |
| **Individual** | **Coping strategies** | Coping strategies for emotional regulation and self-management | Staying away from negativity | Staying away from negative relationships |  |  | [47], [54] | [7], [12], [23], [24] |  |  |
| **Individual** | **Coping strategies** | Coping strategies for emotional regulation and self-management | Staying away from negativity | Avoiding internalised self-stigma |  |  | [44] | [4] |  |  |
| **Interpersonal** | **Caregiver Factors** | Access to caregivers | Two or more caregivers | Having two or more caregivers |  |  |  | [1] |  |  |
| **Interpersonal** | **Caregiver factors** | Caregiver beliefs | Caregiver Factors | Caregivers instil fear and perceived stigma and discrimination |  |  | [41] |  |  |  |
| **Interpersonal** | **Caregiver factors** | Caregiver beliefs | Caregiver wants to preserve family and normality | Caregiver wants to preserve normality or prioritising a normal life for the child |  |  | [69] |  |  |  |
| **Interpersonal** | **Caregiver Factors** | Caregiver beliefs | Competing beliefs of caregiver | Caregiver has competing beliefs and concerns regarding treatment and the condition itself (Caregiver wants to preserve family relationships and conflict between caregiver and child on beliefs about treatment) | [53] |  | [67], [69] |  |  |  |
| **Interpersonal** | **Caregiver Factors** | Caregiver Disclosure | Caregiver disclosure to child after the age of 12 years | Late disclosure |  |  | [41] |  |  |  |
| **Interpersonal** | **Caregiver Factors** | Caregiver Disclosure | Caregiver disclosure to child before the age of 12 years | Early disclosure of status, before age 12 |  |  |  | [1] |  |  |
| **Interpersonal** | **Caregiver Factors** | Caregiver Disclosure | Denial of child’s status | Caregiver denial of child status (especially in perinatally infected youth) and does not want to disclose | [53] |  |  | [1] |  |  |
| **Interpersonal** | **Caregiver Factors** | Caregiver education | Caregiver education | Education level of caregiver |  |  | [55], [68] | [28] |  |  |
| **Interpersonal** | **Caregiver Factors** | Caregiver education | Caregiver education | Caregiver perception of child adherence |  |  |  |  |  |  |
| **Interpersonal** | **Caregiver Factors** | Caregiver reminders | Caregiver reminders | Caregiver support (reminding to take medication and accompanying to clinic) |  |  | [65] | [1], [25] |  |  |
| **Interpersonal** | **Caregiver Factors** | Relation to caregiver | Caregiver is not biological parent | Caregiver is someone else other than biological parent |  |  |  | [28] |  |  |
| **Interpersonal** | **Competing life demands** | Competing life demands | Competing family life demands | Needs of one’s family is prioritised over health needs (such as finance, food, school fees). Competing priorities and role strain (especially for women) | [50], [53], [60], [66] |  | [52], [55], [65], [71] |  | [10], [12], [26] |  |
| **Interpersonal** | **Competing life demands** | Competing life demands | Needs of workplace are prioritised over health needs in order to be financial secure causes difficulty when managing multiple daylong clinic visits. | Needs of workplace are prioritised over health needs in order to be financial secure causes difficulty when managing multiple daylong clinic visits. | [50] |  |  |  | [10] |  |
| **Interpersonal** | **Disclosure** | Disclosure | Disclosure | Disclosure to family members | [50], [73] |  | [41], [73] | [1], [7], [14], [27], [42], [43], [44] | [10], [44] | [10] |
| **Interpersonal** | **Disclosure** | Disclosure | Disclosure to partner | Disclosure to partner |  |  | [41] | [1], [14], [55], [27], [43] | [55] | [55] |
| **Interpersonal** | **Disclosure** | Disclosure | Disclosure with stigma | Post disclosure stigma | [73] |  | [41], [63], [67], [73] |  | [44] |  |
| **Interpersonal** | **Disclosure** | Disclosure | Family and friends reactions to an individual’s HIV positive diagnosis. | Family and friends reactions to an individual’s HIV positive diagnosis. | [50], [73] |  | [41[, [73] | [41[, [44] | [10], [44] | [10], [44] |
| **Interpersonal** | **Disclosure** | Non-disclosure | Non-disclosure to family | Non-disclosure to other family members (for women were at risk of spousal or family rejection , withdrawal from financial support or expulsion from home leading to sporadic care with health care system | [50], [53], [60], [73] |  | [41], [56], [64], [70], [71], [73] |  | [10], [44] |  |
| **Interpersonal** | **Disclosure** | Non-disclosure | Non-disclosure to partner | Non-disclosure of HIV status to women’s sexual partner to avoid conflict | [49], [53], [55] |  | [63], [71] |  |  |  |
| **Interpersonal** | **Disclosure** | Non-disclosure | Non-disclosure to peers | HIV nondisclosure to peers |  |  | [41], [47], [70] |  |  |  |
| **Interpersonal** | **Family on ART** | Family on ART | Other family members on ART | Other family members on ART |  |  |  | [1] |  |  |
| **Interpersonal** | **Family on ART** | Family on ART | Sharing and selling ART | Sharing and selling ART |  |  | [47] |  |  |  |
| **Interpersonal** | **Family on ART** | Family on ART | Sharing medication with others | Sharing medication with others (partner, family or friends) |  |  | [54], [67] | [14] |  |  |
| **Interpersonal** | **Family on ART** | Family on ART | Stealing pills | Relatives stealing ART pills |  |  | [55], [67] |  |  |  |
| **Interpersonal** | **Relationships in household** | Conflict and tension in family relationships | Accused of infecting others | Being accused of infecting others |  |  | [65] | [14] |  |  |
| **Interpersonal** | **Relationships in household** | Conflict and tension in family relationships | Family perceives HIV positive patient as an economic burden and wants legitimate reasons and why extra needs must be met | Family perceives HIV positive patient as an economic burden and wants legitimate reasons and why extra needs must be met |  |  | [63] |  |  |  |
| **Interpersonal** | **Relationships in household** | Conflict and tension in family relationships | Negative relationships will conflict | Poor and conflict in relationships with family | [43], [44] |  | [43], [52], [72] |  |  |  |
| **Interpersonal** | **Relationships in household** | Gender and power in household | Allocation of ARVs to the provider in the house | ARVs are allocated to the provider in the house, sometimes cost does not allow for more than one person to be on ART: social triage |  |  | [63] |  |  |  |
| **Interpersonal** | **Relationships in household** | Gender and power in household | Dependent on partner, lack of autonomy | Sex (wives have lack of autonomy) | [53] |  | [47], [46], [63], [71] |  |  |  |
| **Interpersonal** | **Relationships in household** | Gender and power in household | Dependent on partner, lack of autonomy | Permission needed from or dependence on partner (women cannot seek treatment or travel alone to the clinic in some cultures) | [55] |  | [55], [56], [67], [71] |  | [55], [17] |  |
| **Interpersonal** | **Relationships in household** | Gender and power in household | Fear of domestic violence and abandonment | Fear of domestic violence or abandonment after disclosure: for men and women | [50], [53] |  | [56], [63], [67], [71] |  | [10], [55], [17] |  |
| **Interpersonal** | **Relationships in household** | Gender and power in household | Men have decision making power over resources in household | Women often not entitled to decide over use of resources. Men may deny women ARVs. |  |  | [63] |  |  |  |
| **Interpersonal** | **Relationships in household** | Supportive family relationships | Family mitigating discrimination by support | Experiences of discrimination can be mitigated by patient relying on strong family support |  |  |  | [11] |  |  |
| **Interpersonal** | **Relationships in household** | Supportive family relationships | Family supporting | Social interaction, support and relationships (supportiveness, quality and presence of personal relationship and interactions with others | [73] |  |  | [8], [23], [25], [44] |  | [12] |
| **Interpersonal** | **Relationships in household** | Supportive family relationships | Mutuality-fostering relationships | Mutuality-fostering relationships involving reciprocal care and empathy, providing a sense of family | [44], [50], [73] |  |  | [12], [55], [16], [23], [25], [41], [43], [44] | [10] | [10] |
| **Interpersonal** | **Relationships in household** | Supportive family relationships | Reminder to take medication | Family supporting by assisting with medication reminders |  |  |  | [44] |  |  |
| **Interpersonal** | **Relationships in household** | Supportive partner | Intimate partner | Partner involved in care (assist with reminding to take medication and sending reminders through cell phone when not present) |  |  |  | [27], [41] |  |  |
| **Interpersonal** | **Relationships in household** | Supportive partner | Intimate partner | Partner emotional support | [58] |  |  | [18] |  | [18] |
| **Interpersonal** | **Relationships in household** | Supportive partner | Intimate partner | Partner financial support (money to go to the clinic, money for ART, through providing transport, accompanying to doctors’ visits and couples counselling) | [58] |  |  | [18], [27] |  | [18] |
| **Interpersonal** | **Relationships in household** | Supportive partner | Partner support healthier lifestyle | Partner encouraging to live healthy, eat healthy, exercise and reduce alcohol use | [58] |  |  | [18] |  | [18] |
| **Interpersonal** | **Relationships in household** | Unsupportive family relationships | Family not supporting | No family support | [43], [50], [55] |  | [41], [43], [55], [65], [70], [72] |  | [10], [17] |  |
| **Interpersonal** | **Relationships in household** | Unsupportive family relationships | Family not supporting | Neglect or orphan hood | [73] |  | [73] |  | [44] |  |
| **Interpersonal** | **Relationships in household** | Unsupportive family relationships | Family not supporting | Punishment for adherence slips leads to dishonesty |  |  | [73] |  |  |  |
| **Interpersonal** | **Relationships in household** | Unsupportive family relationships | Negative experiences of stigma in family | Stigma associated with a child’s HIV status: Maternal shame and stigma related to perinatal acquisition |  |  | [54], [56] |  |  |  |
| **Interpersonal** | **Relationships in household** | Unsupportive family relationships | Negative experiences of stigma in family | Stigma from family/friends | [50] |  | [68], [70], [72], [73] |  | [10], [44] |  |
| **Interpersonal** | **Relationships in household** | Unsupportive partner | Intimate partner | Partner on different schedule to take medication of discouraging about taking meds |  |  | [70] |  |  |  |
| **Interpersonal** | **Relationships in household** | Unsupportive partner | Not involved in care | Partner not involved in care | [55] |  |  |  | [55], [17] | [55] |
| **Community** | **Community beliefs and practices** | Beliefs about HIV and ART | Long-held beliefs and shared Community views such as witchcraft, hospitals are places of death | Long-held beliefs and shared community views such as HIV as witchcraft, hospitals are places of death | [50] |  | [63], [64] |  | [10] |  |
| **Community** | **Community beliefs and practices** | Beliefs about HIV and ART | Positive community beliefs about HIV and ART | Positive community beliefs about HIV and ART |  |  |  | [27] |  |  |
| **Community** | **Community beliefs and practices** | Gender norms | Masculinity | Masculinity | [50] |  | [54], [63] | [14] | [10] |  |
| **Community** | **Community beliefs and practices** | Patient lacks autonomy | Lack of autonomous decision making in childbirth for women in rural villages | Lack of autonomous decision making in childbirth for women in rural villages |  |  | [55] |  |  |  |
| **Community** | **Community beliefs and practices** | Patient lacks autonomy | Negative community beliefs about HIV and ART | Negative community beliefs about HIV and ART (Patient has no choice) |  |  | [67] |  |  |  |
| **Community** | **Community beliefs and practices** | Preference for traditional healers and medicines | Bypassing clinic or hospital services for traditional healers. | Bypassing clinic or hospital services for traditional healers (also reported as cheaper) | [50] |  | [63] |  | [10] | [44] |
| **Community** | **Community beliefs and practices** | Supportive traditions | Community traditions supporting patient | Experiences of discrimination can be mitigated by patient relying on cultural and religious traditions |  |  |  | [11] |  |  |
| **Community** | **Financial support** | Financial support | Community financial support | Financial support from social networks to cover expenses, this creates obligation to adhere |  |  |  | [23], [41], [43] |  |  |
| **Community** | **Peers and support groups** | Medication companion | Having a treatment buddy | Having a treatment buddy | [73] |  | [73] | [14], [23] |  | [44] |
| **Community** | **Peers and support groups** | Medication companion | Identification of a confidante | Identification of a confidante (at work, school or within network) | [73] |  |  | [4], [44] |  | [44] |
| **Community** | **Peers and support groups** | Medication companion | Medication companions and treatment partners | Medication companions and treatment partners | [42] |  |  | [2] |  |  |
| **Community** | **Peers and support groups** | Medication companion | Unable to identify a treatment buddy | Unable to identify a treatment buddy | [73] |  |  |  |  |  |
| **Community** | **Peers and support groups** | Peer support | Peer support helped with new normal | Peer support helped with new normal and acted as a proxy for lack of family support | [42] |  |  | [1], [2] |  | [2] |
| **Community** | **Peers and support groups** | Social isolation | Social Isolation | Social Isolation and inadequate community/social support |  |  | [44], [65] |  |  |  |
| **Community** | **Peers and support groups** | Support groups | Peer and support groups | Attending support group allowed patients to share wisdom and attain inner peace/ Support groups, peer support, treatment partners, medication companions helped with linkage, feelings of belongingness and reduce stigma | [44], |  |  | [7], [44] |  | [17], [44] |
| **Community** | **Peers and support groups** | Supportive supervisors and teachers | Support to take ART | Supervisors and teachers should be enlisted to facilitate workers and students taking medication with minimal disruption | [73] |  |  | [44] |  | [44] |
| **Community** | **Peers and support groups** | Supportive supervisors and teachers | Support to take ART | Supervisors and teachers be enlisted to assist with adherence and non-disclosure | [73] |  |  | [2], [44] |  | [44] |
| **Community** | **Peers and support groups** | Unsupportive supervisors and teachers | Supervisors and teachers | Enacted stigma by teachers | [73] |  | [73] |  | [44] |  |
| **Community** | **Religious institutions** | Religious institutions | Religious institutions | Support from religious institutions were helpful when family was not supportive. For prisoners helped to counter rejection, violence and abandonment | [50], [73] |  |  | [24], [44] |  | [10], [44] |
| **Community** | **Social support** | Social support | Family supporting | Material, emotional and social support helps to overcome stigma | [44], [53] |  |  | [7], [12], [14], [16], [23], [25], [41], [42] |  | [12] |
| **Community** | **Stigma and discrimination** | Experiences of stigma | Cannot find work due to stigma | Economic insecurity because of HIV related stigma: unable to find a job | [45] |  |  |  |  |  |
| **Community** | **Stigma and discrimination** | Experiences of stigma | Negative experiences of stigma and discrimination | Multilevel encounters of stigma (family, friends, religious groups, healthcare providers, employers and prison employees. | [53], [66], [73] |  | [52], [72], [73] |  | [12], [17], [26], [44] |  |
| **Community** | **Stigma and discrimination** | Experiences of stigma | Negative experiences of stigma and discrimination | Stigma and Discrimination such as being laughed at, exclusion from activities and being fired) | [53], [73] |  | [52], [54], [64], [73] |  | [12], [44] |  |
| **Community** | **Stigma and discrimination** | Experiences of stigma | Negative experiences of stigma and discrimination | Negative public perceptions and past experiences of stigmatisation | [43], [50], [53] |  | [43] |  | [10] |  |
| **Institutional** | **Benefits of being in care** | Stigma management | Stigma easier to manage when in care rather than out of care | Stigma easier to manage when in care rather than out of care |  |  |  |  |  |  |
| **Institutional** | **Counselling practices and principles** | Addressing shared community uncertainties | Address issues that clinics and hospitals are where people come to die | Address issues that clinics and hospitals are where people come to die. Open communication that addresses fears and anxieties |  |  |  | [4] |  |  |
| **Institutional** | **Counselling practices and principles** | Awareness of literacy and language barriers | Language barriers | Communication barriers between providers and patients including language barriers and inability of provider to explain to patients. Aware of cultural and language differences (patients want pictures and visual aids) | [50], [66] |  | [54], [66] |  | [10], [26] | [2] |
| **Institutional** | **Counselling practices and principles** | Awareness of who is providing the counselling | Provided by peer support groups or case workers | Ongoing patient education from peer support groups or by caseworkers |  | [50] |  |  |  | [10] |
| **Institutional** | **Counselling practices and principles** | In depth pre and post counselling when testing | Assignment to case workers or peer navigators | Assignment to case workers or peer navigators helped to educate participants and address their anxieties and frustrations | [50] |  |  |  | [10] |  |
| **Institutional** | **Counselling practices and principles** | In depth pre and post counselling when testing | In depth pre and post counselling to mitigate shock and denial on diagnosis | In depth pre and post counselling to mitigate shock and denial on diagnosis | [73] | [42], [64] |  | [44] |  | [2], [44] |
| **Institutional** | **Counselling practices and principles** | In depth pre and post counselling when testing | Provided through pre and post-test counselling | Patient education provided through pre and post-test counselling improved linkage and gave patients a chance to ask questions |  | [50], [53] |  |  |  |  |
| **Institutional** | **Counselling practices and principles** | In depth pre and post counselling when testing | Providing more education about ART to enhance familiarity with medication | Providing more education about ART to enhance familiarity with medication |  | [73] |  | [2], [44] |  | [44] |
| **Institutional** | **Counselling practices and principles** | In depth pre and post counselling when testing | Receiving counselling and/or teaching | Receiving counselling and/or teaching |  |  |  | [1] |  |  |
| **Institutional** | **Counselling practices and principles** | Including patients beliefs and respecting cultural practices | Encouragement from a traditional birth attendant | Encouragement from a traditional birth attendant |  |  |  |  |  |  |
| **Institutional** | **Counselling practices and principles** | Including patients beliefs and respecting cultural practices | Integration of spirituality into HIV care | Integration of spirituality into HIV care |  |  |  | [12] |  |  |
| **Institutional** | **Counselling practices and principles** | Including patients beliefs and respecting cultural practices | Medical providers who incorporated traditional beliefs earned patients trust and were sought out in conjunction with traditional medicine | Medical providers who incorporated traditional beliefs earned patients trust and were sought out in conjunction with traditional medicine |  | [50] |  | [2] |  | [2], [10] |
| **Institutional** | **Counselling practices and principles** | Including patients beliefs and respecting cultural practices | Patients want to communicate with health care provider about the known and unknown side effects of traditional medicine on ART | Patients want to communicate with health care provider about the known and unknown side effects of traditional medicine on ART. Patients may hide that they are using traditional medicines |  |  |  | [2], [23] |  | [2] |
| **Institutional** | **Counselling practices and principles** | Including patients beliefs and respecting cultural practices | Respecting the place of traditional medication | Provider respecting the place of traditional medication |  | [50] |  |  |  | [2], [10] |
| **Institutional** | **Counselling practices and principles** | Poor counselling | Does not provide sufficient information | Patient dissatisfaction with HIV/ART information provided |  |  | [47], [70] |  |  |  |
| **Institutional** | **Counselling practices and principles** | Poor counselling | Poor counselling | Lack of or poor counselling: pre and post test | [53], [73] |  | [63], [72] |  | [17], [44] |  |
| **Institutional** | **Counselling practices and principles** | Types of narrative used by health care workers | Providers must frame ART as part of the daily routine | Providers must frame ART as part of the daily routine |  |  |  | [2] |  | [2] |
| **Institutional** | **Counselling practices and principles** | Types of narrative used by health care workers | Reminders of past illness | Reminders of past illness |  |  |  | [44] | [44] |  |
| **Institutional** | **Engagement with health care workers** | Disengaged and unsupportive relationships | Cannot build relationship due to high staff turnover | Inadequate number of skilled providers and high turnover rate at HIV clinics made it difficult for patients to form trusting relationships | [53], [73] |  | [63], [72] |  | [44] |  |
| **Institutional** | **Engagement with health care workers** | Disengaged and unsupportive relationships | No trust and confidentiality | Patient provider relationship (lack of trust/confidence) |  |  | [54], [70] |  | [2], [12] |  |
| **Institutional** | **Engagement with health care workers** | Disengaged and unsupportive relationships | No trust and confidentiality | Lack of confidence and trusting relationship in health care worker |  |  | [47], [48], [54], [65] |  | [12], [20] |  |
| **Institutional** | **Engagement with health care workers** | Disengaged and unsupportive relationships | Unequal power relationship | Unequal power relationship between patient and health care worker |  |  | [47], [48], [54], [65] |  | [12], [20] |  |
| **Institutional** | **Engagement with health care workers** | Frequency and duration of engagements | Directness, intensity, frequency, and extension of provider engagement with women. | The success of efforts to initiate and retain HIV-infected pregnant women in ANC and HIV care was shaped by the directness, intensity, frequency, and extension of provider engagement with women. |  | [46] |  | [28] |  | [6] |
| **Institutional** | **Engagement with health care workers** | Frequency and duration of engagements | More interactions with case managers | More encounters with case manager contributed to providing a holistic approach to care | [50] |  |  |  | [10] |  |
| **Institutional** | **Engagement with health care workers** | Supportive and collaborative relationships | Collaborative relationship | Good relationship with provider/collaborative relationship that provides support. This includes caring attitude, effective communication, frank and clear instructions, being responsive, being accessible, listening and showing respect. |  | [50] |  | [7], [11], [25], [41], [43] |  | [2], [10] |
| **Institutional** | **Engagement with health care workers** | Supportive and collaborative relationships | Open communication | Discuss decision to adhere with provider |  |  |  | [4], [25], [41] |  |  |
| **Institutional** | **Engagement with health care workers** | Supportive and collaborative relationships | Strong relationship | Having a strong relationship with a health care provider can mitigate conspiracy beliefs |  |  |  | [11] |  |  |
| **Institutional** | **Engagement with health care workers** | Supportive and collaborative relationships | Trust | Nursing and physician support to gain trust and overcome social isolation associated with stigma |  | [50], [53] |  | [16] |  | [10] |
| **Institutional** | **Health care worker recommendations and care** | Health care worker does not do timely tests or referrals | Health care workers not always drawing blood for CD4 counts | Health care workers not always drawing blood for CD4 counts leading to low uptake of CD4 count testing. |  |  |  |  |  |  |
| **Institutional** | **Health care worker recommendations and care** | Health care worker does not do timely tests or referrals | Provider fails to refer patient | Provider fails to refer patient and not sensitive to value of timely HAART initiation | [49] |  |  |  |  |  |
| **Institutional** | **Health care worker recommendations and care** | Health care worker does not provide holistic care | Engagement is not patient focused but rather on child | Health care workers focus on infant health instead of HIV related services for their own health | [49] |  |  |  |  |  |
| **Institutional** | **Health care worker recommendations and care** | Health care worker provides holistic care | Provider open and sensitive to needs of patient and other healthcare issues | Provider open and sensitive to needs of patient and other healthcare issues |  |  |  |  |  | [2] |
| **Institutional** | **Health care worker recommendations and care** | Provider input | Input from HCP | Input from health professionals for child adherence |  |  | [69] |  |  |  |
| **Institutional** | **Health care worker recommendations and care** | Provider input | Lack of doctors recommendation to continue ART | Lack of doctors recommendation to continue ART |  |  | [43] |  |  |  |
| **Institutional** | **Health care worker recommendations and care** | Provider input | Lack of doctors recommendation to initiate | Lack of doctors recommendation to initiate | [43], [64] |  |  |  |  |  |
| **Institutional** | **Health care worker recommendations and care** | Provider input | Quality of care in case management | Active referrals in health care and case management which offers a holistic approach |  | [50], [53] |  |  |  | [10] |
| **Institutional** | **Models of Care** | Adolescent services | Assisting with family disclosure | Clinic coordinates disclosure process with families |  | [73] |  | [44] | [44] |  |
| **Institutional** | **Models of Care** | Adolescent services | Offer health education | HCW offer HIV and SRH education |  | [73] |  | [44] | [44] |  |
| **Institutional** | **Models of Care** | Adolescent services | Offer health education | Full disclosure by HCW of HIV status and what ARVs are for. |  | [73] |  | [44] | [44] |  |
| **Institutional** | **Models of Care** | Adolescent services | Relatable counsellors | Nurses and counsellors are similar ages and gender as the patient |  | [73] |  | [44] | [44] |  |
| **Institutional** | **Models of Care** | Adolescent services | Relatable counsellors | HCW sensitized to work with HIV+ adolescents |  | [73] |  | [44] | [44] |  |
| **Institutional** | **Models of Care** | Adolescent services | Youth hours | Youth target hours and services |  | [73] |  | [44] | [44] |  |
| **Institutional** | **Models of Care** | Family driven care | Family driven treatment | Family focused care which enrols HIV positive patient and all HIV positive family members |  |  |  | [16] |  |  |
| **Institutional** | **Models of Care** | Gaps in referrals | Gaps in the referral processes | Gaps in the referral processes | [49] |  |  |  |  |  |
| **Institutional** | **Models of Care** | Gaps in referrals | Lack of continuity of care | Lack of continuity of care | [49] |  |  |  |  |  |
| **Institutional** | **Models of Care** | Hospital admission | Linking those admitted for comorbid diseases | Being admitted to hospital for TB treatment |  | [53] |  |  |  |  |
| **Institutional** | **Models of Care** | Integrated care | Integrated care will reduce patient burden | Integrated care will reduce patient burden: this includes electronic health records resulted in efficiency and reduced processing time and receipt of lab results. |  | [50] |  |  |  | [10] |
| **Institutional** | **Models of Care** | Integrated mental health care | Integrated mental health care | Psychiatric care services |  |  | [54] | [14] |  | [12] |
| **Institutional** | **Models of Care** | Integrated mental health care | Mental health included in care | Treatment of depression and anxiety related to diagnosis |  |  |  | [16] |  |  |
| **Institutional** | **Models of Care** | Integrated mental health care | Mental health included in care | Provider addressing issues of depression through social support |  |  |  | [12] |  |  |
| **Institutional** | **Models of Care** | Integrated mental health care | Mental health included in care | Diagnosing and managing depressive symptoms associated with HIV diagnosis and HIV related stigma |  | [73] |  | [44] | [44] |  |
| **Institutional** | **Models of Care** | Integrated mental health care | Mental health included in care | Early intervention through regular mental health assessment by providers |  | [64], [73] |  | [44] | [44] |  |
| **Institutional** | **Models of Care** | Involving patients as peer facilitators | Participating as a peer facilitator gave meaning and promoted adherence | Participating as a peer facilitator gave meaning and promoted adherence |  | [73] |  | [44] | [44] |  |
| **Institutional** | **Models of Care** | Lack of integrated care | Does not consider structural factors in care | Health care workers focusing exclusively on biomedical aspects and fail to address social factors such as care-seeking, poverty and food insecurity | [49] |  |  |  |  |  |
| **Institutional** | **Models of Care** | Lack of integrated care | Does not include mental health care | Provider not addressing issues of depression |  |  |  |  |  |  |
| **Institutional** | **Models of Care** | Lack of integrated care | Lack of integrated care which increase patient burden | Lack of integrated care which increase patient burden |  |  |  |  |  |  |
| **Institutional** | **Models of Care** | Male services | Male friendly services | Men want male-friendly health clinics and did not want to receive care in an antenatal clinic. |  |  | [42], [63] |  |  | [2] |
| **Institutional** | **Models of Care** | Mobile and home visits | Home visits and mobile care | Being visited at home contributed to visibility of infection and potential involuntary disclosure | [53] |  | [63] |  |  |  |
| **Institutional** | **Models of Care** | Mobile and home visits | Home visits and mobile care units | Mobile care units |  |  |  |  |  | [2] |
| **Institutional this row must be added to tables** | **Models of Care** | PMTCT, ANC and HIV Integration | Completion of PMTCT pre-delivery | Successful completion of PMTCT pre delivery |  |  |  |  | [10], [44] |  |
| **Institutional** | **Models of Care** | PMTCT, ANC and HIV Integration | Enrolment in ART pre delivery | Previous experience with PMTCT |  |  |  | [27] |  |  |
| **Institutional** | **Models of Care** | PMTCT, ANC and HIV Integration | Delivering HIV in the context of ANC | Dropout from and delays in the maternal ART cascade are driven by problems in delivering HIV services in the context of ANC programs. |  |  | [46] |  | [6] |  |
| **Institutional** | **Models of Care** | PMTCT, ANC and HIV Integration | Enrolment in ART postdelivery | Enrolment in Art post pre delivery |  |  |  |  |  |  |
| **Institutional** | **Models of Care** | PMTCT, ANC and HIV Integration | Enrolment in ART pre delivery | Enrolment in Art pre delivery |  |  | [55] |  |  | [55] |
| **Institutional** | **Models of Care** | PMTCT, ANC and HIV Integration | Escorts for women between ANC and HAART services allowing pregnant women to bypassing queues | Escorts for women between ANC and HAART services allowing pregnant women to bypassing queues |  |  |  |  |  |  |
| **Institutional** | **Models of Care** | PMTCT, ANC and HIV Integration | Gaps between ANC/PMTCT and HIV services and dropout along maternal ART cascade | Gaps between ANC/PMTCT and HIV services and dropout along maternal ART cascade | [46], [49] |  | [46] |  | [6] |  |
| **Institutional** | **Models of Care** | PMTCT, ANC and HIV Integration | Integrated models of care | Provision of HAART services on-site only once per week in the integrated model may be insufficient to affect HAART initiation during pregnancy |  |  |  |  |  |  |
| **Institutional** | **Models of Care** | PMTCT, ANC and HIV Integration | Late disengagement (within 41 days of delivery). | Women who disengaged from HIV services in the 41 days before delivery (i.e. late disengagement) were more likely to be lost to follow up postpartum when compared to women who stayed until delivery |  |  |  |  | [55] |  |
| **Institutional** | **Models of Care** | PMTCT, ANC and HIV Integration | Late or low attendance at ANC | Late or low attendance at ANC | [55] |  |  |  | [55] |  |
| **Institutional** | **Models of Care** | PMTCT, ANC and HIV Integration | Maternal ART services struggle to retain women in care and involve partners during postpartum when women are ART ineligible or declined ART | Maternal ART services struggle to retain women in care and involve partners during postpartum when women are ART ineligible or declined ART | [46] |  |  |  |  |  |
| **Institutional** | **Models of Care** | PMTCT, ANC and HIV Integration | Maternal ART under prioritised in ANC. PMTCT and HIV programmes. | Maternal ART under prioritised in ANC. PMTCT and HIV programmes. | [46] |  | [46] |  | [6] |  |
| **Institutional** | **Models of Care** | PMTCT, ANC and HIV Integration | Multii pronged and multileveled interventions | Interventions typically move beyond integrating discrete elements of service delivery, and instead, provide multi-pronged and multi-levelled interventions in the broader health system to support maternal ART initiation, retention and adherence. |  | [64] |  |  |  |  |
| **Institutional** | **Models of Care** | PMTCT, ANC and HIV Integration | PMTCT and ANC | First pregnancy registration |  |  |  |  |  | [55] |
| **Institutional** | **Models of Care** | PMTCT, ANC and HIV Integration | Screening for HAART eligibility and initiation of HAART within pregnancy related services | Screening for HAART eligibility and initiation of HAART within pregnancy related services |  | [49] |  |  |  |  |
| **Institutional** | **Models of Care** | PMTCT, ANC and HIV Integration | Strengthen linkages between PMTCT and HAART services | Strengthen linkages between PMTCT and HAART services |  | [49] |  |  |  |  |
| **Institutional** | **Perception of health care workers** | Expectations of providers | To provide care and instruct patient | Unquestioning acceptance of provider’s attitudes towards their health can affect experiences of health services. |  |  |  | [11] |  |  |
| **Institutional** | **Perception of health care workers** | Expectations of providers | Want to feel safe with providers | Want to feel safe with providers |  |  |  | [2] |  | [2] |
| **Institutional** | **Perception of health care workers** | Negative perceptions of health care workers | Negative perceptions of providers skills | Negative perceptions of providers skills | [43], [53] |  | [43] |  |  | [2] |
| **Institutional** | **Perception of health care workers** | Negative perceptions of health care workers | Poorly trained health care workers | Weak training and supervision of healthcare workers |  |  | [46] |  | [6] |  |
| **Institutional** | **Perception of health care workers** | Negative perceptions of health care workers | Unable to gain attention from staff | Unable to gain attention from staff |  |  | [47] |  |  |  |
| **Institutional** | **Perception of health care workers** | Perception of health care workers | Patients perception of provider skill | Patients perception of provider skill |  |  |  |  | [2] | [2] |
| **Institutional** | **Perception of health care workers** | Positive perceptions of health care workers | Consistent provider | Consistent provider |  |  |  |  |  | [2] |
| **Institutional** | **Perception of health care workers** | Positive perceptions of health care workers | Support mitigates lack of other support | Health care workers as proxy for lack of family support |  | [50] | [63] |  |  | [10] |
| **Institutional** | **Perception of health care workers** | Positive perceptions of health care workers | Supportive | Supportive health care workers |  |  | [63] | [1] |  | [55] |
| **Institutional** | **Perception of health care workers** | Positive perceptions of health care workers | Well trained | Well trained health care staff |  |  | [63] |  |  |  |
| **Institutional** | **Relocation to other facility** | Transfers and Relocation | Change of service provider | Change of service provider due to relocation. Treatment may be interrupted. | [53] |  | [64] |  |  |  |
| **Institutional** | **Relocation to other facility** | Transfers and Relocation | Request to be transferred to other clinic | Patient request to be transferred to other clinic | [53] |  |  |  |  |  |
| **Institutional** | **Relocation to other facility** | Transfers and Relocation | Travelling interrupting treatment | Limited medication availability during migration periods |  |  | [66] |  |  |  |
| **Institutional** | **Service delivery** | Clinic times | Inconvenient clinic times and time needed off work and school | Inconvenient clinic times and no time to go to the clinic/ limited availability of services | [50] |  | [41], [52], [54], [65] |  | [2], [10], [12], [17] |  |
| **Institutional** | **Service delivery** | Clinic times | Long waiting times and queues | Unable to get to clinic due to work constraints. | [53] |  | [47] |  | [2], [17] |  |
| **Institutional** | **Service delivery** | Clinic times | Short waiting time and sufficient time for consultations | Sufficient time for consultations |  |  |  | [1], [41] |  |  |
| **Institutional** | **Service delivery** | Drug and test resources | Drug and test stock outs | Erratic clinic drug supply | [53] |  | [42], [46], [47], [48], [54], [61], [65] |  | [2], [6] |  |
| **Institutional** | **Service delivery** | Drug and test resources | Drug and test stock outs | Prison officials to ensure that there is no interruption in medications |  |  |  |  |  |  |
| **Institutional** | **Service delivery** | Drug and test resources | Laboratory service failure | Laboratory service failure | [53] |  | [42] |  |  | [2] |
| **Institutional** | **Service delivery** | Drug and test resources | Limit on amount of medication given (limit of a 1-month supply) | Limit on amount of medication given (limit of a 1-month supply) |  |  | [48] |  |  |  |
| **Institutional** | **Service delivery** | Drug and test resources | Medication available | Medications that are easily available |  |  |  | [16] |  |  |
| **Institutional** | **Service delivery** | Drug and test resources | Pharmacy problems | Pharmacy problems: medicine not dispensed correctly |  |  | [55] |  |  |  |
| **Institutional** | **Service delivery** | Lack of privacy | Lack of privacy | Fear of breach of confidentiality in health centre | [50]. [53] |  | [55] |  | [10], [12], [55] |  |
| **Institutional** | **Service delivery** | Lack of privacy | Long queues may risk patient being seen and stigmatised. | Long waiting times to see health care provider and clinic visits can take up to a full day. | [50], [53], [55], [60], [73] | [60] | [42], [46], [47], [48], [54], [61], [65] |  | [2], [10], [12], [55], [17], [44] |  |
| **Institutional** | **Service delivery** | Lack of privacy | No privacy | Flaws in the healthcare system such as forced disclosure people delayed decision making |  |  | [63] |  |  |  |
| **Institutional** | **Service delivery** | Lack of privacy | No privacy | Perceived or experienced breaches of confidentiality by health care workers | [50], [60] | [60] | [42] |  | [2], [10] |  |
| **Institutional** | **Service delivery** | Negative experiences at the clinic | Negative experiences at health care facility | Negative health services experiences such as confusion with regards to referral cases and not knowing who needs HIV care | [50], [53], [60] |  | [54] |  | [10], [12] |  |
| **Institutional** | **Service delivery** | Negative experiences at the clinic | Negative experiences of patients HIV testing | Patients experiences of HIV testing | [50], [20] | [50], [20] |  |  | [10] | [10] |
| **Institutional** | **Service delivery** | Negative experiences at the clinic | Negative treatment and interactions with health care staff | Negative attitude, treatment and interactions with health care staff | [49], [53], [55], [58], [66], [73] |  | [47], [48], [54], [55], [65], [66], [73] | [44] | [55], [17], [20], [26], [44] | [44] |
| **Institutional** | **Service delivery** | Physical clinic environment | Comfort and convenience of services | Comfort and conveniences of services |  |  |  |  |  | [2] |
| **Institutional** | **Service delivery** | Physical clinic environment | Confusing set up at clinic | Confusing set up at clinic | [53] |  | [63] |  |  |  |
| **Institutional** | **Service delivery** | Physical clinic environment | General dislike | Dislike of health facilities | [49], [53] |  |  |  |  |  |
| **Institutional** | **Service delivery** | Physical clinic environment | Overcrowding | No privacy at clinic due to crowded pharmacies, consultations with multiple patients and lost files | [73] |  | [54], [63] |  | [17], [44] |  |
| **Institutional** | **Service delivery** | Physical clinic environment | Physical clinic environment | Physical clinic environment |  |  |  |  |  |  |
| **Institutional** | **Service delivery** | Physical clinic environment | Providing disability accommodations | Providing disability accommodations |  |  |  |  |  | [2] |
| **Institutional** | **Service delivery** | Physical clinic environment | Type of facility | Health centre versus hospital care |  |  | [54] | [1] |  |  |
| **Institutional** | **Service delivery** | Physical clinic environment | Welcoming atmosphere at clinic | Staff member to welcome and assist new patients |  | [53] |  |  |  |  |
| **Institutional** | **Service Delivery** | Scheduled appointments | Difficulty getting appointments | Difficulty getting appointments |  |  | [48], [63] |  | [12] |  |
| **Institutional** | **Service Delivery** | Scheduled appointments | Missing scheduled visits | Missing scheduled visits |  |  | [54], [67] |  |  |  |
| **Institutional** | **Service Delivery** | Scheduled appointments | Patient has difficulty scheduling appointments | Difficulty scheduling | [55] |  | [55] |  | [55] |  |
| **Institutional** | **Service Delivery** | Scheduled appointments | Repeated missed appointments | Repeated missed appointments |  |  | [41] |  |  |  |
| **Institutional** | **Service Delivery** | Scheduled appointments | Same day appointments | Same day appointments |  |  |  |  |  | [2] |
| **Institutional** | **Service Delivery** | Scheduled appointments | Scheduling difficulties | Scheduling difficulties. |  |  | [46] |  | [2], [6] |  |
| **Institutional** | **Service Delivery** | Scheduled appointments | Scheduling during school holidays | Scheduling appointments during school holidays |  |  |  | [1] |  |  |
| **Institutional** | **Service Delivery** | Scheduled appointments | Scheduling too far in advance | Medical appointments too far into the future |  |  |  |  | [10] |  |
| **Institutional** | **Service delivery** | Staff turnover | Shortages of staff and supplies to service patients | Shortages of skilled staff and supplies to service patients | [49], [50], [53], [60], [73] | [60] | [63], [73] |  | [10], [44] |  |
| **Institutional** | **Service delivery** | Staff turnover | Staff shortages and high turnover | Staff shortages and high turnover | [53] |  | [46] |  | [6] |  |
| **Institutional** | **Service Delivery** | Weak systems and protocols | Poor patient tracking and weak information systems | Poor follow-up and tracing of patients and weak information systems |  |  | [46] |  | [2], [6] |  |
| **Institutional** | **Service Delivery** | Weak systems and protocols | Using updated protocols | Failure to keep up with rapidly changing treatment protocols and referral procedures. |  |  | [46] |  | [2], [6] |  |
| **Institutional** | **Service Delivery** | Weak systems and protocols | Weak information systems | Each information systems |  |  | [46] |  | [2], [6] |  |
| **Institutional** | **Stigma and health care engagement** | Favouritism | Stigma and discrimination | Favouritism | [53] |  |  |  |  |  |
| **Institutional** | **Stigma and health care engagement** | Gender and sexuality bias | Judgement based on gender | Health care workers attitudes towards gender norms. In some countries women’s HIV status were due to male partners sexual needs and in other women were seen as hypersexual if positive |  |  |  |  |  |  |
| **Institutional** | **Stigma and health care engagement** | Gender and sexuality bias | Sexual orientation | Stigma and discrimination of patients based on sexual orientation | [64] |  | [56] |  |  |  |
| **Institutional** | **Stigma and health care engagement** | Gender and sexuality bias | Sexual orientation | Transgender women are unable to be reissued new identity document and therefore not able to link to care | [60] |  |  |  |  |  |
| **Institutional** | **Stigma and health care engagement** | Gender and sexuality bias | Sexual orientation | Transgender women no longer included on family record and therefore unable to get insurance and then access services | [60] |  |  |  |  |  |
| **Institutional** | **Stigma and health care engagement** | Gender bias | Gender biases and norms | Women cannot seek care without husbands consent | [50] |  |  |  | [10] |  |
| **Institutional** | **Stigma and health care engagement** | Gender bias | Gender biases and norms | Men going and waiting in clinics perceived as a sign of weakness and absence from work reduces ability to be a breadwinner for the family. | [50] |  |  |  | [10] |  |
| **Institutional** | **Stigma and health care engagement** | HIV related stigma | Discriminating against poverty and HIV status | Not helping patients because they are poor and do not have much time to live. HIV seen as a disease of poverty. |  |  | [56], [63] |  |  |  |
| **Institutional** | **Stigma and health care engagement** | HIV related stigma | Stigma and discrimination | HIV-related stigma | [53] |  | [52], [67], [72] |  | [12], [17] |  |
| **Institutional** | **Stigma and health care engagement** | HIV related stigma | Stigma and discrimination | Insensitivity and judgement by the provider | [53] |  |  |  | [12], [17] |  |
| **Institutional** | **Stigma and health care engagement** | HIV related stigma | Stigma and discrimination | Limited sensitisation and high levels of stigma of providers and clients on the importance of CD4 count testing. |  |  |  |  |  |  |
| **Institutional** | **Stigma and health care engagement** | HIV related stigma | Stigma and discrimination | Perceptions about the healthiness of pregnant women. |  |  |  |  |  |  |
| **Institutional** | **Stigma and health care engagement** | HIV related stigma | Stigma and discrimination | Persistent condescending attitudes | [50] |  |  |  | [10] |  |
| **Institutional** | **Stigma and health care engagement** | HIV related stigma | Stigma and discrimination | Provider is sensitive and non-judgemental about other issues such as substance abuse | [50] |  |  |  | [10] |  |
| **Institutional** | **Stigma and health care engagement** | Occupational stigma | Stigma and discrimination | Stigma (sex workers publically called out over intercom to move to back of the line (study 18) |  |  |  |  |  |  |
| **Institutional** | **Stigma and health care engagement** | Patient anticipates stigma | Stigma and discrimination | Perception of racism and discrimination in health care setting | [58] |  | [51] |  |  |  |
| **Institutional** | **Stigma and health care engagement** | Patient anticipates stigma | Stigma and discrimination | Actual or anticipated stigma when seen accessing clinic | [50], [53], [73] |  |  |  | [10], [17], [44] |  |
| **Structural** | **Financial costs for care** | Free ART still has costs | Cost of ART | Mandatory counselling is not free | [73] |  | [73] |  | [10], [17], [44] |  |
| **Structural** | **Financial costs for care** | Free ART still has costs | Cost of ART | Pay for HIV diagnosis and high administration costs including opportunistic infection (but not TB) |  |  | [60] |  |  |  |
| **Structural** | **Financial costs for care** | Free ART still has costs | Free ART still has costs | Costs associated with treatment (even free ART becomes expensive when thinking of the loss of wages and the cost to travel to the clinic) | [43], [49] |  | [42], [43], [47], [54], [55], [56], [60], [63], [64], [72] | [7] | [18], [20] |  |
| **Structural** | **Financial costs for care** | Free ART still has costs | Lack of childcare | Cost of childcare when visiting clinic |  |  |  |  | [2], [12] |  |
| **Structural** | **Financial costs for care** | Free ART still has costs | Loss of grants | Disability grant loss/livelihood |  |  | [47] |  |  |  |
| **Structural** | **Financial relief for care** | Grants | Food supplementation | Provision of food |  | [45] |  | [1] |  |  |
| **Structural** | **Financial relief for care** | Grants | Incentives provided | Food, soap, travel reimbursement, skills training |  |  |  | [44] |  | [44] |
| **Structural** | **Financial relief for care** | Grants | Free ART Treatment | Free ART Treatment |  |  |  | [7] |  |  |
| **Structural** | **Financial relief for care** | Grants | Provision of vouchers for transport | Provision of vouchers for transport | [53] | [53] |  | [1] |  |  |
| **Structural** | **Financial relief for care** | Grants | Provision of vouchers to reduce cost of medication and cut down regimen to reduce cost | Provision of vouchers to reduce cost of medication and cut down regimen to reduce cost |  |  |  | [2], [14] |  |  |
| **Structural** | **Healthcare policies** | Access and eligibility policies | Access laws which send people to their birthplace for medication | Discouraged by access laws which send people to their birthplace for medication |  |  | [42] |  | [2] |  |
| **Structural** | **Healthcare policies** | Access and eligibility policies | Policy limitations | Policies directed as specific populations make it hard to access care such as threats of criminalisation for transgender, sex workers and drug users and deportation threats for immigrants | [50], [58] |  |  |  | [10], [18] |  |
| **Structural** | **Healthcare policies** | Access and eligibility policies | Policy Limitations | Policies not allowing women to seek care without the consent of her husband |  |  |  |  |  |  |
| **Structural** | **Healthcare policies** | Health insurance | Health care insurance policies | Health insurance concerns state, requirements, policies, practices and limitations of one’s health insurance and its coverage for HIV medications | [50] |  | [48] |  | [10] |  |
| **Structural** | **Healthcare policies** | Policies are hard to understand | Patients have a hard time understanding the limitations and eligibility rules as well as the laws and policies that govern adherence and linkage | Discouraged by lack of understanding of laws and policies that govern adherence and linkage |  |  | [42] |  | [2] |  |
| **Structural** | **Income and food security** | Income and financial status | Food Insecurity | Hunger and food insecurity (also side effects from meds such as gastrointestinal upset) and fear that drugs will increase appetite | [45], [53], [58] |  | [47], [48], [54], [55], [58], [63], [65], [67], [70], [72] | [7] |  |  |
| **Structural** | **Income and food security** | Income and financial status | High income | Higher income | [44] |  |  | [55], [43] |  | [55] |
| **Structural** | **Income and food security** | Income and financial status | Low income and poverty | Low income/poverty and difficult material circumstances | [43], [53], [73] |  | [41], [42], [48], [52], [54], [56], [65], [67], [70], [71], [72], [73] |  | [12], [44] | [55], [17], [20] |
| **Structural** | **Living conditions and context** | Housing | Stable housing | Unstable and inadequate or absent housing | [43] |  | [43], [48], [52], [54], [65], [68], [70] |  | [12] |  |
| **Structural** | **Transport and distance to clinic** | Transport and distance to clinic | Geographic location of the facility | Rural living and difficulty accessing comprehensive care | [59] |  | [41], [47], [54], [59], [60], [61], [72] | [1] | [12], [44] | [26] |
| **Structural** | **Transport and distance to clinic** | Transport and distance to clinic | Logistical challenges in accessing care | Transport to access services (including no money to pay for transport, negotiating cost of transport over other household costs, poor road conditions, difficulty accessing reliable transport) | [53], [55], [58], [59], [73] |  | [41], [42], [47], [52], [54], [55], [59], [60], [61], [63], [65], [67], [71], [72], [73] |  | [12], [55], [17], [19], [20], [44] | [19] |
| **Structural** | **Transport and distance to clinic** | Transport and distance to clinic | Safety when traveling to clinic | Escorts to clinic |  | [53], [66] |  |  |  |  |
